# Organized crime groups: A systematic review of individual‐level risk factors related to recruitment

**DOI:** 10.1002/cl2.1218

**Published:** 2022-02-11

**Authors:** Francesco Calderoni, Tommaso Comunale, Gian Maria Campedelli, Martina Marchesi, Deborah Manzi, Niccolò Frualdo

**Affiliations:** ^1^ Transcrime Università Cattolica del Sacro Cuore Milan Italy; ^2^ Security Program Center for the Study of Democracy Sofia Bulgaria; ^3^ Dipartimento di Sociologia e Ricerca Sociale Università degli Studi di Trento Trento Italy; ^4^ Department of Sociology Northwestern University Evanston Illinois USA

## Abstract

**Background:**

Studies from multiple contexts conceptualize organized crime as comprising different types of criminal organizations and activities. Notwithstanding growing scientific interest and increasing number of policies aiming at preventing and punishing organized crime, little is known about the specific processes that lead to recruitment into organized crime.

**Objectives:**

This systematic review aimed at (1) summarizing the empirical evidence from quantitative, mixed methods, and qualitative studies on the individual‐level risk factors associated with the recruitment into organized crime, (2) assessing the relative strength of the risk factors from quantitative studies across different factor categories and subcategories and types of organized crime.

**Methods:**

We searched published and unpublished literature across 12 databases with no constraints as to date or geographic scope. The last search was conducted between September and October 2019. Eligible studies had to be written in English, Spanish, Italian, French, and German.

**Selection Criteria:**

Studies were eligible for the review if they:

- Reported on organized criminal groups as defined in this review.
- Investigated recruitment into organized crime as one of its main objectives.
- Provided quantitative, qualitative, or mixed methods empirical analyses.
- Discussed sufficiently well‐defined factors leading to recruitment into organized crime.
- Addressed factors at individual level.
- For quantitative or mixed‐method studies, the study design allowed to capture variability between organized crime members and non‐members.

**Data Collection and Analysis:**

From 51,564 initial records, 86 documents were retained. Reference searches and experts' contributions added 116 additional documents, totaling 202 studies submitted to full‐text screening. Fifty‐two quantitative, qualitative, or mixed methods studies met all eligibility criteria. We conducted a risk‐of‐bias assessment of the quantitative studies while we assessed the quality of mixed methods and qualitative studies through a 5‐item checklist adapted from the CASP Qualitative Checklist. We did not exclude studies due to quality issues. Nineteen quantitative studies allowed the extraction of 346 effect sizes, classified into predictors and correlates. The data synthesis relied on multiple random effects meta‐analyses with inverse variance weighting. The findings from mixed methods and qualitative studied were used to inform, contextualize, and expand the analysis of quantitative studies.

**Results:**

The amount and the quality of available evidence were weak, and most studies had a high risk‐of‐bias. Most independent measures were correlates, with possible issues in establishing a causal relation with organized crime membership. We classified the results into categories and subcategories. Despite the small number of predictors, we found relatively strong evidence that being male, prior criminal activity, and prior violence are associated with higher odds of future organized crime recruitment. There was weak evidence, although supported by qualitative studies, prior narrative reviews, and findings from correlates, that prior sanctions, social relations with organized crime involved subjects, and a troubled family environment are associated with greater odds of recruitment.

**Authors' Conclusions:**

The available evidence is generally weak, and the main limitations were the number of predictors, the number of studies within each factor category, and the heterogeneity in the definition of organized crime group. The findings identify few risk factors that may be subject to possible preventive interventions.

## 1 PLAIN LANGUAGE SUMMARY

Evidence suggests individual‐level factors predict recruitment into organized crime.

### 1.1 The review in brief

There is relatively strong evidence that being male and having committed prior criminal activity and violence are associated with future organized crime recruitment. There is weak evidence that prior sanctions, social relations with organized crime‐involved subjects and a troubled family environment are associated with recruitment.

### 1.2 What is this review about?

This systematic review examines what individual‐level risk factors are associated with recruitment into organized crime.

Despite the increase of policies addressing organized crime activities, little is known about recruitment. Existing knowledge is fragmented and comprises different types of organized criminal groups.

Recruitment refers to the different processes leading individuals to stable involvement in organized criminal groups, including mafia, drug trafficking organizations, adult gangs and outlawed motorcycle gangs. This systematic review excludes youth (street) gangs, prison gangs and terrorist groups.

**What is the aim of this review?**

This Campbell systematic review examines individual‐level risk factors related to recruitment into organized crime groups. The review summarizes evidence from 52 studies, including 19 quantitative studies, 28 qualitative studies, and five studies that apply mixed methods.

### 1.3 What studies are included?

This review examines empirical studies of sufficiently well‐defined factors associated with involvement in organized crime. Nineteen quantitative, 28 qualitative, and five mixed‐methods studies met all eligibility criteria and were included in the systematic review.

Quantitative studies had to compare data on organized crime members and non‐organized crime members. The meta‐analyses of risk factors associated with recruitment focused on the evidence from 19 quantitative studies.

### 1.4 What are the main findings of this review?

All the included studies presented some important methodological weaknesses. Risk factors were divided into predictors (when the factors occurred before recruitment into organized crime) or correlates (factors measured at the same moment or subsequent to recruitment). Most risk factors were correlates, which causes problems in establishing a causal relation with recruitment into organized crime.

Despite the small number of predictors, there is relatively strong evidence that being male and having committed prior criminal activity and violence are associated with higher probability of future organized crime recruitment.

There is weak evidence, although supported by qualitative studies, prior narrative reviews and findings from correlates, that prior sanctions, social relations with organized crime‐involved subjects and a troubled family environment are associated with greater likelihood of recruitment.

Evidence from correlates indicates that higher levels of education are associated with lower probability of organized crime recruitment Conversely, low self‐control, sanctions, a troubled family environment, violence, being in a relationship, and poor economic conditions are associated with a higher likelihood of involvement in organized crime. These findings, however, should not be confused with predictors, due to difficulties in establishing a clear causal relation between the correlates and organized crime recruitment.

### 1.5 What do the findings of this review mean?

The available evidence is weak. There was a small number of studies for most factor categories. Most quantitative studies were from the United States and the United Kingdom. Thus, it may be difficult to apply the findings to organized crime groups in other countries.

Furthermore, this review encompassed a variety of organized crime groups. Different risk factors may drive recruitment into different types of groups, which may affect the quality of the evidence. Notwithstanding these limitations, the findings identify risk factors that may point to areas for possible interventions.

### 1.6 How up‐to‐date is this review?

The review authors searched for studies up to October 2019.

## 2 BACKGROUND

### 2.1 The issue: Organized crime

The differences in the study of organized crime have influenced the challenge of defining and conceptualizing the concept itself, which has long been debated among researchers (Finckenauer, 2005; Hagan, 1983, 2006; Smith, 1975; Von Lampe, 2008, 2016). The term ‘organized crime’ first emerged in the late 19th century in the United States, but its meaning varied over the past century (Fijnaut & Paoli, 2004; Kenney & Finckenauer, 1994; Woodiwiss, 2001). Organized crime was first associated with activities protected by public officials (e.g., prostitution and racketeering), and subsequently also with fraud and extortion (Woodiwiss, 2003). In the 1950s, the concept evolved toward the “alien conspiracy” approach, due to the influence of the media and US institutions such as the Kefauver Committee. The alien conspiracy approach contended that organized crime was predominantly composed of foreign, especially Italian immigrants, criminals organized in formally hierarchical groups and dominating profitable illegal markets such as gambling, prostitution, and narcotics (Cressey, 1969; Smith, 1976). By the 1960s, several scholars rejected this approach, suggesting that organized crime mostly revolves on social connections, patron‐client relationships and the social organization of the underworld (Albini, 1971; Blok, 1974; Hess, 1970/1973; Ianni & Reuss‐Ianni, 1972). In the 1970s, the paradigm of the “illegal enterprise” replaced the alien conspiracy, shifting the focus on the role of criminal organizations in supplying illegal products and services (Arlacchi, 1983; Block, 1980/1983; Reuter, 1983; Smith, 1975). A particular theoretical interpretation contended that organized crime specializes in the supply of illegal protection (Gambetta, 1993; Varese, 2005, 2010). The economic perspective became equally predominant in Europe, which had largely remained out of the debate until the mid‐1970s (Fijnaut & Paoli, 2004; Paoli & Vander Beken, 2014). Ever since, the organized crime label has become increasingly popular all over the world, and authors have proposed a variety of definitions (Von Lampe, 2016).

Notwithstanding several shifts in the conceptualization of organized crime, the theoretical debate has so far failed to achieve an agreement on its definition. Several studies reviewed existing definitions to identify common dimensions (Finckenauer, 2005; Hagan, 1983, 2006; Maltz, 1976; Van Duyne, 2004; Varese, 2010, 2017; Von Lampe et al., 2006). These efforts yielded several conclusions. First, the problematic element in the concept of organized crime is the term “organized” and its operationalization. Consequently, most interpretations attempted to distinguish organized crime from “crimes that are organized,” that is, complex criminal activities requiring important levels of coordination among the participants but lacking the additional features of organized crime (Finckenauer, 2005; Hagan, 1983, 2006). Second, it is important to distinguish between the characteristics of the group and those of the crimes and activities it perpetrates (Paoli & Vander Beken, 2014; Reuter & Paoli, 2020; Von Lampe, 2016). When considering the groups, organized crime should be conceptualized as an ordinal rather than a binary category, with groups exhibiting different levels of intensity of specific characteristics within a continuum rather than groups having/not having specific elements defined by an arbitrary threshold (Hagan, 1983, 2006, p. 200; Paoli & Vander Beken, 2014). Third, notwithstanding the heterogeneity in the literature, most contributions identify a core set of dimensions of organized crime and namely: (a) its nonideological nature, that is, criminal organizations do not have political or religious motivations; (b) organized crime is profit oriented, aiming to achieve illegal profits; (c) continuity, that is, organized crime aims at the repeated commission of an indeterminate number of crimes; (d) organized crime uses threat and violence to perpetrate crimes; (e) organized crime has an internal organization, not necessarily a formal hierarchy, such as a division of tasks (f) organized crime is embedded in the surrounding social environment and actively interacts with it, for example, by corrupting public officials, providing extra‐legal protection, controlling legal activities, influencing politics (Reuter & Paoli, 2020; Varese, 2017). While the attempts to define organized crime share important similarities, some scholars have contended that the very concept of organized crime is problematic and the result of a social construct rather than a useful tool for empirical analysis (Van Duyne, 1995; Von Lampe et al., 2006). Notwithstanding these criticisms, organized crime has remained a popular concept in the scholarly literature, in the policy debate, and in the public attention.

This systematic review relies on the definition provided by Article 2 of the United Nations Convention against Transnational Organized Crime (United Nations, 2000):

“Organized criminal group” shall mean a structured group of three or more persons, existing for a period of time and acting in concert with the aim of committing one or more serious crimes or offences established in accordance with this Convention, to obtain, directly or indirectly, a financial or other material benefit.

The UN Convention definition is the result of international efforts in stepping up the fight against criminal organizations in the 1990s. Although it has been criticized for being excessively vague (Calderoni, 2012; McClean, 2007; Paoli, 2014), the UN definition suits the purposes of this systematic review by providing a broad, inclusive, operationalization of organized crime. This allows for more flexibility when searching for potentially relevant studies, encompassing a variety of organized criminal groups as the mafias, drug trafficking groups, and some criminal gangs.

### 2.2 Recruitment into organized crime

This systematic review aims at summarizing and consolidating the knowledge of the factors associated with recruitment into organized crime. Entering into an organized criminal group is a significant step in the life of an individual, constituting a negative turning point in life and determining an increase in the risk of offending, harm, and incarceration (Fuller et al., 2019; Laub & Sampson, 1993; Melde & Esbensen, 2011; Morgan et al., 2020). Furthermore, individuals involved in criminal organizations are responsible for serious crimes with wide‐ranging societal implications, including loss of lives, economic impact, and politics (Lavezzi, 2008; Pinotti, 2015). For this review, recruitment refers to the different processes leading individuals to the stable involvement into organized criminal groups. This interpretation comprises individuals deliberately choosing to participate in criminal organizations, but also subjects socialized into criminal groups through family, friendship, and community relations. It also includes, but it is not limited to, the processes of formal or ritual affiliation exhibited by some criminal organizations (which would unnecessarily restrict the scope of the review, were they adopted as operational definition). Conversely, this definition excludes individuals occasionally cooperating or co‐offending with members of organized criminal groups, as they lack stability over time.

### 2.3 The risk factors for recruitment into organized crime

For several years, the field of organized crime studies has remained at the margins of the most popular debates in criminology (Posick & Rocque, 2018). For example, the important dispute on the individual or social causes of criminal behavior has rarely touched on what causes people to join organized crime groups. Some of the most popular contributions to the debate make only a quick reference to criminal organizations, in some case contending that “there is no need for theories designed specifically to account for … organized crime” (Gottfredson & Hirschi, 1990, p. 214).

At the same time, the literature on organized crime has disregarded the contributions of important theoretical and empirical discussions in the discipline. In general, however, organized crime studies relied on a few seminal studies arguing that the social environment plays a central role in the involvement of individuals in criminal organizations, with limited attention to individual characteristics (Albini, 1971; Block, 1980/1983; Ianni & Reuss‐Ianni, 1972). Furthermore, most studies have emphasized the role of the social environment at a meso‐level, contending that factors such as trust, social relations, kinship, and cultural/symbolic elements are crucial for the formation and persistence of criminal groups (Gambetta, 1993; Kleemans & Van de Bunt, 1999; Paoli, 2003). Possibly due to the lack of data, very rarely studies have directly addressed the factors leading to recruitment or involvement into organized crime at the individual level (Von Lampe, 2016). As a result, among earlier contributions, information on the processes that lead individuals to join organized criminal groups is largely dispersed.

Only in recent years a few studies have gained access to better information on individual members of organized crime groups. This enabled scholars to examine the factors influencing the recruitment into organized crime at the individual level. These recent developments in organized crime research have also enabled to reconnect with the broader theoretical debate, for example with the increasing attention on changes in offending patterns within individuals over time spurred by developmental and life‐course criminology (Farrington, 2003; Kleemans & De Poot, 2008).^1^ Availability of individual‐level, longitudinal data on organized crime offenders enabled to explore the factors that lead individuals to join delinquent groups and organized criminal groups within the society they belong to. Yet, in line with the prevalent focus of the field, studies mostly pointed at the role of the social environment (Kleemans & De Poot, 2008; Kleemans & Van de Bunt, 1999; Kleemans & Van Koppen, 2014; Morselli, 2009; Van Koppen et al., 2010). This study has gererally confirmed that social relations and social capital are important drivers of involvement into organized crime, and argued that inviduals join criminal groups due to the social opportunity structure, the social relations giving acces to criminally exploitable opportunities (Kleemans & De Poot, 2008). Furthermore, and possibly due to the impossibility to collect longitudinal socioeconomic and psychological data on such a specific population, studies emphasized the role of previous offending, deviance, violence and contact with the criminal justice system. Several researchers have addressed changes in offending patterns within individuals engaged in organized crime (Kleemans & De Poot, 2008; Morselli & Tremblay, 2004; Morselli, 2003; Van Koppen, de Poot, & Blokland, 2010; Van Koppen, de Poot, Kleemans, et al., 2010), while others have taken a closer look at risk factors for joining organized crime groups (Kleemans & De Poot, 2008; Kleemans & Van de Bunt, 1999; Kleemans & van Koppen, 2014; Klein & Maxson, 2006; Lyman & Potter, 2006). Few recent contributions have addressed the intergenerational transmission of delinquency and organized crime offending within families (Spapens & Moors, 2020; Van Dijk et al., Unpublished), whereas others have drawn attention on economic disadvantages (Carvalho & Soares, 2016; Lavezzi, 2008, 2014). Other studies have focused on the impact of joining organized crime groups or gangs on the life of individuals (Melde & Esbensen, 2011; Pyrooz, 2014; Pyrooz et al., 2016) or of leaving organized crime groups (Berger et al., 2017; Pyrooz et al., 2017; Sweeten et al., 2013).

### 2.4 How the risk factors may impact the recruitment into organized criminal groups

Given the scattered nature of research summarized above, there is a lack of an overarching theoretical framework on the individual‐level drivers of involvement into organized criminal groups. Criminological research has emphasized the social opportunity structure as well as the criminal skills and experiences. Yet these findings are far from providing a comprehensive theoretical framework of all possible factors that influence the recruitment into criminal organizations. For example, demographic, psychological, and economic factors may also drive the recruitment. In this regard, organized crime research remarkably differs from the study of youth gangs, where empirical and theoretical advancements have enabled the development of specific models (Decker et al., 2013; Higginson et al., 2018; Howell & Egley, 2005; Thornberry et al., 2003). The lack of theoretical framework suggests adopting a broad and flexible approach to this systematic review.

Focusing only on the main factor categories pointed out by recent research, social relations, and criminal background, may unnecessarily restrict the scope of this systematic review. Instead, this review focuses on all individual‐level factors presented in the literature, leaving to the included studies the establishment of the boundaries of the analysis. This option provides a comprehensive assessment of the factors identified by empirical research and, at the same time, enables comparison across different factors. Furthermore, it allows the necessary flexibility to encompass the multiple forms and types of organized crime groups, consistently with the broad definition presented above. Several systematic reviews in criminology followed a similar approach and a recent systematic review on the risk and protective factors for radicalization (Wolfowicz et al., 2020).

### 2.5 Why it is important to do the review

A better understanding of the factors associated with recruitment into organized criminal groups is needed to improve and consolidate the knowledge of organized crime, and to design empirically based prevention strategies. For this purpose, this systematic review aims at summarizing the existing empirical evidence about the relative strength of the risk factors related to recruitment into organized criminal groups. The theoretical debate on the definition of organized crime has often neglected empirical research. To the best of our knowledge, there are no systematic reviews with meta‐analysis on organized crime. Only recently a systematic narrative review on this topic examined 47 studies published until 2017 and pointed out the importance of social relations, criminal background, and criminal skills for the recruitment into organized crime (Calderoni et al., 2020; Comunale et al., 2020).

While only partially overlapping with organized crime literature, gang research has produced a few systematic reviews. Previous systematic reviews have focused on youth gang membership and interventions (Hodgkinson et al., 2009; Klein & Maxson, 2006; Raby & Jones, 2016). The Campbell Collaboration has published three systematic reviews on the involvement of young people in gangs (Fisher et al., 2008a, 2008b; Higginson et al., 2015), and more recently one on predictors of youth gang membership in low‐ and middle‐income countries (Higginson et al., 2018). Furthermore, two systematic reviews on the factors leading to radicalization and recruitment into terrorism have been recently published (Wolfowicz et al., 2020, 2021). While these reviews show the growing interest for the risk factors leading to involvement into groups engaged in criminal activities in a broad sense, they did not consider the factors relating to recruitment in organized crime.

A systematic approach on empirically based findings will provide a better understanding of organized crime. The findings of this review can contribute to clarifying the definitional debate around organized crime and push the field to further engage with empirical research by pointing out directions for future inquiry. Systematic analysis of the evidence regarding specific factors may show what mechanisms may drive individuals into organized criminal groups, point out similarities and differences with research on the general offending population and or other groups engaged in crimes (youth gangs, terrorist groups).

This review aims to inform not only researchers but also to support the formulation of effective evidence‐based intervention and prevention policies. By identifying the most important factors of pathways to organized crime membership, this review seeks to provide policy makers with detailed information on how to design potential intervention strategies. The importance of proper prevention policies against organized crime links to the fact that arrests only cause temporary drawbacks to the functioning of organized criminal groups. In fact, their resilience to law enforcement interventions is one of the most distinct features of organized criminal groups. This is due to organized criminal groups' ability to rapidly reorganize and to easily recruit new members. From an opportunity reduction perspective, intervention within the recruitment process could be an effective complementary strategy for combating organized crime. In this regard, the results of this systematic review may be used to inform about the most common risk factors for recruitment into organized crime, and hence to develop intervention strategies mitigating these factors. Finally, the findings may provide policy makers with more comparative insights about the dynamics of recruitment into various organized criminal groups. Shedding light on similarities in pathways into organized crime may help to formulate effective criminal justice policies applicable in various countries.

## 3 OBJECTIVES

This systematic review and meta‐analysis aim at providing a comprehensive overview of current empirical knowledge about the individual‐level risk factors related to recruitment into organized crime. This overarching aim can be subdivided into two main objectives:

- Objective 1: Summarize the empirical evidence from quantitative, mixed methods, and qualitative studies on the individual‐level risk factors associated with the recruitment into organized crime.
- Objective 2: Assess the relative strength of the risk factors from quantitative studies across different factor categories and subcategories and types of organized crime groups.

## 4 METHODS

This review is based on the previously published protocol (Calderoni et al., 2019). This section, except for specifically mentioned updates or changes, draws on the protocol.

### 4.1 Criteria for including and excluding studies

#### 4.1.1 Study design

This systematic review aims to identify and evaluate existing knowledge of individual‐level risk factors relating to recruitment into organized crime. As recruitment into organized crime cannot be the object of experimental interventions, this review examines only empirical evidence resulting from studies using an observational research design.

This review includes studies having as one of the main objectives the analysis of recruitment into organized crime. Also, studies were included if they provided sufficient information and details on the analytical strategy, including sampling technique/data collection, and type of analysis conducted, intended as the relation between a risk factor and recruitment into organized crime. This review retrieved and screened quantitative, qualitative studies, and mixed methods studies, and excluded literature reviews, theoretical and conceptual contributions, and editorial pieces. This section describes in detail the search and screening process leading to the identification and inclusion of eligible studies.

For the synthesis of quantitative research, we relied on studies with variability in recruitment into organized crime, measuring and comparing at least two groups (e.g., organized crime members vs. non‐members). The review searched for studies based on longitudinal and cross‐sectional designs, though the study eligibility assessment resulted in including only cross‐sectional studies. We included in the meta‐analysis quantitative studies reporting at least an effect size or studies providing enough information to calculate an effect size from the reported statistics, as also described in the published protocol of this review (Calderoni et al., 2019). We included qualitative and mixed methods studies (for the qualitative analysis) that reported a clear aim of the research and provided appropriate information regarding the methodology and analytical strategy.

We did not exclude studies based on their geographical scope, year of publication, or quality. We evaluated the risk of bias in included quantitative studies using a risk‐of‐bias tool adapted from Higginson et al. (2018) and PROBAST tool for prediction studies (see Quality assessment of the included studies). We assessed the quality of qualitative and mixed methods studies using the CASP Qualitative Checklist (Critical Appraisal Skills Programme, 2018).

#### 4.1.2 Types of organized crime groups

The literature has long debated on the definition of organized crime and the characteristics of organized criminal groups. With the aim of favoring the inclusion of the largest number of eligible studies, we adopted the broad definition provided by Article 2 of the United Nations Convention against Transnational Organized Crime (United Nations, 2000, p. 5):

“Organized criminal group” shall mean a structured group of three or more persons, existing for a period of time and acting in concert with the aim of committing one or more serious crimes or offences established in accordance with this Convention, to obtain, directly or indirectly, a financial or other material benefit.

Under this definition, a variety of groups are described as organized criminal groups, including traditional mafias, drug trafficking organizations, and adult gangs. We excluded groups described as youth (street) gangs, prison gangs, and terrorist groups. The literature generally discriminates between youth street gangs and organized criminal groups (Decker & Pyrooz, 2014), with the latter having an important share of adult offenders adults involved in potentially more complex criminal activities aiming at profit. Furthermore, previous systematic reviews have already assessed the factors leading to youth gang membership (Higginson et al., 2018; Klein & Maxson, 2006; Raby & Jones, 2016). As for prison gangs, while some are extension of criminal organizations active outside the prison, others establish themselves and thrive in the isolation of the prison setting. Moreover, while there is a relevant literature on prison gangs, this field is mostly separate from the literature on organized crime, which emphasizes the social embeddedness into the legitimate world. For these reasons, we excluded prison gangs, as the recruitment of individuals in such groups occurs in confined settings and therefore is influenced by different contextual factors (Blevins et al., 2010; Wood et al., 2014). Lastly, we excluded terrorist groups due to their ideological/political motivation. In addition, two systematic reviews on the putative risk and protective factors relating to cognitive and behavioral radicalization were recently published (Wolfowicz et al., 2020, 2021).

#### 4.1.3 Types of factors

This systematic review includes only studies measuring recruitment into organized criminal groups at the individual level. We did not limit the search of studies to specific factors, adopting a field‐wide approach to ensure a broad coverage of the available evidence. As a result, we identified several types of factors that can be nonetheless grouped into different categories: sociodemographic, economic, psychological, and criminal history factors.

For a variable to be considered as a risk factor, it must occur before the outcome (Murray et al., 2009). The risk factor therefore must precede recruitment into organized crime, and this would ideally require longitudinal designs for its measurement. However, some factors may be considered as preceding the recruitment even if included in cross‐sectional studies, as they do not vary over the life course (e.g., sex, ethnicity). For this reason, we considered as risk factors for organized crime membership not only predictors measured before organized crime membership but also time‐invariant factors estimated from cross‐sectional studies. We also considered self‐reported retrospective data assessing risk factors preceding the outcome, though they present some biases as they are based on individual's recall of past events (Murray et al., 2009). This choice was driven by the aim of including as many studies as possible and enhance the knowledge of individual‐level factors leading to recruitment into organized crime.

In line with previous systematic reviews (Higginson et al., 2018; Klein & Maxson, 2006), we classified as *predictors* the factors measuring conditions preceding the recruitment into organized criminal groups and as *correlates* the factors measuring conditions occurring simultaneously or after the recruitment. Effects and results of the meta‐analysis of predictors and correlates are reported separately (see Synthesis of results).

#### 4.1.4 Types of outcome measures

The review included self‐ and peer‐reported measures, and practitioner‐ and police‐reported measures of individual organized crime membership. The outcome of interest in this systematic review is the recruitment into organized crime, measured with a dichotomous variable. We considered recruitment as a more general concept referring to the several processes leading individuals to the stable involvement into organized crime groups, without differentiating between different forms of recruitment. For this reason, we included studies focusing on recruitment, affiliation, and other forms of stable involvement. Lastly, we conducted moderator analyses by type of organized criminal group to assess the variation in effect sizes attributable to heterogeneity.

### 4.2 Search methods for identification of studies

#### 4.2.1 Search terms

**Figure 1:**
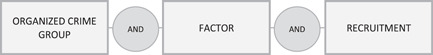
Query structure

This review relied on a three‐fold query structure that ensured systematic, comprehensive, and efficient screening results. The queries incorporate all aspects that are relevant to the factors relating to the recruitment into different types of organized criminal groups. The search terms from each of the three main categories (organized crime groups, factors, and recruitment) combined formed the queries. The Boolean Operator “OR” connected keywords pertaining to the same category, while the Boolean Operator “AND” connected keywords from different categories (Figure 1). This query structure ensured to retrieve all the studies containing at least one term from each word category (see Table 11 in Supporting Information Appendix A: Search categories and related search terms).

#### 4.2.2 Search locations and languages

The search for eligible studies relied on 12 databases relating to different research disciplines—including social, psychological, and economic research—reflecting the transdisciplinary approach of this systematic review.^2^ The search strategy included published or unpublished studies in English, French, German, Italian, and Spanish.^3^ We applied no limitations as to their year of publication or geographic origin. Table 1 reports the list of databases by language of the search and search technique. When available, the preferred technique was to search title, abstract and keywords.

**Table 1:** List of databases and search techniques

**Language**	**Database**	**Sub‐database**	**Search technique**
English	EBSCO	Criminal Justice Abstracts	Abstract
	Open Grey		Full‐text
	ProQuest	Social Sciences Premium	Abstract
		NJCRS	
		APA PsycInfo	
		ABI/INFORM Collection	
		International Bibliography of the Social Sciences	
		Public Health Database	
		Military Database	
		EconLit	
		APA PsycArticles	
	PubMed		Title and Abstract
	Scopus		Title, Abstract & Keyword
	Web of Science	Science Citation Index Expanded	Title
		Social Sciences Citation Index	
		Arts & Humanities Citation Index	
		Conference Proceedings Citation Index—Science	
		Conference Proceedings Citation Index—Social Sciences and Humanities	
		Book Citation Index—Science	
		Book Citation Index—Social Sciences & Humanities	
		Emerging Sources Citation Index	
French	Google Scholar		Full‐text
	Sudoc.Abes		Title
German	Sowiport		Title
Italian	Riviste Web		Full‐text
Spanish	Liliacs		Title, Abstract & Subject
	ProQuest	Latin America & Iberia Database	Full‐text

The initial search was conducted between January and March 2017. An updated search was performed between September and October 2019.

We attended two meetings with a librarian to validate the search terms and queries and ensure the inclusion of all databases relevant to this systematic review (see Table 11 in Supporting Information Appendix A: Search categories and related search terms).

#### 4.2.3 Multistage approach to searching

We identified potentially eligible studies not only through scientific database searching but also through contact with experts in the field of organized crime. The initial list of experts to be contacted was further expanded including the authors of the studies deemed eligible after the full text screening.^4^ Lastly, we identified relevant literature from the bibliographies of the potentially eligible studies retrieved for full‐text screening and we included such studies in the full‐text screening.

### 4.3 Data collection and analysis

#### 4.3.1 Selection of studies

The review process incorporated all the studies retrieved through database search, references search, and experts' contribution. Metadata for each study were imported into the Covidence online platform that provides an environment to manage and conduct systematic reviews.^5^

After the removal of duplicate entries, the research team underwent training sessions for the screening of potentially eligible studies. The trainings provided researchers with background information on the aim of the systematic review as well as with briefings on how to implement the search strategy and screening of studies. A preliminary screening phase was performed, with each reviewer independently conducting the title and abstract screening of a set of 100 studies. The results were then compared among all researchers and disagreements were discussed to reach common criteria for screening and including eligible studies. To ensure reliability throughout the screening process, two reviewers screened each document. A third researcher settled divergent screening decisions, in consultation with the full review team where necessary.^6^

First, the research team performed title and abstract screening to retain only studies investigating recruitment into organized criminal groups as one of the main aims of the study. When the information reported in the title and abstract was not sufficient to include or exclude the document, we retained the study for full‐text screening.

Second, the research team performed full‐text screening of all potentially eligible studies retained.^7^ To be included, each document had to meet all the eligibility criteria listed in the “Eligibility screening form” (see Table 12 in Supporting Information Appendix B: Eligibility screening form). If none of the eligibility criteria could be definitively answered, the study was filtered out. While in the previous phase we favored inclusivity, in this phase every criterion needed to be conclusively met, on penalty of study exclusion.

#### 4.3.2 Data extraction and management

The quantitative, qualitative, and mixed methods studies that met all full‐text screening criteria were independently coded by two reviewers based on a detailed coding guide (see Supporting Information Appendix C: Document coding protocol). We initially planned to code mixed methods studies twice, one entry for the quantitative section and one entry for the qualitative one. However, the full‐text screening resulted in limiting their inclusion to the set of eligible qualitative studies, as the quantitative parts of the mixed‐methods studies did not meet the last item of the “Eligibility screening form,” that is, variability in the outcome measure (see Table 12 in Supporting Information Appendix B: Eligibility screening form). As for the previous screening steps, the results of the reviewers were compared, and any coding conflict was resolved through exchanges with the review manager.

#### 4.3.3 Quality assessment of the included studies

We assessed the risk of study bias for quantitative studies through a section of the coding protocol (questions 58–85 in Table 14 in Supporting Information Appendix C: Document coding protocol). The quality of each study was assessed by two authors. The review manager evaluated the two assessments and promoted a consensus decision for discrepancies. The items in the coding protocol allowed the investigation of a variety of potential issues related to sample selection, risk factors and outcome definition and application and statistical modeling, including diagnostic measures on the statistical models. The protocol allowed to analytically reach an overall risk‐of‐bias rating for each included quantitative study. The quality assessment is largely an adaptation of Higginson and colleagues' systematic review (Higginson et al., 2018) and of PROBAST risk‐of‐bias tool for prediction models (PROBAST, 2018, p. 8). Overall, the risk of bias judgment is as follows:

- Low risk of bias: If all domains were rated low risk of bias.
- High risk of bias: If at least one domain is judged to be at high risk of bias.
- Unclear risk of bias: If an unclear risk of bias was noted in at least one domain and it was low risk for all other domains.

In line with previous meta‐analysis protocols, we did not exclude low‐quality studies (see Higginson et al., 2018) and we opted for the “traffic light” model adopted by de Vibe et al. (2012) to present the results.

For the included qualitative studies and the qualitative parts of mixed‐method studies the quality assessment relied on an adaptation of the CASP Qualitative Checklist (Critical Appraisal Skills Programme, 2018). Of the original 10‐item checklist we retained the following five items (items 98–102 in Table 15 in Supporting Information Appendix C: Document coding protocol:

- *Clear aim on recruitment*: the qualitative study main aim must be on the recruitment into organized crime, or the topic must be addressed in a relevant part of the study (chapter, section, subsection).
- *Research design appropriate*: the study must clearly indicate the research design adopted to investigate the recruitment into organized criminal groups or the research design must be the same for all the objectives of the study, including the recruitment.
- *Data collection appropriate*: the study must clearly state the sources of information to investigate the recruitment into organized crime, and/or the sources must be the same for the rest of the study. The study must offer indications on how the information was collected, verified, and analyzed.
- *Data analysis rigorous*: the study must provide an in‐depth description of the analysis, of the construction of categories and themes, present sufficient data.
- *Clear statement of findings*: the study must clearly present the findings, discuss them in relation to limitations and other contributions.

Also for the quality assessment of qualitative studies we did not exclude low‐quality studies. We presented the results of the assessment adapting the “traffic light” model to the five items.

#### 4.3.4 Effect size metric and calculations

To perform the meta‐analyses, we transformed different statistical measures reported in eligible quantitative studies into comparable effect size measures. When effect sizes were not directly reported in the studies, we calculated them based on the reported and extrapolated statistics. When studies did not report enough information to calculate effect sizes, we contacted the authors to obtain the necessary data (see below, Missing data). We extracted effect sizes and relevant statistics following a detailed coding guide throughout the process (see items 35–57 in Supporting Information Appendix C: Document coding protocol).

We coded all effect sizes extracted from the included quantitative studies based on several dimensions relevant for synthesis and interpretation, including: the document of origin, the nature of the two (or more) groups the effect was assessed on (e.g. organized crime members for the organized crime group and offenders in general for the non‐organized‐crime group), and the risk factor each effect size referred to (items 1–4, 18–19, and 35 of Supporting Information Appendix C: Document coding protocol, respectively). We carried out the statistical synthesis for all the comparable effect sizes between similar pairs of groups. We classified effect sized based on their focus domain (sociodemographic, economic, psychological, criminal history) (see item 36 in Supporting Information Appendix C: Document coding protocol). However, we opted to present the results based on a list of categories and subcategories that were inductively identified from the data (see items 36a and 36b in Supporting Information Appendix C: Document coding protocol).

We calculated effect sizes using two categories of statistics: group means, for continuous variables, and risk‐based association measures between two binary variables. The quantitative studies included in this review reported their results using mainly group mean differences and standard deviations for continuous variables, and contingency tables or odds ratios for binary variables. Such type of data was transformed into effect sizes in the form of *log odds ratios* to perform the meta‐analysis.

The logic of using log odds ratios as a common statistic is twofold. First, both odds ratios and log odds ratios are symmetrical across the two variables they reference. Second, log odds ratios have the property of symmetry around their null value. While odds ratios are defined between 0 and positive infinity with a null value of 1 and asymmetrical standard errors, log odds ratios “normalize” the null value to 0 and are defined between negative infinity and positive infinity, with symmetrical standard errors regardless of sign (see Borenstein et al., 2009, p. 35).

Log odds ratios, however, are difficult to interpret. To assist the reader in interpreting our results, in the Discussion section we converted the average log odds ratios into odds ratios.

The conversion to log odds ratios entails, respectively:

1. For continuous variables for which group means and variance are reported, calculating first Cohen's *d* and *d*'s standard error (Borenstein et al., 2009, p. 21). These measures will then be used to calculate the log odds ratio and the standard error (Borenstein et al., 2009, p. 47).
2. For binary variables for which contingency tables or odds ratios are reported, calculating log odds ratio and standard error (Borenstein et al., 2009, p. 33).

#### 4.3.5 Determining independent findings

Some included studies relied on the same data to investigate different issues. In some cases, however, they reported the same factors. To avoid issues of lack of independence among the estimated effect sizes, we paired six studies employing the same data before the inclusion of the effect sizes in the meta‐analysis. The resulting pairs are: Francis et al. (2013)/Kirby et al. (2016), Decker et al. (2014)/Pyrooz et al. (2015), and Coid et al. (2013)/Wood et al. (2017). The first pair did not pose any issue, since the two studies always reported the same values for the same factors. We thus ensured that the extracted measures were included only once. The other two pairs reported slightly different values, possibly due to few observations being dropped from the analyses for various, unspecified reasons. Nonetheless, the estimated effect sizes were always similar. We thus opted to include the effect sizes from the study reporting the largest samples within each pair.

Second, one study (Pedersen, 2018) reported estimates for two different types of organized criminal groups: outlaw motorcycle gang members (OMCG) and adult gang members. We therefore split the effect sizes extracted from Pedersen (2018) as if they were extracted from two different studies. We reported these effect sizes separately, by labeling them as “Pedersen, 2018—OMCG” and “Pedersen, 2018—Gang.”

Third, several included studies reported different effect sizes falling within the same factor category or subcategory. For example, several studies reported effect sizes comparing organized criminal groups with more than one non‐organized‐crime group type (e.g., offenders in general, violent men). In addition, multiple effect sizes measured the same construct (e.g., several reported measures of violence). This required to combine such measures into one synthetic effect size before inclusion in the analysis (see below, Data synthesis).

#### 4.3.6 Assessment of publication bias

We planned to test publication bias through funnel plots, a specialized form of scatter plots used in meta‐analysis to visually identify publication and other bias (Sterne et al., 2006) and adjust for publication bias with trim and fill analysis following the methodology suggested by Rothstein et al. (2005). However, due to the low number of independent effect sizes included in the meta‐analysis, it was not possible to conduct these tests. Moreover, all included studies were published studies. For these reasons, we acknowledge that the results may be affected by publication bias.

#### 4.3.7 Missing data

One eligible study (Danner & Silverman, 1986) included insufficient data to determine any effect size except one. Another study (Sharpe, 2002) provided only partial information, allowing the computation of only some effect sizes. We could not retrieve the email contacts of the authors of these two studies.

Other eligible studies provided incomplete data for few measures or variables (e.g., reporting only average values without standard deviations). We contacted the authors asking for additional information. We received feedback from several contacted authors, who provided sufficient information to integrate the data from the included studies (Adams & Pizarro, 2014; Carvalho & Soares, 2016; Francis et al., 2013; Kirby et al., 2016; Klement, 2016). For one study, the authors were unable to provide the requested information (Van Koppen et al., 2010). An integration request is still pending for one study (Blokland et al., 2019).

#### 4.3.8 Data synthesis

Whenever included studies reported multiple effect sizes falling within the same factor category or subcategory, we synthesized effect sizes adopting the following procedure:

1. We grouped effect sizes by study, factor category (and subcategory where applicable), and factor type (predictor or correlate).
2. Some studies also reported the same measures for multiple non‐organized‐crime groups (i.e., comparison group, see below Characteristics of included studies for further details). In such cases, in line with the literature on subgroup analysis (see Borenstein et al., 2009, pp. 149–186), we first synthesized effect sizes of the same study by comparison group, then the synthetic measures were subsequently synthesized to obtain a synthetic effect size for each study. Within‐study effect sizes were computed using the Stata *robumeta* command which allows to estimate robust variance in meta‐regression with dependent effect sizes estimates (the analyses used random‐effects models).^8^
3. Whenever possible, we included the synthetic effect sizes in random‐effects meta‐analyses using the Stata *meta* command (StataCorp, 2019). Alternatively, we just reported the synthetic effect sizes (e.g., when no other studies reported on the same measures).

We conducted a random‐effects meta‐analysis using inverse variance weighting when at least two included studies provided predictors or correlates falling within the same factor category and measuring conceptually similar factors. In this way, we calculated the overall weighted mean effect estimate of each separate factor on organized crime recruitment. We carried out meta‐analysis using log odds ratios and we presented the results in a forest plot with 95% confidence intervals. We presented results of meta‐analyses of predictors separately to results of meta‐analyses of correlates. For each type of factors, we performed a meta‐analysis on different factors, including sociodemographic, economic, psychological, and criminal history factors. We initially planned to conduct meta‐analyses including only effect sizes that measured not only the same factor, but also the same pair of organized crime versus non‐organized‐crime group (e.g., organized crime members vs. offenders in general) (see published protocol, Calderoni et al., 2019). However, this sublevel of analysis would have limited the number of meta‐analyses due to the low number of effect sizes retrieved from included quantitative studies. For this reason, differing from the protocol, we conducted meta‐analyses only by type of effect size (predictor, correlate) and type of factor category or subcategory. Nonetheless, we conducted moderator analyses by type of organized criminal group to further investigate statistically significant heterogeneity displayed by the results of meta‐analyses (see below Sensitivity and subgroup analysis and Supporting Information Appendix E: Moderator analyses by type of organized criminal group).

#### 4.3.9 Assessment and investigation of heterogeneity

The study of heterogeneity can provide indications on how to interpret the overall effect size of each meta‐analysis (Borenstein et al., 2009). We assessed heterogeneity between studies with the *I* ^2^ and *τ* ^2^ (Borenstein et al., 2009). Given the diversity of the groups classified as organized crime across time and countries and the controversies surrounding the definition of organized crime (as discussed above in Background), we performed subgroup meta‐analyses moderating studies by type of organized criminal group for all meta‐analyses showing statistically significant heterogeneity. We included forest plots displaying an inverse‐variance weighted random‐effect meta‐analysis of the effect of factor category on involvement into organized criminal groups (see Supporting Information Appendix E: Moderator analyses by type of organized criminal group). Results of moderator analyses should be interpreted with caution, as the number of effect sizes for each moderator category is limited and the inclusion of additional studies may alter the results.

#### 4.3.10 Sensitivity and subgroup analysis

We initially planned to conduct subgroup analyses to further investigate the effect of risk of bias, geographic scope as well as the effect of study heterogeneity. However, due to the low number of included studies in each meta‐analysis, we did not conduct sensitivity analyses of risk of bias and of geographic scope. We assessed the heterogeneity through subgroup meta‐analyses moderating studies by type of organized criminal group, using Stata 16 *meta* command (StataCorp, 2019). Results of the moderator analyses, analogous to the analysis of variance (ANOVA), are presented in a separate subsection at the end of the Results section, and integrally reported in Supporting Information Appendix E: Moderator analyses by type of organized criminal group.

#### 4.3.11 Treatment of qualitative research

Systematic reviews have generally excluded qualitative studies because of the impossibility of using their findings to draw conclusions. Nonetheless, Campbell policies and guidelines have recently encouraged the inclusion of qualitative and descriptive research, which can provide a more comprehensive overview of the object of study. In addition, both anonymous reviewers of the protocol stressed the importance of including relevant qualitative works to achieve the objectives of this review. For these reasons, this systematic review includes quantitative studies as well as qualitative studies.

We systematically retrieved and screened qualitative studies for their inclusion, coding them using part of the coding document also used for the quantitative literature. We assessed the quality of the included studies through a 5‐item list adapted from the CASP Qualitative Checklist (Critical Appraisal Skills Programme, 2018). The included studies were used to inform, contextualize, and expand the knowledge resulting from the evidence and findings of the quantitative studies.

## 5 RESULTS

### 5.1 Description of studies

#### 5.1.1 Results of the search

**Figure 2:**
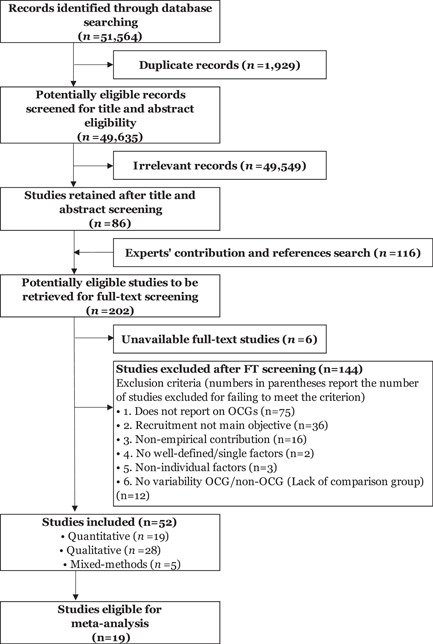
PRISMA flow diagram of search and screening process

The search led to the collection of 51,564 records that were subsequently screened for assessing their eligibility for this systematic review (Figure 2). A team of trained researchers applied common criteria in screening the title and abstract of each study. We considered as relevant for the scope of the review studies focusing on and/or reporting about individual‐level factors for recruitment into organized criminal groups and making an original research contribution. We therefore excluded news articles, theoretical contributions, or reviews of any type.^9^

From the initial number of records, 1929 documents consisted of duplicates and therefore were excluded. A total of 49,547 records were considered irrelevant and largely off‐topic as they did not meet the inclusion criteria for title and abstract screening. We thus retained 86 remaining studies. Experts' contribution and references search led to the identification of 116 additional studies, reaching a total of 202 studies potentially eligible for full‐text screening. Of these, we failed to retrieve six studies as the full text was unavailable. The full‐text screening, based on six items (with the sixth item applied only to quantitative studies), allowed to exclude 144 studies that did not meet one or more of criteria, resulting in 52 studies deemed eligible for inclusion.

#### 5.1.2 Included studies

The search and screening process led to the inclusion of 52 studies adopting a quantitative (19), qualitative (28), or mixed methods approach (5) (Figure 2). The 19 quantitative studies were included in the meta‐analyses while the qualitative information provided by the 28 qualitative and 6 mixed methods studies was coded as relevant factor categories on recruitment into organized crime. We categorized the included studies through a detailed document coding protocol classifying their characteristics based on several items (Supporting Information Appendix C: Document coding protocol).

#### 5.1.3 Excluded studies

Full‐text screening allowed to exclude 144 studies that did not meet any of the six inclusion criteria. The studies were deemed ineligible because they did not report on organized criminal groups as defined for this review (i.e., out of scope studies, *n* = 75), recruitment into organized criminal groups not main objective of the study (*n* = 36), nonempirical contribution (*n* = 16), no well‐defined/single factors (*n* = 2), nonindividual factors (*n* = 3), lack of comparison group (*n* = 12) (Figure 2). A table with the full reference of the excluded studies as well as the reasons for exclusion is reported below in References to excluded studies.

### 5.2 Characteristics of included studies

#### 5.2.1 Quantitative studies

The 19 included quantitative studies are summarized below and in Table 2. The full references are provided in References to included studies.

**Table 2:** Characteristics of included quantitative studies

Study	Study objectives	Country	Type of OCG	Type of comparison group	Methods of data collection and sample	Data analysis	Risk factors (by category)
Adams and Pizarro (2014)	Examine the arrest histories of homicide gang and non‐gang offenders to assess patterns of offense specialization, escalation, or de‐escalation.	US	Gang	Serious offenders	Police intelligence data	The authors use multinomial models to calculate the predicted probability of a particular offense type being committed at each arrest, with specifications for gang members and non‐gang members. Differences in age, number of arrests and ethnicity for gang and non‐gang members are reported, but only data about ethnicity allow effect size calculation.	Age; Ethnicity; Offence and/or contact with CJ system
					Data is retrieved from homicide investigation files compiled by the Newark Police Department's Homicide Unit from 1999 to 2005. The sample includes 140 homicide offenders that had at least five arrests before the homicide (81 non‐gang members and 59 gang members).		
Blokland et al. (2019)	Examine various dimensions of the criminal careers of outlaw bikers, including participation, onset, frequency, and crime mix.	The Netherlands	Biker gang	Offenders in general; Population sample	Police intelligence data	The authors report the means and standard deviations between OMCG members and non‐OMCG members across personal and criminal careers characteristics. T‐tests and Chi2/Fisher Exact tests significance are also reported. They also perform logistic regressions to predict OMCG membership.	Foreign born; Low self‐control; Offence and/or contact with CJ system; Sanctions; Violence
					Data is retrieved from the outlaw motorcycle gangs intelligence unit of the Central Criminal Investigations Division of the Netherlands National Police (OMCG members), and from the Dutch National Vehicle and Driving Licence Registration Authority database “light vehicles” (non‐OMCG members). The criminal careers of OMCG members and the comparison group of motorcycle owners are constructed using extracts from the Judicial Information System. The sample includes 601 OMCG members, and 300 non‐OMCG members.		
Bottini et al. (2017)	Examine which psychological and neuropsychological variables better discriminates differences between the groups of OC prisoners, non‐OC prisoners, and non‐prisoners controls.	Italy	Mafia	Offenders in general; Population sample	Survey (interviews) + professional testing	The authors report mean scores and standard deviations for several variables across the three groups. They test the statistical significance of the differences among groups through different tests. They also perform discriminant analyses between OC and non‐OC prisoners, although results are reported summarily	Age; Anxiety; Cognitive functioning; Depression; Education; Low self‐control; Negative life events; Psychopathy and antisocial personality disorder; Sanctions
					Data is retrieved thanks to the performance of neuropsychological interviews, psychological assessments, and neuropsychological tests. The sample includes 150 male individuals (50 OC prisoners, 50 non‐OC prisoners, and a control group of 50 non‐prisoners). OC and non‐OC prisoners comprised 25 violent and 25 nonviolent individuals per group. The non‐prisoner control group was matched in age, years of education with the prisoner groups.		
Carvalho and Soares (2016)	Characterize drug‐trafficking jobs and study the selection into gangs, analyzing what distinguishes gang‐members from other youth living in favelas.	Brazil	Drug trafficking organization	Population sample	Survey (interviews)	The authors propose equations to estimate gang membership, and earnings for the illegal sector. In addition, key characteristics of interviewees related to ethnicity, religion, marital status, age, education, and labor market status are compared to data from the Brazilian Census of males aged 10–25 living in Rio's favelas; only some of these variables allow effect size calculation.	Age; Being in a relationship; Economic condition; Education; Ethnicity; Living conditions/household (adulthood); Religious beliefs; Troubled family environment
					Data is retrieved from interviews collected by the Brazilian NGO Observatório de Favelas (OF) on individuals working for the drug‐trafficking business in favelas of Rio de Janeiro. The sample includes 230 individuals (98% males) aged 11–24 who were members of different drug‐trafficking gangs.		
Coid et al. (2013)	Investigate associations between gang membership, violent behavior, psychiatric morbidity, and use of mental health services.	UK	Gang	Population sample; Violent men	Survey (questionnaires)	The authors use logistic regressions to compare the demographic characteristics of non‐violent men, violent men, and gang members. Linear trends are established by entering group membership as an ordinal variable. Finally, associations between gang membership, violence, and psychopathology or service use are investigated.	Age; Anxiety; Being in a relationship; Depression; Economic condition; Ethnicity; Foreign born; Low self‐control; Negative life events; Psychopathy and antisocial personality disorder; Violence
					Data is retrieved from a cross‐sectional survey using random location sampling in Great Britain. The survey gathers information about gang membership, violence, use of mental health services, and psychiatric diagnoses. The sample includes 4,664 men aged 18–34, and men from areas with high levels of violence and gang activities are oversampled.		
Danner and Silverman (1986)	Examine the extent to which there is a distinct configuration of demographic, offense history, and attitude characteristics among bikers and non‐biker inmates.	US	Biker gang	Offenders in general	Survey (questionnaires)	The authors perform a discriminant analysis to discover differences between the biker and non‐biker groups regarding their demographic characteristics, offense history, and attitude/value orientations. However, only data about ethnicity allow effect size calculation.	Ethnicity
					Data is retrieved from a survey about demographic characteristics and criminal history administrated to bikers and non‐bikers incarcerated in adult correctional institutions in the United States. The sample includes 168 individuals (63 bikers and 105 non‐bikers).		
Decker et al. (2014)	Validate self‐nomination in gang research.				Survey (interviews)		
	Investigate differences in gang embeddedness across current gang members, former gang members, and those individuals who have never joined a gang.	US	Gang	Population sample	Data is retrieved from interviews conducted with individuals in 5 US cities in settings chosen to include many individuals with involvement in gangs and criminal behavior. The sample includes 621 respondents (188 current gang members, 264 former gang members, and 169 non‐members).	The authors assess the unadjusted differences between non‐, former, and current gang members across the study variables, focusing on the magnitude and statistical significance of differences. Then, standardized differences in a mixed graded response model of gang embeddedness are evaluated across the three statuses of gang membership to assess the validity of self‐nominated gang membership.	Age; Criminal versatility; Education; Ethnicity; Low self‐control; Motivation; Sex; Social environment; Troubled family environment; Violence
Francis et al. (2013)	Examine the criminal histories of offenders who become involved in organized crime by analyzing administrative data on criminal sanctions.	UK	Other organized crime group	Offenders in general; Serious offenders	Judicial/official data	The authors compare the demographics of organized crime offenders and the nature of the inclusion offences to the characteristics of offenders in the comparison groups. The onset of organized offenders' criminal career, the volume of offences before being convicted of organized crime, the specialization within their criminal career, the offending profile, and the escalation of offence seriousness are also investigated.	Age; Criminal versatility; Ethnicity; Foreign born; Offence and/or contact with CJ system; Sex
					Data is retrieved from the Police National Computer (PNC), which includes information on sanctioned offenders in the UK in 2007–2010. The sample includes 4109 offenders convicted for offences linked to organized crime, and two comparison groups (4090 general crime offenders, and 4109 serious crime offenders).		
Kirby et al. (2016)	Explore the feasibility of identifying a greater number of organized crime offenders, currently captured but invisible, within existing national general crime databases.	UK	Other organized crime group	Offenders in general; Serious offenders	Judicial/official data	The authors identify demographics and offending behavior of organized crime offenders, the spatial distribution of organized crime offenders, and they establish whether it is possible to distinguish organized crime offenders from control groups. Gender, age, nationality, ethnicity, and inclusion offence are tested using Chi2 tests. To examine differences between means, the distributional assumption of normality is tested using quantile‐quantile plots and using the Kruskal‐Wallis test if non‐normality is found, and a one‐way ANOVA otherwise.	Age; Criminal versatility; Ethnicity; Foreign born; Offence and/or contact with CJ system; Sex
					Data is retrieved from the Police National Computer (PNC), containing information on sanctioned offenders in the UK in 2007–2010. The sample includes 4109 offenders convicted for an offence linked to organized crime, and two comparison groups (4090 general crime offenders, and 4109 serious crime offenders).		
Kissner and Pyrooz (2009)	Assess the relative independent effects on gang membership of differential association and self‐control measures in terms of both strength and significance.	US	Gang	Population sample	Survey (interviews)	The authors provide descriptive statistics to assess if current gang members are distinguishable from the two control groups. Pearson *χ* ^2^ tests are run for nominal level variables to examine significance between the three groups; independent sample *t* tests are run to examine significance for all other variables. Logistic regressions are executed to evaluate the effect of self‐control on former and current gang membership.	Age; Economic condition; Ethnicity; Low self‐control; Sex; Social environment; Troubled family environment
					Data is retrieved from face‐to‐face interviews to a random cluster sample of inmates located in a large California city. The sample includes 200 inmates that self‐nominated themselves as non‐gang members (*n* = 136), former gang members (*n* = 27), and current gang members (*n* = 33).		
Klement (2016)	Investigate the effect of being an outlaw biker on criminal involvement in Denmark.	Denmark	Biker gang	Offenders in general	Police intelligence data	The authors estimate 46 unstandardized difference‐in‐difference regressions to assess the effect of an affiliation with an outlaw motorcycle club on criminal involvement.	Economic condition; Education; Offence and/or contact with CJ system; Offence type; Sanctions; Violence
					Data is retrieved from the Danish National Police that provides information on a list of 1146 individuals suspected of involvement in organized crime and from Statistics Denmark that provides extensive information on individuals in a depersonalized anonymous format. The final sample includes 297 outlaw bikers that are matched on various background characteristics with 181,931 control individuals.		
Levitt and Venkatesh (2001)	Reconstruct the economic and social histories of a group of young males who spent their adolescence in the early 1990s in a neighborhood economically marginalized and heavily influenced by gangs and drugs.	US	Gang	Population sample	Survey (interviews)	The authors document the labor‐market experiences of individuals included in the sample. Overall means and standard deviations, and the breakdown by gang status, for background characteristics are presented. OLS regressions are employed to assess the relationship between background characteristics of individuals, gang involvement and educational attainment.	Age; Economic condition; Education; Living conditions/household (adulthood); Troubled family environment
					Data is retrieved from a structured survey administered orally to a group of young men who came of age in early ‘90 s and that, at the peak of the crack epidemic, used to live in Chicago in a neighborhood heavily influenced by gangs and drugs. The sample includes 29 gang members and 61 non‐gang members.		
Ostrosky et al. (2012)	Describe a sample of incarcerated serious offenders who participated in Mexican drug gangs, in relation to Psychopathy Checklist, Revised (PCL‐R) scores and an array of cognitive neuropsychology assessments of prefrontal functioning.	Mexico	Drug trafficking organization	Population sample	Survey (interviews) + professional testing	The authors provide a descriptive characterization of the sample by mean and range. To assess differences between groups in all measures, Bonferroni post‐hoc correction tests are performed.	Age; Cognitive functioning; Education; Psychopathy and antisocial personality disorder
					Data related to the psychological assessment is retrieved from semi‐structured interviews, review of files provided by the prison authorities, and the Psychopathy Checklist, Revised (PCL‐R). Data related to the neuropsychological assessment is retrieved from the Executive Functions Battery (BANFE). The sample includes 82 inmates pertaining to different hierarchical levels in criminal organizations related to drug production, trafficking, and marketing and with absence of any psychiatric, medical, or neurological disorder, and a control group of 76 healthy male volunteers with no history of convictions, arrests, or use of drugs.		
Pedersen (2018)	Examine crime specialization and crime seriousness before gang initiation among adult gang members, outlaw bikers and matched comparison groups of offenders.	Denmark	Biker gang; Gang	Offenders in general	Police intelligence data	The author computes the diversity index and the forward specialization coefficient to examine the quantity of each offence type among individuals and the degree of specialization over time. Differences in the offence committed and criminal versatility among groups are also reported, allowing effect size calculation.	Criminal versatility; Offence type; Violence
					Data is retrieved from Statistics Denmark and the Police Intelligence Database and it provides information on criminal records of gang members, outlaw bikers and offenders who stay out of such gangs. The sample includes 564 gang members with a matching group of 1608 non‐gang offenders, and 800 outlaw bikers with a matching group of 2390 non‐biker offenders.		
Pyrooz et al. (2015)	Explore the prevalence of Internet usage among gang and non‐gang youth and young adults. Explore whether gang members have a higher propensity to engage in criminal and deviant activities online than their non‐gang peers.	US	Gang	Population sample	Survey (interviews)	The authors present univariate and bivariate statistics establishing the nature and patterns of online activities among groups. Then, multi‐level logistic IRT modeling is used to relate gang membership status to crime and deviance online.	Age; Criminal versatility; Education; Ethnicity; Foreign born; Internet use and technological capacity; Low self‐control; Motivation; Offence type; Sex; Social environment; Troubled family environment; Violence; Motivation
					Data is retrieved from face‐to‐face interviews about the use of the Internet and involvement in gangs conducted with youth and young adults in five US cities. The sample includes 585 respondents (174 self‐nominated current gang members, 244 self‐nominated former gang members, and 167 self‐nominated non‐gang members).		
Schimmenti et al. (2014)	Examine the levels of psychopathic traits among Mafia members who have been convicted of a criminal offence.	Italy	Mafia	Offenders in general	Survey (interviews)	The authors compute descriptive statistics for sociodemographic and psychopathic trait variables. Student's t‐test and Pearson's Chi2 tests are performed to assess differences between Mafia members and other inmates. A stepwise logistic regression based on Wald statistic is undertaken to examine the associations between psychopathic traits, sociodemographic variables, and the classification of participants into groups.	Age; Being in a relationship; Education; Low self‐control; Psychopathy and antisocial personality disorder; Sanctions
					Data is retrieved from a semi‐structured interview administrating the Psychopathy Checklist‐Revised (PCL‐R) and from prison files review. The sample includes 30 men convicted of Mafia‐related and 39 of non‐Mafia‐related crimes convicted in the Pagliarelli prison in Palermo.		
Sharpe (2002)	Apply the epidemiology model or risk factor approach to determine the level of association of risk factors to gang membership.	US	Gang	Offenders in general	Survey (questionnaires)	The author computes logistic regressions and *t*‐test analysis to determine the nature of the relationship between the identified risk factors and gang membership. Odds ratio analysis is also performed to determine the relative risk an individual faced if exposed to certain factors.	Economic condition; Ethnicity; Negative life events; Offence and/or contact with CJ system; Religious beliefs; Sex; Troubled family environment
					Data is retrieved from a survey focused on all aspects of gang life administrated to a convenience sample of inmates incarcerated in North Carolina. The sample includes 396 self‐identified gang members and a control group of 390 self‐identified non‐gang members.		
Van Koppen et al. (2010)	Make an extensive comparison between offenders who engage in organized crime at a particular moment in their lives and general offenders, based on various dimensions of their criminal careers.	The Netherlands	Other organized crime group	Offenders in general	Judicial/official data	The authors compare career data on age, prevalence and frequency of offending, and seriousness of offending between organized crime offenders and general offenders.	Offence and/or contact with CJ system; Sanctions; Sex
					Data is retrieved from the Dutch Organized Crime Monitor and from Dutch Judicial Documentation System (JDS) that report respectively information on organized crime, and information on all judicial contacts that are registered at the Dutch Public Prosecutor's Office. The sample includes 746 organized crime offenders born in the Netherlands, and a comparison group of 109,719 general offenders also born in the Netherland and that resemble the organized crime offenders regarding the age distribution.		
Wood et al. (2017)	Examine how gang members, gang affiliates, and violent men compare on mental health symptoms and traumatic experiences.	UK	Gang	Gang affiliates; Violent men	Survey (questionnaires)	The authors compare demographics of gang members with affiliates; affiliates with violent men; and gang members with violent men using multinomial logistic regressions to identify potential differences among groups.	Age; Anxiety; Being in a relationship; Depression; Economic condition; Ethnicity; Foreign born; Living conditions/household (adulthood); Low self‐control; Negative life events; Psychopathy and antisocial personality disorder; Violence
					Data is retrieved from the self‐administrated questionnaire for the Men's Health Survey conducted in the United Kingdom in 2011. The sample includes 1,539 adult British males who self‐identified themselves as violent men (n = 1,312), gang members (n = 108), or gang affiliates (n = 119).		

##### Countries

The included studies were conducted in the United States (*n* = 7), the United Kingdom (*n* = 4), Denmark, Italy, the Netherlands (*n* = 2 each), Brazil, and Mexico (*n* = 1 each).

##### Organized crime membership

Nearly half of the included studies analyzed exclusively or mainly adult gangs. Four studies examined outlaw motorcycle gangs (Blokland et al., 2019; Danner & Silverman, 1986; Klement, 2016; Pedersen, 2018), two studies drug‐trafficking organizations (Carvalho & Soares, 2016; Ostrosky et al., 2012), and two studies mafia organizations (Bottini et al., 2017; Schimmenti et al., 2014). Three studies analyzed other organized crime groups (Francis et al., 2013; Kirby et al., 2016; Van Koppen et al., 2010). Only one study analyzed both members of outlaw motorcycle gangs and members of other gangs (Pedersen, 2018).

The included studies provided different approaches to the selection of organized crime members. The most frequent approach (n = 8) relied on interviews with individuals involved in organized crime groups. The samples were drawn from inmates (Bottini et al., 2017; Kissner & Pyrooz, 2009; Ostrosky et al., 2012; Schimmenti et al., 2014) or from specific areas/populations (Carvalho & Soares, 2016; Decker et al., 2014; Levitt & Venkatesh, 2001; Pyrooz et al., 2015). Organized crime membership was determined through self‐nomination, convictions/charges, or authors' assessments.

Four included studies exploited police intelligence or investigation data to identify individuals involved in organized crime groups (Adams & Pizarro, 2014; Blokland et al., 2019; Klement, 2016; Pedersen, 2018).

Four included studies employed surveys of the general population (Coid et al., 2013; Wood et al., 2017), or of inmates (Danner & Silverman, 1986; Sharpe, 2002). Organized crime membership was determined through self‐nomination in the surveys.

Three included studies identified organized crime members through official registers of offenders (Francis et al., 2013; Kirby et al., 2016) or prosecuted individuals (Van Koppen et al., 2010), including in the organized crime sample individuals convicted or prosecuted for specific offences.

##### Comparison groups

The included studies differed for type and number of comparison groups used to assess the characteristics of organized crime members. Most studies confronted organized criminal groups with only one comparison group (*n* = 13), while six studies confronted organized criminal groups with two distinct comparison groups (Blokland et al., 2019; Bottini et al., 2017; Coid et al., 2013; Francis et al., 2013; Kirby et al., 2016; Wood et al., 2017).

Most comparisons were with samples from offenders in general (*n* = 10) or from the general population (*n* = 9). Some studies compared organized crime members with serious offenders (Adams & Pizarro, 2014; Francis et al., 2013; Kirby et al., 2016), violent men (Coid et al., 2013; Wood et al., 2017), and gang affiliates (i.e., non‐members associated with a gang) (Wood et al., 2017).

Three studies compared current gang members with former gang members in addition to non‐gang individuals (Decker et al., 2014; Kissner & Pyrooz, 2009; Pyrooz et al., 2015). The data on these comparisons were excluded from the systematic review to prevent possible biases in the assessment of the factors leading to recruitment into organized criminal groups due to the past involvement of former gang members.

Data on the comparison groups came from the same source of the data on the organized crime members (e.g., interview samples comprising both members and non‐members) or from distinct sources (e.g., national offices of statistics for a sample of the general population, national crime registers for samples of offenders in general).

##### Study design and analysis

Ten studies intended to assess the association between organized crime membership and possible risk factors (Blokland et al., 2019; Bottini et al., 2017; Danner & Silverman, 1986; Francis et al., 2013; Kirby et al., 2016; Kissner & Pyrooz, 2009; Ostrosky et al., 2012; Schimmenti et al., 2014; Sharpe, 2002; Wood et al., 2017). Other studies aimed to assess the impact of organized crime membership on offending or other characteristics of the criminal career (Francis et al., 2013; Klement, 2016; Pedersen, 2018; Van Koppen et al., 2010). The remaining studies had different objectives and normally included organized crime membership as a correlate. They aimed at assessing the level of gang embeddedness across different groups (Decker et al., 2014), estimating the selection and earnings in specific drug‐trafficking jobs based on econometric models (Carvalho & Soares, 2016), evaluating the use of mental health services (Coid et al., 2013), assessing internet usage (Pyrooz et al., 2015), or establishing years in education and income (Levitt & Venkatesh, 2001).

Most studies reported data as mean or percentage values for organized crime members and comparison groups across a variety of characteristics, often providing tests of statistical significance of the differences. For these studies, data extraction relied on bivariate relationships in descriptive statistics. Some studies also reported odds ratios, adjusted odd ratios or logistic regression coefficients (Blokland et al., 2019; Coid et al., 2013; Kissner & Pyrooz, 2009; Sharpe, 2002; Wood et al., 2017). In most cases the data reported in the studies allowed the computation of effect sizes. When information was incomplete, we attempted to contact the authors and integrate the data (see above under Missing data).

We were unable to extract most data from one study (Danner & Silverman, 1986). Remarkably, this is the oldest included quantitative study, and this possibly prevented us from retrieving contacts of the authors to integrate the reported information. The study compared members of OMCGs and offenders in general in the United States and reported on several factors including race, age, and offending.

#### 5.2.2 Qualitative studies

The 33 included qualitative studies are summarized below and in Table 3. The full references are provided in References to included studies.

**Table 3:** Characteristics of included qualitative studies

Study	Objectives	Country	Type of OCG	Subjects	Methods and analysis	Information & Data sources	Risk Factors (by category)
Albini (1971)	Analyze and describe of the social system of American OC in the United States.	US	Mafia	OC in the US from its origins to the second half of the 1960s.	Descriptive qualitative in‐depth analysis of available information.	(a) Interviews with police informants participating in OC.	Economic condition Motivation Silence/omertà Social environment
						(b) Interviews with law enforcement officers, unspecified number.	
						(c) Government reports, archival material, media.	
Ancrum and Treadwell (2017)	Examine commercial cannabis cultivation in England.	UK	Other OCG	N cannabis cultivators in disadvantaged locales in Central and Northern England after 2008.	Descriptive qualitative in‐depth analysis of available information.	(e) Extensive and long‐running ethnographic investigation of cannabis markets in Central and Northern England	Legitimate jobs/skills Motivation Social environment Violence
Arlacchi (1983)	Examine the evolution of the contemporary mafias from their origins to the period after the Second World War.	Italy	Mafia	The Sicilian mafia and the Calabrian 'Ndrangheta, from their origin to the evolution after the Second World War.	Descriptive qualitative in‐depth analysis of available information.	(c) Analysis of secondary sources from law enforcement, court cases and official reports, unspecified number and type.	Age Motivation Offence and/or contact with CJ system Social environment Violence
						(b) Interviews with law enforcement officials, unspecified number and type.	
						(c) Official statistics, police and court files, unspecified number.	
Arsovska (2015)	Analyze the causes, culture, structure, politics, action, and migration of ethnic Albanian OC groups.	Albania; Belgium; US; the Balkans	Other OCG	N Albanian OC groups and offenders since the 1990s.	Triangulation of information from different sources, methods, and countries.	(a) Interviews with 12 OC offenders.	Age Economic condition Ethnicity Motivation Social environment
						(b) Interviews with 70+ law enforcement officials; NGOs, international organizations, journalists, academics, victims of trafficking and smuggling, unspecified number and type; 60+ Albanian migrants in New York.	
						(c) 50+ police files and court cases from Belgium; 37 court cases from the US.	
						(e) Ethnographic participant observation in the Balkans, Western Europe and the US.	
						(f) Cross‐national survey with 864 ethnic Albanian respondents.	
Baird (2018)	Examine the pathways to obtaining masculine identities for gang members.	Colombia	Adult gang	N gang members in Medellín between 2006 and 2012.	Descriptive qualitative in‐depth analysis of available information.	(a) 40 life‐history interviews with gang members in Medellín, Colombia.	Economic condition Motivation Sex Social environment Troubled family environment Violence
						(e) Ethnographic fieldwork conducted intermittently between 2006 and 2012 in Medellín's poor north‐eastern corner, during fieldwork with a community‐based social organization.	
Brancaccio (2017)	Analyze Camorra clans, their social environment and some illicit enterprises they are involved in.	Italy	Mafia	N camorra clans, cigarette smugglers, and counterfeit clothes sellers in Naples since the end of the Second World War.	Descriptive analysis of the available numeric information, with focus on homicide trends and clans' turnover; descriptive qualitative in‐depth analysis of available information with a focus on family trees and clan organization.	(a) Interview with 1 OC member turned informant.	Economic condition Motivation Offence and/or contact with CJ system Social environment Violence
						(b) Interviews with 2 prosecutors and 1 criminal lawyer.	
						(c) Police and court files, including official crime data.	
						(d) Historical documents from the Naples State Archive.	
Brotherton and Barrios (2004)	Seek an alternative definition of “the gang”; Analyze the social movement possibilities of gang members, the form and content of gang reforms, and the societal reactions to the group's transformation.	US	Adult gang	67 male and female Almighty Latin Kings and Queens Nation members.	Descriptive qualitative in‐depth analysis of available information.	(a) 67 individual life history interviews gang members (males and females, young and old, longstanding members and new recruits, leaders, and rank‐and‐file); *N* outsiders who interacted with the gang members (nongroup family members, members of the clergy, defense lawyers, teachers, …).	Economic condition Ethnicity Motivation Sex
						(d) Letters used during the Luis Felipe trial; organization‐produced newsletters; copies of manifestos from New York and other affiliates; Website of the group; …).	
Chalas and Grekul (2017)	Explore motivations for joining and leaving gangs.	Canada	Adult gang	175 male and female adult (ex) gang members in the correctional system.	Descriptive qualitative in‐depth analysis of available information.	(a) 175 semi structured interviews to gang members in the correctional system.	Ethnicity Motivation Offence and/or contact with CJ system Social environment Violence
						(d) Background information of the gang members interviewed.	
Cressey (1969)	Explore the internal structure and the activities of the Italian American mafia.	US	Mafia	The Italian American mafia in the US between since its origin	Descriptive qualitative in‐depth analysis of available information.	(b) Interviews with law enforcement officials, unspecified number and type.	Age Ethnicity Legitimate jobs/skills Motivation Silence/omertà
						(c) Unspecified number and type of judicial/police documents.	
Decker and Chapman (2008)	Understand the steps drug smugglers take in reducing their risks of being caught, losing a load, or being ripped off; and points at which a smuggler may have been deterred.	US	DTO	34 drug smugglers smuggling drugs into the US	Descriptive qualitative in‐depth analysis of available information.	(a) In‐depth interviews with 34 of the highest‐level drug smugglers confined in federal institutions at the time of the study, with questions designed to collect information on how high‐level smugglers assess risk, what they perceive as risks, and how these perceptions vary according to their role in the offense.	Economic condition Ethnicity Legitimate jobs/skills Motivation Offence and/or contact with CJ system Social environment
Densley (2012)	Examine the recruitment into street gangs through the signaling theory.	UK	Adult gang	69 self‐nominated gang members or gang associates across 12 London Gangs.	Descriptive qualitative in‐depth analysis of available information.	(a) Interviews with 69 gang members and gang associates.	Ethnicity Offence and/or contact with CJ system Social environment Violence
						(b) Interviews with 129 friends, relatives, romantic partners, unaffiliated youths, law enforcement officers, social workers.	
						(e) observation of gang set space	
Gambetta (1993)	Examine the Sicilian mafia as a private protection industry.	Italy	Mafia	The Sicilian mafia as a set of firms offering private protection.	Descriptive qualitative in‐depth analysis of available information. Focus on the firm's market, resources, trademarks, mechanisms	(b) Interviews with 26 businessmen and economic agents.	Legitimate jobs/skills Motivation Offence and/or contact with CJ system Sex Silence/omertà
						(c) Analysis of secondary sources from law enforcement, court cases and official reports, unspecified number and type.	
						(e) Observation of some specific markets in Palermo.	
Gordon (2000)	Determine why individuals get involved in gangs.	Canada	Adult gang	128 adult known gang members in the Greater Vancouver area in 1995.	Descriptive qualitative in‐depth analysis of available information and content analysis.	(a) 33 interviews with adult gang members.	Age Ethnicity Motivation Sex
						(b) Family visits and interviews.	
						(c) Review of case files for all subjects.	
Hess ([1970] 1998)	Analyze the Sicilian mafia as a social system. Examine the social structure, the mafiosi, the structure of mafia groups, and the functions of the mafia.	Italy	Mafia	The Sicilian mafia between the 1860s and 1960s.	Descriptive qualitative in‐depth analysis of available information and content analysis.	(c) Analysis of secondary sources from law enforcement, court cases and official reports, unspecified number and type.	Economic condition Motivation Offence and/or contact with CJ system Silence/omertà Social environment Violence
						(d) Analysis of historical documents from the Palermo State Archives.	
Hixon (2010)	Examine the initiation rituals, mythology, symbols, and behaviors of a White supremacist street gang through analytical psychology.	US	Adult gang	8 White supremacist male gang members.	Descriptive qualitative in‐depth analysis of available information and content analysis.	(a) 8 semi‐structured interviews.	Age Ethnicity Legitimate jobs/skills Motivation Psychopathy and antisocial personality disorder Sex
						(e) Subjective observational data (silent communications including body posture, eye movement, and facial expression); and objective observational data (type of clothing worn, arrival time, …).	
						(d) Personal history documentation and literature review used for distribution and studied by the gang members.	
Ianni and Reuss‐Ianni (1972)	Analyze the social organization and the internal relationship of an Italian American OC family	US	Mafia	The Lupollo family, fictive name for an OC family based in New York from its origin to the 1960s.	Descriptive qualitative in‐depth analysis of available information.	(a) Interviews with the core members of the family and other individuals involved with other families.	Social environment
						(c) Police files to develop kinship and network charts.	
						(e) Three years of participant observation with several members of the family	
Kemp, Zolghadriha, and Gill (2020)	Analyze the engagement processes and pathways into OC	n/a	Other OCG	100 individuals engaged in OC in different periods and countries	Mixed methods approach: content analysis of (auto/) biographies; and binary logistic regressions aiming at establishing the risk factors for joining and/or forming an OCG through.	(g) 83 biographies and autobiographies of individuals involved in OC	Age Motivation Negative life events Offence and/or contact with CJ system Social environment
Kleemans and De Poot (2008)	Understand how people become involved in OC, why certain offenders “progress” to certain types of OC whereas others become involved only later in life, and whether the main findings of developmental and life‐course criminology are applicable to OC.	The Netherlands	Other OCG	979 suspects involved in 79 different OC cases for which prosecution started in the period 1995–1999.	Descriptive analysis of the available numeric information; and descriptive qualitative in‐depth analysis of available information.	(c) Judicial/police documents from the Dutch Organized Crime Monitor, unspecified number and type.	Legitimate jobs/skills Motivation Negative life events Offence and/or contact with CJ system Social environment
Kleemans and Van de Bunt (2008)	Understand the connection between occupations, work relations, work settings, and OC activities.	The Netherlands	Other OCG	120 large scale criminal investigations involving 1623 suspects, completed in the period 1996‐2006.	Descriptive analysis of the available numeric information; and descriptive qualitative in‐depth analysis of available information.	(b) Structured interviews with police officers and public prosecutors, unspecified number and type.	Legitimate jobs/skills Social environment
						(c) Judicial/police documents from the Dutch Organized Crime Monitor, unspecified number and type.	
Knox et al. (1997)	To clarify the facts about gang life in the US	US	Adult gang	10,166 confined offenders, of which 4140 self‐reported gang members.	Descriptive analysis of the available numeric information (self‐report methodology).	(f) Survey distributed in 17 states in 85 different correctional facilities (prisons, boot camps, juvenile institutions, etc.).	Ethnicity Sex
Leukfeldt et al. (2019)	Explore how the use of information technology affects the processes of origin and growth of criminal networks	The Netherlands	Other OCG	30 large scale criminal investigations completed in the period 2011–2016.	Descriptive qualitative in‐depth analysis of available information.	(c) Judicial/police documents from the Dutch Organized Crime Monitor (include traditional OC, traditional OC in which IT is an innovative element, low tech cybercrimes and high‐tech cybercrimes), unspecified number.	Ethnicity Legitimate jobs/skills Offence and/or contact with CJ system Social environment
May and Bhardwa (2018)	Explore the routes into OC and fraud.	UK	Other OCG	31 convicted fraudsters	Descriptive qualitative in‐depth analysis of available information.	(a) Interviews to 31 convicted fraudsters.	Legitimate jobs/skills Motivation Negative life events Social environment
Paoli (2003)	Analyze the structure, functioning, activities of the Italian mafias	Italy	Mafia	The Italian mafias, OC groups belonging to the Sicilian mafia, the Neapolitan Camorra, and the Calabrian 'Ndrangheta	Descriptive qualitative in‐depth analysis of available information.	(b) Interviews with law enforcement, local politicians, and anti‐mafia activists, unspecified number and type.	Ethnicity Motivation Offence and/or contact with CJ system Silence/omertà Social environment
						(c) Secondary sources from law enforcement, court cases and official reports, including confessions by OC members.	
Pedersen (2018)—Unpublished	Analyze the motivations and processes for joining a gang.	Denmark	Outlaw motorcycle gang	15 male offenders involved in adult gangs in Denmark.	Descriptive qualitative in‐depth analysis of available information.	(a) Interviews with 15 male offenders once gang involved.	Ethnicity Motivation Silence/omertà Social environment Violence
Radaelli et al. (2019)	Understand how external agents penetrate professional organizations, recruit professionals in their misconduct projects, and keep them under control.	Italy	Mafia	N professors at the Troy University investigated in 2000–2015.	Descriptive qualitative in‐depth analysis of available information. Longitudinal analysis of the misconduct strategies by the clan.	(c) 2 arrest warrants and 4 verdicts.	Age Legitimate jobs/skills
						(d) Newspaper articles, documentary transcripts, reportages, parliament reports.	
Spapens and Moors (2019)	Observe criminal behavior and involvement in OC groups and their leadership in successive generations. Understand the transmission mechanisms.	The Netherlands	Other OCG	7 OC families who lived in the south of the Netherlands for at least three generations	Descriptive qualitative in‐depth analysis of available information. Focus on the reconstruction of families' composition combined members' criminal records.	(b) Interviews with 14 current and former police/investigative officers, 4 probation officers, 3 welfare workers, 4 historians of North Brabant.	Education Sex Social environment Troubled family environment Violence
						(c) Files of criminal investigation cases, unspecified number and type.	
						(d) Historical archives (court, local police, municipal and regional administrative bodies).	
Van Dijk, Kleemans, and Eichelsheim (2019)	Explore the extent and the mechanisms of intergenerational continuity of crime in families of OC offenders. discontinuity.	The Netherlands	Other OCG	25 OC offenders based in Amsterdam with one or more children between 19 and 33 years old.	Descriptive analysis of the available numeric information and qualitative in‐depth analysis of text information.	(a) Three interviews with employees of the involved organizations.	Social environment
						(c) Police files and judicial data of all the family members (father, mother and children); and child protection files.	
						(d) Media search on the fathers.	
Van Koppen and De Poot (2013)	Understand how individuals become involved in OC by studying mechanisms and opportunities at different life stages for early versus late onset offenders.	The Netherlands	Other OCG	16 inmates convicted and imprisoned for OC	Descriptive qualitative in‐depth analysis of available information and content analysis.	(a) In‐depth semi‐structured interviews with 16 inmates all convicted and imprisoned for OC.	Legitimate jobs/skills Offence and/or contact with CJ system Social environment Troubled family environment
						(c) Verdicts and rap sheets.	
Van Koppen (2013)	Understand why and how individuals become involved in OC	The Netherlands	Other OCG	15 large‐scale police investigations from in the period 1994‐2006.	Descriptive qualitative in‐depth analysis of available information and content analysis.	(c) Judicial/police documents from the Dutch Organized Crime Monitor, mainly police files and rap sheets, unspecified number.	Legitimate jobs/skills Motivation Negative life events Offence and/or contact with CJ system Social environment
Van Koppen et al. (2010)	Whether or not there is a relationship between trajectories in OC and the kind of criminal activities people are engaged in.	The Netherlands	Other OCG	120 large scale criminal investigations completed in the period 1996–2006.	Descriptive analysis of the available numeric information; and Descriptive qualitative in‐depth analysis of available information.	(c) Judicial/police documents from the Dutch Organized Crime Monitor, mainly police files and rap sheets, unspecified number.	Age Offence and/or contact with CJ system
Van San and Sikkens (2017)	Understand how women become connected to drug‐trafficking networks; and what are the differences between recruitment regions historically connected to the Netherlands (Curacao) or not (Peru)	The Netherlands	DTO	N female drug mules in two prisons in the Netherlands; and N Curacao families in the Netherlands, whose members were active in the drug economy over 1993–1997, 2004–2006, and 2006–2008.	Descriptive qualitative in‐depth analysis of available information.	(e) Ethnographic research among female drug mules in two prisons in the Netherlands, and ethnographic research among female drug mules in two Peruvian women's prisons.	Economic condition Sex Social environment
Varese (2011)	Analyze the movement of mafia groups to new locations.	Italy	Mafia	Several case‐studies of successful/unsuccessful instances of mafia movement from the 'Ndrangheta, the Russian Mafia, the Sicilian Mafia, the Chinese Triads.	Descriptive qualitative in‐depth analysis of available information. Focus on factors and conditions favoring/impeding the movement of mafias to new locations.	(a) Interviews, unspecified number/type.	Economic condition Offence and/or contact with CJ system Social environment Violence
						(b) Interviews with key informants, unspecified number and type.	
						(c) Judicial/police documents, unspecified number and type.	
						(d) Historical documents, unspecified number and type.	
Zhang and Chin (2002)	Uncover the attributes of Chinese human smuggling organizations to understand how snakeheads assume roles or perform fundamental functions.	China	Other OCG	90 individuals directly involved in human smuggling networks from China to the US	Descriptive qualitative in‐depth analysis of available information and content analysis.	(a) Structured interviews with 90 individuals directly involved in human smuggling networks from China to the US selected through ethnographic strategies (community contacts and snowball technique).	Education Ethnicity Legitimate jobs/skills Sex Social environment
						(e) Field observation in human smuggling networks from China to the US	

##### Countries

Most studies focused on organized crime groups in one specific country. Only two studies covered different countries (Arsovska, 2015; Kemp et al., 2020). The most frequently studied countries were the Netherlands (nine studies), Italy and the United States (seven studies each), and the United Kingdom (three studies).

##### Organized crime membership

Thirteen studies focused on other organized crime groups. Ten studies focused on mafias and seven studies on adult gangs. Only two studies examined drug‐trafficking organizations and one study outlaw motorcycle gangs.

##### Study design and analysis

Sixteen studies were peer reviewed journal articles and 11 were research monographs. The rest included two book chapters, two unpublished papers, one research report, and one dissertation.

Only 11 studies addressed directly the recruitment into organized criminal groups among the main objectives (Ancrum & Treadwell, 2017; Chalas & Grekul, 2017; Densley, 2012; Gordon, 2000; Kemp et al., 2020; Kleemans & De Poot, 2008; May & Bhardwa, 2018; Pedersen, Unpublished; Van Koppen & De Poot, 2013; Van Koppen, 2013). The other studies mainly focused on other topics, although they provided information on the recruitment into organized crime in the process. These also included research monographs, which often addressed a variety of objectives and topics relating to organized crime.

We classified the data sources used by qualitative studies into seven different categories (seventh column in Table 3). Twelve studies used data only from one type of source, 10 studies relied on two sources, eight studies on three sources, two studies used information from four types of sources (Brancaccio, 2017; Varese, 2011), while only one study relied on information from five different source categories (Arsovska, 2015).

Regarding the most frequent sources of information and methodologies, 20 studies relied on judicial and or police documentation. Seventeen studies interviewed current or former organized crime offenders. Twelve studies conducted interviews with key informants not directly involved in organized crime groups. Nine studies resorted to ethnographic participant observation, while eight studies examined historical documentation. Only two studies conducted surveys and one study examined biographies and autobiographies of organized crime offenders.

### 5.3 Quality assessment of the included studies

#### 5.3.1 Risk of bias assessment of included quantitative studies

For each of the included quantitative studies, we conducted the risk of bias assessment using a document coding protocol consisting of 28 items (items 58–85 of Supporting Information Appendix C: Document coding protocol, see also Quality assessment of the included studies). Results are presented by summary items and through the traffic light model adapted from De Vibe et al. (2012) (Table 4). A detailed description of results is provided in Table 16 in Supporting Information Appendix D: Risk‐of‐bias assessment of the included quantitative studies.^10^

**Table 4:** Risk of bias assessment for the eligible quantitative studies

Study reference	a. Sampling and setting	b. Risk factors and outcomes	c. Statistical procedures	d. Overall study RB
Adams and Pizarro (2014)	Low	High	Low	High
Blokland et al. (2019)	High	High	High	High
Bottini, Fiorina, and Salvato (2017)	Unclear	High	Low	High
Carvalho and Soares (2016)	High	High	Low	High
Coid et al. (2013)	Unclear	High	Low	High
Danner and Silverman (1986)	High	High	Low	High
Decker et al. (2014)	High	High	Low	High
Francis et al. (2013)	Low	High	Low	High
Kirby et al. (2016)	Low	High	Low	High
Kissner and Pyrooz (2009)	Unclear	High	Low	High
Klement (2016)	Low	Low	Low	Low
Levitt and Venkatesh (2001)	Unclear	High	Low	High
Ostrosky et al. (2012)	Unclear	High	Low	High
Pedersen (2018)	Low	Low	Low	Low
Pyrooz, Decker, and Moule (2015)	High	High	Low	High
Schimmenti et al. (2014)	Unclear	High	Low	High
Sharpe (2002)	Low	High	High	High
Van Koppen, de Poot, and Blokland (2010)	Low	Low	Low	Low
Wood, Kallis, and Coid (2017)	Unclear	High	Low	High

The risk of bias assessment shows that most studies (16 out of 19) have a high risk of bias. The overall high score of risk of bias is mainly due to the research design of the included studies, as their cross‐sectional nature introduces a large risk of bias for inferential interpretation. We were able to retrieve retrospective information (or time‐invariant factors) from several studies, though most of the information collected from included studies consisted of factor categories classified as correlates. Most studies provided appropriate information on data collection and statistical procedures, reporting complete descriptive tables for both the characteristics of the sample and the statistical analysis.

Several studies presented issues related to the use of prisoner samples, including lack of transparent selection of the eligible study participants and small sample size. Small sample size is often due to safety and security reasons and researchers' limitations in getting access to prisoners for interviews and testing. Authors of two included studies, explicitly reported that they were not granted access to a subset of the prisoners in their focus institutions (Kissner & Pyrooz, 2009; Schimmenti et al., 2014).

Studies using official data (administrative, judicial, or police files) may include large organized crime samples that can be analyzed together with comparable samples of non‐organized‐crime members obtained through matching statistical techniques. Studies employing this analytical approach resulted in having an overall low risk of bias (Klement, 2016; Pedersen, 2018; Van Koppen et al., 2010). Moreover, studies employing sample matching were also the only ones providing some longitudinal analysis (Blokland et al., 2019; Francis et al., 2013; Kirby et al., 2016; Klement, 2016; Pedersen, 2018; Van Koppen et al., 2010). Nonetheless, these studies mostly focus on demographic and criminal history data, unlike survey‐based and interview‐based studies that report more varied type of information (including demographic, economic, psychological, and criminal history variables).

In conclusion, the risk of bias assessment highlights that most of the included quantitative studies have a high risk of bias, pointing out that results of this systematic review should be interpreted with caution.

#### 5.3.2 Quality assessment of included qualitative studies

We assessed the quality of the included qualitative studies and the qualitative parts of included mixed‐method studies through a 5‐item checklist adapted from the CASP Qualitative Checklist (Critical Appraisal Skills Programme, 2018). Table 5 reports the results of the assessment.

**Table 5:** Quality assessment for the eligible qualitative studies

Study reference	a. Clear aim on recruitment	b. Research design	c. Data collection	d. Data analysis rigorous	e. Clear statement of finding
Albini (1971)	NO	YES	NO	YES	NO
Ancrum and Treadwell (2017)	YES	YES	YES	YES	YES
Arlacchi (1983)	NO	YES	NO	NO	NO
Arsovska (2015)	NO	YES	YES	YES	YES
Baird (2018)	NO	YES	YES	YES	YES
Brancaccio (2017)	NO	YES	YES	YES	YES
Brotherton and Barrios (2004)	NO	YES	YES	YES	YES
Chalas and Grekul (2017)	YES	YES	YES	YES	YES
Cressey (1969)	NO	YES	NO	NO	YES
Decker and Chapman (2008)	NO	YES	YES	YES	YES
Densley (2012)	YES	YES	YES	YES	YES
Gambetta (1993)	NO	YES	NO	NO	YES
Gordon (2000)	YES	YES	YES	YES	YES
Hess ([1970] 1973)	NO	YES	NO	YES	NO
Hixon (2010)	NO	YES	YES	YES	YES
Ianni and Reuss‐Ianni E (1972)	YES	YES	YES	YES	YES
Kemp, Zolghadriha, and Gill (2020)	YES	YES	YES	YES	YES
Kleemans and de Poot (2008)	YES	YES	YES	YES	YES
Kleemans and Van de Bunt (2008)	NO	YES	YES	YES	YES
Knox et al. (1997)	NO	YES	YES	YES	YES
Leukfeldt et al. (2019)	NO	YES	YES	YES	YES
May and Bhardwa (2018)	YES	YES	YES	YES	YES
Paoli (2003)	NO	YES	YES	YES	YES
Pedersen (2018)—Unpublished	YES	YES	YES	YES	YES
Radaelli et al. (2019)	NO	YES	YES	YES	YES
Spapens and Moors (2019)	NO	YES	YES	YES	YES
Van Dijk et al. (2019)	NO	YES	YES	YES	YES
Van Koppen and De Poot (2013)	YES	YES	YES	YES	YES
Van Koppen (2013)	YES	YES	YES	YES	YES
Van Koppen et al. (2010)	NO	YES	YES	YES	YES
Van San and Sikkens (2017)	YES	YES	YES	YES	YES
Varese (2011)	NO	YES	YES	YES	YES
Zhang and Chin (2002)	NO	YES	YES	YES	YES

Overall, only 12 out of the 33 included qualitative studies satisfied all the five items of the checklist. All twelve studies were articles published in peer‐ reviewed journals, which explicitly addressed the recruitment into organized criminal groups among the main objectives of the analyses, provided detailed information on the methods, and presented and discussed the results in detail.

The remaining 21 studies failed to meet all items of the checklist, with the first item reporting the highest frequency of negative assessment. While all these studies included relevant considerations on possible drivers of recruitment into organized crime, these were rarely the focus of the analyses. Consequently, it was difficult to find extensive details on specific factors.

Considering the variety of qualitative research methods, all studies adopted appropriate research designs to examine, among the various objectives, also the recruitment into organized crime (item 2). Nevertheless, some studies offered limited detail on the source of information, as reported by item 3 (Albini, 1971; Arlacchi, 1983; Cressey, 1969; Gambetta, 1993; Hess, 1970/1973). Remarkably, these were all research monographs published until the early 1990s and offering broader analyses on the nature of organized crime. Only one study from the same period dedicated an entire chapter to the presentation of the sources, data collection and analysis (Ianni & Reuss‐Ianni, 1972). Overall, some classic studies in the field offer less methodological detail, possibly due to the evolution of research standards.

Most of the studies presented detailed, rigorous analyses of the data and reported a clear statement of the main findings (items 4 and 5). They offered critical considerations on the reliability of the findings, attempted to triangulate across distinct sources, discussed the results in the context of the previous literature and addressed possible limitations of the analyses.

In conclusion, the quality assessment suggests that, while generally well‐designed, only a minority of the included studies addressed the recruitment into organized criminal groups as one of the main objectives of the analysis. Studies failing to do so offered limited amount of information on the factors leading to recruitment.

### 5.4 Synthesis of results

Following the reviewers' requests and the protocol, we have extracted data from quantitative, qualitative, and mixed methods studies. As already mentioned, all mixed methods studies were included only for their empirical qualitative parts.

For quantitative studies, as described in the “Determining independent findings” section, we paired six studies reporting on the same data: Francis et al. (2013)/Kirby et al. (2016), Decker et al. (2014)/Pyrooz et al. (2015), and Coid et al. (2013)/Wood et al. (2017). Furthermore, we considered the data by Pedersen (2018) as distinct data sets (reported below as “Pedersen, 2018—OMCG” and “Pedersen, 2018—Gang,” respectively). Overall, this process led to a total of 17 data sources (henceforth studies) to extract relevant effect sizes. All qualitative and mixed methods studies reported on different populations or samples; thus, no pairing was necessary.

Findings from all studies were classified into a common categorization system inductively identified from the data. There is some overlap between quantitative and qualitative studies, but also categories with only one type of studies (Table 6). We decided to expose the results by category in alphabetical order to simultaneously present the readers with findings from qualitative studies and qualitative studies.

**Table 6:** Number of quantitative/qualitative studies by factor category

Category	Quantitative studies	Qualitative studies
Age	10	8
Anxiety	2	
Being in a relationship	3	
Cognitive functioning	2	
Criminal versatility	4	
Depression	2	
Economic condition	6	9
Education	7	2
Ethnicity	8	13
Foreign born	4	
Internet use and technological capacity	1	
Legitimate jobs/skills		13
Living conditions/household (adulthood)	3	
Low self‐control	6	
Motivation	1	19
Negative life events	3	4
Offence and/or contact with CJ system	6	15
Offence type	4	
Psychopathy and antisocial personality disorder	4	1
Religious beliefs	2	
Sanctions	5	4
Sex	5	9
Silence/omertà		6
Social environment	2	25
Troubled family environment	5	3
Violence	6	10

#### 5.4.1 Synthesis of quantitative studies

The synthesis of results draws from the seventeen quantitative studies allowing to extract sufficient data to compute effect sizes. We were unable to extract most data from one study (Danner & Silverman, 1986) nor to retrieve email contacts of the authors. Overall, we identified 407 measures. Information was insufficient for 24 measures, and we were unable to retrieve it from the authors and integrate the studies. Furthermore, 18 measures were excluded as duplicates or because the underlying constructs were unclear and could not be verified with the authors. Lastly, out of 38 measures reported in two paired reports for the same study, we retained only 19 measures (one per study). This process led to a total of 346 measures we could extract effect sizes from. We classified the measures into mutually exclusive factor categories and, where applicable, sub‐categories (total measures per categories are reported in the last column of Table 7, with the number of source studies in parenthesis).

**Table 7:** Distribution of measures by category and by factor type and inclusion into the meta‐analyses

	Included in the meta‐analyses^a^		Not included in the meta‐analyses^b^		Total measures (studies)
Category	Predictors (n of studies)	Correlates (n of studies)	Predictors (*n* of studies)	Correlates (n of studies)	
Age		15 (10)			15 (10)
Anxiety		9 (2)			9 (2)
Being in a relationship		5 (3)			5 (3)
Cognitive functioning		41 (2)			41 (2)
Criminal versatility	2 (2)	5 (2)			7 (4)
Depression		5 (2)			5 (2)
Economic condition		17 (5)	2 (1)		19 (6)
Education		12 (7)		1 (1)	13 (7)
Ethnicity	24 (8)		1 (1)		24 (8)
Foreign born	7 (4)				7 (4)
Internet use and technological capacity				9 (1)	9 (1)
Living conditions/household (adulthood)		3 (2)		12 (1)	15 (3)
Low self‐control		18 (6)	1 (1)		19 (6)
Motivation				1 (1)	1 (1)
Negative life events		21 (2)	1 (1)		22 (3)
Offence and/or contact with CJ system	18 (4)	8 (3)	2 (1)	2 (1)	30 (6)
Offence type	20 (2)	11 (2)			31 (4)
Psychopathy and antisocial personality disorder		19 (4)			19 (4)
Religious beliefs			3 (1)	1 (1)	4 (2)
Sanctions		8 (4)	2 (1)		10 (5)
Sex	6 (5)				6 (5)
Social environment		4 (2)	2 (1)		6 (2)
Troubled family environment		4 (4)	2 (1)		6 (5)
Violence	5 (3)	17 (4)			22 (6)
Grand total	82 (12)	222 (13)	16 (6)	26 (4)	346 (17)

^a^ Included measures were synthetized (when two or more measures from the same study fell into one category/subcategory) and used for meta‐analyses by categories. When possible, that is, when at least two effect sizes from at least two data sets were available, meta‐analyses by subcategories were performed.

^b^ Measures not included in the meta‐analyses were excluded because only one study was available for a factor category/subcategory. In some cases, measures were not included as they measured risk factors conceptually different from the other risk factors in the category/subcategory.

In line with the literature (see Higginson et al., 2018), we further divided the measures into either predictors or correlates depending on the likely causal relation between the factors and the recruitment into organized crime. Measures classified as predictors are time‐invariant factors (e.g., ethnicity, sex) or variables measured before onset of organized crime membership (e.g., prior violent offences). Measures classified as correlates are all other factors, including those for which it impossible to assess whether the reported estimates were measured before onset of organized crime membership (see Table 7 for the total number of measures and source studies by factor category and predictors/correlates).

We then extracted effect sizes from all the 346 measures.

Out of the total number of effect sizes (*n* = 346), 12.1% (*n* = 42) were not included in the meta‐analyses. The effect sizes not included in the meta‐analyses belonged to factor categories or subcategories comprising only one study, thus making it impossible to conduct a meta‐analysis. In a few cases, we also excluded from the meta‐analyses risk factors conceptually or operationally different from all other risk factors. We reported these effect sizes in the results, separately from the meta‐analysis.

Overall, 304 effect sizes were eligible for meta‐analysis (Table 7, column “Included in the meta‐analyses”). We synthesized the eligible effect sizes to ensure that only one independent effect size per study contributed each meta‐analysis (see Determining independent findings section and Data synthesis). We followed the same procedure also for effect sizes not included in the meta‐analyses (whenever one study reported multiple measures for the same construct). The synthesis produced 138 independent effect sizes at the factor category level.^11^

We used the 138 independent effect sizes to perform random‐effects meta‐analyses whenever a category/subcategory comprised at least two independent effect sizes measuring conceptually comparable constructs. We performed 25 meta‐analyses at factor category level to investigate a total of 21 factor categories (Table 7, column “Included in the meta‐analyses”): 7 were meta‐analyses of predictors and 18 meta‐analyses of correlates.^12^ Furthermore, when possible, we conducted meta‐analyses at the subcategory level.

Table 8 reports results for predictors for factor category and subcategories (if present), and it is ordered by the number of independent estimates for each category (*N*) and size of the estimate. To facilitate interpretation, we also report the odds ratios (OR), derived from the average log OR calculated in the analyses.

**Table 8:** Summary of results for predictors by factor category and subcategory

Category	Subcategory	*N*	OR	log OR	LL	UL	*I* ^2^ (%)	*τ* ^2^
Ethnicity	Any non‐White	6	1.90	0.64	−0.20	1.48	94.5*	0.972
	Black	6	1.70	0.53	−0.01	1.18	93.5*	0.353
	White	4	0.51	−0.67	−1.11	−0.23	75.7*	0.140
	Mixed race	1	0.68	−0.38	−0.65	−0.10	‐	‐
Sex (Male)		5	2.03	0.71	0.50	0.93	0	0
Foreign born		4	0.87	−0.14	−0.7	0.42	76.9*	0.206
Offence and/or contact with CJ system	All	4	1.51	0.41	−0.41	1.22	91.7*	0.326
	Ever convicted/fined	3	2.86	1.05	0.87	1.22	0	0
	N. of convictions	2	1.31	0.27	−0.96	1.49	90.0*	0.703
	Age first offence/conviction	2	0.86	−0.15	−0.21	−0.09	0	0
	Career duration	1	1.77	0.57	−0.52	1.66	‐	‐
Violence	All	3	1.68	0.52	0.14	0.91	98.7*	0.097
	Violent offences	3	1.67	0.51	0.12	0.9	78.2*	0.079
	Violent first offence	2	1.52	0.42	−0.02	0.86	89.3*	0.090
Criminal versatility		2	1.08	0.08	−0.03	0.2	0	0
Offence type	First offence: weapon	2	1.15	0.5	0.26	0.73	0	0
	Other offences	2	0.78	0.41	0.1	0.73	26	0.013
	First offence: other	2	1.43	0.36	−0.59	1.31	86.6*	0.008
	Drug offences	2	0.67	0.14	−0.02	0.30	0	0
	Property offences	2	0.46	−0.21	−0.30	−0.13	0	0
	First offence: drugs	2	1.65	−0.25	−0.51	0.02	18.6	0.008
	First offence: property	2	1.51	−0.4	−0.53	−0.28	0	0
	Weapon offences	2	0.81	−0.67	−2.84	1.5	99.1*	2.426
	Sexual offences	2	0.47	−0.76	−2.44	0.92	42.3	0.632
	First offence: sexual	2	0.51	−0.77	−2.99	1.45	75.6*	1.991
Economic condition	Risk	1	1.23	0.21	−0.03	0.45	‐	‐
Low self‐control		1	4.76	1.56	−0.47	3.60	‐	‐
Negative life events		1	1.45	0.37	−0.06	0.81	‐	‐
Religious beliefs		1	1.11	0.10	−0.09	0.30	‐	‐
Sanctions		1	1.95	0.67	0.53	0.80	‐	‐
Social environment		1	24.29	3.19	2.21	4.16	‐	‐
Troubled family environment		1	24.29	3.19	2.21	4.16	‐	‐

* Significant heterogeneity (*p* < 0.05).

The included studies enabled calculation of 32 associations with predictors at the category or subcategory level. However, 23 associations relied only on one or two independent measures, pointing out the scarcity of evidence for these factors. Six associations included three or four independent effect sizes, providing an average amount of evidence. Only three associations (ethnicity—any non‐White, ethnicity—Black, male sex) comprised five or six independent measures.

Table 9 reports the results for correlates, ordered by the number of independent estimates (*N*) and size of the estimate. To facilitate interpretation, we also report the odds ratios, derived from the log OR calculated in the analyses.

**Table 9:** Summary of results for correlates by factor category and subcategory

Category	Subcategory	*N*	OR	log OR	LL	UL	*I* ^2^ (%)	*τ* ^2^
Age		10	0.72	−0.33	−0.88	0.22	89.4*	0.649
Education	All	7	0.55	−0.60	−1.30	−0.18	82.6*	0.235
	Years of education	6	0.75	−0.29	−0.51	−0.07	0	0
	High school	2	0.14	−1.98	−4.04	0.08	83.8*	1.865
	Parental education	1	0.96	−0.04	−0.42	0.34	‐	‐
Low self‐control	All	6	2.01	0.70	0.08	1.32	89.3*	0.458
	Low self‐control (subcategory)	3	1.13	0.88	0.84	0.92	0	0
	Drug use and addiction problems	3	2.41	0.12	−2.79	3.04	95.7*	6.325
Psychopathy and antisocial personality disorder	All	4	5.87	1.77	−1.51	5.04	98.4*	10.939
	Psychopathy	3	7.92	2.07	−3.58	7.72	98.9*	24.66
	Antisocial personality disorder	2	1.67	0.51	−0.27	1.29	0	0
Sanctions	All	4	2.34	0.85	0.55	1.15	8	0.017
	Sanction seriousness	4	2.34	0.85	0.39	1.31	91.2*	0.157
	Prison experience	2	1.15	0.14	−0.52	0.81	0	0
Troubled family environment	All	4	1.92	0.65	0.44	0.86	0	0
	Raised by single mother	2	2.03	0.71	0.44	0.98	0	0
Violence	All	4	8.33	2.12	0.31	3.93	97.6*	3.253
	Violent offences	3	7.92	2.07	−0.17	4.3	99.1*	3.851
	Violent tendencies	2	4.90	1.59	0.89	2.3	0	0
	Instrumental violence	1	23.34	3.15	2.7	3.61	‐	‐
Being in a relationship		3	2.56	0.94	0.55	1.34	0	0
Economic condition	Protective	3	0.46	−0.77	−2.04	0.51	97.3*	1.196
	Risk	3	3.00	1.10	0.09	2.1	96.4*	0.718
Offence and/or contact with CJ system	All	3	2.86	1.08	−0.92	3.07	99.3*	3.068
	N. of convictions	3	2.94	1.05	−0.4	2.51	99.4*	1.643
	Age last known conviction	1	1.45	0.37	−0.1	0.85	‐	‐
Anxiety		2	2.34	0.85	−0.45	2.15	91.0*	0.803
Cognitive functioning	All	2	0.71	−0.34	−1.49	0.81	91.8*	0.635
	Executive functioning	2	0.80	−0.22	−1.66	1.22	92.3*	0.996
Criminal versatility		2	1.46	0.38	−0.53	1.29	95.6*	0.415
Depression		2	1.92	0.65	0.34	0.97	0	0
Living conditions/household (adulthood)	No children	2	2.69	0.99	0.31	1.68	68.8	0.186
	Number of siblings	1	1.15	0.51	−0.29	1.32	‐	‐
	Lives alone	1	1.39	0.33	−0.75	0.09	‐	‐
	Non‐intact household	1	0.84	0.16	−0.29	0.6	‐	‐
	Intact household	1	1.17	0.14	−0.21	0.49	‐	‐
	Lives with parents	1	1.67	−0.17	−0.73	0.39	‐	‐
Negative life events	All	2	2.46	0.90	0.52	1.28	0	0
	Traumatic physical occurrence	2	2.86	1.05	0.53	1.58	0	0
Offence type	Drug offences	2	5.26	1.66	−0.21	3.54	90.8*	1.674
	Property offences	2	2.86	1.05	−0.36	2.45	81.5*	0.865
	Weapon offences	1	2.34	3.35	3.15	3.56	‐	‐
	Traffic offences	1	1.16	2.40	2.19	2.60	‐	‐
	Online‐related offending	1	11.02	0.85	0.20	1.51	‐	‐
	Sexual offences	1	28.50	0.15	−0.05	0.36	‐	‐
Social environment		2	25.28	3.23	3.18	3.2	0	0
Internet use and technological capacity	Deviant online activities	1	1.84	0.61	0.15	1.06	‐	‐
	Nondeviant online activities	1	0.94	−0.06	−0.39	0.27	‐	‐
Motivation		1	17.64	2.87	2.44	3.31	‐	‐
Religious beliefs		1	0.41	−0.88	−1.14	−0.61	‐	‐

* Significant heterogeneity (*p* < 0.05).

We calculated a total of 50 associations with correlates at the category or subcategory level. As for the predictors, most associations (*n* = 32) comprised only one to two independent measures, suggesting that the evidence base for these relations is extremely weak. Fourteen associations included three or four independent effect sizes, while four associations (age, education, education—years of education, and low self‐control) included from six to ten independent measures.

#### 5.4.2 Factors

This section presents the results for each factor category and, when available, subcategory. When possible, we conducted meta‐analyses at the factor category and subcategory level (if the extracted effect sizes allowed to compute additional meta‐analyses). When both predictors and correlates are available, we report the results separately to avoid confusing factors measured before recruitment and factors measured at the same time. When meta‐analyses showed statistically significant heterogeneity, we conducted subgroup meta‐analyses moderating studies by type of organized criminal group (the results of moderator analyses are summarized in subsection Type of organized crime group as effect size moderator, and in Supporting Information Appendix E: Moderator analyses by type of organized criminal group). Each factor category also includes the description of the effect sizes not included in the meta‐analysis as well as the narrative synthesis obtained from the included qualitative studies.

##### Age

###### 5.4.2.1 Meta‐analysis

**Figure 3:**
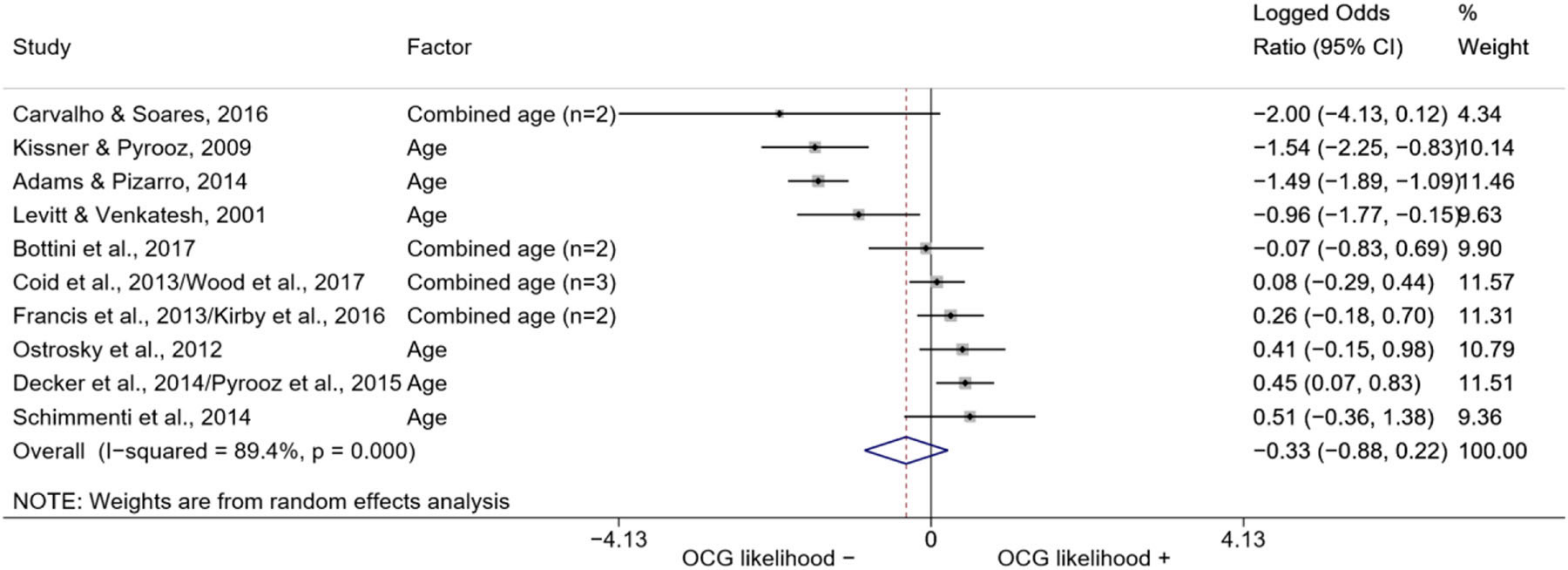
Age

Ten studies investigated the relation between age and organized crime membership, providing a total of 15 estimates (Adams & Pizarro, 2014; Bottini et al., 2017; Carvalho & Soares, 2016; Coid et al., 2013; Decker et al., 2014; Francis et al., 2013; Kirby et al., 2016; Kissner & Pyrooz, 2009; Levitt & Venkatesh, 2001; Ostrosky et al., 2012; Pyrooz et al., 2015; Schimmenti et al., 2014; Wood et al., 2017). Four studies reported multiple measures that were synthesized before their inclusion in the analysis (Bottini et al., 2017; Carvalho & Soares, 2016; Coid et al., 2013; Francis et al., 2013; Kirby et al., 2016; Wood et al., 2017). The overall pooled effect shows no statistically significant association between age and organized crime membership (log OR: −0.33, LL: −0.88, UL: 0.22) (Figure 3). Results also show significant variability among the measures (*I* ^2^: 89.4%, *p* < 0.001; *τ* ^2^ = 0.649).

###### 5.4.2.2 Qualitative studies

Eight qualitative studies considered the relation between age and involvement in organized criminal groups (Arlacchi, 1983; Arsovska, 2015; Cressey, 1969; Gordon, 2000; Hixon, 2010; Kemp et al., 2020; Radaelli et al., 2019; Van Koppen et al., 2010). The recruitment of younger individuals is frequently reported as a way of guaranteeing the intergenerational continuity of organized criminal groups (Arlacchi, 1983; Arsovska, 2015; Cressey, 1969; Hixon, 2010); However, late starters are not exceptional in organized crime recruitment, which can be related to opportunities coming from the social environment of adult individuals such as work and leisure activities, or to specific skills developed by individuals in late their life (Cressey, 1969; Gordon, 2000; Kemp et al., 2020; Kleemans & De Poot, 2008; Radaelli et al., 2019; Van Koppen et al., 2010).

##### Anxiety

###### 5.4.2.1 Meta‐analysis

**Figure 4:**
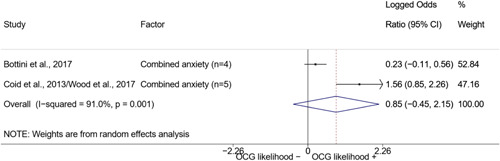
Anxiety

Two studies examined a total of nine correlates of anxiety and its relation with organized crime membership (Bottini et al., 2017; Coid et al., 2013; Wood et al., 2017). Bottini et al. (2017) investigated emotional and cognitive determinants of involvement into organized crime, reporting four measures, two for each comparison group (offenders in general, population sample), relating to state and trait anxiety. Similarly, Coid et al. (2013)/Wood et al. (2017) reported five estimates of anxiety (including fear of violent victimization) that were. Overall, the pooled effect indicates no statistically significant association between anxiety and organized crime membership (log OR: 0.85, LL: −0.45, UL: 2.15) (Figure 4), with high heterogeneity between studies (*I* ^2^: 91.0%, *p* = 0.001; *τ* ^2^ = 0.803).

##### Being in a relationship

###### 5.4.2.1 Meta‐analysis

**Figure 5:**
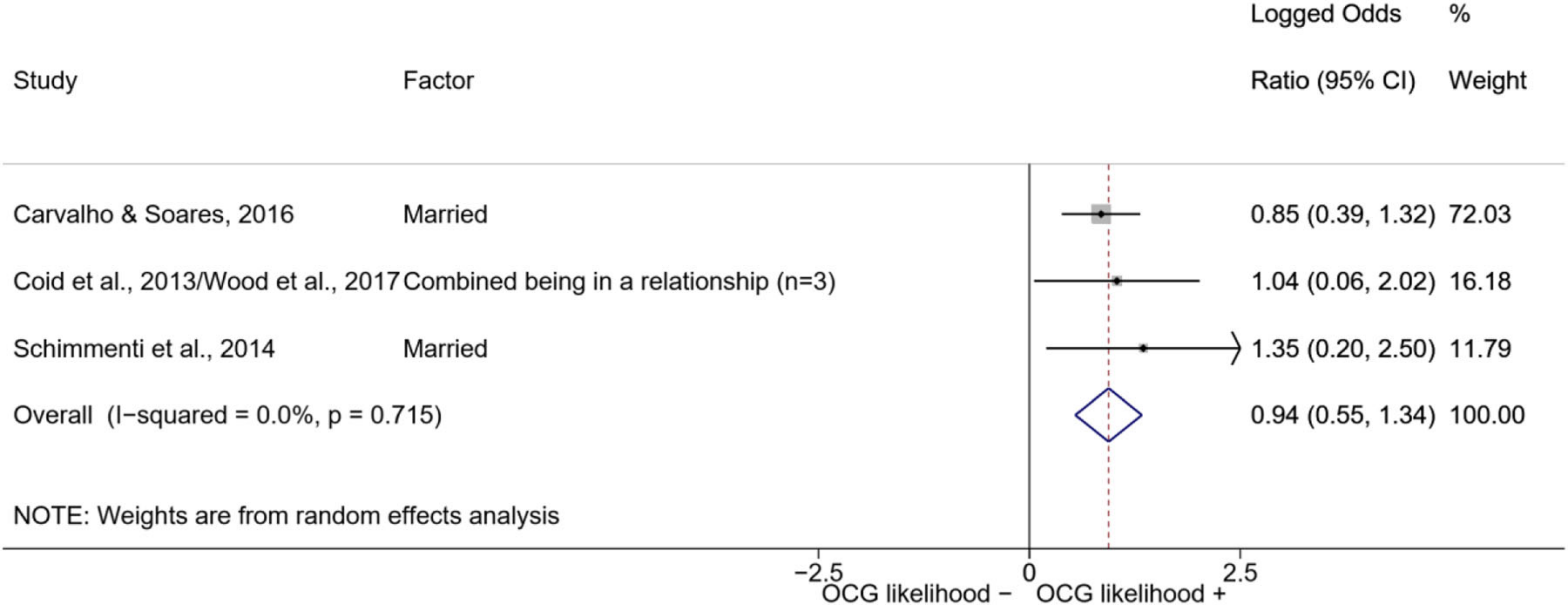
Being in a relationship

Three studies reported a total of five correlates of being in a relationship (Carvalho & Soares, 2016; Coid et al., 2013; Schimmenti et al., 2014; Wood et al., 2017). Carvalho and Soares (2016) and Schimmenti et al. (2014) reported each a binary variable of being married. Coid et al. (2013)/Wood et al. (2017), reported three correlates of being single which were reverse coded to represent being in a relationship and to have the same direction of the estimate relating to being married. The estimates were subsequently synthesized into a unique effect size before their inclusion in the analysis. Overall, the pooled effect shows a positive and significant association between being in a relationship and involvement into organized criminal groups (log OR: 0.94, LL: 0.55, UL: 1.34) (Figure 5). The result also shows that the measures are highly homogeneous (*I* ^2^: 0.0%, *p* = 0.715; *τ* ^2^ = 0.000).

#### 5.4.3 Cognitive functioning

##### Meta analyses

Two studies investigated the psychological sphere of organized crime members, contributing a total of 41 measures of cognitive functioning (Bottini et al., 2017; Ostrosky et al., 2012). Ostrosky et al. (2012) assessed the neuropsychological traits of individuals through the Executive Functions Battery (BANFE) test related to frontal and executive functions (p. 22). The effect size extracted from the total score of the BANFE test shows a negative and statistically significant association with organized crime membership. Bottini et al. (2017) reported forty estimates across two comparison groups, offenders in general and population sample.

**Figure 6:**
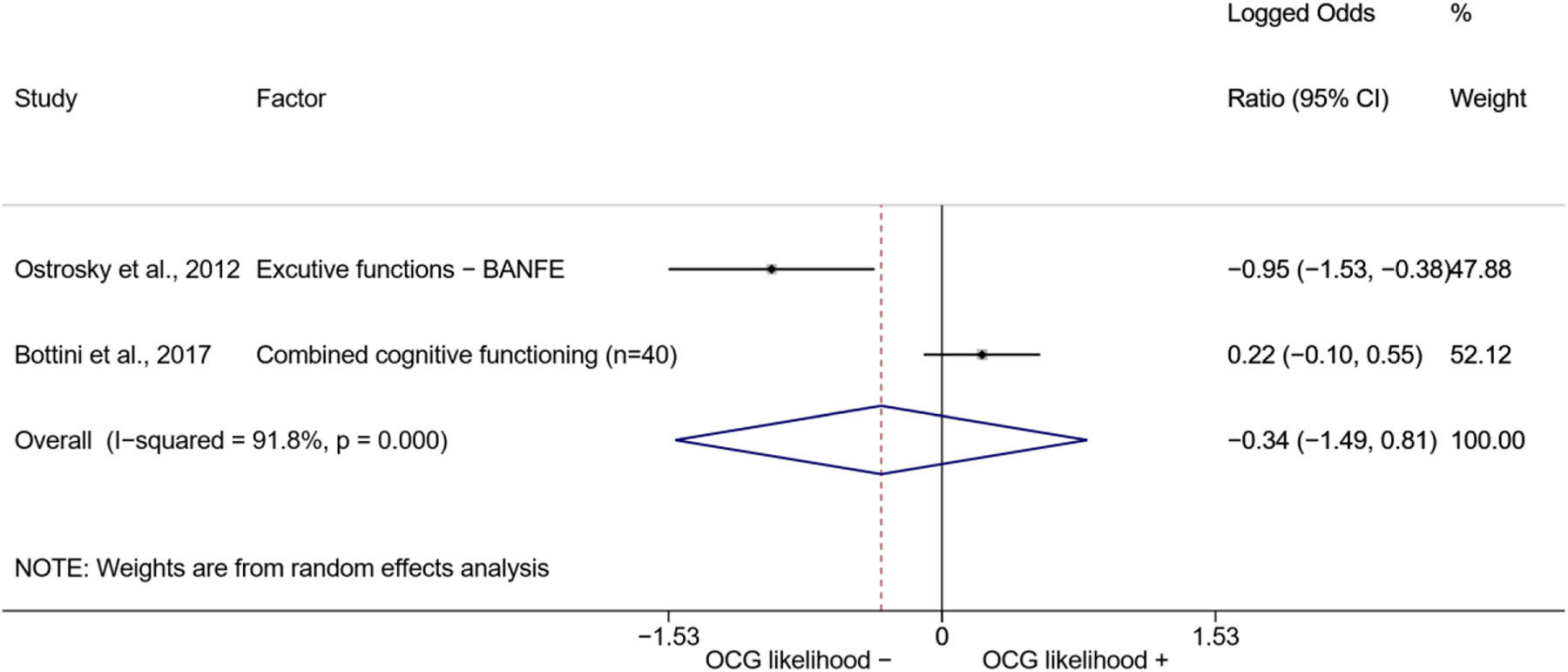
Cognitive functioning

The measures were grouped into six subcategories: attention, comprising reaction times and visual information processing measures; body representation, related to body awareness; emotion, referring to emotion recognition (assessing anger, disgust, fear, happiness, sadness); executive functions (including spatial working memory and multitasking test amongst others); memory, comprising paired associate learning and verbal memory; other, a residual category including global cognitive functioning and intelligence. To avoid issues related to lack of independence among intra‐study effect sizes, the estimates were synthesized. Overall, the pooled effect shows no statistically significant association between cognitive functioning and involvement into organized crime (log OR: −0.34, LL: −1.49, UL: 0.81), with high heterogeneity between studies (*I* ^2^: 91.8%, *p* < 0.001; *τ* ^2^ = 0.635) (Figure 6).

##### Executive function

**Figure 7:**
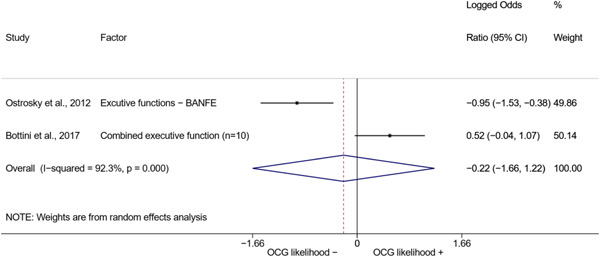
Executive function

Two studies (Bottini et al., 2017; Ostrosky et al., 2012) investigated the relation between individuals' executive functions and likelihood of organized crime membership, reporting a total of 11 measures. Ostrosky et al. (2012) provided a measure for the total score of the Executive Functions Battery (BANFE) test. Bottini et al. (2017) reported ten estimates of executive functions across two comparison groups, offenders in general and population sample. These measures were combined into a unique effect size. The overall pooled effect indicates negative but statistically nonsignificant relation between executive functions and organized crime membership (log OR: −0.22, LL: −1.66, UL: 1.22) (Figure 7). Results also show significant variability among the measures (*I* ^2^: 92.3%, *p* < 0.001; *τ* ^2^ = 0.996).

##### Criminal versatility

###### 5.4.3.1 Predictors—Meta‐analysis

**Figure 8:**
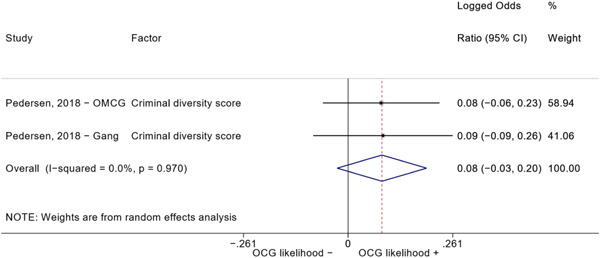
Criminal versatility—Predictors

Pedersen (2018) measured the association between criminal versatility and involvement into organized criminal groups reporting two continuous variables (i.e., a criminal diversity score): one for OMCG members and one for gang members (vs. offenders in general). The pooled estimate suggests a nonsignificant relation between prior criminal versatility and organized crime membership (log OR: 0.08, LL: −0.03, UL: 0.20) (Figure 8). The result of the meta‐analysis also shows that the measures are highly homogeneous (*I* ^2^: 0.0%, *p* = 0.970; *τ* ^2^ = 0.000).

###### 5.4.3.2 Correlates—Meta‐analysis

**Figure 9:**
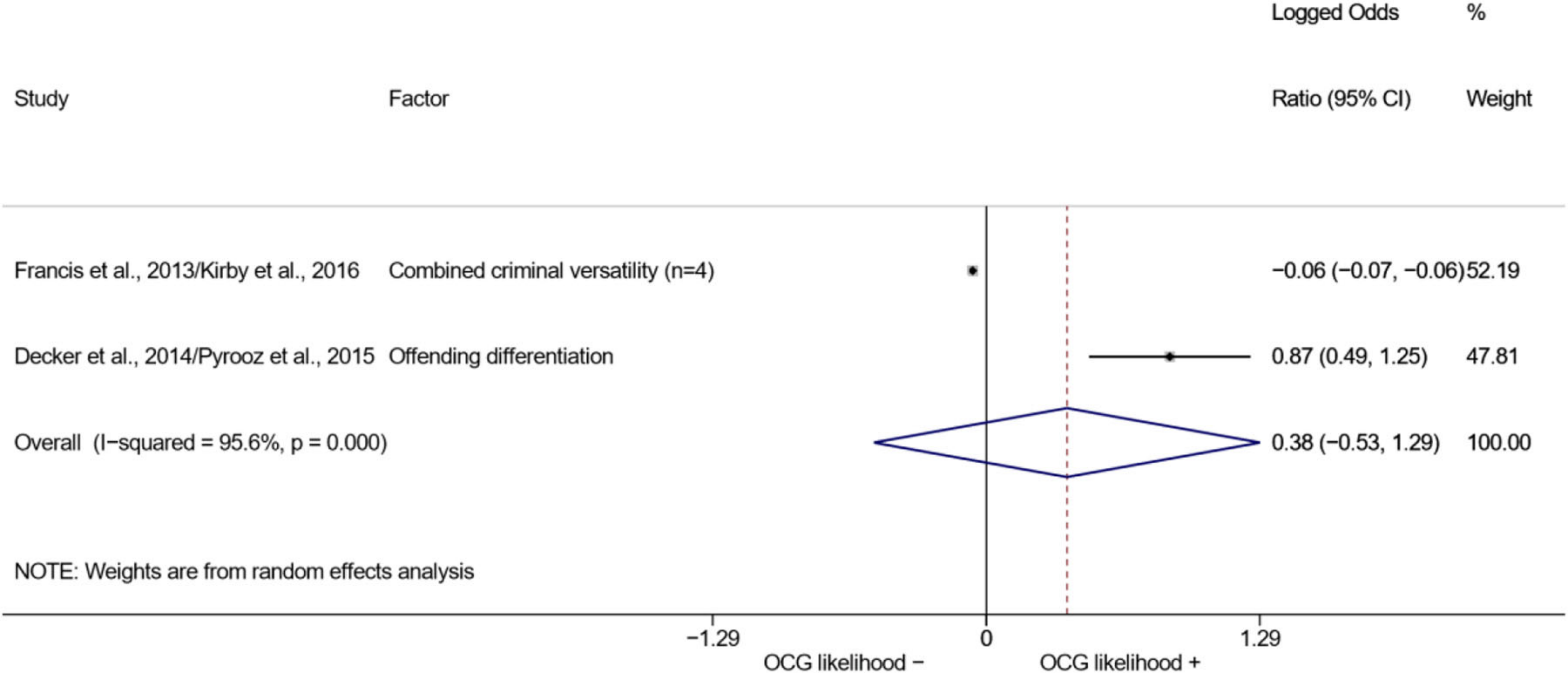
Criminal versatility—Correlates

Two studies reported five measures of criminal versatility (Decker et al., 2014; Francis et al., 2013; Kirby et al., 2016; Pyrooz et al., 2015). Francis et al. (2013)/Kirby et al. (2016) investigated criminal versatility reporting a total of four measures, two for each comparison group (serious offenders, offenders in general). The estimates were synthesized before their inclusion in the analysis. Overall, the meta‐analysis yields no statistically significant association between criminal versatility and organized crime membership (log OR: 0.38, LL: −0.53, UL: 1.29) (Figure 9), with high heterogeneity among studies (*I* ^2^: 95.6%, *p* < 0.001; *τ* ^2^ = 0.415).

##### Depression

**Figure 10:**
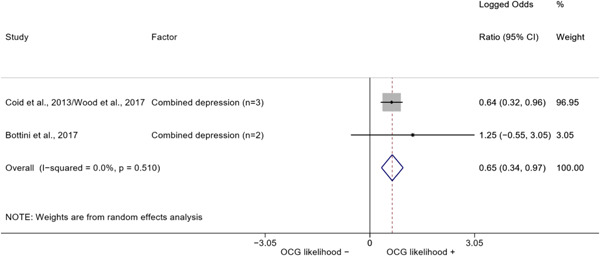
Depression

Depression was analyzed by two studies for a total of five measures (Bottini et al., 2017; Coid et al., 2013; Wood et al., 2017). Coid et al. (2013)/Wood et al. (2017) reported three estimates of depression, one for each comparison group (affiliates, population sample, violent men). Bottini et al. (2017) measured depression through the Beck Depression Inventory (BDI) and reported two estimates, one for the comparison group of offenders in general and one for population sample. The overall pooled effect shows a positive and significant association between suffering from depression and involvement into organized crime (log OR: 0.65, LL: 0.34, UL: 0.97) (Figure 10). Results also indicate that the correlates are highly homogeneous (*I* ^2^: 0.0%, *p* < 0.001; *τ* ^2^ = 0.000).

##### Economic condition

###### 5.4.3.1 Meta‐analysis

A total of five studies measured the association between economic condition and involvement into organized criminal groups (Carvalho & Soares, 2016; Coid et al., 2013; Kissner & Pyrooz, 2009; Klement, 2016; Levitt & Venkatesh, 2001; Wood et al., 2017). Three studies (Coid et al., 2013; Kissner & Pyrooz, 2009; Klement, 2016; Wood et al., 2017) reported a total of 14 estimates conceptualized as risk factors, whilst three studies (Carvalho & Soares, 2016; Klement, 2016; Levitt & Venkatesh, 2001) reported three correlates conceptualized as protective factors.

**Figure 11:**
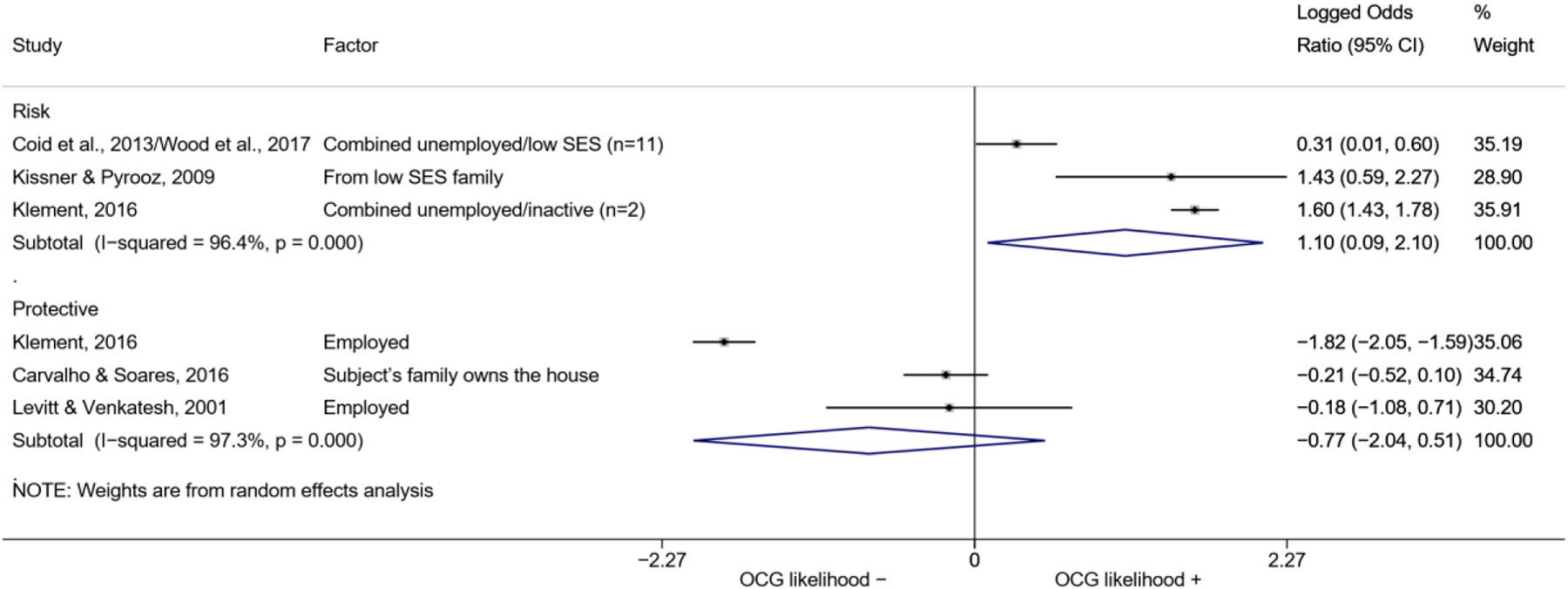
Economic condition

Regarding risk factors, Coid et al. (2013)/Wood et al. (2017) contributed 11 measures across three comparison groups (violent men, population sample, affiliates). The measures related to unemployment and/or low socioeconomic status (including coming from low social class family, being homeless, having serious money problems or made bankrupt) and were combined before their inclusion in the meta‐analysis to avoid issues of lack of independence. The pooled effect shows a positive and statistically significant association. The same result was found by Kissner and Pyrooz (2009) and Klement (2016). The former study included one estimate relating to coming from a family with low socioeconomic status, the latter included two measures addressing unemployment or being inactive (i.e., being outside the labor market). Overall, the result of the meta‐analysis indicates a positive and statistically significant association between being unemployed and/or having a low socioeconomic status and organized crime membership (log OR: 1.10, LL: 0.09, UL: 2.10) (Figure 11). Results also show a high variability amongst the measures (*I* ^2^: 96.4%, *p* < 0.001; *τ* ^2^ = 0.718).

Regarding protective factors, Carvalho and Soares (2016) reported an estimate of living in a house owned by the family (vs. coming from the favelas) while Levitt and Venkatesh (2001) and Klement (2016) included each a measure of being employed. The pooled effect indicates a negative but statistically nonsignificant association (log OR: −0.77, LL: −2.04, UL: 0.51) (Figure 11), with significant heterogeneity between the measures (*I* ^2^: 97.3%, *p* < 0.001; *τ* ^2^ = 1.196).

###### 5.4.3.2 Effect sizes not included in meta‐analysis

Sharpe (2002) assessed two predictors relating to economic conditions at the individual level: lack of legal economic opportunities and inability to find a good job. The overall pooled effect indicates a positive but statistically nonsignificant association between facing socioeconomic barriers and increased likelihood of becoming involved into organized criminal groups (log OR: 0.21, LL: −0.03, UL: 0.45), with no significant heterogeneity between the measures (*I* ^2^: 59.2%, *p* = 0.118; *τ* ^2^ = 0.021). The pooled effect was not included in the meta‐analysis because there were no other studies reporting predictors relating to economic conditions.

###### 5.4.3.3 Qualitative studies

Nine studies considered the relation between individuals' economic condition and involvement in organized criminal groups (Albini, 1971; Arsovska, 2015; Baird, 2018; Brancaccio, 2017; Brotherton & Barrios, 2004; Decker & Chapman, 2008; Hess, 1970/1973; Van San & Sikkens, 2017; Varese, 2011). Poverty can lead individuals to see the drug trafficking market as an acceptable way of earning money (Decker & Chapman, 2008; Van San & Sikkens, 2017); to join gangs to emancipate from low socioeconomic conditions (Baird, 2018; Brotherton & Barrios, 2004); and immigrants to join mafia organizations (Varese, 2011). A lower class background is also related to values and means that can make mafia organization as an acceptable way of surviving, receiving respect and career opportunities in difficult environments (Albini, 1971; Arsovska, 2015; Brancaccio, 2017; Hess, 1970/1973).

##### Education

###### 5.4.3.1 Meta‐analyses

**Figure 12:**
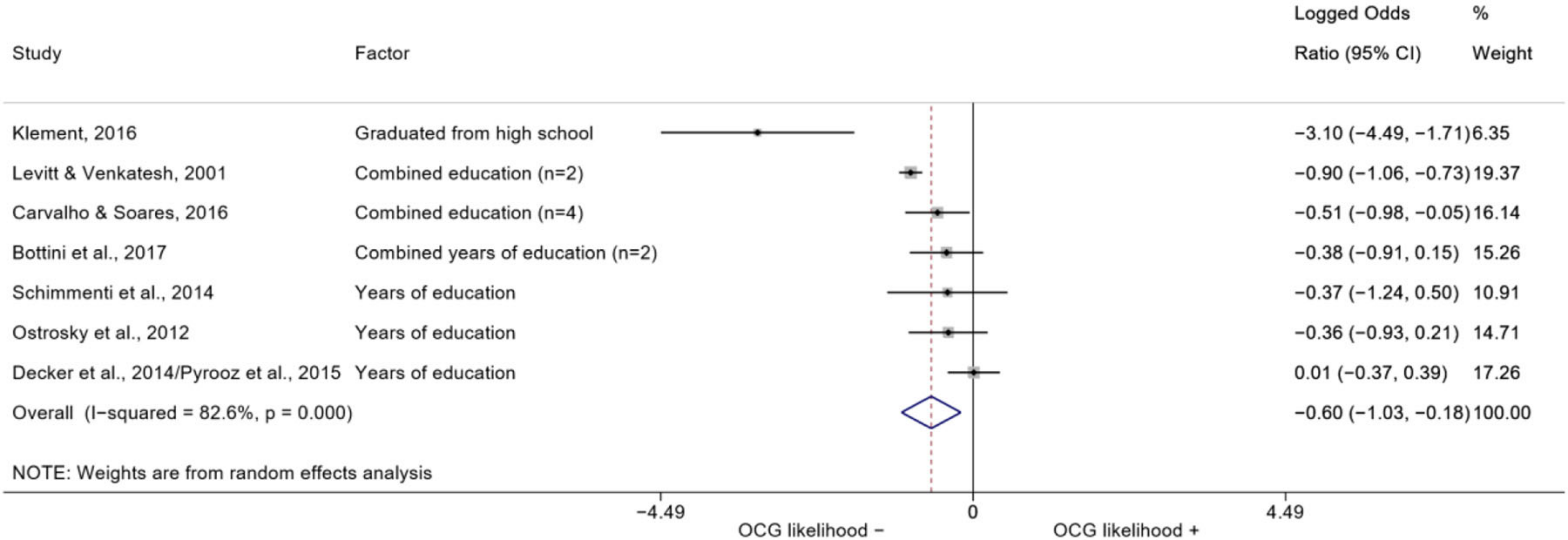
Education

Seven studies included a total of 12 correlates relating to individuals' level of education (Bottini et al., 2017; Carvalho & Soares, 2016; Decker et al., 2014; Klement, 2016; Levitt & Venkatesh, 2001; Ostrosky et al., 2012; Pyrooz et al., 2015; Schimmenti et al., 2014). Klement (2016) reported a categorical variable comprising three modalities: graduated from primary school, graduated from vocational/technical school, and graduated from upper secondary level school (i.e., high school). To make the comparison with other correlates possible, we opted to include the measure relating to the highest level of education. Carvalho and Soares (2016) reported four measures: two related to years of schooling, one to currently attending school, and one to being illiterate, which was reverse‐coded to represent being literate and to have the same direction of the other measures extracted. The measures were synthesized before their inclusion in the analysis. Overall, the pooled effect shows a negative and statistically significant association between higher levels of education and involvement into organized criminal groups (log OR: −0.60, LL: −1.03, UL: −0.18) (Figure 12), though there is significant heterogeneity between studies (*I* ^2^: 82.6%, *p* < 0.001; *τ* ^2^ = 0.235).

####### 5.4.3.1.1 High School

**Figure 13:**
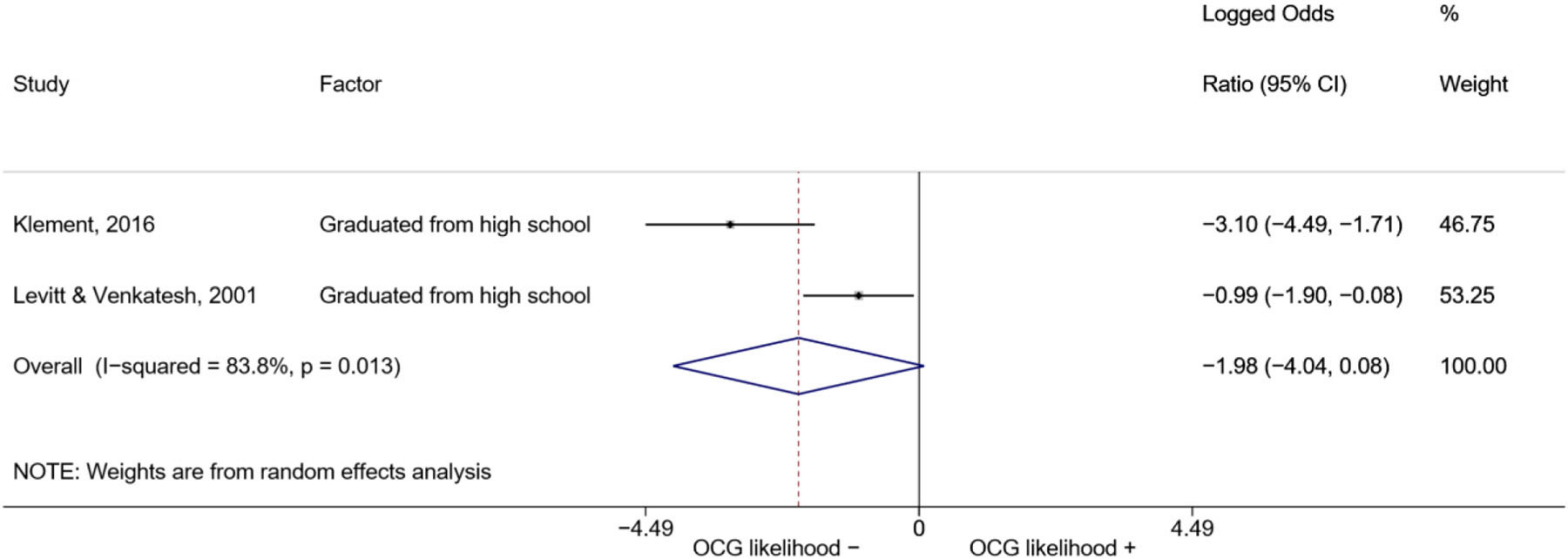
High school

Two studies provided a total of two correlates relating to being graduated from high school (Carvalho & Soares, 2016; Klement, 2016). The pooled effect indicates a nonsignificant relation with organized crime membership (log OR: −1.98, LL: −4.04, UL: 0.08) (Figure 13). Also, the result show that there is high heterogeneity between the studies (*I* ^2^: 83.8%, *p* = 0.013; *τ* ^2^ = 1.865).

####### 5.4.3.1.2 Years of education

**Figure 14:**
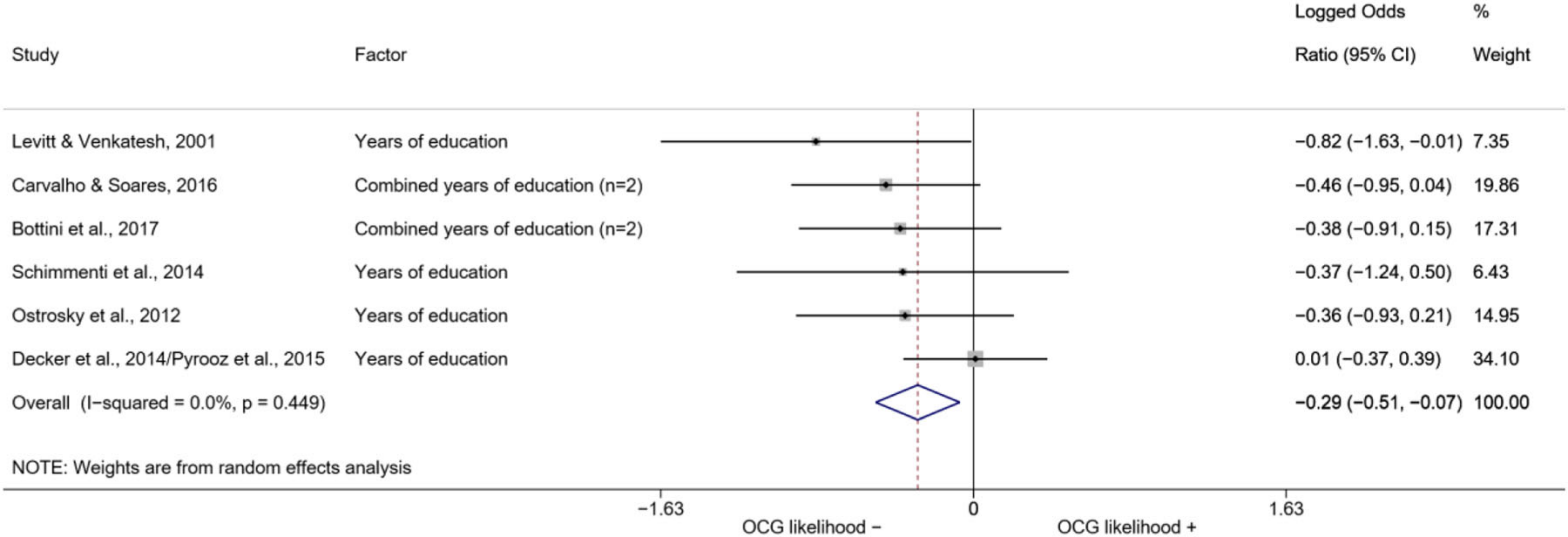
Years of education

Six studies analyzed individuals' level of education reporting a total of eight correlates relating to number of years of education. Of the included studies, two reported measures of number of education years of mafia members (Bottini et al., 2017; Schimmenti et al., 2014), two of gang members (Decker et al., 2014; Levitt & Venkatesh, 2001; Pyrooz et al., 2015), and two of members of drug‐trafficking organizations (Carvalho & Soares, 2016; Ostrosky et al., 2012). The overall pooled effect indicates a negative and statistically significant association with organized crime membership (log OR: −0.29, LL: −0.51, UL: −0.07) (Figure 14). The result also shows that the measures are highly homogeneous (*I* ^2^: 0.0%, *p* = 0.449; *τ* ^2^ = 0.000).

###### 5.4.3.2 Effect sizes not included in meta‐analysis

Decker et al. (2014)/Pyrooz et al. (2015) reported one measure of parental education (log OR: −0.04, LL: −0.42, UL: 0.34) which was not included in the meta‐analysis as no other studies reported a conceptually comparable correlate.

###### 5.4.3.3 Qualitative studies

Two qualitative study mentioned the low level of education of individuals becoming involved in organized criminal groups (Spapens & Moors, 2020; Zhang & Chin, 2002). A study of Chinese human smuggling organizations found that most of the subjects examined had a high school education or less (Zhang & Chin, 2002). A study on the intergenerational transmission of delinquent behavior in organized crime families highlighted the frequency of low levels of education and dropping out of school in members of organized criminal groups (Spapens & Moors, 2020).

##### Ethnicity (predictors)

###### 5.4.3.1 Meta‐analysis

Eight studies examined the relationship between ethnicity and involvement into organized crime groups, providing a total of 24 estimates (Adams & Pizarro, 2014; Carvalho & Soares, 2016; Coid et al., 2013; Danner & Silverman, 1986; Decker et al., 2014; Francis et al., 2013; Kirby et al., 2016; Kissner & Pyrooz, 2009; Pyrooz et al., 2015; Sharpe, 2002; Wood et al., 2017). The analysis was performed by ethnic groups, namely: Black, White, and any non‐White. Three studies reported multiple measures for ethnicity and organized crime membership (Carvalho & Soares, 2016; Coid et al., 2013; Francis et al., 2013; Kirby et al., 2016; Wood et al., 2017). For each study, these measures were synthesized by ethnic group before inclusion in the final meta‐analysis.

Regarding being Black, the meta‐analysis included six different studies estimates. The overall pooled estimate suggests positive but not statically significant association between being Black and organized crime membership (log OR: 0.53, LL: −0.01, UL: 1.08), with high heterogeneity amongst the measures (*I* ^2^: 93.5%, *p* < 0.001; *τ* ^2^ = 0.353).

All measures showed a negative association between being White and organized crime membership, with only one study finding a nonsignificant relationship (Sharpe, 2002). The pooled estimate shows a negative association between being White and organized crime membership (log OR: −0.67, LL: −1.11, UL: −0.23). Overall, being White decreases the likelihood of becoming involved into organized, with significant heterogeneity across studies (*I* ^2^: 75.7%, *p* = 0.006; *τ* ^2^ = 0.140).

**Figure 15:**
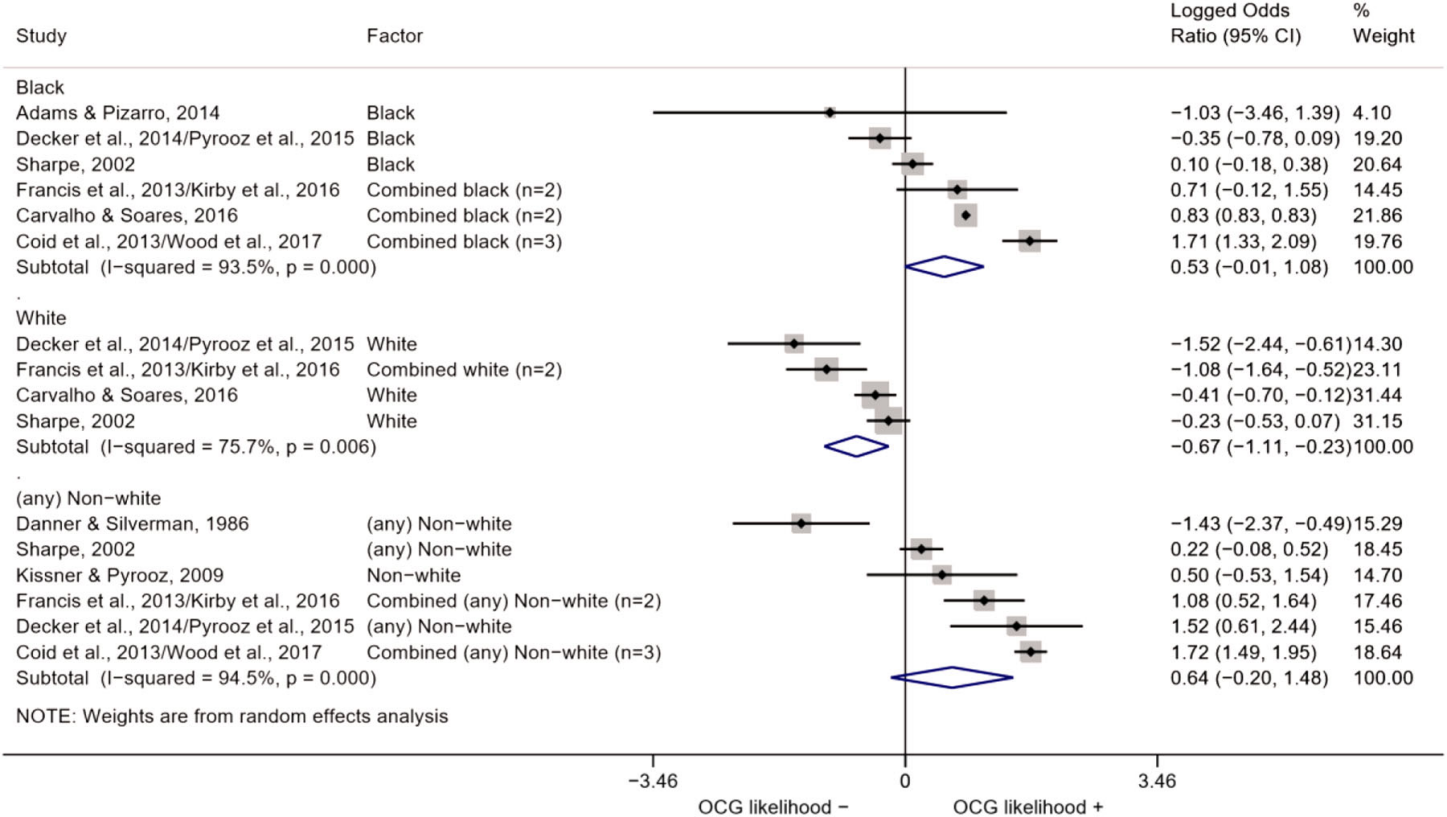
Ethnicity

Lastly, six studies investigated the relation between (any) non‐White race and involvement into organized crime. Overall, the meta‐analysis yields no statistically significant association between being of (any) non‐White race and belonging to an organized crime group (log OR: 0.64, LL: −0.20, UL: 1.48) (Figure 15). Also, there is a high heterogeneity between the studies (*I* ^2^: 94.5%, *p* < 0.001; *τ* ^2^ = 0.972).

###### 5.4.3.2 Effect size not included in meta‐analysis

One study (Carvalho & Soares, 2016) reported one measure of mixed‐race (log OR: −0.38, LL: −0.65, UL: −0.10) which was not included in the analysis as no other studies reported a conceptually comparable predictor.

###### 5.4.3.3 Qualitative studies

Fifteen qualitative studies examined the relation between ethnicity and involvement in organized criminal groups (Arsovska, 2015; Brotherton & Barrios, 2004; Chalas & Grekul, 2017; Cressey, 1969; Decker & Chapman, 2008; Densley, 2012; Gordon, 2000; Hixon, 2010; Knox et al., 1997; Leukfeldt et al., 2019; Paoli, 2003; Pedersen, Unpublished; Zhang & Chin, 2002). Eight studies highlighted the role of ethnic homogeneity in developing mutual trust, which is a key element in organized criminal groups against the risk of detection and arrest (Arsovska, 2015; Cressey, 1969; Decker & Chapman, 2008; Gordon, 2000; Leukfeldt et al., 2019; Paoli, 2003; Pedersen, Unpublished; Zhang & Chin, 2002). By contrast, three studies examined how ethnic marginality can also lead individuals to become involved in organized criminal groups to overcome their ethnic minority status (Arsovska, 2015; Chalas & Grekul, 2017; Gordon, 2000). Finally, four studies highlighted the relationship between a specific ethnic group and the involvement in organized criminal groups in specific contexts, including being White in the case of White supremacist gangs (Hixon, 2010); any non‐White ethnicity in the case of Canadian (Gordon, 2000) and US (Knox et al., 1997) gangs; and Black ethnicity in London's gangs (Densley, 2012).

###### 5.4.3.4 Foreign born (predictors)

 

###### Meta‐analysis

**Figure 16:**
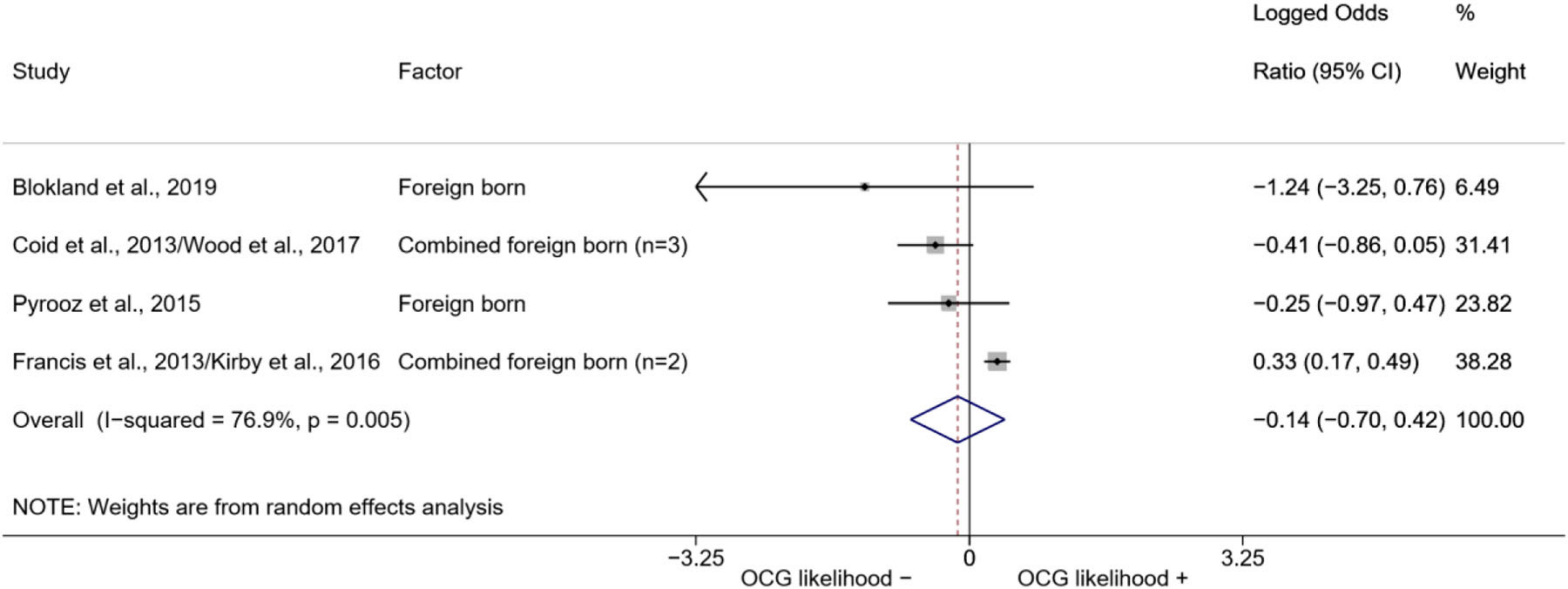
Foreign born

Four studies provided a total of seven estimates for having foreign origins (Blokland et al., 2019; Coid et al., 2013; Francis et al., 2013; Kirby et al., 2016; Pyrooz et al., 2015; Wood et al., 2017).^13^ Two studies reported multiple measures which were synthesized before inclusion in the analysis (Coid et al., 2013; Francis et al., 2013; Kirby et al., 2016; Wood et al., 2017). The overall pooled estimate indicates no statistically significant association with organized crime membership (log OR: −0.14, LL: −0.70, UL: 0.42) (Figure 16). Results also show significant variability amongst the effects (*I* ^2^: 76.9%, *p* = 0.005; *τ* ^2^ = 0.206).

##### Internet use and technological capacity

###### 5.4.3.1 Nondeviant online activities

Pyrooz et al. (2015) investigated Internet use and technological capacity of gang members (vs. population sample), reporting seven correlates relating to internet use and technological capacity (including internet use prevalence and frequency, online shopping, YouTube viewing, and use of social networks). The pooled estimate shows a nonsignificant association with organized crime membership (log OR: −0.06, LL: −0.39, UL: 0.27), with moderate and significant heterogeneity among the measures (*I* ^2^: 64.7%, *p* = 0.009; τ^2^ = 0.124).

###### 5.4.3.2 Deviant online activities

Pyrooz et al. (2015) reported two correlates for deviant online activities, as illegal downloads and upload deviant videos. The pooled effect indicates that engaging in deviant online activities is positively associated with involvement into organized criminal groups (log OR: 0.61, LL: 0.15, UL: 1.06). The result also shows that the measures are highly homogeneous (*I* ^2^: 0.0%, *p* = 0.353; *τ* ^2^ = 0.000).

##### Legitimate job/skills

###### 5.4.3.1 Qualitative studies

The qualitative studies emphasized organized crime groups' preference for individuals who have developed strategic skills/expertise or who are specialized in specific job sectors thanks to their legitimate life and career only emerged from qualitative literature. These factors were reported by thirteen studies (Ancrum & Treadwell, 2017; Cressey, 1969; Decker & Chapman, 2008; Gambetta, 1993; Hixon, 2010; Kleemans & De Poot, 2008; Kleemans & Van de Bunt, 2008; Leukfeldt et al., 2019; May & Bhardwa, 2018; Radaelli et al., 2019; Van Koppen & De Poot, 2013; Van Koppen, 2013; Zhang & Chin, 2002).

Ten studies examined the attractiveness of individuals with job positions strategic for organized crime groups (Ancrum & Treadwell, 2017; Decker & Chapman, 2008; Kleemans & De Poot, 2008; Kleemans & Van de Bunt, 2008; Leukfeldt et al., 2019; May & Bhardwa, 2018; Van Koppen & De Poot, 2013; Van Koppen, 2013; Zhang & Chin, 2002). The most frequently mentioned are individuals having autonomous occupation or a certain degree of independence at work (Kleemans & De Poot, 2008; Van Koppen & De Poot, 2013; Van Koppen, 2013; Zhang & Chin, 2002); individuals involved in the transport and logistic industry, especially for what concerns smuggling activities (Decker & Chapman, 2008; Kleemans & De Poot, 2008; Kleemans & Van de Bunt, 2008; Van Koppen, 2013); and individuals who can act as enablers such as bank employees, business men, lawyers, financial and legal consultants, tax experts, or individuals with political connections (Kleemans & De Poot, 2008; Kleemans & Van de Bunt, 2008; Leukfeldt et al., 2019; May & Bhardwa, 2018). Occasionally, isolated cases of other job sectors also emerge depending on the specific needs of the criminal organization under examination, from legal weapons dealers (Kleemans & De Poot, 2008); to agriculture producers who can convert their legal plantations in drug cultivation (Ancrum & Treadwell, 2017); up to university professors who showed predisposition toward misconduct and who can favor certain students protected by organized crime (Radaelli et al., 2019).

Seven studies highlighted how organized criminal groups can be attracted by individuals who have developed strategic and specialized skills during their life and career in the legal economy, which can be useful for specific illegal tasks and business (Ancrum & Treadwell, 2017; Cressey, 1969; Gambetta, 1993; Hixon, 2010; Kleemans & De Poot, 2008; Kleemans & Van de Bunt, 2008; Leukfeldt et al., 2019). Some examples are White supremacist gangs encouraging the recruitment of individuals with military experience who have trained in obedience and conformity, are familiar with weapons and violence, and can teach the military skills to the other gang members (Hixon, 2010); hackers for online‐related crimes, who can provide specific technical services (Leukfeldt et al., 2019); and individuals skilled in handling explosives, chemists for the drug industry, doctors, and priests (Gambetta, 1993). In some cases, the criminal organization itself makes long‐term investment on certain individuals by financing their education in strategic sectors, so that they would become responsible for modern large‐scale business operations within the group (Cressey, 1969).

##### Living conditions/household (adulthood)

Three studies contributed to the relation between gang members' household and living condition during adulthood and organized crime membership, reporting a total of 15 correlates (Carvalho & Soares, 2016; Levitt & Venkatesh, 2001; Wood et al., 2017). To avoid mixing conceptually different factors, we did not conduct a meta‐analysis of this category, but we opted to present results by type of subcategory.

###### 5.4.3.1 Intact household

Wood et al. (2017) provided two correlates being in contact with own children, conceptualized as intact household during adulthood. The pooled effect suggests a nonsignificant association with organized crime membership (log OR: 0.14, LL: −0.21, UL: 0.49), though the measures were highly homogeneous (*I* ^2^: 0.0%, *p* = 0.604; *τ* ^2^ = 0.000).

###### 5.4.3.2 Non‐intact household

Wood et al. (2017) reported two correlates of not being in contact with own children or children in authority care, conceptualized as non‐intact household during adulthood. The pooled effect indicates a nonsignificant association with organized crime membership (log OR: 0.16, LL: −0.29, UL: 0.60), though the measures were highly homogeneous (*I* ^2^: 0.0%, *p* = 0.644; *τ* ^2^ = 0.000).

###### 5.4.3.3 Lives alone

Wood et al. (2017) assessed the relation between living alone and organized crime membership, providing a total of two correlates. The pooled effect shows nonsignificant relation (log OR: 0.33, LL: −0.75, UL: 0.09), with no heterogeneity among the measures (*I* ^2^: 0.0%, *p* = 0.410; τ^2^ = 0.000).

###### 5.4.3.4 Lives with parents

Wood et al. (2017) reported two correlates of living with parents and the synthesized effect shows a negative but nonsignificant association (log OR: −0.17, LL: −0.73, UL: 0.39), with no significant heterogeneity among the measures (*I* ^2^: 62.5%, *p* = 0.103; *τ* ^2^ = 0.101).

###### 5.4.3.5 No children

Wood et al. (2017) investigated the relation between having no children and involvement into organized crime groups, providing a total of two correlates. The pooled effect shows nonsignificant relation (log OR: −0.26, LL: −0.58, UL: 0.06), with no heterogeneity among the measures (0.0%, *p* = 0.768; *τ* ^2^ = 0.000).

###### 5.4.3.6 Number of siblings

####### 5.4.3.6.1 Meta‐analysis

**Figure 17:**
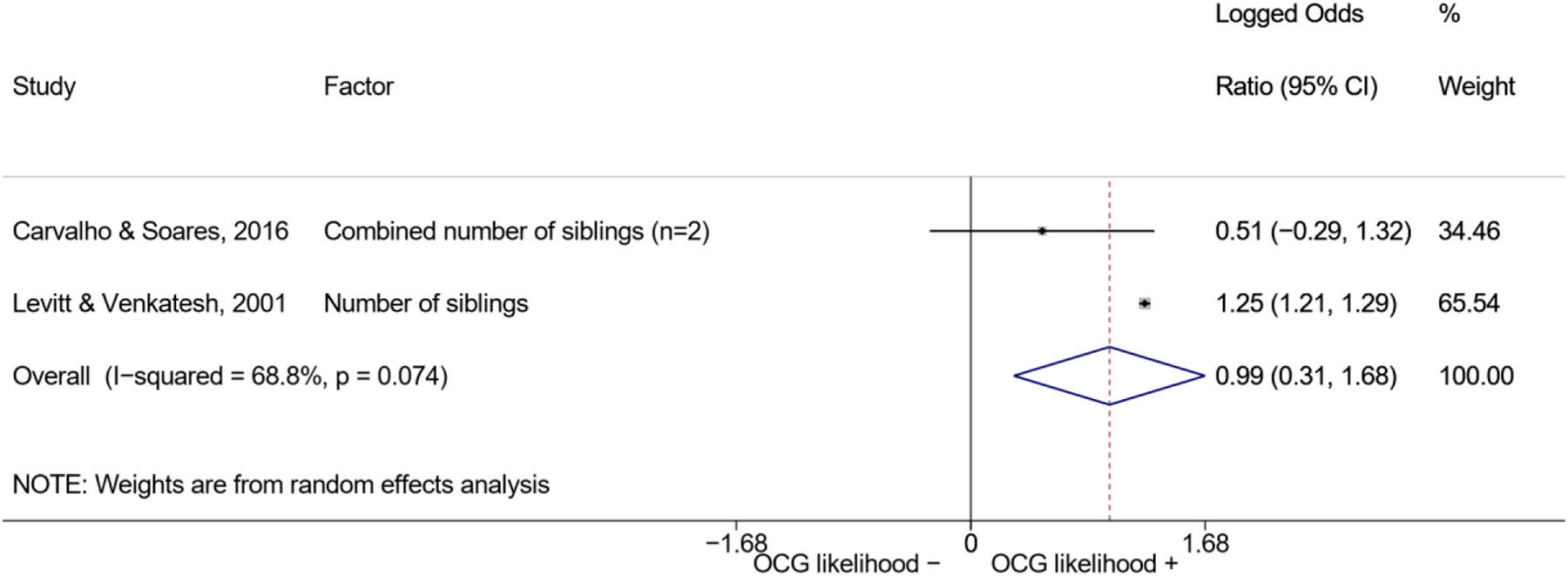
Number of siblings

Two studies (Carvalho & Soares, 2016; Levitt & Venkatesh, 2001) investigated the relation between number of siblings and involvement into organized crimes, reporting a total of three correlates. The overall pooled effect indicates a positive and statistically significant relation with organized crime membership (log OR: 0.99, LL: 0.31, UL: 1.68) (Figure 17), with no significant heterogeneity between studies measures (68.8%, *p* = 0.074; *τ* ^2^ = 0.186).

##### Low self‐control

###### 5.4.3.1 Meta‐analyses

**Figure 18:**
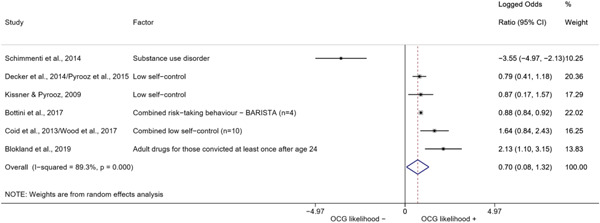
Low self‐control

Six studies provided 18 estimates of low self‐control (Blokland et al., 2019; Bottini et al., 2017; Coid et al., 2013; Decker et al., 2014; Kissner & Pyrooz, 2009; Pyrooz et al., 2015; Schimmenti et al., 2014; Wood et al., 2017). Schimmenti et al. (2014) reported substance use disorder as a binary variable, Decker et al. (2014)/Pyrooz et al. (2015) and Kissner and Pyrooz (2009) reported a correlate of low self‐control. Bottini et al. (2017) measured risk‐taking behavior through the Body and Balloon Analogue Risk Task (BARISTA) test reporting a total of four measures, two for each comparison group (offenders in general, population sample). These measures were first combined by comparison group and then further synthesized into a unique effect before their inclusion in the analysis. Coid et al. (2013)/Wood et al. (2017) included ten estimates of drug use and addiction problems across two comparison groups (affiliates, violent men) and conceptualized as low self‐control, including: drug dependence, alcohol dependence, pathological gambling, problem pornography/porn addiction. The measures were synthesized before their inclusion in the analysis. Lastly, Blokland et al. (2019) measured drug use and addiction problems reporting one correlate of drug offending for individuals convicted at least once after age 24, a cut‐off threshold for involvement into organized criminal groups (see Blokland et al., 2019, p. 15). Overall, the pooled effect indicates a positive and statistically significant relation between measures of low self‐control and involvement into organized criminal groups (log OR: 0.70, LL: 0.08, UL: 1.32) (Figure 18). Result of the meta‐analysis also shows that there is high and significant heterogeneity between studies (*I* ^2^: 89.3%, *p* < 0.001; *τ* ^2^ = 0.458).

####### 5.4.3.1.1 Drug use and addiction problems

**Figure 19:**
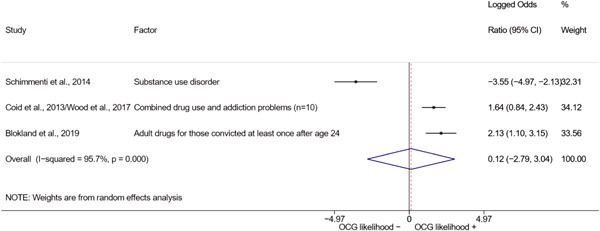
Drug use and addiction problems

Three studies provided 12 estimates of drug use and addiction problems (Blokland et al., 2019; Coid et al., 2013; Schimmenti et al., 2014; Wood et al., 2017). Blokland et al. (2019) measured drug use and addiction problems reporting one correlate of drug offending for individuals convicted at least once after age 24 (i.e., after the onset of organized crime membership). Schimmenti et al. (2014) reported substance use disorder as a binary variable. Coid et al. (2013)/Wood et al. (2017) included ten estimates of drug use and addiction problems across two comparison groups (affiliates, violent men), including: drug dependence, alcohol dependence, pathological gambling, problem pornography/porn addiction. These measures were first combined by comparison group and then further synthesized into a unique effect before their inclusion in the analysis. Overall, the pooled effect indicates a statistically nonsignificant relation between measures of drug use and addiction problems and involvement into organized criminal groups (log OR: 0.12, LL: −2.79, UL: 3.04) (Figure 19), with high heterogeneity amongst the effects (*I* ^2^: 95.7%, *p* < 0.001; *τ* ^2^ = 6.325).

####### 5.4.3.1.2 Low self‐control (subcategory)

**Figure 20:**
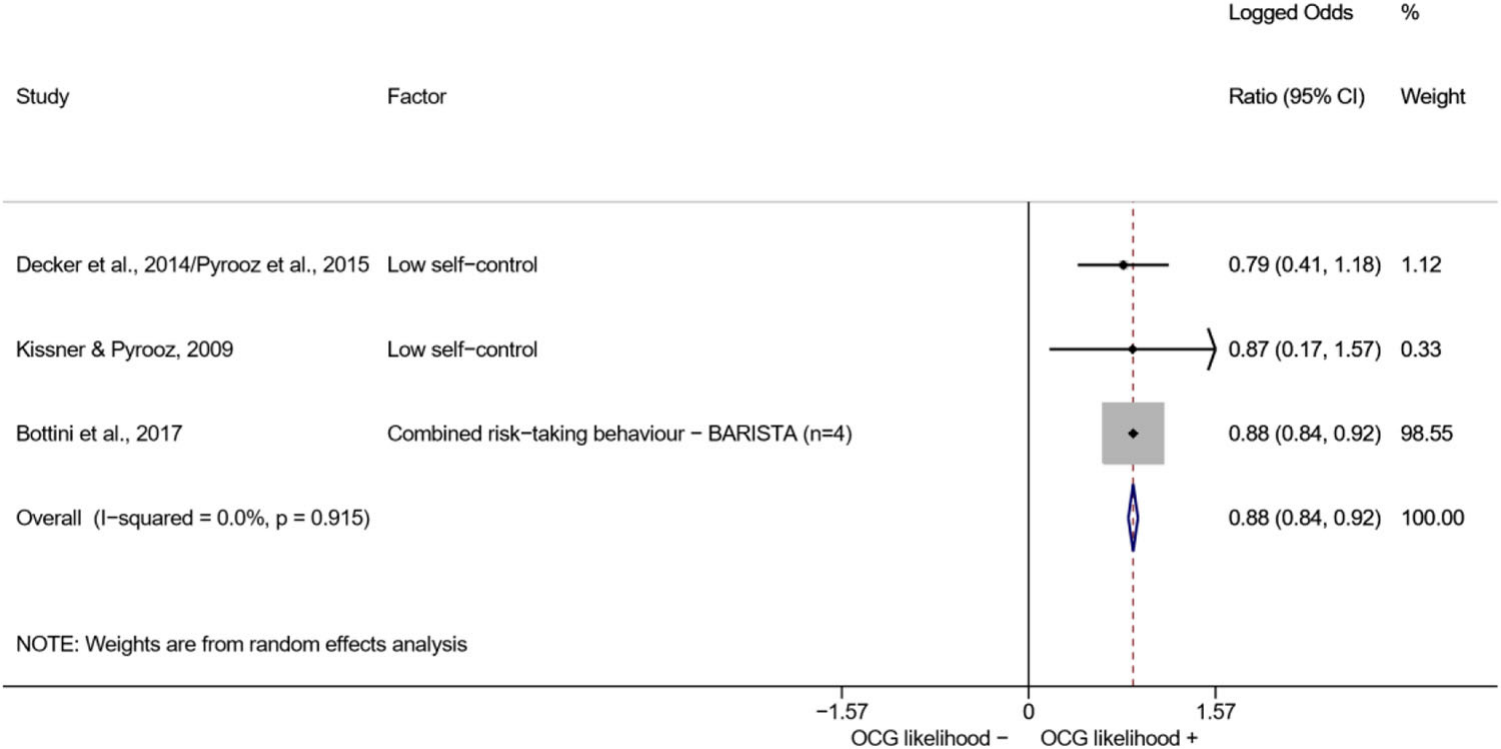
Low self‐control (subcategory)

Three studies investigated low self‐control providing six correlates which were used to analyze this factor at the subcategory level (Bottini et al., 2017; Decker et al., 2014; Kissner & Pyrooz, 2009; Pyrooz et al., 2015). The overall pooled estimate shows a positive and statistically significant association with involvement into organized crime groups (log OR: 0.88, LL: 0.84, UL: 0.92) (Figure 20), and the measures are highly homogeneous (*I* ^2^: 0.0%, *p* = 0.915; *τ* ^2^ = 0.000).

###### 5.4.3.2 Effect sizes not included in meta‐analysis

Of the included studies, only one study reported a predictor for low‐self‐control. Blokland and colleagues (2019) examined drug use during juvenile years/early adulthood for OMCG members (vs. offenders in general) convicted at least once before age 25 (a cut‐off point for organized crime membership). The estimate shows a positive but nonsignificant association between low self‐control and involvement into organized criminal groups (log OR: 1.56, LL: −0.47, UL: 3.60).

##### Motivation

###### 5.4.3.1 Effect sizes not included in meta‐analysis

Decker et al. (2014)/Pyrooz et al. (2015) measured the association of the importance of gang to respondents (gang vs. population sample), conceptualized as motivation, and organized crime membership. The effect suggests that the individual's motivation is a positive and statistically significant factor (log OR: 2.87, LL: 2.44, UL: 3.31).

###### 5.4.3.2 Qualitative studies

The personal motivation leading individuals to join organized criminal groups was frequently examined by qualitative literature, and in particular by nineteen studies (Albini, 1971; Ancrum & Treadwell, 2017; Arlacchi, 1983; Arsovska, 2015; Baird, 2018; Brancaccio, 2017; Brotherton & Barrios, 2004; Chalas & Grekul, 2017; Cressey, 1969; Decker & Chapman, 2008; Gambetta, 1993; Gordon, 2000; Hess, 1970/1973; Hixon, 2010; Kemp et al., 2020; Kleemans & De Poot, 2008; May & Bhardwa, 2018; Paoli, 2003; Pedersen, Unpublished; Van Koppen, 2013).

The sense of social cohesion provided by criminal groups where individuals share values and belong to the same subculture is a key factor leading individuals to join organized criminal groups (Albini, 1971; Arsovska, 2015; Brancaccio, 2017; Brotherton & Barrios, 2004; Chalas & Grekul, 2017; Cressey, 1969; Gambetta, 1993; Hess, 1970/1973; Hixon, 2010; Paoli, 2003; Pedersen, Unpublished). Brotherhood, loyalty, mutual protection, and shared values create a strong sense of belonging to organized crime (Arsovska, 2015; Brancaccio, 2017; Brotherton & Barrios, 2004; Chalas & Grekul, 2017; Paoli, 2003; Pedersen, Unpublished). Secrecy and exclusiveness create family‐like environments in mafia organizations (Cressey, 1969; Hixon, 2010; Paoli, 2003). The sense of belonging is often reinforced by initiation rituals and ceremonies especially in mafia organizations (Albini, 1971; Gambetta, 1993; Hess, 1970/1973; Hixon, 2010; Paoli, 2003).

The perspective of financial gain ensuring high income is highly attractive for individuals coming from low socioeconomic environment, allowing a lifestyle that would otherwise have been unavailable to them (Ancrum & Treadwell, 2017; Gordon, 2000), facing financial setback and debts (Kemp et al., 2020; Kleemans & De Poot, 2008; May & Bhardwa, 2018; Van Koppen, 2013), having specific needs such as drug addiction (Van Koppen, 2013), or simply animated by the desire of money and material goods (Chalas & Grekul, 2017; May & Bhardwa, 2018; Pedersen, Unpublished). Also, middle‐class individuals may be attracted by the opportunity for enrichment and social mobility offered by joining the mafias (Arlacchi, 1983).

The ambition of being successful in life and displaying social status are also recurrent motivations for joining organized criminal groups (Arlacchi, 1983; Baird, 2018; Brancaccio, 2017; Chalas & Grekul, 2017; Cressey, 1969; Decker & Chapman, 2008; Kemp et al., 2020; Paoli, 2003; Pedersen, Unpublished; Van Koppen, 2013). The exciting lifestyle that comes with money, power, respect, devoted friends, adventure, and party attracts individuals into drug‐trafficking organizations (Decker & Chapman, 2008), gangs (Baird, 2018; Chalas & Grekul, 2017; Pedersen, Unpublished), mafias (Arlacchi, 1983; Brancaccio, 2017; Cressey, 1969), and other organized crime groups (Van Koppen, 2013). Because of this, successful organized crime role models play a key role in fascinating individuals and bring them into organized criminal groups (Baird, 2018; Chalas & Grekul, 2017; Cressey, 1969; Hess, 1970/1973; Kemp et al., 2020; Pedersen, Unpublished).

##### Negative life events

###### 5.4.3.1 Meta‐analyses

**Figure 21:**
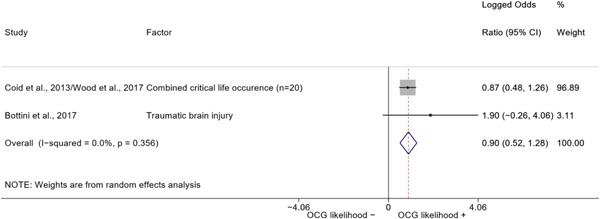
Negative life events

Two included studies contributed to the relation between negative life events and organized crime membership, reporting a total of 21 correlates (Bottini et al., 2017; Coid et al., 2013; Wood et al., 2017).^14^ Coid et al. (2013)/Wood et al. (2017) reported 20 binary measures across three comparison groups (affiliates, violent men, population sample). These measures, addressing traumatic and/or physical life occurrences and conceptualized as negative life events, referred to: victimization (including domestic violence from a partner, violent victimization, and being victim of stalking), critical life occurrences (comprising suicide attempts, being injured as a result of physical attack, being sacked or made redundant, serious/life threatening injury, deliberate self‐harm, marital separation/steady relation breakdown, and death of husband/wife, partner, or child). The second included study reported one measure of traumatic brain injury (Bottini et al., 2017). The overall effect indicates that experiencing negative life events increases is positively associated with involvement into organized criminal groups (log OR: 0.90, LL: 0.52, UL: 1.28) (Figure 21). The result also shows that the measures are highly homogeneous (*I* ^2^: 0.0%, *p* = 0.356; *τ* ^2^ = 0.000).

####### 5.4.3.1.1 Traumatic physical occurrence

Two studies analyzed traumatic physical occurrence providing a total of 8 correlates (Bottini et al., 2017; Coid et al., 2013; Wood et al., 2017). Bottini et al. (2017) reported one estimate of traumatic brain injury. Coid et al. (2013)/Wood et al. (2017) reported seven measures across three comparison groups (affiliates, violent men, population sample) and relating to deliberate self‐harm, serious/life threatening injury, and suicide attempt. The correlates were first synthesized into a unique effect size before their inclusion in the analysis.

**Figure 22:**
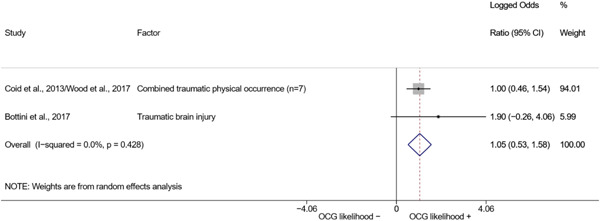
Traumatic physical occurrence

The overall pooled effect shows a positive and statistically significant association with involvement into organized criminal groups (log OR: 1.05, LL: 0.53, UL: 1.58) (Figure 22). The result of the meta‐analysis also shows the measures are highly homogeneous (*I* ^2^: 0.0%, *p* = 0.428; *τ* ^2^ = 0.000).

###### 5.4.3.2 Effect sizes not included in meta‐analysis

Sharpe (2002) provided one predictor for victimization experiences as being bullied in school. The effect shows a positive but statistically nonsignificant association between such type of negative life events and organized crime membership (log OR: 0.37, LL: −0.06, UL: 0.81).

###### 5.4.3.3 Qualitative studies

Four qualitative studies examined the relation between negative life events and individuals' involvement in organized criminal groups (Kemp et al., 2020; Kleemans & De Poot, 2008; May & Bhardwa, 2018; Van Koppen, 2013). These include financial setback and debts (Kemp et al., 2020; Kleemans & De Poot, 2008; May & Bhardwa, 2018; Van Koppen, 2013) but also personal frustration and lack of excitement and results (Van Koppen, 2013). Traumatic events are also frequently mentioned such as the death of a relative or imprisonment (Kemp et al., 2020), or a messy divorce (May & Bhardwa, 2018).

#### 5.4.4 Offence and/or contact with CJ system

##### Predictors—Meta‐analyses

Four studies examined a total of 18 measures of offending or contact with the criminal justice system before the recruitment into organized crime (Blokland et al., 2019; Francis et al., 2013; Kirby et al., 2016; Sharpe, 2002; Van Koppen et al., 2010). These estimates were treated as predictors only if there was sufficient information that they were measured before the outcome variable.^15^ Two studies reported a single individualized risk factor. Sharpe (2002) measured engagement in delinquent behavior of adult gang members and Van Koppen et al. (2010) reported a measure of prior criminal record (at least one prior offence) for organized crime membership. The other two studies comprised multiple variables measuring prior involvement in offending (Blokland et al., 2019; Francis et al., 2013; Kirby et al., 2016). In particular, Francis et al. (2013)/Kirby et al. (2016) investigated the offending histories of individuals involved in organized crime in the United Kingdom, providing a total of 12 estimates across two types of comparison groups (serious offenders, offenders in general). Blokland et al. (2019) reported four measures related to criminal records of outlaw motorcycle gang members compared to offenders in general and population sample. Given the lack of independence among measures, the estimates of each study were synthesized—first by comparison group, and subsequently into a single measure—and the resulting pooled effect was included in the final meta‐analysis.

**Figure 23:**
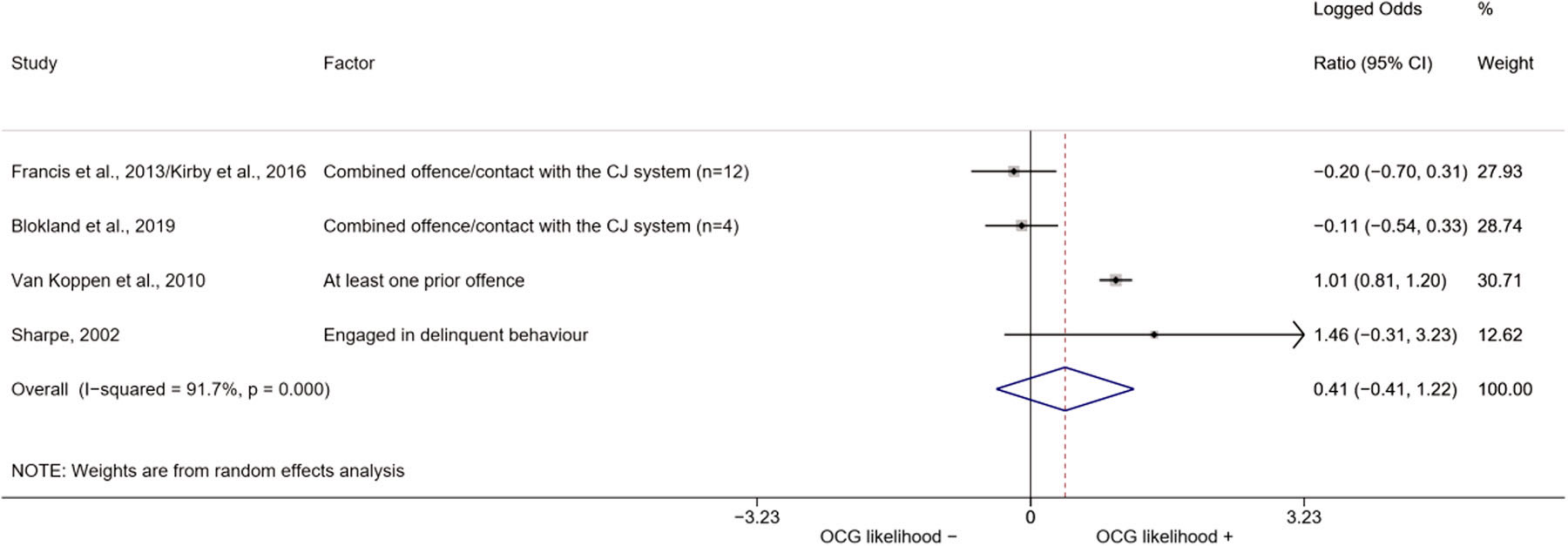
Offence and/or contact with CJ system—Predictors

The overall pooled effect indicates a positive but statistically nonsignificant association between prior offending or contact with the criminal justice system and involvement into organized criminal groups (log OR: 0.41, LL: −0.41, UL: 1.22) (Figure 23). Also, result of the meta‐analysis shows that there is significant heterogeneity amongst the effects (*I* ^2^: 91.7%, *p* < 0.001; *τ* ^2^ = 0.326).

The relatively large number of effect sizes enabled classifying predictors into one subcategory named criminal record or ever convicted/fined. This subcategory comprises predictors from more than one study, thus allowing to conduct a further meta‐analysis.

###### 5.4.4.1 Age first offence/conviction

**Figure 24:**
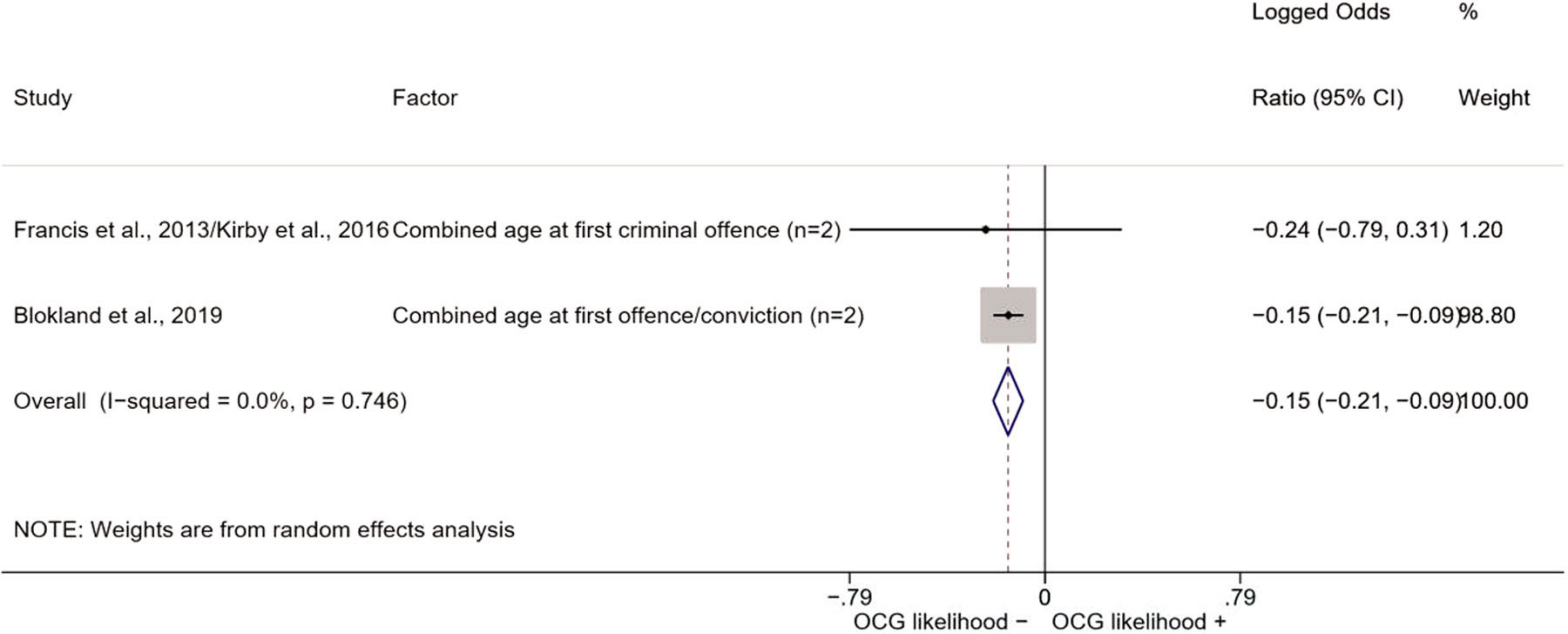
Age first offence/conviction

Two studies assessed the association between age at first offence or conviction and organized crime membership, reporting a total of four predictors (Blokland et al., 2019; Francis et al., 2013; Kirby et al., 2016). Blokland et al. (2019) measured criminal history before recruitment into outlaw motorcycle gangs and provided two estimates for individuals convicted at least once: age of first known conviction and age of first known incarceration. Francis et al. (2013)/Kirby et al. (2016) reported two measures relating to age at first criminal offence across two comparison group (serious offenders, offenders in general). For each study, the predictors were first synthesized before their inclusion in the analysis. The overall pooled effect shows that the age at first offence or conviction is negatively associated with organized crime membership (log OR: −0.15, LL: −0.21, UL: −0.09) (Figure 24). The result also shows that the measures are highly homogeneous (*I* ^2^: 0.0%, *p* = 0.746; *τ* ^2^ = 0.000).

###### 5.4.4.2 Ever convicted/fined—Predictors

**Figure 25:**
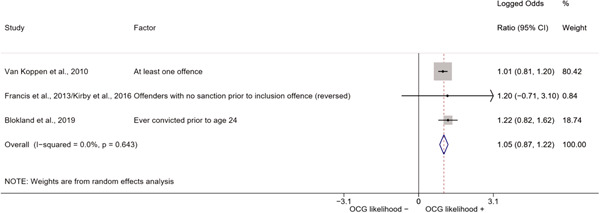
Ever convicted/fined—Predictors

Three studies investigated criminal record or convictions/fines before onset of organized crime membership, reporting a total of four predictors (Blokland et al., 2019; Francis et al., 2013; Kirby et al., 2016; Van Koppen et al., 2010). Francis et al. (2013)/Kirby et al. (2016) provided two measures relating to percentage of offenders with no sanction before inclusion offence (i.e., organized crime‐related offence). These measures were reverse coded to represent having a sanction before inclusion offence. Van Koppen et al. (2010) reported a measure for having at least one offence and Blokland et al. (2019) measured having ever been convicted before age 24 (i.e., prior the onset of organized crime membership). The overall pooled estimate suggests that having a criminal history is a statistically significant risk factor (log OR: 1.05, LL: 0.87, UL: 1.22) (Figure 25). Results also show that the measures are highly homogeneous (*I* ^2^: 0.0%, *p* = 0.643; *τ* ^2^ = 0.000).

###### 5.4.4.3 N. of convictions—Predictors

**Figure 26:**
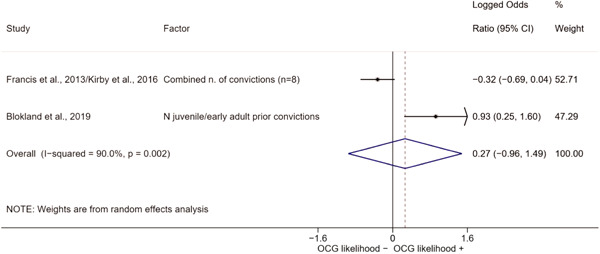
N. of convictions–Predictors

Two studies provided a total of nine measures of number of convictions before onset of organized crime membership (Blokland et al., 2019; Francis et al., 2013; Kirby et al., 2016). Francis et al. (2013)/Kirby et al. (2016) reported eight predictors, four for each comparison group (offenders in general, serious offenders) relating to the number of prior convictions relating to prior sanction occasions (court appearances, police caution occasions), criminal offences, convictions, or conviction occasions. The measures were first synthesized before their inclusion in the analysis. Blokland et al. (2019) reported an estimate of the number of juvenile/early adult convictions for those convicted at least once before age 25 (a cut‐off point for organized crime membership). Overall, the pooled effect indicates no significant relation between number of prior convictions and organized crime membership (log OR: 0.27, LL: −0.96, UL: 1.49) (Figure 26), with high heterogeneity amongst the measures (*I* ^2^: 90.0%, *p* = 0.002; *τ* ^2^ = 0.703).

###### 5.4.4.4 Effect sizes not included in meta‐analysis—Predictors

Francis et al. (2013)/Kirby et al. (2016) provided two predictors relating to time from onset to inclusion sanction (career duration, in years). The pooled effect shows a positive but statistically nonsignificant association with organized crime membership (log OR: 0.57, LL: −0.52, UL: 1.66), with high heterogeneity amongst the measures (*I* ^2^: 99.7%, *p* < 0.001; *τ* ^2^ = 0.617). We did not include these predictors due to limited comparability with the other included measures.

###### 5.4.4.5 Correlates—Meta‐analyses

Three studies reported a total of 8 correlates of offence and/or contact with the criminal justice system (Adams & Pizarro, 2014; Blokland et al., 2019; Klement, 2016). Adams and Pizarro (2014) provided a correlate of the number of arrests of gang members (vs. serious criminals). Blokland et al. (2019) conducted a study on OMCGs and provided six correlates across two comparison groups (population sample, offenders in general): three binary variables of ever being convicted, incarcerated, or fined; three continuous variables of number of convictions and/or fines. Lastly, Klement (2016) reported a continuous variable of the number of overall convictions of OMCG members (vs. offenders in general).

**Figure 27:**
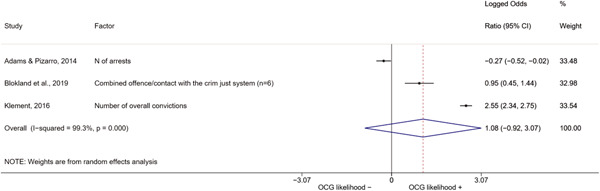
Offence and/or contact with CJ system—Correlates

Overall, the pooled effect indicates positive but statistically nonsignificant association between offending and/or contact with the criminal justice system and involvement into organized criminal groups (log OR: 1.08, LL: −0.92, UL: 3.07) (Figure 27). Also, the result shows high and significant heterogeneity between studies (*I* ^2^: 99.3%, *p* < 0.001; *τ* ^2^ = 3.068).

###### 5.4.4.6 N. of convictions—Correlates

Three studies investigated the association between individuals' number of convictions and involvement into organized crime groups, providing a total of five correlates (Adams & Pizarro, 2014; Blokland et al., 2019; Klement, 2016). Adams and Pizarro (2014) reported an estimate of the number of arrests of gang members (vs. serious criminals); Blokland et al. (2019) reported three continuous variables of number of convictions and/or fines (OMCG members vs. offenders in general); and Klement (2016) reported a continuous variable of the number of overall convictions of OMCG members (vs. offenders in general).

**Figure 28:**
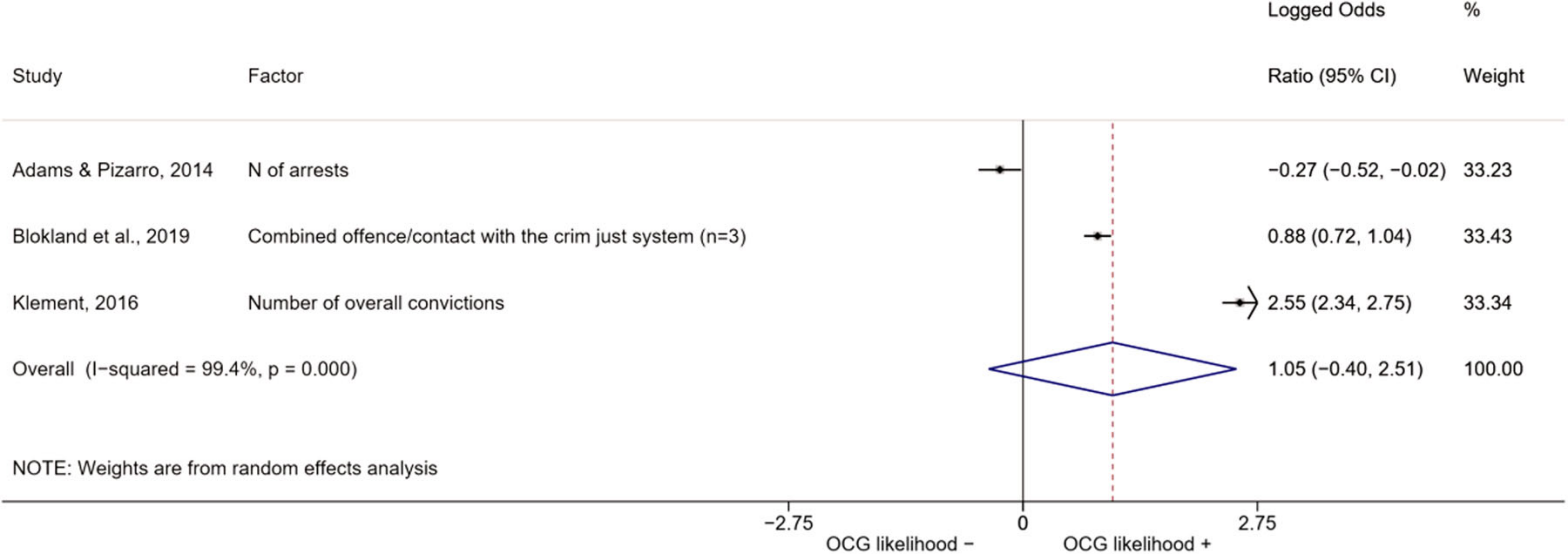
N. of convictions—Correlates

The pooled effect suggests a positive but statistically nonsignificant association with organized crime membership (log OR: 1.05, LL: −0.40, UL: 2.51) (Figure 28), with high and significant heterogeneity amongst the measures (*I* ^2^: 99.4%, *p* < 0.001; *τ* ^2^ = 1.643).

###### 5.4.4.7 Effect sizes not included in meta‐analysis—Correlates

Blokland et al. (2019) provided two correlates relating to age at last known conviction (or incarceration) for individuals' convicted at least once (OMCG members vs. offenders in general). The pooled effect suggests a positive but statistically nonsignificant association with organized crime membership (log OR: 0.37, LL: −0.10, UL: 0.85), with no significant heterogeneity amongst the measures (*I* ^2^: 65.4%, *p* = 0.089; *τ* ^2^ = 0.076). We did not include these correlates due to limited comparability with the other included measures.

###### 5.4.4.8 Qualitative studies

Fifteen studies highlighted organized crime groups' preference for individuals with a prior criminal history (Arlacchi, 1983; Brancaccio, 2017; Chalas & Grekul, 2017; Decker & Chapman, 2008; Densley, 2012; Gambetta, 1993; Hess, 1970/1973; Kemp et al., 2020; Kleemans & De Poot, 2008; Leukfeldt et al., 2019; Paoli, 2003; Van Koppen & De Poot, 2013; Van Koppen, 2013; Van Koppen et al., 2010; Varese, 2011). Differently from the quantitative literature, the qualitative studies usually referred to a generic criminal background rather than to specific characteristics of criminal careers. For mafia groups, the studies pointed out that a criminal background indicates contempt toward legal institutions and criminally‐relevant skills (Arlacchi, 1983; Brancaccio, 2017; Gambetta, 1993; Hess, 1970/1973; Paoli, 2003; Varese, 2011). For gangs, past criminal behavior is considered as the best sign of criminal potential, and a sign of distinction (Chalas & Grekul, 2017; Densley, 2012). A criminal background was also frequent in drug‐trafficking organizations and other organized criminal groups (Decker & Chapman, 2008; Kemp et al., 2020; Kleemans & De Poot, 2008; Leukfeldt et al., 2019; Van Koppen & De Poot, 2013; Van Koppen, 2013; Van Koppen et al., 2010).

Regarding the onset of criminal activity, the qualitative literature indicated that many organized crime members were early onset offenders with a long list of crimes committed (Kleemans & De Poot, 2008; Van Koppen et al., 2010); however, several studies also emphasized the relevant share of late onset offenders among organized crime members (Kemp et al., 2020; Kleemans & De Poot, 2008; Van Koppen & De Poot, 2013; Van Koppen et al., 2010).

##### Offence type

Four studies (Decker et al., 2014; Klement, 2016; Pedersen, 2018; Pyrooz et al., 2015—OMCG; Pedersen, 2018—Gang) examined the relation between different types of offences and organized crime membership, reporting a total of 31 estimates, of which 20 were classified as predictors (e.g. offences committed before the recruitment into organized criminal groups or first offences in the criminal career) and 11 as correlates (offences committed during the whole criminal career). We grouped these estimates under a common category. However, we only conducted meta‐analyses at the subcategory level to avoid mixing different types of offending. Given the theoretical relevance of violence for organized criminal groups and the availability of other measures of violence not associated with offending, we classified violent offences into a separate category (see below Violence).

###### 5.4.4.1 Predictors—Meta‐analyses

####### Drug offences—Predictors

**Figure 29:**
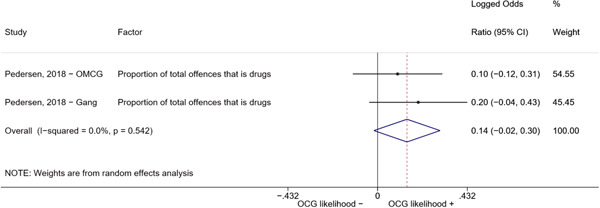
Drug offences—Predictors

Two studies (Pedersen, 2018—OMCG; Pedersen, 2018—Gang) investigated drug offences of OMCG members and gang members (compared to offenders in general), reporting a total of two predictors relating to the share of drug offences out of the total offences committed before recruitment into organized criminal groups. The pooled effect indicates a positive but statistically nonsignificant relation with organized crime membership (log OR: 0.14, LL: −0.02, UL: 0.30) (Figure 29), with no heterogeneity amongst the measures (*I* ^2^: 0.0%, *p* = 0.542; *τ* ^2^ = 0.000).

####### First offence: Drugs

**Figure 30:**
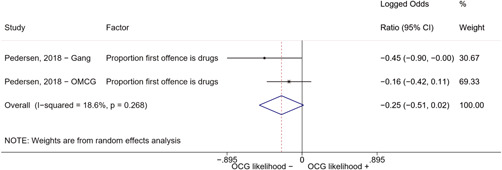
First offence: Drugs

Two studies (Pedersen, 2018—OMCG; Pedersen, 2018—Gang) examined the type of the first offences of OMCG members and gang members (compared to offenders in general), reporting a total of two predictors relating to the share of first drug offences out of the total first offences. The pooled effect shows a negative but statistically nonsignificant relation with involvement into organized criminal groups (log OR: −0.25, LL: −0.51, UL: 0.02) (Figure 30), with no significant heterogeneity between the studies (*I* ^2^: 18.6%, *p* = 0.268; *τ* ^2^ = 0.008).

####### Other offences

**Figure 31:**
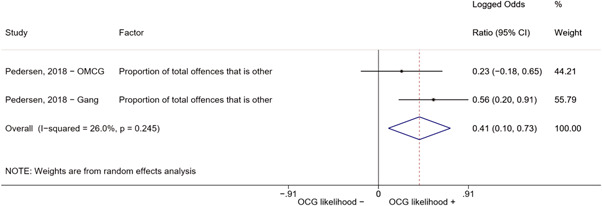
Other offences

Two studies (Pedersen, 2018—OMCG; Pedersen, 2018—Gang) investigated other, non‐specified, offences of OMCG members and gang members (compared to offenders in general), reporting a total of two predictors. The measures related to the share of other offences out of the total offences committed before recruitment into organized crime. The pooled effect shows a positive and statistically significant relation with involvement into organized criminal groups (log OR: 0.41, LL: 0.10, UL: 0.73) (Figure 31), with no significant heterogeneity amongst the measures (*I* ^2^: 26.0%, *p* = 0.245; *τ* ^2^ = 0.013).

####### First offence: Other

**Figure 32:**
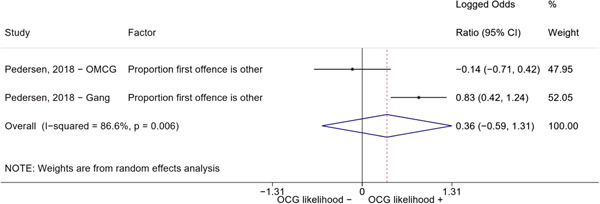
First offence: Other

Two studies (Pedersen, 2018—OMCG; Pedersen, 2018—Gang) investigated the type of the first offences of OMCG members and gang members (compared to offenders in general), providing a total of two predictors relating to the share of first other offences out of the total first offences. The pooled effect yielded a nonsignificant result (log OR: 0.36, LL: −0.59, UL: 1.31) (Figure 32), with high heterogeneity amongst the measures (*I* ^2^: 86.6%, *p* = 0.006; *τ* ^2^ = 0.008).

####### Property offences—Predictors

**Figure 33:**
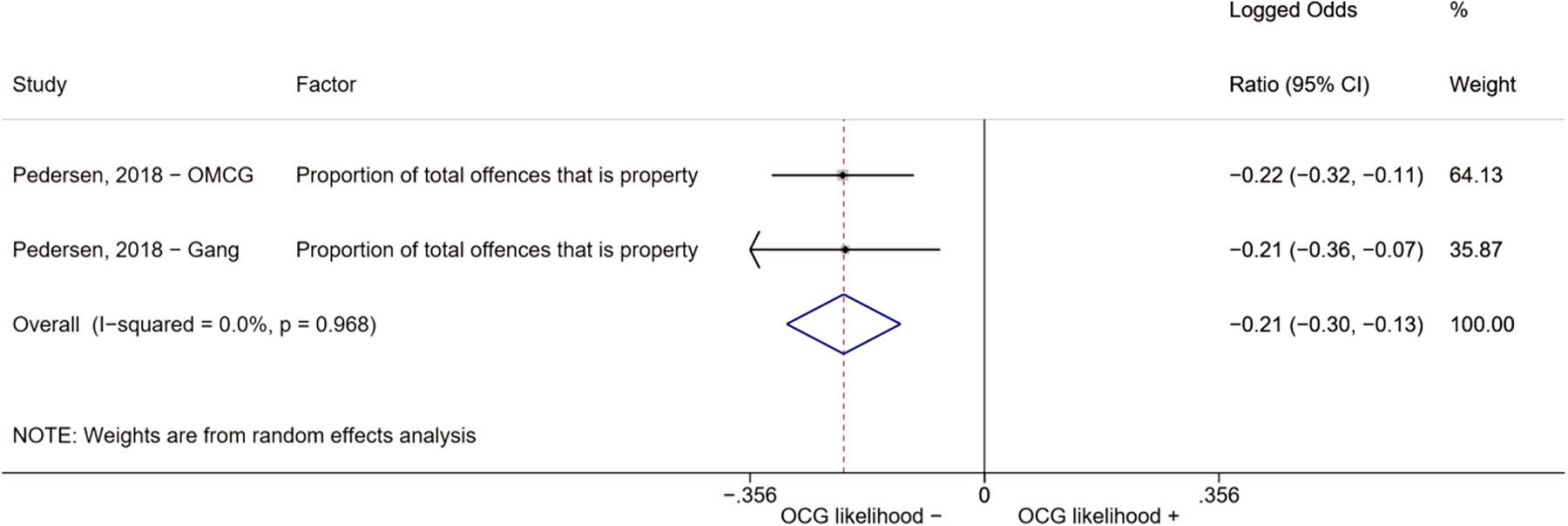
Property offences—Predictors

Two studies (Pedersen, 2018—OMCG; Pedersen, 2018—Gang) investigated property offences of OMCG members and gang members (compared to offenders in general), reporting a total of two predictors relating to the share of property offences out of the total offences committed before recruitment into organized crime. The pooled effect indicates a negative and statistically significant relation with organized crime membership (log OR: −0.21, LL: −0.30, UL: −0.13) (Figure 33), with no heterogeneity amongst the measures (*I* ^2^: 0.0%, *p* = 0.968; *τ* ^2^ = 0.000).

####### First offence: Property

**Figure 34:**
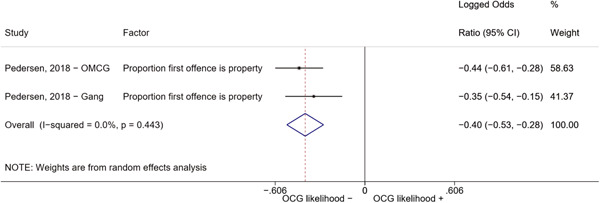
First offence: Property

Two studies (Pedersen, 2018—OMCG; Pedersen, 2018—Gang) analyzed the type of the first offences of OMCG members and gang members (compared to offenders in general), reporting a total of two predictors relating to the share of first property offences out of the total first offences. The pooled effect suggests a negative and statistically significant relation with involvement into organized criminal groups (log OR: −0.40, LL: −0.53, UL: −0.28) (Figure 34), with no significant heterogeneity between the studies (*I* ^2^: 0.0%, *p* = 0.443; *τ* ^2^ = 0.000).

####### Sexual offences—Predictors

**Figure 35:**
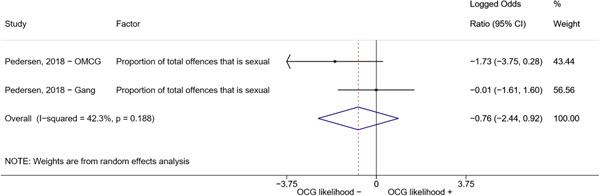
Sexual offences—Predictors

Two studies (Pedersen, 2018—OMCG; Pedersen, 2018—Gang) investigated sexual offences of OMCG members and gang members (compared to offenders in general), reporting a total of two predictors relating to the share of sexual offences out of the total offences committed before recruitment into organized crime. The pooled effect indicates a nonsignificant relation involvement into organized criminal groups (log OR: −0.76, LL: −2.44, UL: 0.92) (Figure 35), with no significant heterogeneity amongst the measures (*I* ^2^: 42.3%, *p* = 0.188; *τ* ^2^ = 0.632).

####### First offence: Sexual

**Figure 36:**
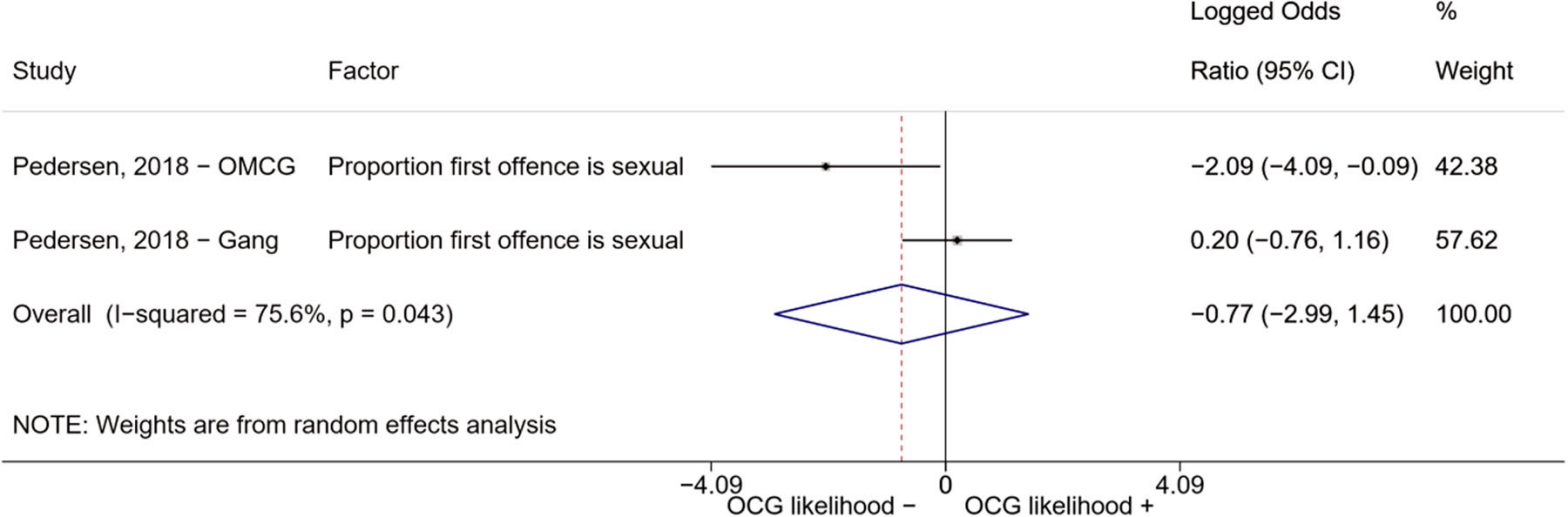
First offence: Sexual

Two studies (Pedersen, 2018—OMCG; Pedersen, 2018—Gang) investigated the type of the first offences of OMCG members and gang members (compared to offenders in general), reporting a total of two predictors relating to the share of first sexual offences out of the total first offences. The pooled estimate indicates a nonsignificant relation with organized crime membership (log OR: −0.77, LL: −2.99, UL: 1.45) (Figure 36), with significant heterogeneity between the studies (*I* ^2^: 75.6%, *p* = 0.043; *τ* ^2^ = 1.991).

####### Weapon offences—Predictors

**Figure 37:**
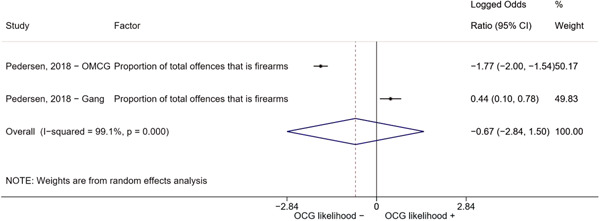
Weapon offences—Predictors

Two studies (Pedersen, 2018—OMCG; Pedersen, 2018—Gang) analyzed weapon offences of OMCG members and gang members (compared to offenders in general), reporting a total of two predictors relating to the share of firearm offences out of the total offences committed before recruitment into organized crime. The pooled effect indicates a nonsignificant relation with involvement into organized criminal groups (log OR: −0.67, LL: −2.84, UL: 1.50) (Figure 37), with high heterogeneity amongst the measures (*I* ^2^: 99.1%, *p* < 0.001; *τ* ^2^ = 2.426).

####### First offence: Weapon

**Figure 38:**
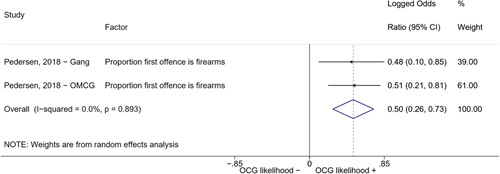
First offence: Weapon

Two studies (Pedersen, 2018—OMCG; Pedersen, 2018—Gang) examined the type of the first offences of OMCG members and gang members (compared to offenders in general), reporting a total of two predictors relating to the share of first firearm offences out of the total first offences. The pooled effect shows a positive and statistically significant relation with involvement into organized criminal groups (log OR: 0.50, LL: 0.26, UL: 0.73) (Figure 38), with no heterogeneity amongst the measures (*I* ^2^: 0.0%, *p* = 0.893; *τ* ^2^ = 0.000).

###### 5.4.4.2 Correlates—Meta‐analyses

####### 5.4.4.2.1 Drug offences—Correlates

**Figure 39:**
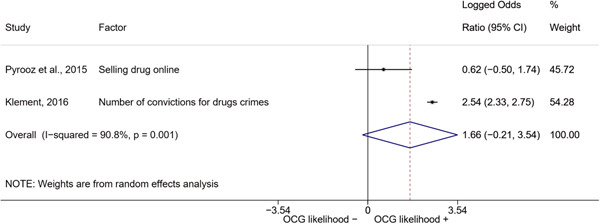
Drug offences—Correlates

Two studies assessed the association between drug offences and organized crime membership reporting a total of two correlates (Klement, 2016; Pyrooz et al., 2015). Pyrooz et al. (2015) analyzed deviant and criminal behavior of gang members (vs. population sample) in online settings and provided one measure for selling drugs online. Klement (2016) investigated the criminal background of OMCG members (vs. offenders in general) and reported one estimate of number of convictions for drug crimes. The pooled estimate indicates a positive but statistically nonsignificant relation between drug‐related criminal behavior and involvement into organized criminal groups (log OR: 1.66, LL: −0.21, UL: 3.54) (Figure 39), with high heterogeneity among the studies (*I* ^2^: 90.8%, *p* = 0.001; *τ* ^2^ = 1.674).

####### 5.4.4.2.2 Online‐related offending

Pyrooz et al. (2015) measured online‐related offending behavior of gang members (compared to population sample) providing a total of four correlates relating to: harassing other online, coordinate assaults through email or social networks, search social networks to steal from or rob people, and attacking others in real life because of inline occurrences. The pooled effect indicates that online criminal and deviant activity is positively associated with involvement into organized criminal groups (log OR: 0.85, LL: 0.20, UL: 1.51), with no significant heterogeneity between the measures (*I* ^2^: 29.2%, *p* = 0.237; *τ* ^2^ = 0.131).

####### 5.4.4.2.3 Property offences—Correlates

**Figure 40:**
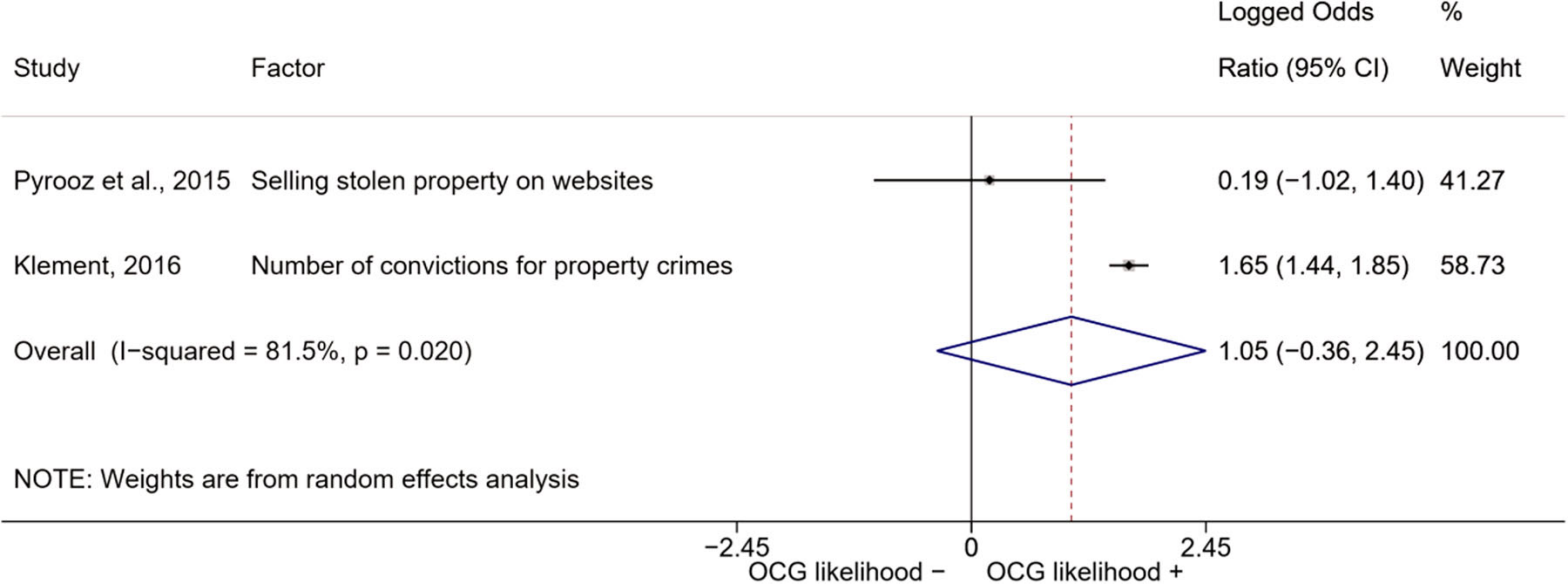
Property offences—Correlates

Two studies investigated the relation between property offences and organized crime membership, providing a total of two correlates (Klement, 2016; Pyrooz et al., 2015). Pyrooz et al. (2015) reported an estimate of selling stolen property online, while Klement (2016) reported one measure of number of convictions for property crimes. The result of the meta‐analysis yielded a nonsignificant result (log OR: 1.05, LL: −0.36, UL: 2.45) (Figure 40), with high heterogeneity between the studies (*I* ^2^: 81.5%, *p* = 0.020; *τ* ^2^ = 0.865).

####### 5.4.4.2.4 Sexual offences—Correlates

Klement (2016) assessed sexual offences of OMCG members (compared to offenders in general), reporting a correlate relating to number of conviction for sex crimes. The computed effect size indicates a nonsignificant association with involvement into organized criminal groups (log OR: 0.15, LL: −0.05, UL: 0.36).

####### 5.4.4.2.5 Traffic offences

Klement (2016) investigated traffic offences of OMCG members (compared to offenders in general), reporting a correlate relating to number of conviction for traffic offences. The computed effect size suggests a positive and statistically significant relation with OGG membership (log OR: 2.40, LL: 2.19, UL: 2.60).

####### 5.4.4.2.6 Weapon offences—Correlates

Klement (2016) analyzed weapon offences of OMCG members (compared to offenders in general), reporting a correlate of number of conviction for weapon crimes. The computed effect size indicates a positive relation with organized crime membership (log OR: 3.35, LL: 3.15, UL: 3.56).

##### Psychopathy and antisocial personality disorder

###### 5.4.4.1 Meta‐analyses

Four studies reported a total of 18 estimates relating to psychopathy and antisocial personality disorder (Bottini et al., 2017; Coid et al., 2013; Ostrosky et al., 2012; Schimmenti et al., 2014; Wood et al., 2017).^16^ Schimmenti et al. (2014) and Ostrosky et al. (2012) reported each a measure obtained through the Psychopathy Check List‐Revised (PCL‐R), while Bottini et al. (2017) reported two estimates obtained through the Psychopathic Personality Inventory‐Revised (PPI‐R). Lastly, Coid et al. (2013)/Wood et al. (2017) reported 14 measures across three comparison groups (violent men, population sample, affiliates) and relating to assistance for psychiatric problems (including consulted psychiatrist/psychologist, psychiatric admission, psychotropic medication), psychosis, and antisocial personality disorders. For each study reporting multiple measures, correlates were synthesized into a unique effect size before their inclusion in the analysis.

**Figure 41:**
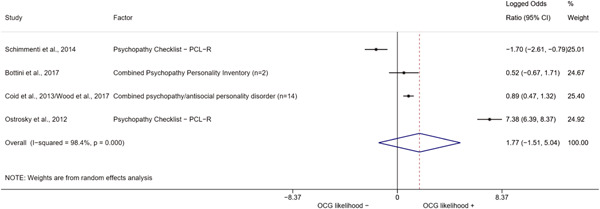
Psychopathy and antisocial personality disorder

Overall, the pooled estimate shows a positive but statistically nonsignificant relation between psychopathy and antisocial personality disorder and organized crime membership (log OR: 1.77, LL: −1.51, UL: 5.04) (Figure 41), with high heterogeneity amongst the measures (*I* ^2^: 98.4%, *p* < 0.001; *τ* ^2^ = 10.939).

####### 5.4.4.1.1 Antisocial personality disorder

**Figure 42:**
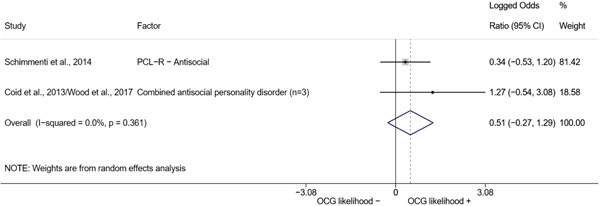
Antisocial personality disorder

Two studies investigated antisocial personality disorders (Coid et al., 2013; Schimmenti et al., 2014; Wood et al., 2017). Schimmenti et al. (2014) reported one measure obtained through the Psychopathy Check List‐Revised (PCL‐R), Coid et al. (2013)/Wood et al. (2017) three estimates of antisocial personality disorder that were combined before their inclusion in the analysis. The overall pooled effect shows no statistically significant association with organized crime membership (log OR: 0.51, LL: −0.27, UL: 1.29) (Figure 42), with no significant heterogeneity among the measures (*I* ^2^: 0.0%, *p* = 0.361; *τ* ^2^ = 0.000).

####### 5.4.4.1.2 Psychopathy

**Figure 43:**
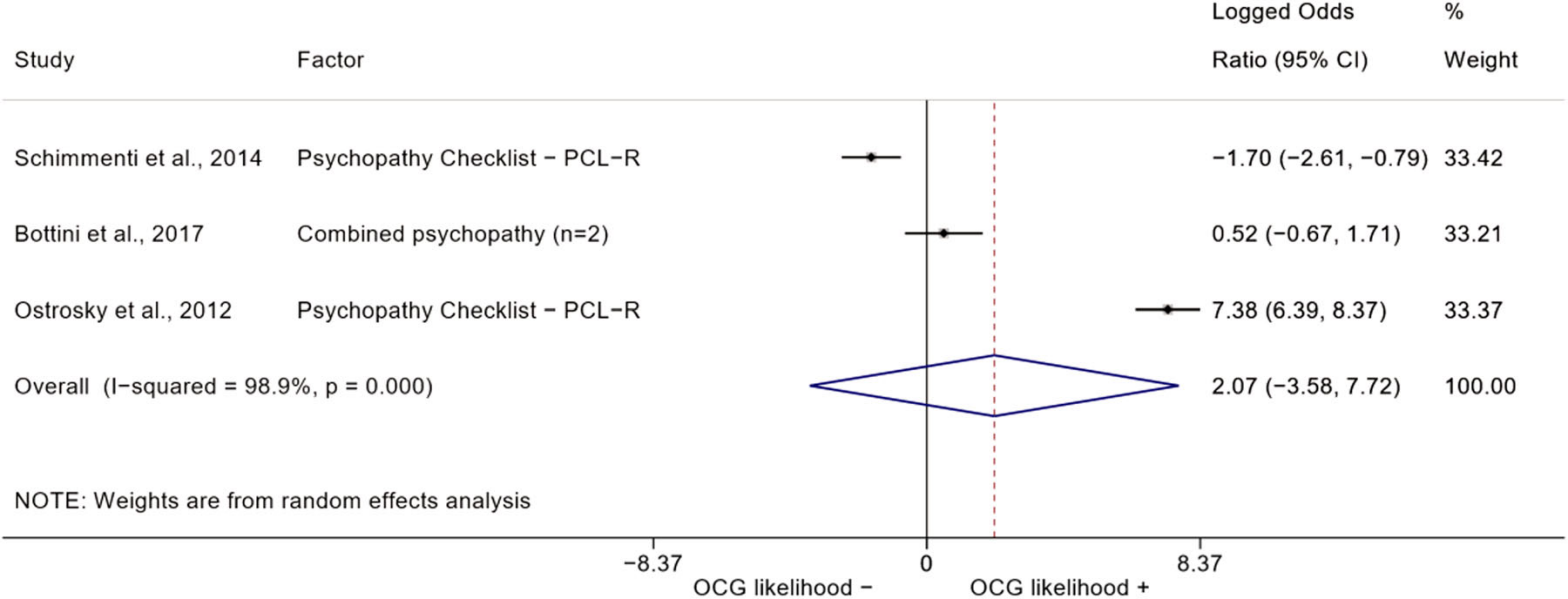
Psychopathy

Three studies investigated the relation between psychopathy and involvement into organized crime groups, reporting a total of four estimates (Bottini et al., 2017; Ostrosky et al., 2012; Schimmenti et al., 2014). The result of the meta‐analysis indicates a nonsignificant association between psychopathy and organized crime membership (log OR: 2.07, LL: −3.58, UL: 7.72) (Figure 43), with high heterogeneity among the effects (*I* ^2^: 98.9%, *p* < 0.001; *τ* ^2^ = 24.660).

###### 5.4.4.2 Qualitative studies

One qualitative study mentioned that individuals recruited into organized criminal groups can have antisocial personality disorders during their adulthood, which comes from an extensive history of negative and arrested development during adolescence (Hixon, 2010).

##### Religious beliefs

###### 5.4.4.1 Predictors

Sharpe (2002) provided three predictors of religious beliefs, two relating to being religious and one referring to non‐religiousness, which was reverse coded to represent being religious. The pooled estimate shows positive but statistically nonsignificant association with organized crime membership (log OR: 0.10, LL: −0.09, UL: 0.20), with high heterogeneity among the measures (I^2^: 95.3%, *p* < 0.001; *τ* ^2^ = 0.027).

###### 5.4.4.2 Correlates

Carvalho and Soares (2016) reported one correlate relating to religion (catholic, evangelical, other) for members of drug‐trafficking organizations. The effect shows a negative and significant association (log OR: −0.88, LL: −1.14, UL: −0.61), with being religious decreasing the likelihood of organized crime membership by a factor of 0.88.

##### Sanctions

###### 5.4.4.1 Meta‐analyses

**Figure 44:**
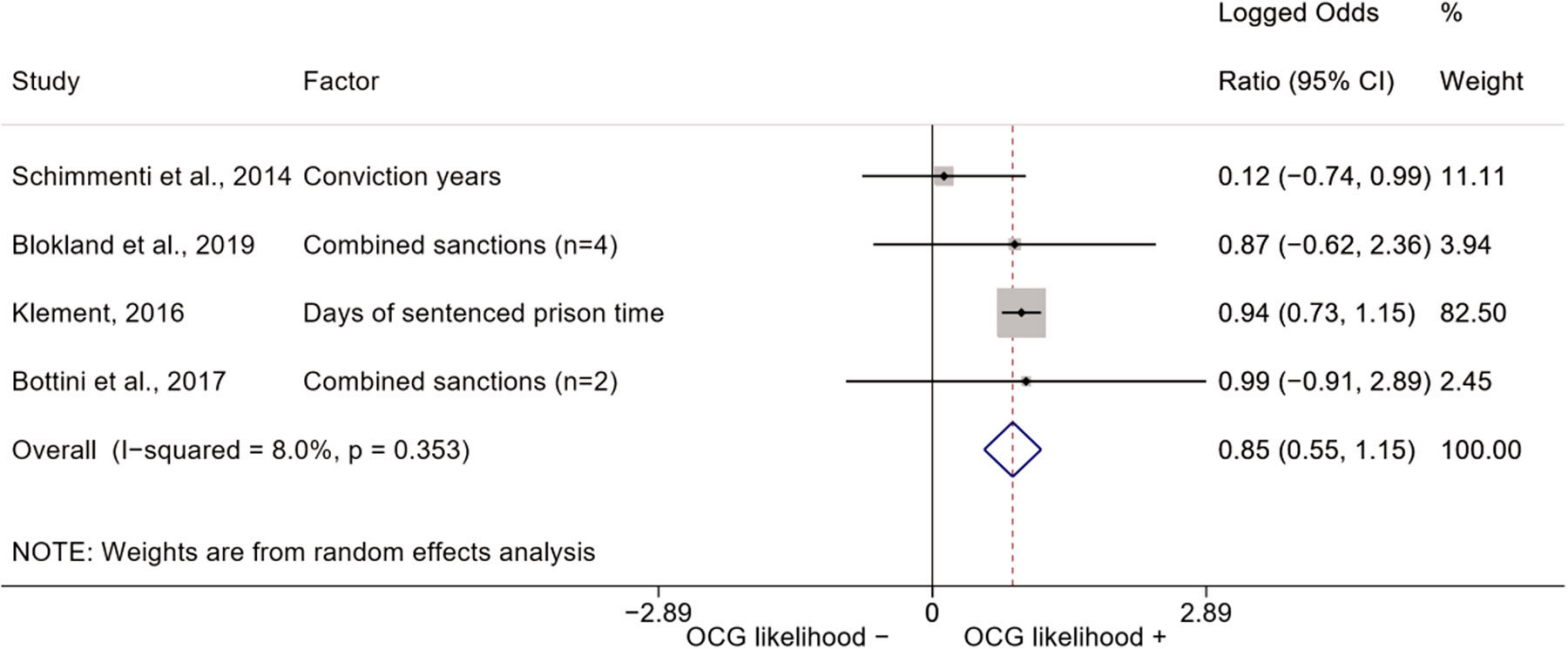
Sanctions

Four studies measured the relation between criminal sanctions and organized crime membership, reporting a total of eight correlates (Blokland et al., 2019; Bottini et al., 2017; Klement, 2016; Schimmenti et al., 2014). Schimmenti et al. (2014) provided a continuous measure of conviction years, while Klement (2016) reported a correlate of sentenced prison time. Blokland et al. (2019) provided four measures for individuals with at least one conviction: number of incarcerations, total incarceration length, total amount fined, and ever been incarcerated. The measures were synthesized before their inclusion in the analysis. Bottini et al. (2017) reported two measures, detention duration and number of incarcerations, that were combined into a unique effect size. Overall, the pooled estimate indicates that criminal sanctions are positively associated with organized crime membership (log OR: 0.85, LL: 0.55, UL: 1.15) (Figure 44), with no significant heterogeneity between the studies (*I* ^2^: 8.0%, *p* = 0.353; *τ* ^2^ = 0.017).

####### 5.4.4.1.1 Prison experience

**Figure 45:**
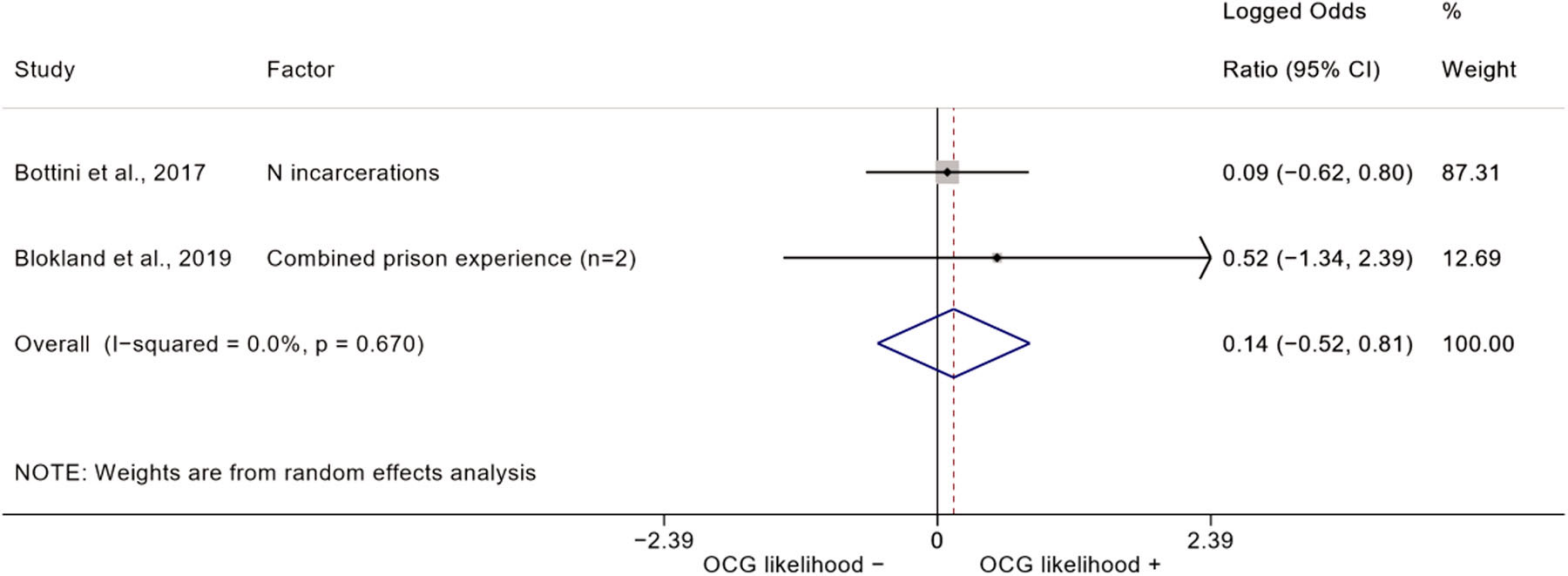
Prison experience

Two studies reported three estimates relating to individuals' prison experience (Blokland et al., 2019; Bottini et al., 2017). Blokland et al. (2019) provided two measures, one relating to number of incarcerations for those convicted at least once, and a binary measure of having been incarcerated, that were synthesized before their inclusion in the analysis. Bottini et al. (2017) included one correlate of number of incarcerations. The overall pooled effect yielded no significant results (log OR: 0.14, LL: −0.52, UL: 0.81) (Figure 45). Also, the measures are highly homogenous (*I* ^2^: 0.0%, *p* = 0.670; *τ* ^2^ = 0.000).

####### 5.4.4.1.2 Sanction seriousness

**Figure 46:**
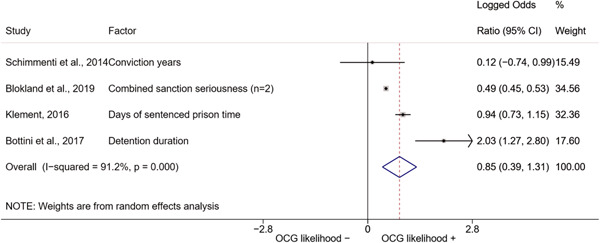
Sanction seriousness

Four studies measured the relation between sanction seriousness and organized crime membership, reporting a total of 5 correlates (Blokland et al., 2019; Bottini et al., 2017; Klement, 2016; Schimmenti et al., 2014). Schimmenti et al. (2014) provided a continuous measure of conviction years. Klement (2016) provided a correlate of sentenced prison time. Blokland et al. (2019) provided two measures of sanction seriousness for individuals with at least one conviction (total incarceration length and total amount fined) that were synthesized before their inclusion in the analysis. Lastly, Bottini et al. (2017) provided a correlate of detention duration. Overall, the pooled estimate indicates that sanction seriousness is positively associated with organized crime membership (log OR: 0.85, LL: 0.39, UL: 1.31) (Figure 46). Also, the result shows that there is high heterogeneity between the studies (*I* ^2^: 91.2%, *p* < 0.001; *τ* ^2^ = 0.157).

###### 5.4.4.2 Effect sizes not included in meta‐analysis

Van Koppen et al. (2010) provided two predictors relating to prison experience of organized crime offenders (compared to offenders in general). The pooled effect suggests that prior prison experience is a risk factor for involvement into organized criminal groups (log OR: 0.67, LL: 0.53, UL: 0.80), with no heterogeneity amongst the measures (*I* ^2^: 0.0%, *p* = 0.527; *τ* ^2^ = 0.000).

###### 5.4.4.3 Qualitative studies

Four studies mentioned prison experience as a turning point toward organized crime engagement (Chalas & Grekul, 2017; Kemp et al., 2020) or as a desired characteristic in organized criminal groups recruits (Densley, 2012; Van Koppen & De Poot, 2013).

##### Sex (male) (predictors)

###### 5.4.4.1 Meta‐analysis

**Figure 47:**
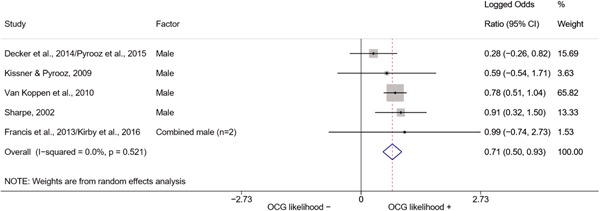
Sex (male)

Five studies investigated the relation between male sex and involvement into organized criminal groups (Decker et al., 2014; Francis et al., 2013; Kirby et al., 2016; Kissner & Pyrooz, 2009; Pyrooz et al., 2015; Sharpe, 2002; Van Koppen et al., 2010). All studies reported one measure of male, except for Francis et al. (2013)/Kirby et al. (2016) who reported two measures—first synthesized before inclusion in the analysis. The pooled effect results in a positive and statistically significant association between being male and organized crime membership (log OR: 0.71, LL: 0.50, UL: 0.93) (Figure 47). The result also shows that the measures are highly homogeneous (*I* ^2^: 0.0%, *p* = 0.521; *τ* ^2^ = 0.000).

###### 5.4.4.2 Qualitative studies

Nine studies considered the relation between sex and involvement in organized crime (Baird, 2018; Brotherton & Barrios, 2004; Gambetta, 1993; Gordon, 2000; Hixon, 2010; Knox et al., 1997; Spapens & Moors, 2020; Van San & Sikkens, 2017; Zhang & Chin, 2002). Consistent with the results of quantitative studies, also qualitative literature indicated that individuals who join organized criminal groups are predominantly males (Baird, 2018; Brotherton & Barrios, 2004; Gordon, 2000; Hixon, 2010; Knox et al., 1997; Zhang & Chin, 2002). The studies that specifically focused on the factors leading women to recruitment into organized criminal groups concluded that being women is not a precondition for recruitment; while women's participation in organized crime often occurs through family or emotional ties (Brotherton & Barrios, 2004; Gambetta, 1993; Spapens & Moors, 2020; Van San & Sikkens, 2017).

##### Silence/omertà

###### 5.4.4.1 Qualitative studies

The appreciation for individuals showing a silence/omertà attitude in organized criminal groups only emerged from qualitative literature, and in particular from six studies (Albini, 1971; Cressey, 1969; Gambetta, 1993; Hess, 1970/1973; Paoli, 2003; Pedersen, Unpublished). Silence/omertà typically emerges in mafia organizations as a value proving individuals' loyalty, which is essential to run illegal business based on secrecy and discretion (Albini, 1971; Cressey, 1969; Gambetta, 1993; Hess, 1970/1973; Paoli, 2003). However, silence/omertà has also been reported as a desirable characteristic in good and trusted gang members, who must prove that they are capable to be discreet and to maintain strict silence about gang business (Pedersen, Unpublished).

##### Social environment

###### 5.4.4.1 Meta‐analysis

**Figure 48:**
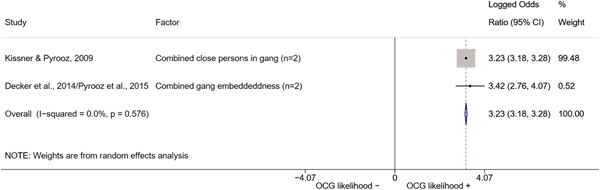
Social environment

Two studies provided a total of four measures of social environment, intended as having close persons in gang (or gang embeddedness) (Decker et al., 2014; Kissner & Pyrooz, 2009; Pyrooz et al., 2015). Decker et al. (2014)/Pyrooz et al. (2015) reported two correlates relating to the proportion of friends in gangs and frequency of contact with gang, Kissner and Pyrooz (2009) included two correlates relating to having gang friends and older sibling in gangs. The overall pooled estimate indicates that gang embeddedness is positively associated with involvement into organized crime groups (log OR: 3.23, LL: 3.18, UL: 3.28) (Figure 48). The result of the meta‐analysis also shows the measures are highly homogeneous (*I* ^2^: 0.0%, *p* = 0.576; τ^*2*^ = 0.000).

###### 5.4.4.2 Effect sizes not included in meta‐analysis

Kissner and Pyrooz (2009) investigated the relation between individuals' social environment and organized crime membership, reporting two predictors: parental gang membership and older relative gang membership. The pooled effect indicates a positive and statistically significant association with involvement into organized criminal groups (log OR: 3.19, LL: 2.21, UL: 4.16). The result also shows that there is no significant heterogeneity among the measures (*I* ^2^: 19.5%, *p* = 0.265; *τ* ^2^ = 0.156).

###### 5.4.4.3 Qualitative studies

Twenty six qualitative studies examined the role of social environment and relations in facilitating recruitment into organized criminal groups, because mutual knowledge guarantees trust (Albini, 1971; Ancrum & Treadwell, 2017; Arlacchi, 1983; Arsovska, 2015; Baird, 2018; Brancaccio, 2017; Chalas & Grekul, 2017; Decker & Chapman, 2008; Densley, 2012; Hess, 1970/1973; Ianni & Reuss‐Ianni, 1972; Kemp et al., 2020; Kleemans & De Poot, 2008; Kleemans & Van de Bunt, 2008; Leukfeldt et al., 2019; May & Bhardwa, 2018; Paoli, 2003; Pedersen, Unpublished; Spapens & Moors, 2020; Van Dijk et al., 2019; Van Koppen & De Poot, 2013; Van Koppen, 2013; Van San & Sikkens, 2017; Varese, 2011; Zhang & Chin, 2002). Even organized criminal groups operating online would not only rely on online social networks and forums, but also on pre‐established relationships in the offline world for recruiting individuals (Leukfeldt et al., 2019).

Kinship and blood ties were the most frequently mentioned factors driving recruitment into organized criminal groups due to established trust, prior interaction, protection against outsiders (Albini, 1971; Arlacchi, 1983; Arsovska, 2015; Baird, 2018; Brancaccio, 2017; Chalas & Grekul, 2017; Decker & Chapman, 2008; Densley, 2012; Hess, 1970/1973; Ianni & Reuss‐Ianni, 1972; Kemp et al., 2020; Kleemans & De Poot, 2008; Leukfeldt et al., 2019; Paoli, 2003; Spapens & Moors, 2020; Van Dijk et al., Unpublished; Van Koppen, 2013; Van San & Sikkens, 2017). Family members are a source of trusted members for drug trafficking organizations (Decker & Chapman, 2008; Van San & Sikkens, 2017), mafias (Albini, 1971; Arlacchi, 1983; Brancaccio, 2017; Hess, 1970/1973; Ianni & Reuss‐Ianni, 1972; Paoli, 2003), gangs (Baird, 2018; Chalas & Grekul, 2017; Densley, 2012) and other types of organized crime groups (Arsovska, 2015; Kemp et al., 2020; Kleemans & De Poot, 2008; Leukfeldt et al., 2019; Spapens & Moors, 2020; Van Dijk et al., Unpublished; Van Koppen, 2013).

Other types of relations examined by the qualitative literature are friends, acquaintances, and romantic relationships, which establish trust and opportunities for involvement into organized crime groups (Albini, 1971; Arsovska, 2015; Leukfeldt et al., 2019; May & Bhardwa, 2018; Pedersen, Unpublished; Van Koppen, 2013; Van Koppen, de Poot, Kleemans, et al., 2010; Van San & Sikkens, 2017).

In addition to kinship and other close personal relations, also leisure and work ties contribute to the involvement into organized crime (Decker & Chapman, 2008; Kleemans & De Poot, 2008; Leukfeldt et al., 2019; May & Bhardwa, 2018; Paoli, 2003). Professional ties are particularly relevant for individuals involved in organized crime well into their adulthood, due to the larger network of work‐related connections (Kleemans & De Poot, 2008; Kleemans & Van de Bunt, 2008).

Furthermore, recruitment into organized crime also favors individuals living in the same neighborhood or area of existing members, and especially when organized crime groups have control of specific territories (Arsovska, 2015; Baird, 2018; Ianni & Reuss‐Ianni, 1972; Kemp et al., 2020; Leukfeldt et al., 2019; Paoli, 2003; Pedersen, Unpublished; Spapens & Moors, 2020; Van Dijk et al., Unpublished; Van Koppen & De Poot, 2013; Van Koppen, 2013; Varese, 2011). The neighborhood enhances prior knowledge (Arsovska, 2015), favors observation and control (Densley, 2012), ensures that individuals share subcultural values and experiences (Pedersen, Unpublished; Van Koppen, 2013), provides a pool of potential volunteers with delinquent and criminal experiences (Ianni & Reuss‐Ianni, 1972). Furthermore, “bad” neighborhoods ensure that recruits have already been exposed to violence, delinquency, and illicit trade (Baird, 2018; Brotherton & Barrios, 2004; Kemp et al., 2020; Kleemans & De Poot, 2008).

Lastly, four studies emphasized that criminal relations before organized crime involvement can also lead individuals to join organized crime groups (Ancrum & Treadwell, 2017; Kemp et al., 2020; Leukfeldt et al., 2019; Van Koppen, 2013).

##### Troubled family environment

###### 5.4.4.1 Meta‐analyses

**Figure 49:**
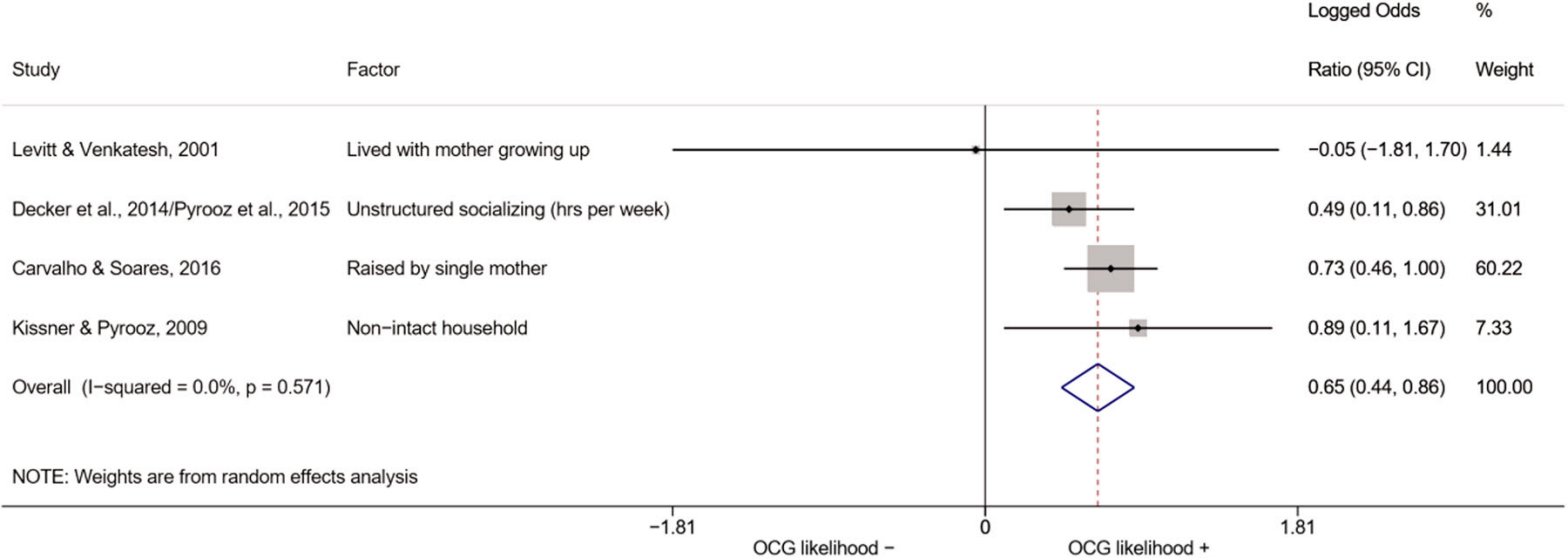
Troubled family environment

Four studies examined a total of four correlates of family environment (Carvalho & Soares, 2016; Decker et al., 2014; Kissner & Pyrooz, 2009; Levitt & Venkatesh, 2001; Pyrooz et al., 2015). The included studies assessed the relation between non‐intact household/unstructured socializing (i.e., growing up with or being raised by a single mother, lack of parental supervision) and organized crime membership. The pooled effect indicates a positive relation between growing up or living in a problematic family environment and involvement into organized criminal groups (log OR: 0.65, LL: 0.44, UL: 0.86) (Figure 49). The result of the meta‐analysis also shows that the measures are highly homogeneous (*I* ^2^: 0.0%, *p* = 0.571; *τ* ^2^ = 0.000).

####### 5.4.4.1.1 Raised by single mother

**Figure 50:**
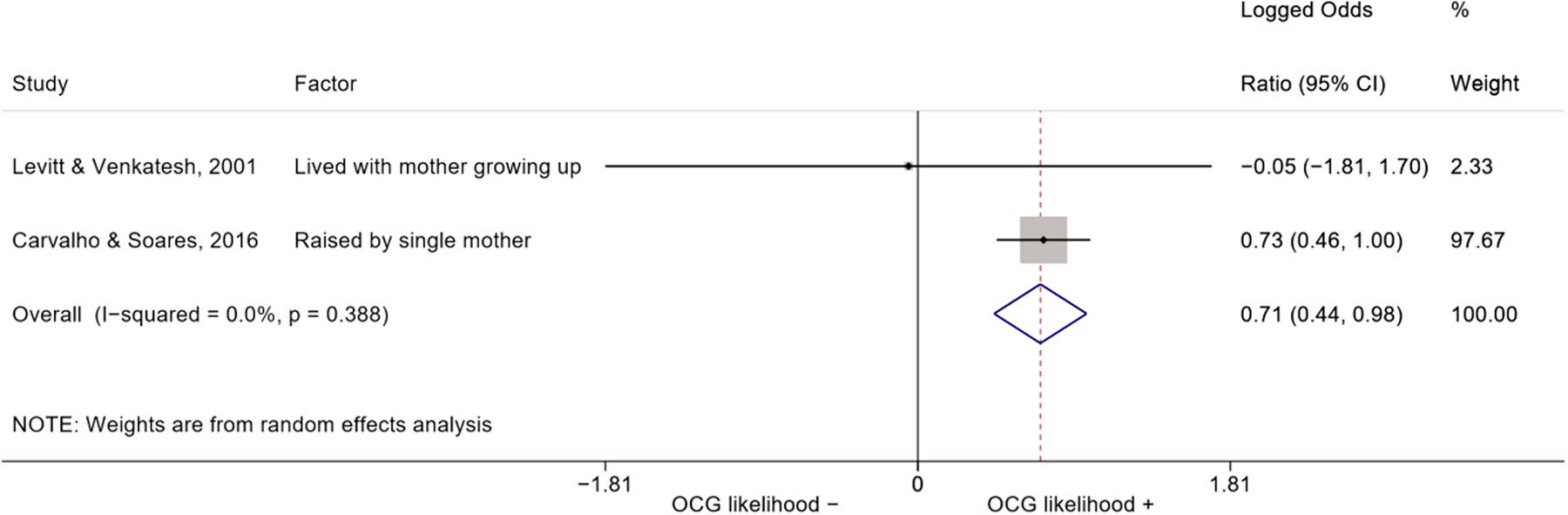
Raised by single mother

Two studies included a total of two correlates relating to being raised by a single mother (Carvalho & Soares, 2016; Levitt & Venkatesh, 2001). The pooled effect shows a positive and significant association between being raised by a single mother and involvement into organized criminal groups (log OR: 0.71, LL: 0.44, UL: 0.98) (Figure 50), with no measurable heterogeneity between the studies (*I* ^2^: 0.0%, *p* = 0.389; *τ* ^2^ = 0.000).

###### 5.4.4.2 Effect sizes not included in meta‐analysis

Sharpe (2002) included two predictors of the individuals' family environment: violent parents in household and lack of parental supervision growing up. The pooled effect indicates that troubled family environment is a positive and statistically significant risk factor for involvement into organized criminal groups (log OR: 3.19, LL: 2.21, UL: 4.16). Also, there is no significant heterogeneity among the measures (*I* ^2^: 19.5%, *p* = 0.265; *τ* ^2^ = 0.156).

###### 5.4.4.3 Qualitative studies

Three qualitative studies highlighted a relation between having a troubled family environment and becoming involved in organized criminal groups (Baird, 2018; Kleemans & De Poot, 2008; Spapens & Moors, 2020). This is the case of individuals with family dysfunctions becoming gang members in Colombia (Baird, 2018); of early onset offenders who experienced troubled childhood, family break‐up, parental drug‐use, or foster care in Dutch organized criminal groups (Van Koppen & De Poot, 2013); and of children of Dutch organized crime families who experienced divorce, regular absence of the father because of his criminal activities and detention, or traumas and stress caused by threats and violence in a life of crime (Spapens & Moors, 2020).

##### Violence

###### 5.4.4.1 Predictors—Meta‐analyses

**Figure 51:**
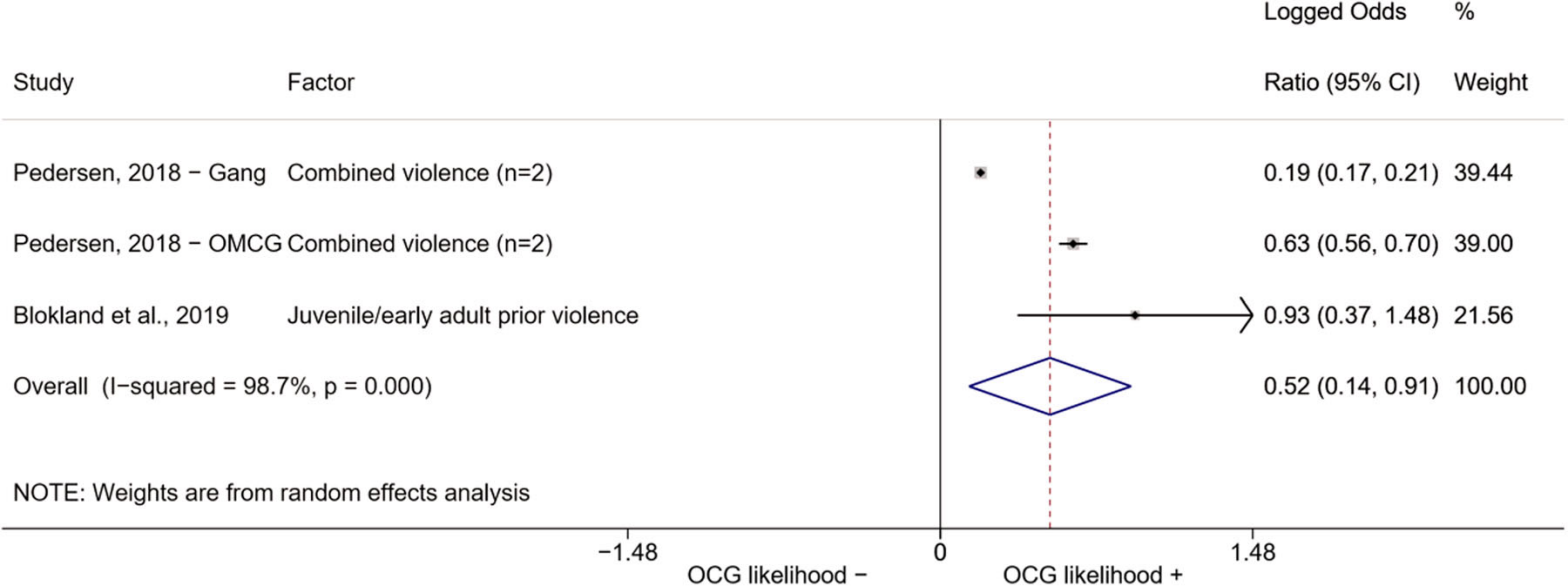
Violence—Predictors

Three studies provided a total of five estimates of violence before onset of organized crime membership (Blokland et al., 2019; Pedersen, 2018—OMCG; Pedersen, 2018—Gang). Pedersen (2018, OMCG) and Pedersen (2018, Gang) reported each two predictors relating to violence: share of first violent offences out of the total first offences, and share of violent offences out of the total offences. For each study, the measures were first synthesized into a unique effect size before the inclusion in the meta‐analysis. Blokland et al. (2019) reported one predictor of juvenile/early adult violence for those convicted at least once before age 25 (i.e., before involvement into organized crime groups). The overall effect shows a positive and statistically significant association between prior violence and organized crime membership (log OR: 0.52, LL: 0.14, UL: 0.91) (Figure 51), with high and significant heterogeneity among studies (*I* ^2^: 98.7%, *p* < 0.001; *τ* ^2^ = 0.097).

####### 5.4.4.1.1 Violent first offence

**Figure 52:**
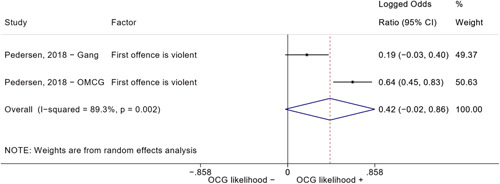
Violent first offence

Two studies (Pedersen, 2018—OMCG; Pedersen, 2018—Gang) examined the type of the first offences of OMCG members and gang members (compared to offenders in general), providing a total of two predictors relating to the share of first violent offences out of the total first offences. The pooled effect yielded a nonsignificant result (log OR: 0.42, LL: −0.02, UL: 0.86) (Figure 52), with high heterogeneity amongst the measures (*I* ^2^: 89.3%, *p* = 0.002; *τ* ^2^ = 0.090).

####### 5.4.4.1.2 Violent offences—Predictors

**Figure 53:**
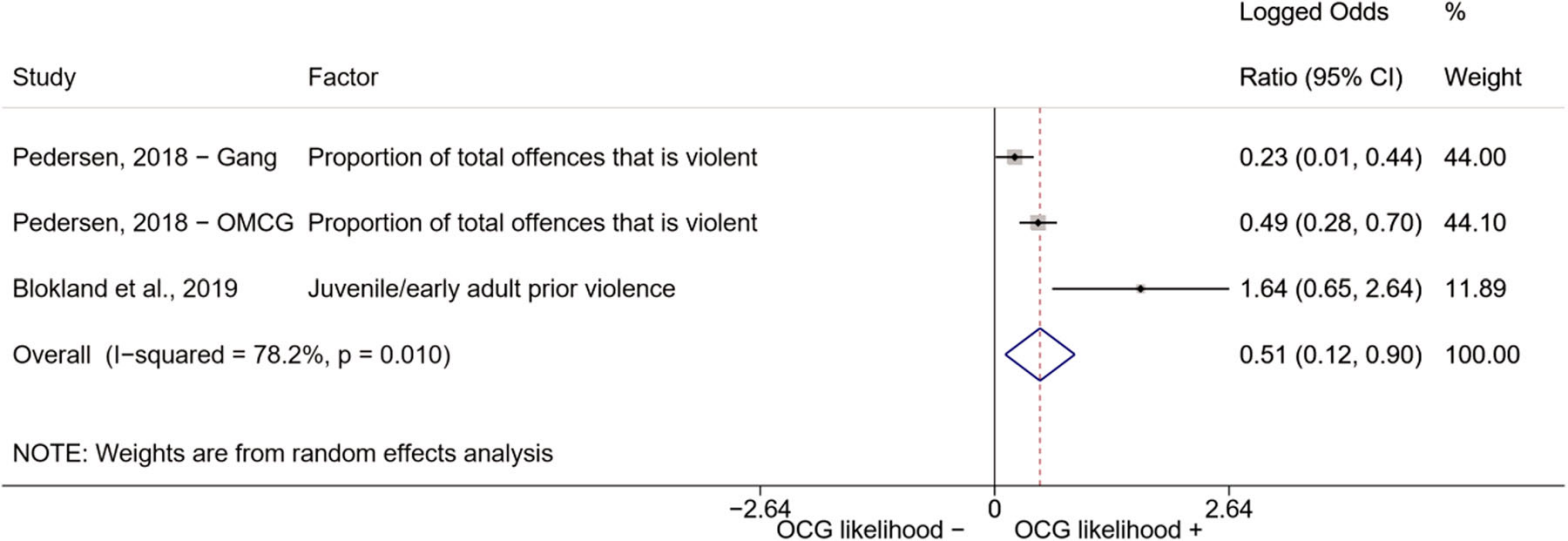
Violent offences—Predictors

Three studies provided a total of three estimates of violent offences before onset of organized crime membership (Blokland et al., 2019; Pedersen, 2018—OMCG; Pedersen, 2018—Gang). Pedersen (2018, OMCG) and Pedersen (2018, Gang) reported one predictor relating to the share of violent offences out of the total offences. Blokland et al. (2019) reported one predictor of juvenile/early adult violence for those convicted at least once before age 25 (i.e., before involvement into organized crime groups). The overall effect shows a statistically significant association between prior violent offences and organized crime membership (log OR: 0.51, LL: 0.12, UL: 0.90) (Figure 53), with significant heterogeneity among studies (*I* ^2^: 78.2%, *p* = 0.010; *τ* ^2^ = 0.079).

###### 5.4.4.2 Correlates—Meta‐analyses

**Figure 54:**
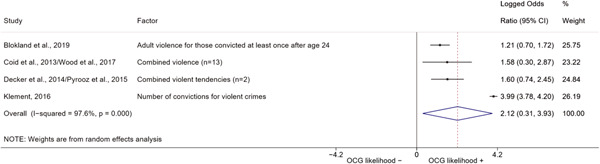
Violence—Correlates

Four studies investigated violence reporting a total of 17 correlates (Blokland et al., 2019; Coid et al., 2013; Decker et al., 2014; Klement, 2016; Pyrooz et al., 2015; Wood et al., 2017). Two studies provided one correlate: Klement (2016) of number of convictions for violent crimes, Blokland et al. (2019) of adult violence for those convicted at least once after age 24 (cut‐off for involvement into organized crime groups). Decker et al. (2014)/Pyrooz et al. (2015) reported two correlates of frequency of assaults with gang and adoption of the “code of the street.”^17^ Lastly, Coid et al. (2013)/Wood et al. (2017) measured violence across three comparison groups (violent men, affiliates, population sample) reporting 13 binary variables relating to: violent if disrespected, violent ruminations, excited by violence, sexual assault, stalking others, violence at work, previous conviction for violence, and instrumental violence. For each study reporting multiple correlates, the estimates were combined before their inclusion in the meta‐analysis. Overall, the pooled effect shows a positive and statistically significant association with involvement into organized criminal groups (log OR: 2.12, LL: 0.31, UL: 3.93) (Figure 54), with high heterogeneity among the measures (*I* ^2^: 97.6%, *p* < 0.001; *τ* ^2^ = 3.253).

####### 5.4.4.2.1 Instrumental violence

Coid et al. (2013)/Wood et al. (2017) examined violence of gang members (compared to violent men), reporting a correlate of instrumental violence. The computed effect size indicates a positive relation with organized crime membership (log OR: 3.15, LL: 2.70, UL: 3.61).

####### 5.4.4.2.2 Violent offences—Correlates

**Figure 55:**
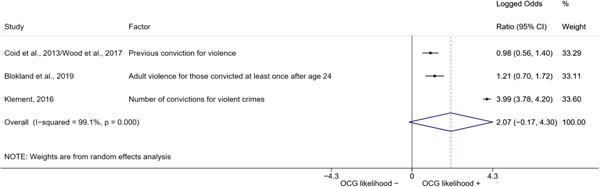
Violent offences—Correlates

Three studies investigated violent offences reporting a total of 3 correlates (Blokland et al., 2019; Coid et al., 2013; Klement, 2016; Wood et al., 2017). Overall, the pooled effect shows a positive but statistically nonsignificant association with involvement into organized crime (log OR: 2.07, LL: −0.17, UL: 4.30) (Figure 55), with high heterogeneity among the measures (*I* ^2^: 99.1%, *p* < 0.001; *τ* ^2^ = 3.851).

####### 5.4.4.2.3 Violent tendencies

**Figure 56:**
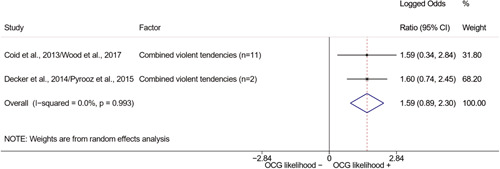
Violent tendencies—Correlates

Two studies investigated violent tendencies reporting a total of 13 correlates (Coid et al., 2013; Decker et al., 2014; Pyrooz et al., 2015; Wood et al., 2017). Coid et al. (2013)/Wood et al. (2017) measured violent tendencies across three comparison groups (violent men, affiliates, population sample) reporting 11 binary variables relating to: violent if disrespected, violent ruminations, excited by violence, sexual assault, stalking others, violence at work. Decker et al. (2014)/Pyrooz et al. (2015) reported two correlates referring to frequency of assaults with gang and adoption of the “code of the street.” For each study, the estimates were first synthesized into a unique effect size before the inclusion in the meta‐analysis. Overall, the pooled effect shows a positive and statistically significant association between violent tendencies and involvement into organized criminal groups (log OR: 1.59, LL: 0.89, UL: 2.30) (Figure 56). Also, there is no significant heterogeneity among the measures (*I* ^2^: 0.0%, *p* = 0.993; *τ* ^2^ = 0.000).

###### 5.4.4.3 Qualitative studies

Ten qualitative studies examined violent attitudes, tendencies, and offences among individuals who join organized criminal groups (Ancrum & Treadwell, 2017; Arlacchi, 1983; Baird, 2018; Brancaccio, 2017; Chalas & Grekul, 2017; Densley, 2012; Hess, 1970/1973; Spapens & Moors, 2020; Varese, 2011). Gang recruits must be willing to learn and display violence as a rite of passage to the ganging process, but also to perform gang activities and fight against rival groups. Several studies argue that individuals with a pre‐established reputation of disciplined violence, fighting skills and courage are often preferred (Baird, 2018; Chalas & Grekul, 2017; Densley, 2012; Pedersen, Unpublished). Displaying aggressiveness and having a reputation of violent tendencies and offences is also essential for individuals joining drug‐trafficking organizations (Ancrum & Treadwell, 2017); and mafia organizations (Arlacchi, 1983; Hess, 1970/1973; Varese, 2011), and because of this the recruitment of petty criminals and neofascist activists is reported (Varese, 2011). Finally, a study of Dutch organized crime families highlighted how children often grow in a context where violence is considered as an acceptable strategy to solve disputes and to obtain desired outcomes, and those who get to internalize and reproduce this approach are at risk of becoming organized crime active members (Spapens & Moors, 2020).

#### 5.4.5 Type of organized crime group as effect size moderator

Given the diversity of theoretical and operational definitions of organized criminal groups across countries and social context, we explored different types of organized crime groups as moderator variable for all meta‐analyses reporting a statistically significant heterogeneity.

We conducted a total of 32 moderator analyses, integrally reported in Supporting Information Appendix E: Moderator analyses by type of organized criminal group. In general, moderator analyses were affected by the small number of independent effect sizes available across different groups. We thus invite caution in interpreting the results. For 15 moderator analyses the number of independent measures was equal to the number of groups, and for 10 moderator analyses it exceeded the number groups by just one unit (e.g., three independent measures between two groups).

Overall, most moderator analyses reported a statistically significant between‐group heterogeneity, except for age, economic condition (risk factors), ethnicity—Black, ethnicity—White, low self‐control, Offence/contact with the CG system (predictors), sanction seriousness, violent offences (predictors), and violence and violent offences (correlates). While the amount of evidence is weak, the results suggest that there may be differences in the risk factors across types of organized criminal groups. Frequently, the moderator analyses showed that the association between risk factors and membership in one type of organized criminal group was statistically significant whereas the above‐presented meta‐analyses showed a nonsignificant association. Some moderator analyses showed opposite associations between a risk factor and different types of groups (see in Supporting Information Appendix E: Moderator analyses by type of organized criminal group: Figure 61. Moderator—Criminal versatility—Correlates, Figure 68. Moderator—Ethnicity, any (non‐White), Figure 69. Moderator—Foreign born, Figure 73. Moderator—Offence/contact with the criminal justice system—Correlates, Figure 74. Moderator—N. of convictions—Correlates, Figure 75. Moderator—Drug use and addiction problems, Figure 78. Moderator—Weapon offences—Predictors). For example, the moderator analysis for ethnicity—any non‐White comprised six independent effect sizes across gangs (*n* = 4), biker gangs (*n* = 1), and other organized crime groups (*n* = 1). The analysis reports a large and statistically significant (*p* = 0.000) heterogeneity among groups. Being of any non‐White ethnic group is positively associated with organized crime membership for gangs and other organized crime groups. Conversely, the association is negative for biker gangs (Figure 68. Moderator—Ethnicity, any [non‐White]).

The results rely only on a very small number of studies per group (mostly just one), and we warn against drawing conclusion from such limited evidence. Overall, we consider that the moderator analyses showed that the broad conceptualization of organized crime likely encompasses a variety of groups and that different risk factors may drive recruitment into the different types of groups. Future updates of this review and future research should distinguish the different types of organized criminal groups and further explore possible subgroup differences in determining the factors for recruitment.

## 6 DISCUSSION

### 6.1 Summary of main results

We summarize the results separately for predictors and correlates. For each analysis, we report the number of studies to enable an assessment of the amount of evidence contributing to each analysis. For an overview of the main results, we refer the reader to Tables 8 and 9.

#### 6.1.1 Predictors

The largest amount of evidence concerned predictors of ethnicity or race. However, results for any non‐White race (*n* = 6) or Black race (*n* = 6) were statistically nonsignificant. We found a negative association between White race (*n* = 4) and recruitment into organized crime, indicating that White individuals reported nearly half the odds of organized crime membership than other individuals. All the meta‐analyses showed a high degree of variability. One estimate of mixed race reported a negative association with the risk of involvement into organized crime groups.

There was strong evidence that male sex is a predictor of organized crime recruitment (*n* = 5). Males reported having twice the odds of membership than females.

We found mixed evidence on the association between prior offending and contact with the criminal justice system and organized crime membership. We found nonsignificant association between recruitment into organized crime and all the available independent estimates (*n* = 4), as well as the number of prior convictions (*n* = 2) and criminal career duration (*n* = 1). However, previously convicted/fined individuals had nearly three times the odds of organized crime membership. Furthermore, the commission of the first offence at a later age was associated with lower odds of recruitment into organized crime (*n* = 2). This counterintuitive finding, however, resulted from only two independent measures.

Individuals reporting prior violence (*n* = 3) showed 68% greater odds of organized crime involvement. We found similar results for individuals having committed violent offences (*n* = 3, OR 1.67). Yet both findings were based on studies with high variability. No significant association was found between organized crime recruitment and the commission of a violent offence as the first offence of a criminal career (*n* = 1).

There was weak evidence regarding the type of prior offences and participation in organized crime groups. We explored the category through ten subcategories, all relying on only two independent estimates. There was weak evidence that a first offence concerning weapons and the proportion of other offences are associated with 65% and 51% larger odds of organized crime membership, respectively. Conversely, the proportion of prior property offences, and whether the first offence was a property offence, were associated with 19% and 33% lower odds of organized crime recruitment, respectively. All other subcategories reported nonsignificant associations.

There was a nonsignificant association between criminal versatility (*n* = 2) and recruitment into organized crime.

We found very weak evidence for predictors regarding prior sanctions (*n* = 1), showing double the odds of organized crime recruitment. Also, very weak evidence was found for predictors regarding the social environment (*n* = 1) and a troubled family environment (*n* = 1), although both reported extremely high odds of organized crime membership.

No statistically significant associations were found for poor economic condition (*n* = 1), low self‐control (*n* = 1), negative life events (*n* = 1), and religious beliefs (n = 1).

#### 6.1.2 Correlates

The largest amount of evidence (between 10 and 6 independent estimates) concerned age, education and low self‐control.

There was no statistically significant association between age and organized crime membership (*n* = 10), with studies showing a very high degree of variability.

We investigated the association between education and membership of organized crime groups. All available estimates of education (*n* = 7) showed a negative association, with individuals with higher education reporting 45% lower odds of being organized crime members. The studies reported high levels of variability. Similarly, more years of education (*n* = 6) result in 25% lower odds of organized crime involvement. We found no statistically significant association for high school completion (*n* = 2) and parental education (*n* = 1).

We found relatively strong evidence on the association between low self‐control and organized crime recruitment. All available estimates (*n* = 6) show that individuals with low self‐control report twice the odds of being involved in organized crime groups, although the studies reported high degree of variability. The direction of the association was confirmed by the subcategory including only measures of low self‐control and risk‐taking behavior (*n* = 3), with 140% higher odds of organized crime membership. Conversely, no association was found with drug use and addiction problems (*n* = 3).

An average amount of evidence (between four and three independent effect sizes) regarded violence, sanctions, troubled family environment, psychopathy and antisocial personality disorder, economic condition, being in a relationship, and offence and/or contact with the criminal justice system.

All independent correlates of violence (*n* = 4) showed that violent individuals had over eight times the odds of being an organized crime member, although the studies reported a very high degree of variability. The subcategory focusing solely on violent offences (*n* = 4) reported no statistically significant association, while subjects with violent tendencies (*n* = 2) showed nearly five times the odds of organized crime membership. One independent estimate of instrumental violence also reported positive association, with 156% greater odds of involvement into organized crime.

All correlates on sanctions (*n* = 4) showed that individuals with any type of criminal sanction reported 134% higher odds of being organized crime members. Also effect sizes focusing on the seriousness of the criminal sanctions (*n* = 4) reported that individuals receiving more serious sanctions showed 134% larger odds of involvement into organized crime. The studies, however, showed very high variability. We found no association between prison experience and organized crime membership.

Individuals with a troubled family environment reported nearly twice the odds of organized crime recruitment (*n* = 4). The result was confirmed by the subcategory focusing on individuals raised by a single mother (*n* = 2), who reported over twice the odds of organized crime membership.

We found no statistically significant association between psychopathy and antisocial personality disorders and organized crime membership. We investigated the relation with three analyses, focusing on all available independent estimates (*n* = 4), psychopathy (*n* = 4), and antisocial personality disorders (*n* = 2). All analyses were statistically nonsignificant, and studies reported high degrees of variability.

We investigated the association between economic conditions and organized crime membership with two analyses. No statistically significant association was found between medium‐high economic conditions (as a protective factor) and organized crime recruitment (*n* = 3). Conversely, we found that individuals in poor economic conditions had three times the odds of organized crime membership (*n* = 3). However, the studies showed high levels of variability.

Being in a relationship (*n* = 3) unexpectedly reported a statistically significant and positive association with organized crime membership. Individuals in a relationship had over 2.5 greater odds of being involved in organized crime.

There was no statistically significant association between offences and contact with the criminal justice system and organized crime membership (*n* = 3). This finding was confirmed by the analysis of number of convictions (*n* = 3) and by one independent estimate of age of last known conviction.

All other factor categories reported a limited amount of evidence (two independent effect sizes or less).

There was a statistically significant and positive association between the social environment and organized crime involvement (*n* = 2). Individuals embedded in social relations associated with gangs had nearly 25 times the odds of being member of organized crime groups.

We found a positive relation between having experience negative life events and organized crime membership. All available estimates (*n* = 2) showed nearly two and half times higher odds of organized crime membership, while the subcategory on traumatic physical occurrences (*n* = 2) reported nearly three times higher odds.

Individuals with signs of depression (*n* = 2) had nearly twice the odds of being members of organized crime groups.

We explored the relation between living and household conditions and organized membership across six different subcategories. Individuals without children (*n* = 2) reported 167% larger odds of being involved in organized crime. All other five subcategories comprise only one independent estimate and never reported a statistically significant association.

We investigated the association between the type of committed offences and involvement into organized crime groups through six distinct subcategories. Only two subcategories comprised two independent estimates (drug and property offences) and reported no statistically significant association. We found a positive association between single effect sizes of weapon offences, traffic offences, and online‐related offending. No statistically significant association was found with a single measure of sexual offences.

There was no statistically significant association for anxiety (*n* = 2), cognitive functioning (*n* = 2), and criminal versatility (*n* = 2).

Only one independent estimate of the importance of organized crime group reported a positive and statistically significant association with membership. Individuals responding that the group was important to them reported nearly 18 times higher odds of joining criminal organizations.

Subjects engaging in deviant online activities (*n* = 1) had nearly twice the odds of involvement in organized crime. However, there was a nonsignificant association between nondeviant online activities and organized crime membership.

Individuals with religious beliefs (*n* = 1) reported nearly 60% lower odds of joining organized crime.

#### 6.1.3 Correspondence between predictors and correlates

We reported separately the results for predictors and correlates to avoid biases due to the observational, cross‐section designs of most studies. We acknowledge that the results from correlates require caution and may result from mere associations between factors and organized crime membership. Nevertheless, we found some correspondence with evidence from predictors.

Eleven categories comprised both predictors and correlates. For offence type—weapons, sanctions, social environment, troubled family environment, and violence both predictors and correlates indicated a statistically significant association with recruitment into organized crime. Correspondence between the two types of factors point to particularly relevant categories in understanding the involvement into organized crime.

For offence and/or contact with the criminal justice system we found a statistically significant relation between predictors of prior criminal activity (ever convicted/fined and age of first offence/conviction) and organized crime membership. However, the findings about correlates found not statistically significant association.

Regarding economic condition, low self‐control, negative life events, and religious beliefs our we recorded only significant associations for correlates. We thus caution against drawing causal implications from these categories, as the analysis of predictors reported nonsignificant results.

For criminal versatility we found no statistically significant relation for either predictors or correlates.

### 6.2 Overall completeness and applicability of evidence

This systematic review comprised multiple databases and languages, with no time‐period or geographic restrictions. We integrated the results of the search with reference search and contributions by several scholars active in the field. The search process yielded nineteen eligible studies addressing multiple risk factors for the recruitment into organized crime groups. Thanks to the cooperation of several authors, we were able to integrate the data and extract most of available information. We failed to retrieve sufficient information from only one study. We trust that our search process was able to identify all existing research meeting our inclusion criteria.

While the included studies provided insight on multiple risk factors, we consider that this body of evidence is still incomplete for several reasons. First, it focused on a small set of countries. Second, while many studies were published in recent years, several included qualitative studies date to the 1960s or 1970s, and their findings may have little relevance for understanding contemporary recruitment into organized crime. Third, most of the studies adopted a cross‐sectional design, and only a minority of the extracted effect sizes could be considered predictors. Consequently, many of our analyses examine correlates of organized crime membership and it is impossible to establish a clear causal direction. Fourth, studies differed remarkably on the types of examined factors. As a result, most of the associations we were able to analyze included only one or two independent measures. Only in few cases the analyses comprised more than four independent effect sizes.

Furthermore, this review has also systematically searched and analyzed qualitative research. We consider that this decision offered additional insights on the possible risk factors of organized crime membership. As shown in Table 6, there is only partial overlap between the evidence from quantitative studies and the results of qualitative research, suggesting that the available evidence from quantitative research did not explore several potential risk factors. In particular, we were unable to retrieve any independent measure regarding legitimate jobs/skills in the quantitative studies, while thirteen qualitative works pointed out that individuals with specific professional positions or skills may be at higher risk of recruitment into organized crime. Similarly, only one quantitative study investigated the importance of motivations for recruitment into organized crime. Remarkably, nineteen qualitative studies examined the different motivations for individuals to join criminal organizations, emphasizing the importance of factors such as the sense of social cohesion and subcultural values, financial gain, and ambitions for a successful life and social status. Furthermore, the quantitative literature yielded only two independent estimates on the impact of the social environment on the risk of recruitment into organized crime. Qualitative studies often analyzed these mechanisms, with 25 studies focusing on elements such as the role of family and kinship, friends and acquaintances, professional connections, coming from the same neighborhood, and criminal relations. Lastly, six qualitative works analyzed the capacity to keep silence as a core skill for organized crime recruits, while we could extract no quantitative measure addressing this factor.

### 6.3 Quality of the evidence

In general, the nineteen quantitative studies offered detailed analysis of the background, hypotheses, and methods employed. However, they rarely aimed at establishing the risk factors for recruitment into organized crime and this affected the quality of the information we could derive from them. Our risk‐of‐bias assessment pointed out that they mostly adopted a cross‐sectional design, limiting the capacity to establish a clear causal direction between factors and the recruitment into organized crime. Furthermore, only a few studies matched the organized crime and the non‐organized‐crime samples, and the matching strategies often differed. Also, the size of the samples of organized crime members showed substantial variation, ranging from 29 to 4019 (mean = 525, median = 209). The pooled total of nearly 9000 organized crime members was relatively small, with the largest sample accounting for just over 4000 members.

The studies selected different types of non‐organized‐crime comparison groups. These included general population samples, offenders in general, or serious offenders. While the choices were justified by the specific objectives of each study, the variety of comparison groups may affect the direction of the associations between risk factors and organized crime membership and the size of the estimates. While we had considered to conduct a subgroup analysis to investigate possible discrepancies across comparison groups, the paucity of effect sizes across different groups prevented us to do so.

Because of the above issues, we had to rely on raw, unadjusted measures, to extract useful information for our review. This may also explain the high degree of variability in most analyses, as the independent measures may be the result of different matching procedures, of comparison with different comparison groups, as well as different unaccounted confounders in the original studies.

### 6.4 Limitations and potential biases in the review process

The main limitations of this review were the limited number of predictors, the small number of studies within each factor category and subcategory, and the heterogeneity in the definition of organized crime group.

We have addressed the first main limitation by adopting a precautionary approach when classifying risk factors between predictors and correlates. We included factors among predictors only when they addressed time‐invariant factors (e.g., sex or ethnicity) or when the included studies were clearly measuring aspects before involvement into organized crime (e.g., ever convicted before recruitment into organized crime). Furthermore, we reported and analyzed the results of predictors and correlates separately.

Regarding the small number of studies by category and subcategory, this may increase the biases due to the studies' heterogeneity in objectives, sampling, matching, measurements. We thus suggest caution in interpreting the results, particularly considering that they refer to a small set of countries, mostly the US, the Netherlands, the UK, and Italy. However, most of the included studies are relatively recent. We thus expect that their number will further grow in the next years and that future updates of this review will be able to collect more data.

About the third main limitation (heterogeneity in the definition of organized crime), we acknowledged in the Background that this is a typical characteristic of this field of research, with varying definitions and perceptions of organized crime groups across time and space. For example, nearly half of the quantitative studies focused on US or UK adult gangs. The generalizability of the risk factors from this study to other types of organized crime groups may be scarce. In part, the heterogeneity of organized crime groups reflected in our results, with most meta‐analyses reporting a high level of heterogeneity. We attempted to address this by conducting a subgroup analysis by type of organized crime group (See Type of organized crime group as effect size moderator and Supporting Information Appendix E). In most cases, we found that heterogeneity among groups is statistically significant. This may suggest that part of the heterogeneity observed in the meta‐analyses may be due to the variability across organized crime groups. In turn, this may point to different associations between risk factors and types of organized crime groups. However, due to the small number of studies, most moderator analyses included only one independent estimate by type of organized crime group. Consequently, we warn about the weak evidence base supporting these analyses and we caution against drawing strong conclusion from them. Rather, we consider that they may indicate promising paths for future research comparing factors across different types of organized crime groups.

Furthermore, researchers are divided on the nature of some organized crime groups, particularly for the groups with a legitimate or quasi legitimate form such as motorcycle clubs. While the media and institutions often equate these organizations to criminal organizations, not all members may actively engage into criminal activities and especially organized crime activities (for recent contributions to this debate, see Lauchs, 2019; Morgan et al., 2020; Von Lampe & Blokland, 2020). The included studies adopted a variety of sampling strategies, and they never selected the samples merely on the formal membership of a specific group such as gangs or motorcycle club. In fact, they often relied on self‐nomination in surveys and interviews or police intelligence. We thus consider that the research included in this systematic review focused on individuals involved in organized crime activities. Nevertheless, the selected studies were rarely explicitly on this specific point.

### 6.5 Agreements and disagreements with other studies or reviews

As anticipated in the background, no other systematic review with meta‐analyses has examined the risk factors of recruitment into organized crime. After the publication of the protocol for this review, some authors of this review published a systematic review with narrative synthesis analyzing 47 quantitative, qualitative and mixed‐methods studies published until 2017 (Calderoni et al., 2020; Comunale et al., 2020). The narrative review provided a summary of the existing empirical evidence from the available literature but lacked any meta‐analysis and thus the capacity to establish the causal nature and relative importance of different risk factors. The findings emphasized the relevance of social relations, criminal background, and criminal skills as the most frequently discussed factors for recruitment into organized crime.

The results of this systematic review are only partially consistent with these findings. This is mostly due to the small number of independent estimates from quantitative studies falling into the main factor categories pointed out in the narrative synthesis. For example, while the latter argued the importance of social ties for the involvement into organized crime, our systematic review only retrieved one predictor and two correlates classified in the social relations category. While all independent estimates report a positive and statistically significant association with organized crime membership, the amount of evidence is weak and provides only partial support to the arguments of the narrative review.

## 7 AUTHORS' CONCLUSIONS

### 7.1 Implications for practice and policy

As we had anticipated in our protocol, our systematic review found mostly observational studies with a cross‐sectional design. We were able only to identify a minority of predictors of organized crime recruitment, whereas most available evidence is on correlates of membership of organized criminal groups. Given the amount and type of evidence collected, it is difficult to formulate detailed practical or policy implications. Nevertheless, we consider that our results may indicate promising directions for developing programs aiming at preventing recruitment into organized crime.

Within the small amount of evidence about predictors, we found relatively strong evidence that factors such as being male, prior criminal activity, and prior episodes of violence (including violent offences) are risk factors of future recruitment into organized criminal groups. We found weak evidence, although supported by several qualitative studies, narrative reviews, and the findings about correlates, regarding prior criminal sanctions, social relations with organized crime involved subjects, and a troubled family environment.

However, we warn that the evidence base extracted from the included studies is far from complete and it is likely that important risk factors have been overlooked by the existing literature. The inclusion of qualitative studies in this systematic review enabled to identify broad indications for potential risk factors. Yet, the lack of evidence from quantitative studies suggests that these potential drivers of recruitment into organized crime would require further exploration before being included in preventive approaches.

While several countries in the world have implemented various policies aiming at preventing the activities and crimes of organized criminal groups, these were often based on very limited evidence and even more rarely subject to evaluations. We consider that the evidence produced by this systematic review could offer some preliminary indications to practitioners and policy makers in developing strategies to prevent recruitment into organized crime.

### 7.2 Implications for research

Although we were able to include nineteen quantitative and thirty‐three qualitative studies, this systematic review showed that the available evidence about the factors leading to recruitment into organized crime is often incomplete and weak. However, many included studies were published in recent years, suggesting that this field of research is growing rapidly.

Attention to both quantitative and qualitative studies enabled us not only to inform and contextualize the evidence from quantitative works, but also to assess the completeness of evidence in the field. For some categories, we found that abundant analyses by qualitative research did not find a corresponding number of quantitative studies (particularly for factors in the legitimate jobs/skills, motivation, and social relations categories). Future quantitative studies may consider addressing risk factors falling within these categories, particularly due to the substantial amount of evidence from qualitative research. We acknowledge that it may be difficult to design studies comprising at least two groups (an organized crime group and a comparison group) and addressing issues such as social relation with organized crime members and motivations. However, few quantitative studies managed to include these factors and the small amount of evidence indicates positive and strong associations (including predictors). These studies were based on surveys, more likely to require greater resources than research based on criminal record registers. Furthermore, survey‐based studies analyzed relatively small samples (less than 200 organized crime members), which may affect the validity of the results.

All included quantitative studies adopted a cross‐sectional approach, with a few including retrospective data collection. Longitudinal designs could more effectively establish causal relations between risk factors and organized crime recruitment. However, conducting quantitative longitudinal research in the field of organized crime is particularly challenging, as the population targeted actively avoid attention by scholars and researchers. Retrospective data from included studies often concerned criminal activities, contact with the criminal justice system, and criminal sanctions derived from official crime records or police intelligence data. One possible, although challenging, direction to expand the number of possible predictors would be to link data from the criminal justice system with general population registers, which may provide additional retrospective information regarding for example parental income and education, individuals' wealth, income, education, and professional position.

## REFERENCES TO EXCLUDED STUDIES

This table lists each unique reference and the reason/s for exclusion.

Legend: 0 = The study does not meet the criterion; 1 = The study meets the criterion; 99 = Can't tell.

**Table:** 

Full citation		1 Report on OCGs?	2 Recruitment into OCGs?	3 Empirical contribution?	4 Well‐defined/single factors?	5 Individual level factors?	6 Variability OCG/non‐OCG?
001	Abram, K. M. (1989). The effect of co‐occurring disorders on criminal careers: Interaction of antisocial personality, alcoholism, and drug disorders. *International Journal of Law and Psychiatry, 12*(2–3), 133–148. https://doi.org/10.1016/0160-2527(89)90004-6	0	‐	‐	‐	‐	‐
002	Anderson, A. G. (1979). *The business of organized crime: A Cosa Nostra family*. Hoover Press.	1	0	‐	‐	‐	‐
003	Andrae, D., McIntosh, T., & Coster, S. (2017). Marginalised: An insider's view of the state, state policies in New Zealand and gang formation. *Critical Criminology, 25*(1), 119–135. https://doi.org/10.1007/s10612-016-9325-8	1	1	0	‐	‐	‐
004	Armstrong, T. A., & Britt, C. L. (2004). The effect of offender characteristics on offense specialization and escalation. *Justice Quarterly, 21*(4), 843–876. https://doi.org/10.1080/07418820400096011	0	‐	‐	‐	‐	‐
005	Atkinson‐Sheppard, S. (2017). ‘Mastaans’ and the market for social protection exploring mafia groups in Dhaka, Bangladesh. *Asian Journal of Criminology, 12*(4), 235–253. https://doi.org/10.1007/s11417-017-9246-9	1	1	1	1	0	‐
006	Beaver, K. M., & Barnes, J. C. (2012). Admission of drug‐selling behaviors is structured by genetic and nonshared environmental factors: Results from a longitudinal twin‐based study. *Addictive Behaviors, 37*(6), 697–702. https://doi.org/10.1016/j.addbeh.2012.02.005	0	‐	‐	‐	‐	‐
007	Behan, T. (1996). *The Camorra*. Routledge.	1	1	0	‐	‐	‐
008	Bendixen, M., Endresen, I. M., & Olweus, D. (2006). Joining and leaving gangs: Selection and facilitation effects on self‐reported antisocial behaviour in early adolescence. *European Journal of Criminology, 3*(1), 85–114. https://doi.org/10.1177/1477370806059082	0	‐	‐	‐	‐	‐
009	Benson, M. L., & Moore, E. (1992). Are White‐collar and common offenders the same? An empirical and theoretical critique of a recently proposed general theory of crime. *Journal of Research in Crime and Delinquency, 29*(3), 251–272. https://doi.org/10.1177/0022427892029003001	0	‐	‐	‐	‐	‐
010	Bijlenga, N., & Kleemans, E. R. (2018). Criminals seeking ICT‐expertise: An exploratory study of Dutch cases. *European Journal on Criminal Policy and Research, 24*(3), 253–268. https://doi.org/10.1007/s10610-017-9356-z	0	‐	‐	‐	‐	‐
011	Bjerregaard, B. (2010). Gang membership and drug involvement: Untangling the complex relationship. *Crime & Delinquency, 56*(1), 3–34. https://doi.org/10.1177/0011128707307217	0	‐	‐	‐	‐	‐
012	Bjerregaard, B. (2002). Self‐definitions of gang membership and involvement in delinquent activities. *Youth & Society, 34*(1), 31–54. https://doi.org/10.1177/0044118X02034001002	0	‐	‐	‐	‐	‐
013	Block, A. A. (1980). *East side, west side: Organizing crime in New York, 1930‐1950*. Transaction Publishers.	1	0	‐	‐	‐	‐
014	Blok, A. (1975). *The Mafia of a Sicilian Village, 1860‐1960: A study of violent peasant entrepreneurs*. Harper & Row.	1	1	1	0	‐	‐
015	Blokland, A., Leest, W. V. D., & Soudijn, M. (2020). Officially registered criminal careers of members of Dutch outlaw motorcycle gangs and their support clubs. *Deviant Behavior, 41*(11), 1393–1412. https://doi.org/10.1080/01639625.2019.1619422	1	9	1	9	1	0
016	Boudiaf, H. H. (2019). La religión y las nuevas tecnologías al servicio de las redes nigerianas de explotación sexual de niñas migrantes. *Sociología y tecnociencia: Revista digital de sociología del sistema tecnocientífico, 9*(1), 49–68.	1	1	0	‐	‐	‐
017	Bowker, L. H., & Klein, M. W. (1983). The etiology of female juvenile delinquency and gang membership: A test of psychological and social structural explanations. *Adolescence, 18*(72), 739–751.	0	‐	‐	‐	‐	‐
018	Brown, R., & Smith, R. G. (2018). Exploring the relationship between organised crime and volume crime. *Trends and Issues in Crime and Criminal Justice, 565*. aic.gov.au/publications/tandi/tandi565	1	0	‐	‐	‐	‐
019	Burt, C., Simons, R., & Simons, L. (2006). A longitudinal test of the effects of parenting and the stability of self‐control: Negative evidence for the General Theory of Crime. *Criminology, 44*, 353–396. https://doi.org/10.1111/j.1745-9125.2006.00052.x	0	‐	‐	‐	‐	‐
020	Campedelli, G. M., Calderoni, F., Comunale, T., & Meneghini, C. (2021). Life‐course criminal trajectories of mafia members. *Crime & Delinquency, 67*(1), 111. https://doi.org/10.1177/0011128719860834	1	0	‐	‐	‐	‐
021	Castiello, M., Mosca, M., & Villani, S. (2018). The gentle use of violence and the human capital recruitment strategies of the Casalesi Clan. In M. Massari, V. Martone, & B. Waterhouse, *Mafia violence. Political, symbolic, and economic forms of violence in Camorra Clans* (1st ed., pp. 174–194). Routledge. https://doi.org/10.4324/9780429467554-10	1	0	‐	‐	‐	‐
022	Catino, M. (2019). *Mafia organizations: The visible hand of criminal enterprise*. Cambridge University Press.	1	1	0	‐	‐	‐
023	Cepeda, A., Valdez, A., & Nowotny, K. M. (2016). Childhood trauma among Mexican american gang members and delinquent youth: A comparative exploratory study. *Child Abuse Review, 25*(3), 205–217. https://doi.org/10.1002/car.2340	0	‐	‐	‐	‐	‐
024	Chapsos, I., & Hamilton, S. (2019). Illegal fishing and fisheries crime as a transnational organized crime in Indonesia. *Trends in Organized Crime, 22*(3), 255–273. https://doi.org/10.1007/s12117-018-9329-8	1	0	‐	‐	‐	‐
025	Charette, Y., & Papachristos, A. V. (2017). The network dynamics of co‐offending careers. *Social Networks, 51*, 3–13. https://doi.org/10.1016/j.socnet.2016.12.005	0	‐	‐	‐	‐	‐
026	Chui, W. H., & Khiatani, P. V. (2018). Delinquency among members of Hong Kong youth street gangs: The role of the organizational structures of gangs and triad affiliations. *International Journal of Offender Therapy and Comparative Criminology, 62*(9), 2527–2547. https://doi.org/10.1177/0306624X17730616	0	‐	‐	‐	‐	‐
027	Ciconte, E. (1992). '*Ndrangheta dall'Unità a oggi*. Laterza.	1	1	1	0	‐	‐
028	Craparo, G., David, V., Costanzo, G., & Gori, A. (2018). Cosa Nostra and the Camorra: Assessment of personality, alexithymic traits, and attachment styles. *International Journal of Law and Psychiatry, 58*, 17–26. https://doi.org/10.1016/j.ijlp.2018.02.010	1	1	1	1	1	0
029	Crocker, R., Webb, S., Skidmore, M., Garner, S., Gill, M., & Graham, J. (2019). Tackling local organised crime groups: Lessons from research intwo UK cities. *Trends in Organized Crime, 22*(4), 433–449. https://doi.org/10.1007/s12117-018-9335-x	1	0	‐	‐	‐	‐
030	Cureton, S. R. (2017). CHIRAQ: Oppression, homicide, concentrated misery, and gangsterism in Chicago. *Journal of Gang Research, 25*(1), 1–18.	1	1	0	‐	‐	‐
031	Decker, S. H., Katz, C. M., & Webb, V. J. (2008). Understanding the black box of gang organization: Implications for involvement in violent crime, drug sales, and violent victimization. *Crime & Delinquency, 54*(1), 153–172. https://doi.org/10.1177/0011128706296664	0	‐	‐	‐	‐	‐
032	DeLisi, M., Berg, M. T., & Hochstetler, A. (2004). Gang members, career criminals and prison violence: Further specification of the importation model of inmate behavior. *Criminal Justice Studies, 17*(4), 369–383. https://doi.org/10.1080/1478601042000314883	0	‐	‐	‐	‐	‐
033	DeLisi, M., Barnes, J. C., Beaver, K. M., & Gibson, C. L. (2009). Delinquent gangs and adolescent victimization revisited: A propensity score matching approach. *Criminal Justice and Behavior, 36*(8), 808–823. https://doi.org/10.1177/0093854809337703	0	‐	‐	‐	‐	‐
034	Douglas, K., & Smith, R. G. (2018). Disengagement from involvement in organised crime: Processes and risks. *Trends and Issues in Crime and Criminal Justice, 542*, 1–15.	1	0	‐	‐	‐	‐
035	Drury, L., & Travaglino, G. A. (2020). Demobilising by legitimising: Masculine honour, positive and negative contact, and social activism against criminal organisations. *Group Processes & Intergroup Relations, 23*(3), 402–417. https://doi.org/10.1177/1368430219842917	9	0	‐	‐	‐	‐
036	Eidson, J. L., Roman, C. G., & Cahill, M. (2017). Successes and challenges in recruiting and retaining gang members in longitudinal research: Lessons Learned from a multisite social network study. *Youth Violence and Juvenile Justice, 15*(4), 396–418. https://doi.org/10.1177/1541204016657395	0	‐	‐	‐	‐	‐
037	Eitle, D., Gunkel, S., & Van Gundy, K. (2004). Cumulative exposure to stressful life events and male gang membership. *Journal of Criminal Justice, 32*(2), 95–111. https://doi.org/10.1016/j.jcrimjus.2003.12.001	0	‐	‐	‐	‐	‐
038	Esbensen, F.‐A., & Huizinga, D. (1993). Gangs, drugs, and delinquency in a survey of urban youth. *Criminology, 31*(4), 565–589. https://doi.org/10.1111/j.1745-9125.1993.tb01142.x	0	‐	‐	‐	‐	‐
039	Esbensen, F.‐A., Huizinga, D., & Weiher, A. W. (1993). Gang and non‐gang youth: Differences in explanatory factors. *Journal of Contemporary Criminal Justice, 9*(2), 94–116. https://doi.org/10.1177/104398629300900203	0	‐	‐	‐	‐	‐
040	Evans, T. D., Cullen, F. T., Burton, V. S., Dunaway, R. G., & Benson, M. L. (1997). The social consequences of self‐control: Testing the general theory of crime*. *Criminology, 35*(3), 475–504. https://doi.org/10.1111/j.1745-9125.1997.tb01226.x	0	‐	‐	‐	‐	‐
041	Fijnaut, C., Bovenkerk, F., Bruinsma, G., & Van de Bunt, H. G. (1998). *Organized crime in the Netherlands*. Martinus Nijhoff Publishers.	1	0	‐	‐	‐	‐
042	Francis, B., Soothill, K., & Fligelstone, R. (2004). Identifying patterns and pathways of offending behaviour: A new approach to typologies of crime. *European Journal of Criminology, 1*(1), 48–87. https://doi.org/10.1177/1477370804038707	0	‐	‐	‐	‐	‐
043	Freeman, D., McManus, S., Brugha, T., Meltzer, H., Jenkins, R., & Bebbington, P. (2011). Concomitants of paranoia in the general population. *Psychological Medicine, 41*(5), 923–936. https://doi.org/10.1017/S0033291710001546	0	‐	‐	‐	‐	‐
044	García, M. M. (2006). ‘Narcoballads’: The psychology and recruitment process of the ‘Narco.’ *Global Crime, 7*(2), 200–213. https://doi.org/10.1080/17440570601014461	1	0	‐	‐	‐	‐
045	Garcia, N. M. (2017). *The dark side of social media: The case of the Mexican drug war* [Ph.D. Dissertation, University of Miami]. scholarship.miami.edu/discovery/fulldisplay/alma991031447170702976/01UOML_INST:ResearchRepository	1	0	‐	‐	‐	‐
046	Gibbs, J. T. (2000). Gangs as alternative transitional structures. *Journal of Multicultural Social Work, 8*(1–2), 71–99. https://doi.org/10.1300/J285v08n01_04	1	1	1	1	1	0
047	Giménez‐Salinas Framis, A., Requena Espada, L., & De la Corte Ibáñez, L. (2011). ¿Existe un perfil de delincuente organizado?: Exploración a partir de una muestra española. *Revista electrónica de ciencia penal y criminología, 13*, 3.	1	0	‐	‐	‐	‐
048	Gordon, R. A., Lahey, B. B., Kawai, E., Loeber, R., Stouthamer‐Loeber, M., & Farrington, D. P. (2004). Antisocial behavior and youth gang membership: Selection and socialization. *Criminology, 42*(1), 55–88. https://doi.org/10.1111/j.1745-9125.2004.tb00513.x	0	‐	‐	‐	‐	‐
049	Hagedorn, J. M. (2017). Gangs, schools, and social change: An institutional analysis. *The ANNALS of the American Academy of Political and Social Science, 673*(1), 190–208. https://doi.org/10.1177/0002716217726965	0	‐	‐	‐	‐	‐
050	Hennigan, K. M., Maxson, C. L., Sloane, D. C., Kolnick, K. A., & Vindel, F. (2014). Identifying high‐risk youth for secondary gang prevention. *Journal of Crime and Justice, 37*(1), 104–128. https://doi.org/10.1080/0735648X.2013.831208	0	‐	‐	‐	‐	‐
051	Holt, T. J., & Bossler, A. M. (2008). Examining the applicability of lifestyle‐routine activities theory for cybercrime victimization. *Deviant Behavior, 30*(1), 1–25. https://doi.org/10.1080/01639620701876577	0	‐	‐	‐	‐	‐
052	Huff, C. R. (1998). *Comparing the criminal behavior of youth gangs and at‐risk youths. Research in Brief* (No. 172852; NIJ Research in Brief, p. 8). National Institute of Justice of the U.S. Department of Justice. www.ncjrs.gov/pdffiles/172852.pdf	0	‐	‐	‐	‐	‐
053	Hutchings, A. (2014). Crime from the keyboard: Organised cybercrime, co‐offending, initiation and knowledge transmission. *Crime, Law and Social Change, 62*(1), 1–20. https://doi.org/10.1007/s10611-014-9520-z	1	0	‐	‐	‐	‐
054	Ianni, F. A. J. (1973). *Ethnic succession in organized crime: Summary report*. U.S. Department of Justice, Law Enforcement Assistance Administration, National Institute of Law Enforcement and Criminal Justice.	1	1	1	1	0	‐
055	Ianni, F. A. J. (1974). *Black mafia: Ethnic succession in organized crime*. Simon & Schuster.	1	1	1	1	0	‐
056	Jahnsen, S. Ø. (2018). Scandinavian approaches to outlaw motorcycle gangs. *Trends and Issues in Crime and Criminal Justice, 543*, 1–15.	1	0	‐	‐	‐	‐
057	Jhi, K. Y., & Gerber, J. (2015). Texan gangs in “Da Hood”: The impact of actual and perceptual neighborhood qualities on gang membership | Semantic Scholar. *Justice Policy Journal, 12*(2). www.cjcj.org/uploads/cjcj/documents/jpj_texan_gangs_fall_2015.pdf	0	‐	‐	‐	‐	‐
058	Katz, C. M., Webb, V. J., Fox, K., & Shaffer, J. N. (2011). Understanding the relationship between violent victimization and gang membership. *Journal of Criminal Justice, 39*(1), 48–59. https://doi.org/10.1016/j.jcrimjus.2010.10.004	0	‐	‐	‐	‐	‐
059	Katz, C. M., Webb, V. J., & Decker, S. H. (2005). Using the Arrestee Drug Abuse Monitoring (ADAM) program to further understand the relationship between drug use and gang membership. *Justice Quarterly, 22*(1), 58–88. https://doi.org/10.1080/0741882042000333645	0	‐	‐	‐	‐	‐
060	Kerig, P. K., Chaplo, S. D., Bennett, D. C., & Modrowski, C. A. (2016). “Harm as harm”: Gang membership, perpetration trauma, and posttraumatic stress symptoms among youth in the juvenile justice system. *Criminal Justice and Behavior, 43*(5), 635–652. https://doi.org/10.1177/0093854815607307	0	‐	‐	‐	‐	‐
061	King, J. E., Walpole, C. E., & Lamon, K. (2007). Surf and turf wars online—Growing implications of internet gang violence. *Journal of Adolescent Health, 41*(6, Supplement), S66–S68. https://doi.org/10.1016/j.jadohealth.2007.09.001	1	1	0	‐	‐	‐
062	Kleemans, E. R. (2013). Organized crime and the visible hand: A theoretical critique on the economic analysis of organized crime. *Criminology & Criminal Justice, 13*(5), 615–629. https://doi.org/10.1177/1748895812465296	1	1	0	‐	‐	‐
063	Klein, M. W., Weerman, F. M., & Thornberry, T. P. (2006). Street gang violence in Europe. *European Journal of Criminology, 3*(4), 413–437. https://doi.org/10.1177/1477370806067911	1	1	0	‐	‐	‐
064	Klein, M. W., & Maxson, C. L. (2006). Street gang patterns and policies. In *Street gang patterns and policies*. Oxford University Press. oxford.universitypressscholarship.com/view/10.1093/acprof:oso/9780195163445.001.0001/acprof‐9780195163445	1	1	0	‐	‐	‐
065	Klement, C. (2016). Crime prevalence and frequency among Danish outlaw bikers. *Journal of Scandinavian Studies in Criminology and Crime Prevention, 17*(2), 131–149. https://doi.org/10.1080/14043858.2016.1240420	1	1	1	1	1	0
066	Knox, G. W. (n.d.). *Gang members on Facebook: Should we look the other way? A special report of the National Gang Crime Research Center*. National Gang Crime Research Center. Retrieved February 5, 2021, from https://www.ngcrc.com/gangface.html	1	1	0	‐	‐	‐
067	Koivu, K. L. (2018). Illicit partners and political development: How organized crime made the state. *Studies in Comparative International Development, 53*(1), 47–66. https://doi.org/10.1007/s12116-017-9242-1	1	0	‐	‐	‐	‐
068	Lacourse, E., Nagin, D., Tremblay, R. E., Vitaro, F., & Claes, M. (2003). Developmental trajectories of boys' delinquent group membership and facilitation of violent behaviors during adolescence. *Development and Psychopathology, 15*(1), 183–197. https://doi.org/10.1017/s0954579403000105	0	‐	‐	‐	‐	‐
069	Langhorn, M. (2018). Human trafficking and sexual servitude: Organised crime's involvement in Australia. *Salus Journal, 6*(1). salusjournal.com/wp‐content/uploads/2018/03/Langhorn_Salus_Journal_Volume_6_Number_1_2018_pp_1‐25. pdf	1	0	‐	‐	‐	‐
070	Lauchs, M., & Staines, Z. (2019). An analysis of outlaw motorcycle gang crime: Are bikers organised criminals? *Global Crime, 20*(2), 69–89. https://doi.org/10.1080/17440572.2019.1583107	1	0	‐	‐	‐	‐
071	Leukfeldt, E. R. (2014). Cybercrime and social ties. *Trends in Organized Crime, 17*(4), 231–249. https://doi.org/10.1007/s12117-014-9229-5	1	0	‐	‐	‐	‐
072	Levi, M. (2008). Organized fraud and organizing frauds: Unpacking research on networks and organization. *Criminology & Criminal Justice, 8*(4), 389–419. https://doi.org/10.1177/1748895808096470	1	1	0	‐	‐	‐
073	Levitt, S. D., & Venkatesh, S. A. (2000). An economic analysis of a drug‐selling gang's finances. *Quarterly Journal of Economics, 115*, 755–789. https://doi.org/10.3386/w6592	0	‐	‐	‐	‐	‐
074	Lo, T. W. (2010). Beyond social capital: Triad organized crime in Hong Kong and China. *The British Journal of Criminology, 50*(5), 851–872. https://doi.org/10.1093/bjc/azq022	1	0	‐	‐	‐	‐
075	Lo Verso, G., & Lo Coco, G. (2004). Working with patients involved in the mafia: Considerations from Italian psychotherapy experiences. *Psychoanalytic Psychology, 21*(2), 171–182. https://doi.org/10.1037/0736-9735.21.2.171	1	0	‐	‐	‐	‐
076	Lochner, L., & Moretti, E. (2004). The effect of education on crime: Evidence from prison inmates, arrests, and self‐reports. *American Economic Review, 94*(1), 155–189. https://doi.org/10.1257/000282804322970751	0	‐	‐	‐	‐	‐
077	Lochner, L. (2011). Education policy and crime. In P. Cook, J. Ludwig, & J. McCrary (Eds.), *Controlling crime: Strategies and tradeoffs* (p. 634). University Of Chicago Press. https://doi.org/10.3386/w15894	0	‐	‐	‐	‐	‐
078	Luong, H. T. (2017). *Transnational narcotics trafficking across Vietnam borderland and Lao People's Democratic Republic* [PhD dissertation, RMIT University]. Retrieved February 5, 2021, from https://www.grin.com/document/366349	1	1	1	1	1	0
079	Lupo, S. (2016). *Storia della mafia: La criminalità organizzata in Sicilia dalle origini ai giorni nostri*. Donzelli Editore.	1	0	‐	‐	‐	‐
080	MacNeil, G., Stewart, J. C., & Kaufman, A. V. (2000). Social support as a potential moderator of adolescent delinquent behaviors. *Child and Adolescent Social Work Journal, 17*(5), 361–379. https://doi.org/10.1023/A:1007555014397	0	‐	‐	‐	‐	‐
081	Martinez‐Vaquero, L. A., Dolci, V., & Trianni, V. (2019). Evolutionary dynamics of organised crime and terrorist networks. *Scientific Reports, 9*(1), 9727. https://doi.org/10.1038/s41598-019-46141-8	1	0	‐	‐	‐	‐
082	McDaniel, D. D. (2012). Risk and protective factors associated with gang affiliation among high‐risk youth: A public health approach. *Injury Prevention, 18*(4), 253–258. https://doi.org/10.1136/injuryprev-2011-040083	0	‐	‐	‐	‐	‐
083	McFarlane, H. J. (2016). *An agent based model of community authority structure resilience* [PhD dissertation, George Mason University]. http://mars.gmu.edu/handle/1920/10438	0	‐	‐	‐	‐	‐
084	Mcgloin, J. M., Schreck, C. J., Stewart, E. A., & Ousey, G. C. (2011). Predicting the violent offender: The discriminant validity of the subculture of violence. *Criminology, 49*(3), 767–794. https://doi.org/10.1111/j.1745-9125.2011.00235.x	0	‐	‐	‐	‐	‐
085	Medina, J., Aldridge, J., Shute, J., & Ross, A. (2013). Measuring gang membership in England and Wales: A latent class analysis with Eurogang survey questions. *European Journal of Criminology, 10*(5), 591–605. https://doi.org/10.1177/1477370813475393	0	‐	‐	‐	‐	‐
086	Melde, C., & Esbensen, F.‐A. (2011). Gang membership as a turning point in the life course*. *Criminology, 49*(2), 513–552. https://doi.org/10.1111/j.1745-9125.2011.00227.x	0	‐	‐	‐	‐	‐
087	Miller, W. B. (2011). *City gangs*. https://live-crim.ws.asu.edu/sites/default/files/%5Bterm%3Aname%5D/%5Bnode%3Acreate%3Acustom%3AYm%5D/city-gangs-book.pdf	0	‐	‐	‐	‐	‐
088	Moore, J. W. (1990). *Gangs, drugs, and violence*. NIDA Research Monograph. https://pubmed.ncbi.nlm.nih.gov/2096286/	1	1	0	‐	‐	‐
089	Morselli, C. (2003). Career opportunities and network‐based privileges in the Cosa Nostra. *Crime, Law and Social Change, 39*(4), 383–418. https://doi.org/10.1023/A:1024020609694	1	0	‐	‐	‐	‐
090	Morselli, C., Tremblay, P., & Mccarthy, B. (2006). Mentors and criminal achievement*. *Criminology, 44*(1), 17–43. https://doi.org/10.1111/j.1745-9125.2006.00041.x	1	1	1	1	1	0
091	Ngo, H. V., Calhoun, A., Worthington, C., Pyrch, T., & Este, D. (2017). The unravelling of identities and belonging: Criminal gang involvement of youth from immigrant families. *Journal of International Migration and Integration, 18*(1), 63–84. https://doi.org/10.1007/s12134-015-0466-5	0	‐	‐	‐	‐	‐
092	Pedersen, M. L. (2014). Gang joining in Denmark: Prevalence and correlates of street gang membership. *Journal of Scandinavian Studies in Criminology and Crime Prevention, 15*(1), 55–72. https://doi.org/10.1080/14043858.2014.886892	0	‐	‐	‐	‐	‐
093	Petta, D. L. (2017). The triads and the secret societies in China: Between myth and demystification. *Revista Brasileira de Ciências Sociais, 32*(93). https://doi.org/10.17666/329309/2017	1	0	‐	‐	‐	‐
094	Pyrooz, D. C. (2014). “From your first cigarette to your last dyin' day”: The patterning of gang membership in the life‐course. *Journal of Quantitative Criminology, 30*(2), 349–372. https://doi.org/10.1007/s10940-013-9206-1	1	1	1	1	1	0
095	Pyrooz, D. C., & Decker, S. H. (2013). Delinquent behavior, violence, and gang involvement in China. *Journal of Quantitative Criminology, 29*(2), 251–272. https://doi.org/10.1007/s10940-012-9178-6	0	‐	‐	‐	‐	‐
096	Pyrooz, D. C., & Densley, J. A. (2016). Selection into street gangs: Signaling theory, gang membership, and criminal offending. *Journal of Research in Crime and Delinquency, 53*(4), 447–481. https://doi.org/10.1177/0022427815619462	0	‐	‐	‐	‐	‐
097	Quinn, K., Pacella, M. L., Dickson‐Gomez, J., & Nydegger, L. A. (2017). Childhood adversity and the continued exposure to trauma and violence among adolescent gang members. *American Journal of Community Psychology, 59*(1–2), 36–49. https://doi.org/10.1002/ajcp.12123	0	‐	‐	‐	‐	‐
098	Ralph, L. (2014). *Renegade dreams: Living through Injury in Gangland Chicago*. University of Chicago Press.	0	‐	‐	‐	‐	‐
099	Ramirez, E. B. (2016). *Mentorship in commercial domestic cannabis cultivation* [M.Sc. dissertation, California State University]. https://search.proquest.com/openview/9f9dcfa44a4c60602977a9df44cc586c/1?pq-origsite=gscholar%26cbl=18750%26diss=y	0	‐	‐	‐	‐	‐
100	Requena, L., De Juan, M., Giménez‐Salinas, A., & De la Corte, L. (2014). A psychosocial study on crime and gender: Position, role and status of women in a sample of Spanish criminal organizations. *International Journal of Social Psychology, 29*(1), 121–149. https://doi.org/10.1080/02134748.2013.878572	1	1	1	1	1	0
101	Rodrigues, T., Kalil, M., Zepeda, R., & Rosen, J. D. (2017). War Zone Acapulco: Urban drug trafficking in the Americas. *Contexto Internacional, 39*(3), 609–631. https://doi.org/10.1590/s0102-8529.2017390300008	1	0	‐	‐	‐	‐
102	Rosenbaum, J. E. (1995). Changing the geography of opportunity by expanding residential choice: Lessons from the Gautreaux program. *Housing Policy Debate, 6*(1), 231–269. https://doi.org/10.1080/10511482.1995.9521186	0	‐	‐	‐	‐	‐
103	Smith, R. G. (2014). Responding to organised crime through intervention in recruitment pathways. *Trends & Issues in Crime and Criminal Justice*, 473. aic.gov.au/publications/tandi/tandi473	1	1	0	‐	‐	‐
104	Sales, I. (2015). *Storia dell'Italia mafiosa: Perché le mafie hanno avuto successo*. Rubbettino.	1	0	‐	‐	‐	‐
105	Giménez‐Salinas, A., & Fernandez Regadera, S. (2016). Multiple affiliations in criminal organizations: Analysis of a Spanish sample. *Crime, Law and Social Change, 65*(1). https://doi.org/10.1007/s10611-015-9597-z	1	1	1	1	1	0
106	Sergi, A. (2016). A qualitative reading of the ecological (dis)organisation of criminal associations. The case of the ‘Famiglia Basilischi’ in Italy. *Trends in Organized Crime, 19*(2), 149–174.	1	0	‐	‐	‐	‐
107	Sergi, A. (2018). Widening the Antimafia Net: Child protection and the socio‐cultural transmission of mafia behaviours in Calabria. *Youth Justice, 18*(2), 149–168. https://doi.org/10.1177/1473225418791420	1	0	‐	‐	‐	‐
108	Silverman, I. J., & Dinitz, S. (1974). Compulsive masculinity and delinquency: An empirical investigation. *Criminology, 11*(4), 498–515. https://doi.org/10.1111/j.1745-9125.1974.tb00610.x	0	‐	‐	‐	‐	‐
109	Skinner, W. F., & Fream, A. M. (1997). A social learning theory analysis of computer crime among college students. *Journal of Research in Crime and Delinquency, 34*(4), 495–518. https://doi.org/10.1177/0022427897034004005	0	‐	‐	‐	‐	‐
110	Smith, R. G. (2014). Responding to organised crime through intervention in recruitment pathways. *Trends & Issues in Crime and Criminal Justic*e, 473. https://www.aic.gov.au/publications/tandi/tandi473	1	1	0	‐	‐	‐
111	Smithson, H., Ralphs, R., & Williams, P. (2013). Used and abused: The problematic usage of gang terminology in the United Kingdom and its implications for ethnic minority youth. *The British Journal of Criminology, 53*(1), 113–128.	0	‐	‐	‐	‐	‐
112	Soothill, K., Francis, B., Ackerley, E., & Humphreys, L. (2008). Changing patterns of offending behaviour among young adults. *The British Journal of Criminology, 48*(1), 75–95.	0	‐	‐	‐	‐	‐
113	Sullivan, C. J., McGloin, J. M., Ray, J. V., & Caudy, M. S. (2009). Detecting specialization in offending: Comparing Analytic Approaches. *Journal of Quantitative Criminology, 25*(4), 419–441. https://doi.org/10.1007/s10940-009-9074-x	0	‐	‐	‐	‐	‐
114	Sullivan, C. J., Mcgloin, J. M., Pratt, T. C., & Piquero, A. R. (2006). Rethinking the “norm” of offender generality: Investigating specialization in the short‐term. *Criminology, 44*(1), 199–233. https://doi.org/10.1111/j.1745-9125.2006.00047.x	0	‐	‐	‐	‐	‐
115	Tapia, M. (2017). *The Barrio Gangs of San Antonio, 1915‐2015*. Texas Christian University Press.	0	‐	‐	‐	‐	‐
116	Taylor, M., & Potter, G. R. (2013). From “social supply” to “real dealing”: Drift, friendship, and trust in drug‐dealing careers. *Journal of Drug Issues, 43*(4), 392–406. https://doi.org/10.1177/0022042612474974	0	‐	‐	‐	‐	‐
117	Thornberry, T. P., Lizotte, A. J., Krohn, M. D., Farnworth, M., & Jang, S. J. (1994). Delinquent peers, beliefs, and delinquent behavior: A longitudinal test of interactional theory. *Criminology, 32*(1), 47–83. https://doi.org/10.1111/j.1745-9125.1994.tb01146.x	0	‐	‐	‐	‐	‐
118	Thornberry, T. P., & Krohn, M. D. (Eds.). (2003). *Taking stock of delinquency: An overview of findings from contemporary longitudinal studies*. Springer US. https://doi.org/10.1007/b105384	1	1	0	‐	‐	‐
119	Thornberry, T. P., Krohn, M. D., Lizotte, A. J., & Chard‐Wierschem, D. (1993). The role of juvenile gangs in facilitating delinquent behavior. *Journal of Research in Crime and Delinquency, 30*(1), 55–87. https://doi.org/10.1177/0022427893030001005	0	‐	‐	‐	‐	‐
120	Thrasher, F. M. (1927). The gang: A study of 1,313 gangs in Chicago (Abridged edition). University Of Chicago Press.	0	‐	‐	‐	‐	‐
121	Tolle, H. (2017). Gang affiliation as a measure of social structure in social structure social learning theory. *Deviant Behavior, 38*(8), 870–878. https://doi.org/10.1080/01639625.2016.1206712	0	‐	‐	‐	‐	‐
122	U.K. Government. (2011). *Ending gang and youth violence: A cross‐government report including further evidence and good practice case studies* (p. 84) [Policy paper]. U.K. Government, Violent and Youth Crime Prevention Unit. https://www.gov.uk/government/publications/ending-gang-and-youth-violence-cross-government-report	0	‐	‐	‐	‐	‐
123	Ullrich, S., Deasy, D., Smith, J., Johnson, B., Clarke, M., Broughton, N., & Coid, J. (2008). Detecting personality disorders in the prison population of England and Wales: Comparing case identification using the SCID‐II screen and the SCID‐II clinical interview. *The Journal of Forensic Psychiatry & Psychology, 19*(3), 301–322. https://doi.org/10.1080/14789940802045182	0	‐	‐	‐	‐	‐
124	Unlu, A., & Ekici, B. (2012). The extent to which demographic characteristics determine international drug couriers' profiles: A cross‐sectional study in Istanbul. *Trends in Organized Crime, 15*(4), 296–312. https://doi.org/10.1007/s12117-012-9152-6	1	1	1	1	1	0
125	Valdez, A., Kaplan, C. D., & Codina, E. (2000). Psychopathy among Mexican American gang members: A comparative study. *International Journal of Offender Therapy and Comparative Criminology, 44*(1), 46–58. https://doi.org/10.1177/0306624X00441005	0	‐	‐	‐	‐	‐
126	van de Ven, K. (2015). *The formation and development of illicit performance and image enhancing drug markets: Exploring supply and demand, and control policies in Belgium and the Netherlands* [PhD dissertation, University of Kent]. https://kar.kent.ac.uk/54398/	0	‐	‐	‐	‐	‐
127	van de Ven, K., & Mulrooney, K. J. D. (2017). Social suppliers: Exploring the cultural contours of the performance and image enhancing drug (PIED) market among bodybuilders in the Netherlands and Belgium. *The International Journal on Drug Policy, 40*, 6–15. https://doi.org/10.1016/j.drugpo.2016.07.009	0	‐	‐	‐	‐	‐
128	van Dijk, M., Kleemans, E., & Eichelsheim, V. (unpublished). Intergenerational continuity of crime among children of organized crime offenders in the Netherlands.	1	1	1	1	1	0
129	Varese, F. (2001). *The Russian mafia: Private protection in a new market economy* (1st ed. edizione). OUP Oxford.	1	0	‐	‐	‐	‐
130	Varese, F. (2006). How mafias migrate: The case of the 'Ndrangheta in Northern Italy. *Law & Society Review, 40*(2), 411–444.	1	0	‐	‐	‐	‐
131	Varese, F. (2011). How mafias take advantage of globalization: The Russian mafia in Italy. *British Journal of Criminology, 52*, 235–253. https://doi.org/10.1093/bjc/azr077	1	0	‐	‐	‐	‐
132	Varese, F. (2012). The structure and the content of criminal connections: The Russian mafia in Italy. *European Sociological Review, 29*, 899–909. https://doi.org/10.1093/esr/jcs067	1	0	‐	‐	‐	‐
133	Viuhko, M. (2018). Hardened professional criminals, or just friends and relatives? The diversity of offenders in human trafficking. *International Journal of Comparative and Applied Criminal Justice, 42*(2–3), 177–193. https://doi.org/10.1080/01924036.2017.1391106	9	0	‐	‐	‐	‐
134	Vuk, M. (2017). Parenting styles and gang membership: Mediating factors. *Deviant Behavior, 38*(4), 406–425. https://doi.org/10.1080/01639625.2016.1197011	0	‐	‐	‐	‐	‐
135	Wang, W. (2013). *Conventional capital, criminal capital, and criminal careers in drug trafficking* [PhD dissertation, Simon Fraser University]. http://summit.sfu.ca/item/13633	1	1	1	1	1	0
136	Watson, J. M. (1980). Outlaw motorcyclists: An outgrowth of lower class cultural concerns. *Deviant Behavior, 2*(1), 31–48. https://doi.org/10.1080/01639625.1980.9967541	1	0	‐	‐	‐	‐
137	Webb, V. J., Katz, C. M., & Decker, S. H. (2006). Assessing the validity of self‐reports by gang members: Results from the Arrestee Drug Abuse Monitoring Program. *Crime & Delinquency, 52*(2), 232–252. https://doi.org/10.1177/0011128705277972	0	‐	‐	‐	‐	‐
138	Weisburd, D., Chayet, E. F., & Waring, E. J. (1990). White‐collar crime and criminal careers: Some preliminary findings. *Crime & Delinquency, 36*(3), 342–355. https://doi.org/10.1177/0011128790036003003	0	‐	‐	‐	‐	‐
139	Williams, R. K., & Arnold, B. L. (2002). Offense specialization among serious habitual juvenile offenders in a Canadian city during the early stages of criminal careers. *International Criminal Justice Review, 12*(1), 1–21. https://doi.org/10.1177/105756770201200101	0	‐	‐	‐	‐	‐
140	Winfree, L. T., Taylor, T. J., He, N., & Esbensen, F.‐A. (2006). Self‐control and variability over time: Multivariate results using a 5‐year, multisite panel of youths. *Crime & Delinquency, 52*(2), 253–286. https://doi.org/10.1177/0011128705278012	0	‐	‐	‐	‐	‐
141	Wood, J. L. (2014). Understanding gang membership: The significance of group processes. *Group Processes & Intergroup Relations, 17*(6), 710–729. https://doi.org/10.1177/1368430214550344	1	1	0	‐	‐	‐
142	Wood, J. L., Alleyne, E., Mozova, K., & James, M. (2014). Predicting involvement in prison gang activity: Street gang membership, social and psychological factors. *Law and Human Behavior, 38*(3), 203–211. https://doi.org/10.1037/lhb0000053	0	‐	‐	‐	‐	‐
143	Wood, J., & Dennard, S. (2017). Gang membership: Links to violence exposure, paranoia, PTSD, anxiety, and forced control of behavior in prison. *Psychiatry, 80*(1), 30–41. https://doi.org/10.1080/00332747.2016.1199185	0	‐	‐	‐	‐	‐
144	Zapata, G. B. (2017). Aproximación a la narcocultura como referente de la construcción identitaria de jóvenes en México. *El Cotidiano, 206*, 59–67.	1	1	0	‐	‐	‐

## ROLES AND RESPONSIBILITIES

- Content: Francesco Calderoni, Tommaso Comunale, Gian Maria Campedelli, Niccolò Frualdo, Deborah Manzi, and Martina Marchesi
- Systematic review methods: Francesco Calderoni, Tommaso Comunale, Gian Maria Campedelli, and Martina Marchesi
- Statistical analysis: Francesco Calderoni, Tommaso Comunale, and Niccolò Frualdo
- Information retrieval: Tommaso Comunale, Gian Maria Campedelli, Deborah Manzi, and Martina Marchesi

## SOURCES OF SUPPORT

The early stages of this review received financial support by project PROTON (Modelling the PRocesses leading to OC and TerrOrist Networks), a European Commission funded project within the Horizon 2020 programme (Grant Agreement: 699824).

## DECLARATIONS OF INTEREST

None of the authors has previously been involved in relevant interventions or has published other reviews on the topic.

## PLANS FOR UPDATING THE REVIEW

The authors plan to update the review every 5 years.

## Supporting information

Supporting information.

## Footnotes

1 Developmental and life‐course criminology, term coined by Farrington (2003), is concerned with key factors for offending, effects of life events and life transitions on offending and development of offending.

2 We obtained temporary access to two specific sub‐databases: the National Criminal Justice Reference Service (NJCRS) and the Latin America & Iberia Database.

3 We excluded studies in Dutch since multiple contacts with Dutch scholars confirmed that most of the studies published in Dutch are also indexed and published in English.

4 The experts that contributed to this systematic review are: Jay Albanese (Virginia Commonwealth University, USA), Paolo Campana (University of Cambridge, UK), Scott Decker (Arizona State University, USA), Edward Kleemans (Vrije University of Amsterdam, NL), Klaus Von Lampe (Berlin School of Economics and Law, DE), Carlo Morselli (University of Montreal, CA), Letizia Paoli (Katholieke Universiteit Leuven, BE), David Pyrooz (University of Colorado Boulder, USA), Sonja Wolf (Centro de Investigación y Docencia Económicas, MEX).

5 The Covidence platform is a core component of Cochrane's review production toolkit improving the production of systematic reviews. For more information, see https://www.covidence.org/about-us.

6 Overall, divergent screening decisions remained below 10% of all screened documents.

7 Additional trainings were held to ensure consistency in performing full‐text screening among reviewers.

8 This estimate method allows to specify different values of correlation between effect sizes (i.e., *rho* option). In line with methodological literature on robust variance estimation in meta‐analysis, we tried different values of *rho* obtaining the same results (see Z. Fisher & Tipton, 2015, pp. 9–10).

9 A detailed description of the search/coding process and of the eligibility assessment is provided in the Supporting Information Appendix: Appendix A: Search categories and related search terms, Appendix B: Eligibility screening form, Appendix C: Document coding protocol.

10 The full risk of bias assessment table with the 28 items is available upon request.

11 On some occasions we synthesized additional independent measures using only effect sizes falling within each subcategory. The drop from 304 eligible effect sizes to 138 independent effect size is mostly due to the concentration of multiple effect sizes measuring conceptually similar constructs in few studies. For example, Bottini and colleagues (2017) reported 40 effect sizes of cognitive functioning of mafia members compared to offenders in general and a population sample. We synthesized the 40 measures into a single independent measure. Thanks to the collaboration of the authors, we also classified the effect sizes into distinct subcategories, in turn synthesizing different independent effect sizes for each subcategory.

12 The analyses were performed using the Stata 16 *meta* command (StataCorp, 2019).

13 Of the paired studies Decker et al. (2014)/Pyrooz et al. (2015), only Pyrooz et al. (2015) investigated being of foreign origins for involvement into organized crime. For this reason, this analysis includes only Pyrooz et al. (2015).

14 Bottini et al. (2017) also reported a measure of traumatic brain injury, which was excluded due to computational issues associated with no events observed in one of the two groups.

15 Included studies also reported factors treated as correlates when they were not measured before organized crime membership or when it was impossible to determine whether the events had occurred before involvement into organized crime. The analysis of correlates is in the following subsection.

16 Schimmenti et al. (2014) reported two measures: PCL‐R – Antisocial and the overall Psychopathy Check List‐Revised (PCL‐R). For this meta‐analysis, we only included the latter measure, while for the meta‐analysis of the subcategory “Antisocial personality disorder” we included the former measure.

17 The correlate of adoption of the “code of the street” was conceptualized as violence as the authors indicate that the code of the street is based on how well respondents of the study agreed with the use of violence (see Decker et al., 2014, p. 9).
