# Supplementary material for: Organized crime groups: A systematic review of individual‐level risk factors related to recruitment
Source: Campbell Syst Rev. 2022 Feb 11;18(1):e1218. doi: 10.1002/cl2.1218 (PMC8833286; doi:10.1002/cl2.1218)

# Appendix

## Appendix A: Search categories and related search terms

The initial search was conducted between January and March 2017. An updated search was performed between September and October 2019.

Table 10. Search categories and related search terms

| **Search category** | **Search terms** |
| --- | --- |
| Organized Crime Group | criminal organisation |
|  | criminal organization |
|  | criminal association |
|  | organized crime |
|  | organised crime |
|  | mafia |
|  | crim* network* |
|  | dto* |
|  | drug trafficking organ* |
|  | motorcycle gang* |
|  | bikie gang* |
|  | crim* group* |
|  | crim* cartel |
| Factor | risk factor* |
|  | predictor* |
|  | driver* |
|  | determinant* |
|  | correlate* |
| Recruitment | involv* |
|  | recruit* |
|  | starter* |
|  | affiliat* |
|  | membership |
|  | criminal career* |
|  | criminal trajector* |

Table 11. Databases and related queries

| 1. **Database** | 1. **Query** |
| --- | --- |
| 1. **Criminal Justice Abstracts** 2. **(EBSCOHost)** 3. **(1910-2019)** | 1. AB (("criminal organisation" OR "criminal organization" OR "criminal association" OR "organized crime" OR "organised crime" OR mafia OR "crim* network*" OR dto* OR "drug trafficking organ*" OR "drug cartel*" OR "motorcycle gang*" OR "bikie gang*" OR "crim* group*" OR "crim* cartel")) AND AB ((involv* OR starter* OR affiliat* OR membership OR recruit* OR "criminal career*" OR "criminal trajector*")) AND AB (("risk factor*" OR predictor* OR driver* OR determinant* OR correlate*)) |
| 1. **Open Grey** 2. **(2008-2019)** | 1. ("criminal organisation" OR "criminal organization" OR "criminal association" OR "organized crime" OR "organised crime" OR mafia OR "crim* network*" OR dto* OR "drug trafficking organ*" OR "drug cartel*" OR "motorcycle gang*" OR "bikie gang*" OR "crim* group*" OR "crim* cartel") AND (involv* OR starter* OR affiliat* OR membership OR recruit* OR "criminal career*" OR "criminal trajector*") AND ("risk factor*" OR predictor* OR driver* OR determinant* OR correlate*) NOT(narcosis OR ganglion* OR narcolept* OR marathon* OR organ* OR maraviroc* OR gangetic* OR gangue OR "marangoni" OR narcoleps* OR ganger OR mafic OR maranh*) lang:"en" |
| 1. **Social Sciences Premium** 2. **(1871-2019)** 3. **+** 4. **NJCRS (1912-2015)** 5. **+** 6. **APA PsycInfo** 7. **(1878-2019)** 8. **+** 9. **ABI/INFORM Collection** 10. **(1970-2019)** 11. **+** 12. **International Bibliography of the Social Sciences (1951-2019)** 13. **+** 14. **Public Health Database** 15. **(1987-2019)** 16. **+** 17. **Military Database (1976-2019)** 18. **+** 19. **EconLit (1969-2019)** 20. **+** 21. **APA PsycArticles (1894-2019)** 22. **(ProQuest)** | 1. AB("criminal organisation" OR "criminal organization" OR "criminal association" OR "organized crime" OR "organised crime" OR mafia OR "crim* network*" OR dto* OR "drug trafficking organ*" OR "drug cartel*" OR "motorcycle gang*" OR "bikie gang*" OR "crim* group*" OR "crim* cartel") AND AB(involv* OR starter* OR affiliat* OR membership OR recruit* OR "criminal career*" OR "criminal trajector*") AND AB("risk factor*" OR predictor* OR driver* OR determinant* OR correlate*) |
| 1. **PubMed** 2. **(1966-2019)** | 1. ("organized crime"[Title/Abstract] OR "organised crime"[Title/Abstract] OR "criminal organization"[Title/Abstract] OR "criminal organisation"[Title/Abstract] OR "mafia"[Title/Abstract] OR "drug trafficking organization"[Title/Abstract] OR "drug trafficking organisation"[Title/Abstract]) AND (recruitment[Title/Abstract] OR affiliation[Title/Abstract] OR membership[Title/Abstract] OR "risk factor" [Title/Abstract] OR predictor[Title/Abstract] OR correlate[Title/Abstract]) |
| 1. **Scopus** 2. **(1823-2019)** | 1. (TITLE-ABS-KEY (("organised crime" OR "organized crime" OR "criminal organization" OR "criminal organisation" OR "mafia" OR "drug trafficking organization" OR "drug trafficking organisation")) AND TITLE-ABS-KEY ((recruitment OR affiliation OR membership "risk factor" OR predictor OR correlate)) AND NOT TITLE-ABS-KEY ((gangl OR narcosis OR narcolept OR marathon OR organ OR organs OR maraviroc OR gangetic))) AND (LIMIT-TO (SUBJAREA, "SOCI") OR LIMIT-TO (SUBJAREA, "MEDI") OR LIMIT-TO (SUBJAREA, "PSYC") OR LIMIT-TO (SUBJAREA, "ARTS") OR LIMIT-TO (SUBJAREA, "ECON") OR LIMIT-TO (SUBJAREA, "BUSI") OR LIMIT-TO (SUBJAREA, "NURS") OR LIMIT-TO (SUBJAREA, "NEUR") OR LIMIT-TO (SUBJAREA, "HEAL")) AND (LIMIT-TO (SRCTYPE, "j") OR LIMIT-TO (SRCTYPE, "b") OR LIMIT-TO (SRCTYPE, "k") OR LIMIT-TO (SRCTYPE, "p") OR LIMIT-TO (SRCTYPE , "d")) |
| 1. **Science Citation Index Expanded** 2. **+** 3. **Social Sciences Citation Index** 4. **+** 5. **Arts & Humanities Citation Index** 6. **+** 7. **Conference Proceedings Citation Index – Science** 8. **+** 9. **Conference Proceedings Citation Index – Social Sciences and Humanities** 10. **+** 11. **Book Citation Index – Science** 12. **+** 13. **Book Citation Index – Social Sciences & Humanities** 14. **+** 15. **Emerging Sources Citation Index** 16. **(Web of Science)** 17. **(1985-2019)** | 1. (TI=("criminal organisation" OR "criminal organization" OR "criminal association" OR "organized crime" OR "organised crime" OR mafia OR "crim* network*" OR dto* OR "drug trafficking organ*" OR "drug cartel*" OR "motorcycle gang*" OR "bikie gang*" OR "crim* group*" OR "crim* cartel") AND TI=(involv* OR starter* OR affiliat* OR membership OR recruit* OR "criminal career*" OR "criminal trajector*") AND TI=("risk factor*" OR predictor* OR driver* OR determinant* OR correlate*)) AND LANGUAGE: (English); Indexes=SCI-EXPANDED, SSCI, A&HCI, CPCI-S, CPCI-SSH, BKCI-S, BKCI-SSH, ESCI, CCR-EXPANDED, IC Timespan=All years |
| 1. **Google Scholar** 2. **(1792-2019)** | 1. (milieu OR organisat* criminelle* OR criminalité organisée OR criminels organisés OR cartel criminel OR mafia) AND (facteur* OR risq* OR recrut*) |
| 1. **Sudoc.Abes** 2. **(1814-2019)** | 1. (milieu OR organisat* criminelle* OR criminalité organisée OR criminels organisés OR cartel criminel OR mafia) AND (facteur* OR risq* OR recrut*) |
| 1. **Sowiport** 2. **(1974-2019)** | 1. (“organisierte kriminalität” OR kriminelle* organisation* OR kriminelle* vereinigung* OR kriminelle* kartell* OR mafia* OR mafiaähnlich* OR motorradclub*) AND (OR faktor* OR prädiktor*) |
| 1. **Liliacs** 2. **(1919-2019)** | 1. (mafia OR "grupo criminal" OR "asociacion criminal" OR "crimen organizado" OR cartel OR "delincuencia organizada") AND (riesgo OR reclutamiento OR "carrera criminal" OR factor) |
| 1. **Latin America & Iberia Database** 2. **(ProQuest)** 3. **(1990-2019)** | 1. FT((mafia OR “grupo criminal” OR “asociacion criminal” OR “crimen organizado” OR cartel OR “delincuencia organizada”) AND (riesgo OR reclutamiento OR “carrera criminal” OR factor)) |
| 1. **Riviste Web** 2. **(1990-2019)** | 1. (“crimine organizzato” OR “criminalità organizzata” OR “associazione delinquere” OR mafia OR “organizzazione criminale”) AND (reclut* OR affilia* OR fattor* OR rischi* OR carriera) |

## Appendix B: Eligibility screening form

Table 12. Eligibility screening form

| **Eligibility item** | **Values** |
| --- | --- |
| 1. Does the document report on the OCGs as defined in this review? | 0 = No  1 = Yes  99 = Can’t tell  *If no, then stop* |
| 2. Does the document investigate recruitment into OCGs as one of its main objectives? | 0 = No  1 = Yes  99 = Can’t tell  *If no, then stop* |
| 3. Does the document make any empirical contribution to the study of the recruitment into OCGs? | 0 = No  1 = Yes  99 = Can’t tell  *If no, then stop* |
| 4. Does the study discuss sufficiently well-defined factor leading to recruitment into OCGs? For quantitative studies, does each factor measure a single, reasonably defined characteristic? | 0 = No  1 = Yes  99 = Can’t tell  *If no, then stop* |
| 5. Are factors of recruitment into OCGs assessed on an individual level? | 0 = No  1 = Yes  99 = Can’t tell  *If no, then stop* |
| 6. If the document follows a quantitative or mixed-method approach, does the study design allow to capture a sufficient variability between OCG members and non-OCG members? | 0 = No  1 = Yes  99 = Can’t tell  *If no, then stop* |

## Appendix C: Document coding protocol

Table 13. Document coding protocol (all documents)

| **Section** | **Item no.** | **Item** | **Value** |
| --- | --- | --- | --- |
| **Reference information** | 1 | Study ID |  |
|  | 2 | Study authors |  |
|  | 3 | Study title |  |
|  | 4 | Publication year |  |
|  | 5 | Reference type | 1. Peer reviewed journal article 2. Book 3. Book chapter 4. Thesis or dissertation 5. Other: _____ |
|  | 6 | Complete APA reference |  |
| **Study details** | 7 | Language | 1. English 2. Spanish 3. Italian 4. French 5. German |
|  | 8 | Geographic scope | World region/Country |
|  | 9 | Data source | 1. Compiled by researcher (e.g., survey) 2. Publicly available database: _____ 3. Judicial records: _____ 4. Investigative/police files: _____ 5. Other: _____ |
|  | 10 | Research period | 1. Start: _____ 2. Finish: _____ |
|  | 11 | Ethical issues | 1. N 2. Y: _____ |
|  | 12 | Type of OCG | 1. Mafia 2. DTO 3. Adult gang 4. Outlaw motorcycle gang 5. Other OCG: _____ |
|  | 13 | OCG name |  |
|  | 14 | Study methodology | 1. Quantitative 2. Mixed methods 3. Qualitative |

If the study is classified as “quantitative” go to Table 14. If the study is classified as “qualitative” or “mixed methods”, go to Table 15.

Table 14. Coding protocol (only quantitative)

| **Section** | **Item no.** | **Item** | **Value** |
| --- | --- | --- | --- |
| **Study methodology** | 15 | Type of observational study, if applicable | 1. Longitudinal 2. Cross-sectional 3. Case control 4. NA |
|  | 16 | Is the data source the same for the OCG and non-OCG groups? | 1. Y 2. N 3. Unclear |
|  | 17 | If not, what is the data source for non-OCG group(s)? | 1. Compiled by researcher (e.g., survey) 2. Publicly available database: _____ 3. Judicial records:____ 4. Investigative/police files: _____ 5. Other: _____ |
|  | 18 | Non-OCG group(s) composition (check any applicable) | 1. Former OCG members 2. Involved with (not formal affiliates of) an OCG 3. Serious non-OCG offenders 4. General non-OCG offenders 5. Non-criminal sample(s) (e.g., community/ population sample) 6. Other |
|  | 19 | Measure of OCG recruitment | 1. organized crime membership 2. OCG affiliation 3. Involvement in OC 4. Other |
|  | 20 | Nature of OCG recruitment measure | 1. Dichotomous 2. Categorical |
|  | 21 | Source of OCG recruitment measure | 1. Self-reported 2. Official data (e.g., judicial/police) 3. Other: _____ |
|  | 22 | Is OCG recruitment described in replicable detail? | 1. Y 2. N 3. Unclear |
|  | 23 | Total sample size |  |
|  | 24 | Size of OCG group |  |
|  | 25 | Size of non-OCG group |  |
|  | 26 | Sample gender | 1. M: _____ 2. F: _____ 3. Mixed |
|  | 27 | Sample SES | 1. Low 2. Average 3. High 4. Mixed |
|  | 28 | Is the study population described in replicable detail? | 1. Y 2. N 3. Unclear |
|  | 29 | Statistical model(s) used (e.g., logistical modelling) |  |
|  | 30 | Was (Were) the statistical model(s) internally or externally validated? |  |
|  | 31 | Model validation method(s) |  |
|  | 32 | Performance measures of the model(s) |  |
|  | 33 | Was data missing on risk factors or outcomes? | 1. Y 2. N 3. Unclear |
|  | 34 | If yes, how was missing data dealt with? |  |
| **Risk factors** | 35 | Risk factor |  |
|  | 36 | Risk factor domain | a. Sociodemographic  b. Economic status  c. Criminal history  d. Psychological  e. Other |
|  | 36a | Risk factor category |  |
|  | 36b | Risk factor subcategory |  |
|  | 37 | Conceptual definition of risk factor |  |
|  | 38 | Operational definition |  |
|  | 39 | Source of risk factor measure | 1. Self-reported 2. Official data (e.g., judicial/police) 3. Other: ____ |
|  | 40 | Risk factor measured retrospectively | 1. Y 2. N 3. Unclear |
|  | 41 | Is the risk factor time-invariant? | 1. Y 2. N. In this case in non-longitudinal studies the factor will be classified as correlate 3. Unclear |
|  | 42 | Was the effect size reported? | 1. Y 2. N |
|  | *If yes:* | | |
|  | 43 | Reported risk factor effect size |  |
|  | 44 | ES standard error |  |
|  | 45 | ES confidence intervals |  |
|  | *If not, we will use available data to calculate it:* | | |
|  | 46 | Mean value (OCG and non-OCG groups) |  |
|  | 47 | Standard deviation (OCG and non-OCG groups) |  |
|  | 48 | Alternatively, unadjusted correlation coefficient |  |
|  | 49 | Alternatively, standardized correlation coefficient |  |
|  | 50 | Alternatively, unadjusted regression coefficient |  |
|  | 51 | Alternatively, standardized regression coefficient |  |
|  | 52 | If dichotomous, fraction of OCG and non-OCG groups with risk factor |  |
|  | 53 | n size of OCG and non-OCG groups for risk factor |  |
|  | 54 | Risk factor difference between OCG and non-OCG groups |  |
|  | 55 | Extrapolated risk factor effect size |  |
|  | 56 | Extrapolated ES standard error |  |
|  | 57 | Extrapolated ES confidence intervals |  |
| **Risk of study bias** | *a. Risk of bias due to sampling and setting* | | |
|  | 58 | Are all sample inclusion/exclusion criteria listed? | 1. Y 2. N 3. Unclear |
|  | 59 | What are the inclusion/exclusion criteria? |  |
|  | 60 | Sample selection precedes OCG involvement? | 1. Y 2. N 3. Unclear |
|  | 61 | Initial response rate, if applicable |  |
|  | 62 | Attrition rate, if applicable |  |
|  | 63 | Were all participants inclusion and exclusion choices appropriate? | a. Y  b. N  c. Unclear |
|  | 64 | Overall risk of bias due to sample selection? | a. Low  b. High  c. Unclear |
|  | 65 | Rationale of bias rating: |  |
|  | *b. Risk of bias due to the risk factors or their assessment* | | |
|  | 66 | Were all risk factors described in replicable detail? | 1. Y 2. N 3. Unclear |
|  | 67 | Were risk factors defined and assessed in a similar way for all participants? | a. Y  b. N  c. Unclear |
|  | 68 | Were all risk factors based on validated measures? | 1. Y 2. N 3. Unclear |
|  | 69 | Is there a pre-measure for all risk factors (including obtained retrospectively)? | 1. Y 2. N 3. Unclear |
|  | 70 | Were confounding factors measured before OCG involvement (including obtained retrospectively)? | 1. Y 2. N 3. Unclear |
|  | 71 | Overall risk of bias due to risk factors or their assessment? | a. Low  b. High  c. Unclear |
|  | 74 | Rationale of bias rating: |  |
|  | *d. Risk of bias due to statistical procedures* | | |
|  | 75 | Was there a reasonable number of individuals in the sample? | a. Y  b. N  c. Unclear |
|  | 76 | Were continuous and categorical risk factors statistically handled appropriately? | a. Y  b. N  c. Unclear |
|  | 77 | If applicable, were all enrolled participants included in the analysis? | a. Y  b. N  c. Unclear |
|  | 78 | Were missing data handled appropriately? | a. Y  b. N  c. Unclear |
|  | 79 | Were complexities in the data (e.g., sampling of controls) accounted for appropriately? | a. Y  b. N  c. Unclear |
|  | 80 | Overall risk of bias due to statistical analysis? | a. Low  b. High  c. Unclear |
|  | 81 | Rationale of bias rating: |  |
|  | *e. Overall study risk of bias* | | |
|  | 82 | Overall judgement of risk of bias | a. Low  b. High  c. Unclear |
|  | 83 | Summary of sources of potential risk |  |
|  | 84 | Overall judgement of study applicability to the research question | a. Low  b. High  c. Unclear |
|  | 85 | Summary of applicability concerns |  |

Table 15. Coding protocol (only mixed methods and qualitative)

| **Section** | **Item no.** | **Item** | **Value** |
| --- | --- | --- | --- |
| **Study methodology** | 86 | Type of observational study, if applicable | a. Longitudinal |
|  |  |  | b. Cross-sectional |
|  |  |  | c. Case control |
|  |  |  | d. NA |
|  | 87 | Measure of OCG recruitment | a. organized crime membership |
|  |  |  | b. OCG affiliation |
|  |  |  | c. Involvement in OC |
|  |  |  | d. Other |
|  | 88 | Source of OCG recruitment measure | a. Self-reported |
|  |  |  | b. Official data (e.g., judicial/police) |
|  |  |  | c. Other: _____ |
|  | 89 | Total sample size, if applicable |  |
|  | 90 | Is the study population described in replicable detail? | a. Y |
|  |  |  | b. N |
|  |  |  | c. Unclear |
|  | 91 | Information & Data sources | a. Interviews |
|  |  |  | b. Interviews with key informants |
|  |  |  | c. Judicial/police documents |
|  |  |  | d. Historical documents |
|  |  |  | e. Ethnographic participant observation |
|  |  |  | f. Survey |
|  |  |  | g. Other sources |
| **Risk factors** | 92 | Risk factor |  |
|  | 93 | Risk factor domain | a. Sociodemographic |
|  |  |  | b. Economic status |
|  |  |  | c. Criminal history |
|  |  |  | d. Psychological |
|  |  |  | e. Other |
|  | 93a | Risk factor category |  |
|  | 93b | Risk factor subcategory |  |
|  | 94 | Conceptual definition of risk factor |  |
|  | 95 | Operational definition, if available |  |
|  | 96 | Risk factor measured retrospectively | a. Y |
|  |  |  | b. N |
|  |  |  | c. Unclear |
|  | 97 | Is the risk factor time-invariant? | a. Y |
|  |  |  | b. N. In this case in non-longitudinal studies the factor will be classified as correlate |
|  |  |  | c. Unclear |
| **Quality assessment** | 98 | Clear aim on recruitment: the study main aim must be on the recruitment into organized crime, or the topic must be addressed in a relevant part of the study | a. Y |
|  |  |  | b. N |
|  |  |  | c. Unclear |
|  | 99 | Research design appropriate: clear indication of the research design adopted to investigate the recruitment into OCGs, or the research design must be the same for all the objectives of the study, including the recruitment. | a. Y |
|  |  |  | b. N |
|  |  |  | c. Unclear |
|  | 100 | Data collection appropriate: the study must clearly state the sources of information to investigate the recruitment into OCG, and/or the sources must be the same for the rest of the study. The study must offer indications on how the information was collected, verified, and analyzed. | a. Y |
|  |  |  | b. N |
|  |  |  | c. Unclear |
|  | 101 | Data analysis rigorous: the study must provide an in-depth description of the analysis, of the construction of categories and themes, present sufficient data. | a. Y |
|  |  |  | b. N |
|  |  |  | c. Unclear |
|  | 102 | Clear statement of findings: the study must clearly present the findings, discuss them in relation to limitations and other contributions. | a. Y |
|  |  |  | b. N |
|  |  |  | c. Unclear |

## Appendix D: Risk-of-bias assessment of the included quantitative studies

Table 16. Description of summary items of risk-of-bias assessment

| **Study** | **Domain of RB rating** | | **RB**  **rating** | | **Rating motivation** | |
| --- | --- | --- | --- | --- | --- | --- |
| **Adams & Pizarro, 2014** | a. Sampling and setting | Low | | Adequate inclusion criteria, same  data source for whole sample | |  |
|  |  |  |  |  |  |  |
|  |  |  |  |  |  |  |
|  | b. Risk factors and outcomes | High | | No pre-measures, unclear assessment of OC recruitment | |  |
|  |  |  |  |  |  |  |
|  |  |  |  |  |  |  |
|  | c. Statistical procedures | Low | | No statistical complexities | |  |
|  |  |  |  |  |  |  |
|  | d. Overall study RB | High | | Cross-sectional design, no pre-  measures, unclear assessment of OC  recruitment | |  |
|  |  |  |  |  |  |  |
|  |  |  |  |  |  |  |
|  |  |  |  |  |  |  |
| **Blokland et al., 2019** | a. Sampling and setting | High | | Different data source for OC and  non-OC groups with limited  matching (age) | |  |
|  |  |  |  |  |  |  |
|  |  |  |  |  |  |  |
|  | b. Risk factors and outcomes | High | | No pre-measures | |  |
|  | c. Statistical procedures | High | | Non-OC group is only matched on  age and otherwise random | |  |
|  |  |  |  |  |  |  |
|  |  |  |  |  |  |  |
|  | d. Overall study RB | High | | Cross-sectional design, no pre-  measures, possible sampling issues | |  |
|  |  |  |  |  |  |  |
|  |  |  |  |  |  |  |
| **Bottini et al., 2017** | a. Sampling and setting | Unclear | | Random selection for relatively small samples | |  |
|  |  |  |  |  |  |  |
|  |  |  |  |  |  |  |
|  | b. Risk factors and outcomes | High | | No pre-measures | |  |
|  |  |  |  |  |  |  |
|  | c. Statistical procedures | Low | | No statistical complexities | |  |
|  |  |  |  |  |  |  |
|  | d. Overall study RB | High | | Cross-sectional design, no pre-  measures, random selection for  relatively small samples | |  |
|  |  |  |  |  |  |  |
|  |  |  |  |  |  |  |
|  |  |  |  |  |  |  |
|  |  |  |  |  |  |  |
| **Carvalho**  **& Soares,**  **2016** | a. Sampling and setting | High | | Loose participation criteria; different sources for OC and non-OC samples | |  |
|  |  |  |  |  |  |  |
|  |  |  |  |  |  |  |
|  | b. Risk factors and outcomes | High | | No pre-measures; risk factors  assessed differently for OC and non-  OC samples | |  |
|  |  |  |  |  |  |  |
|  |  |  |  |  |  |  |
|  |  |  |  |  |  |  |
|  | c. Statistical procedures | Low | | No statistical complexities | |  |
|  |  |  |  |  |  |  |
|  | d. Overall study RB | High | | Cross-sectional design; no pre-  measures; different sources and risk  factor definitions for OC and non-OC  groups; loose participation criteria | |  |
|  |  |  |  |  |  |  |
|  |  |  |  |  |  |  |
|  |  |  |  |  |  |  |
|  |  |  |  |  |  |  |
|  |  |  |  |  |  |  |
| **Coid et al.,**  **2013** | a. Sampling and setting | Unclear | | Same data source for both samples;  generally appropriate participation  criteria except for age (why limit to  18-34?) | |  |
|  |  |  |  |  |  |  |
|  |  |  |  |  |  |  |
|  |  |  |  |  |  |  |
|  |  |  |  |  |  |  |
|  |  |  |  |  |  |  |
|  | b. Risk factors and outcomes | High | | No pre-measures | |  |
|  | c. Statistical procedures | Low | | Motivated oversampling of focus  (disadvantaged) population; no other statistical complexities | |  |
|  | d. Overall study RB | High | | Cross-sectional design; no pre-  measures | |  |
| **Danner &**  **Silverman,**  **1986** | a. Sampling and setting | High | | Loose participation criteria | |  |
|  | b. Risk factors and outcomes | High | | No pre-measures, exclusion of some  control variables, dubious assessment of OC membership | |  |
|  | c. Statistical procedures | Low | | Appropriate statistical tools given  data structure (risk factors measured using items that used binomial variables) | |  |
|  | d. Overall study RB | High | | Cross-sectional design; no pre-  measures; loose participation criteria; dubious OC membership assessment | |  |
| **Decker et al.,**  **2014** | a. Sampling and setting | | High | | Unclear participation criteria | |
|  | b. Risk factors and outcomes | High | | No pre-measures | |  |
|  | c. Statistical procedures | Low | | No statistical complexities | |  |
|  | d. Overall study RB | High | | Cross-sectional design; no pre-  measures | |  |
| **Francis et**  **Al., 2013** | a. Sampling and setting | Low | | Adequate inclusion criteria, same  data source for whole sample | |  |
|  | b. Risk factors and outcomes | High | | No pre-measures | |  |
|  | c. Statistical procedures | Low | | No statistical complexities | |  |
|  | d. Overall study RB | High | | Cross-sectional design; no pre-  measures | |  |
| **Kirby et al.,**  **2016** | a. Sampling and setting | Low | | Adequate inclusion criteria, same  data source for whole sample | |  |
|  | b. Risk factors and outcomes | High | | No pre-measures | |  |
|  | c. Statistical procedures | Low | | No statistical complexities | |  |
|  | d. Overall study RB | High | | Cross-sectional design; no pre-  measures | |  |
| **Kissner &**  **Pyrooz,**  **2009** | a. Sampling and setting | Unclear | | Loose inclusion criteria (random  sample of inmates with oversampling  of females) | |  |
|  | b. Risk factors and outcomes | High | | No pre-measures | |  |
|  | c. Statistical procedures | Low | | No statistical complexities | |  |
|  | d. Overall study RB | High | | Cross-sectional design; no pre-  measures; participation based on  random sampling (with oversampling  for females) | |  |
| **Klement,**  **2016** | a. Sampling and setting | Low | | Extensive discussion of participation  criteria; while OC and non-OC  samples have a different source  extensive matching should  compensate for this issue | |  |
|  | b. Risk factors and outcomes | Low | | Pre-measures present | |  |
|  | c. Statistical procedures | Low | | No statistical complexities; extensive matching of OC and non-OC groups | |  |
|  | d. Overall study RB | Low | | Pre-measures present and used to carry out diff-in-diff regression | |  |
| **Levitt &**  **Venkatesh,**  **2001** | a. Sampling and setting | Unclear | | Very localized (inhabitants of one housing project building in Chicago) but systematic sample. Age restriction may bias results | |  |
|  |  |  |  |  |  |  |
|  |  |  |  |  |  |  |
|  | b. Risk factors and outcomes | High | | No pre-measures | |  |
|  | c. Statistical procedures | Low | | No statistical complexities | |  |
|  |  |  |  |  |  |  |
|  | d. Overall study RB | High | | Cross-sectional design; no pre-  measures; probable issues of external validity due to highly localized sample | |  |
|  |  |  |  |  |  |  |
|  |  |  |  |  |  |  |
|  |  |  |  |  |  |  |
|  |  |  |  |  |  |  |
|  |  |  |  |  |  |  |
|  |  |  |  |  |  |  |
| **Ostrosky et**  **al., 2012** | a. Sampling and setting | Unclear | | OC sample was extracted (using  appropriate participation criteria)  from a list of high-security prisoner  case files of unclear origin, scope or  representativity | |  |
|  |  |  |  |  |  |  |
|  |  |  |  |  |  |  |
|  |  |  |  |  |  |  |
|  |  |  |  |  |  |  |
|  |  |  |  |  |  |  |
|  | b. Risk factors and outcomes | High | | No pre-measures | |  |
|  |  |  |  |  |  |  |
|  | c. Statistical procedures | Low | | No statistical complexities | |  |
|  | d. Overall study RB | High | | Cross-sectional design; no pre-  measures | |  |
|  |  |  |  |  |  |  |
|  |  |  |  |  |  |  |
| **Pedersen,**  **2018** | a. Sampling and setting | Low | | OC sample comes from high-quality  administrative sources; adequate  matched non-OC group comes from  similar sources | |  |
|  |  |  |  |  |  |  |
|  |  |  |  |  |  |  |
|  |  |  |  |  |  |  |
|  |  |  |  |  |  |  |
|  | b. Risk factors and outcomes | Low | | Pre-measures present; few  confounding factors considered (sex,  age, country of origin), but they are  used for matching | |  |
|  |  |  |  |  |  |  |
|  |  |  |  |  |  |  |
|  |  |  |  |  |  |  |
|  |  |  |  |  |  |  |
|  | c. Statistical procedures | Low | | No statistical complexities; matching of OC and non-OC groups | |  |
|  |  |  |  |  |  |  |
|  |  |  |  |  |  |  |
|  | d. Overall study RB | Low | | Pre-measures present; matching of  OC and non-OC groups | |  |
|  |  |  |  |  |  |  |
|  |  |  |  |  |  |  |
| **Pyrooz**  **Decker &**  **Moule,**  **2015** | a. Sampling and setting | High | | Unclear participation criteria | |  |
|  | b. Risk factors and outcomes | High | | No pre-measures or retrospective  data; some unclearly managed  missing data ("In some cases,  respondents declined to answer  specific items in the questionnaire.") | |  |
|  |  |  |  |  |  |  |
|  |  |  |  |  |  |  |
|  |  |  |  |  |  |  |
|  |  |  |  |  |  |  |
|  |  |  |  |  |  |  |
|  |  |  |  |  |  |  |
|  |  |  |  |  |  |  |
|  | c. Statistical procedures | Low | | No statistical complexities | |  |
|  |  |  |  |  |  |  |
|  | d. Overall study RB | High | | Cross-sectional design; no pre-  measures; some missing data | |  |
|  |  |  |  |  |  |  |
|  |  |  |  |  |  |  |
| **Schimmenti**  **et al., 2014** | a. Sampling and setting | Unclear | | Very localized (prisoners from one  institution) but systematic sample.  Small size though (30 and 39  individuals respectively in the OC  and non-OC samples) | |  |
|  |  |  |  |  |  |  |
|  |  |  |  |  |  |  |
|  |  |  |  |  |  |  |
|  |  |  |  |  |  |  |
|  | b. Risk factors and outcomes | High | | No pre-measures | |  |
|  | c. Statistical procedures | Low | | No statistical complexities, though  the sample is fairly small | |  |
|  |  |  |  |  |  |  |
|  |  |  |  |  |  |  |
|  | d. Overall study RB | High | | Cross-sectional design; no pre-  measures | |  |
|  |  |  |  |  |  |  |
|  |  |  |  |  |  |  |
| **Sharpe,**  **2002** | a. Sampling and setting | Low | | Intentionally loose and inclusive  participation criteria; however, the  final sample is large (396 and 1116  respectively in the OC and non-OC  samples) and distributed across a  number of institutions in North  Carolina, which should limit the risk of bias | |  |
|  |  |  |  |  |  |  |
|  |  |  |  |  |  |  |
|  |  |  |  |  |  |  |
|  |  |  |  |  |  |  |
|  |  |  |  |  |  |  |
|  |  |  |  |  |  |  |
|  |  |  |  |  |  |  |
|  | b. Risk factors and outcomes | | High | No pre-measures | |  |
|  |  |  |  |  |  |  |
|  |  |  |  |  |  |  |
|  | c. Statistical procedures | | High | Unclear treatment of non-OC group:  a fraction of responders was used in analysis; unclear if they were extracted randomly or matched at all | |  |
|  |  |  |  |  |  |  |
|  |  |  |  |  |  |  |
|  |  |  |  |  |  |  |
|  |  |  |  |  |  |  |
|  | d. Overall study RB | | High | Cross-sectional design; no pre-  measures; possibly biased use of  available non-OC group | |  |
|  |  |  |  |  |  |  |
|  |  |  |  |  |  |  |
| **Van**  **Koppen, de**  **Poot, &**  **Blokland,**  **2010** | a. Sampling and setting | | Low | Loose but adequate participation  criteria; large sample used; age-  matching | |  |
|  |  |  |  |  |  |  |
|  |  |  |  |  |  |  |
|  |  |  |  |  |  |  |
|  | b. Risk factors and outcomes | | Low | Pre-measures present, although only  judicial history is considered | |  |
|  |  |  |  |  |  |  |
|  |  |  |  |  |  |  |
|  |  |  |  |  |  |  |
|  | c. Statistical procedures | | Low | No statistical complexities; age-  matching of non-OC group | |  |
|  |  |  |  |  |  |  |
|  |  |  |  |  |  |  |
|  |  |  |  |  |  |  |
|  | d. Overall study RB | | Low | Pre-measures present; some matching | |  |
| **Wood,**  **Kallis &**  **Coid, 2017** | a. Sampling and setting | | Unclear | Same data source for both samples;  generally appropriate participation  criteria except for age (unclear limit to age range 19-30) | |  |
|  |  |  |  |  |  |  |
|  |  |  |  |  |  |  |
|  |  |  |  |  |  |  |
|  |  |  |  |  |  |  |
|  | b. Risk factors and outcomes | | High | No pre-measures; some variables are based on validated diagnostic  questionnaires, but for others it is  unclear how they were assessed | |  |
|  |  |  |  |  |  |  |
|  |  |  |  |  |  |  |
|  |  |  |  |  |  |  |
|  |  |  |  |  |  |  |
|  |  |  |  |  |  |  |
|  | c. Statistical procedures | | Low | No statistical complexities | |  |
|  |  |  |  |  |  |  |
|  |  |  |  |  |  |  |
|  |  |  |  |  |  |  |
|  | d. Overall study RB | | High | Cross-sectional design, no pre-  measures, potential risks associated  possibly with some risk factor  with age brackets for inclusion and  assessment | |  |
|  |  |  |  |  |  |  |
|  |  |  |  |  |  |  |

## Appendix E: Moderator analyses by type of organized criminal group

Figure 57. Moderator – Age


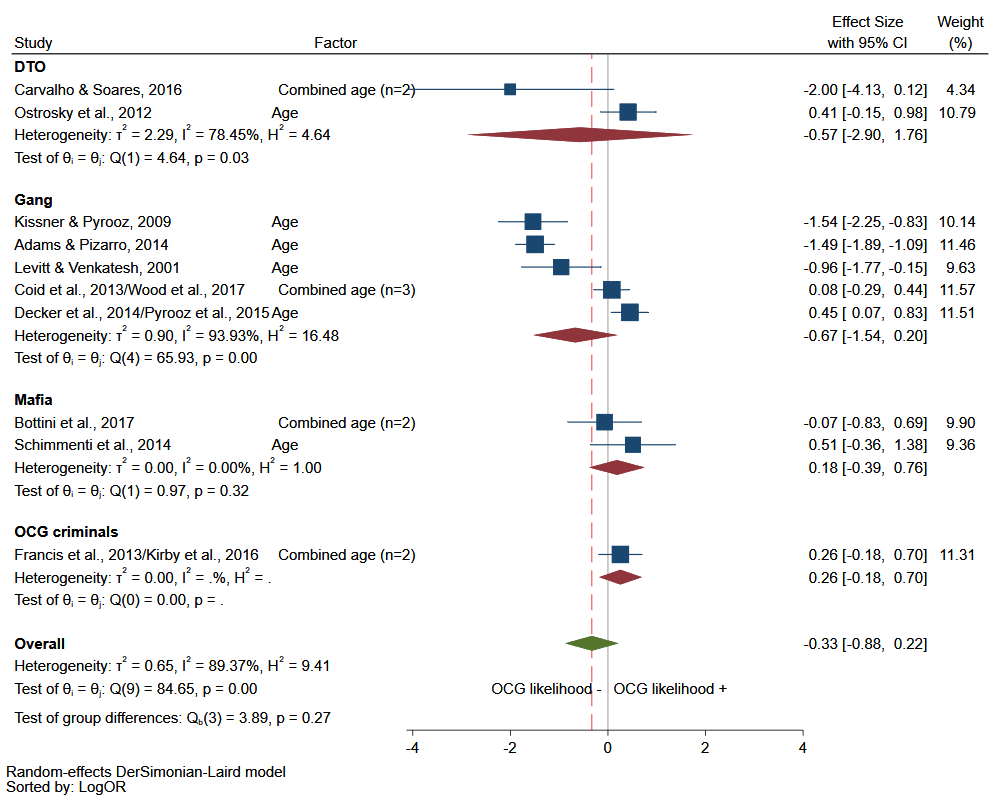


Figure 58. Moderator – Anxiety


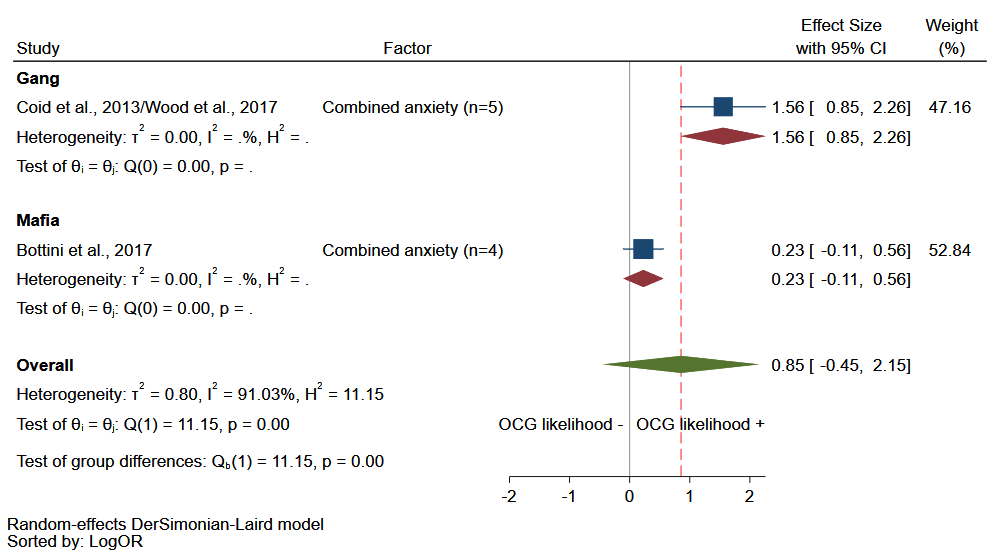


Figure 59. Moderator – Cognitive functioning


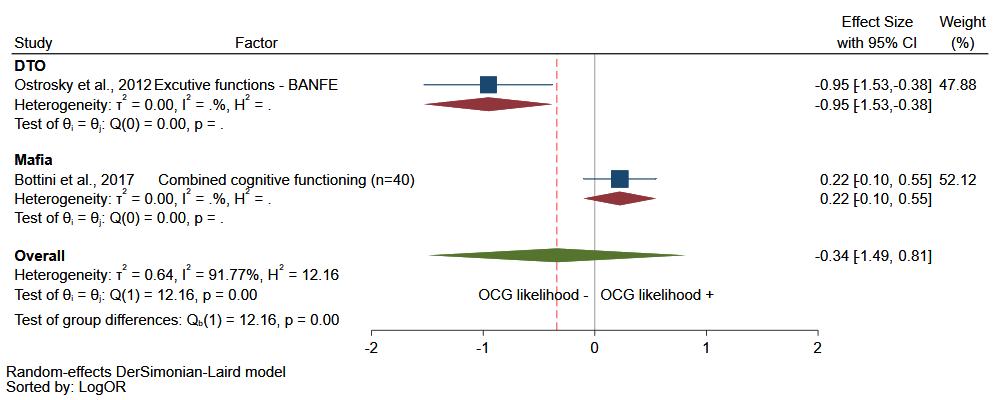


Figure 60. Moderator – Executive function


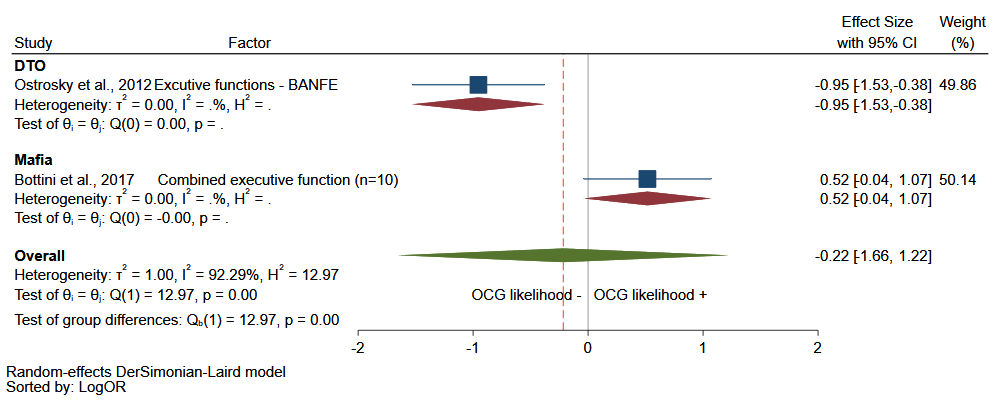


Figure 61. Moderator – Criminal versatility – Correlates


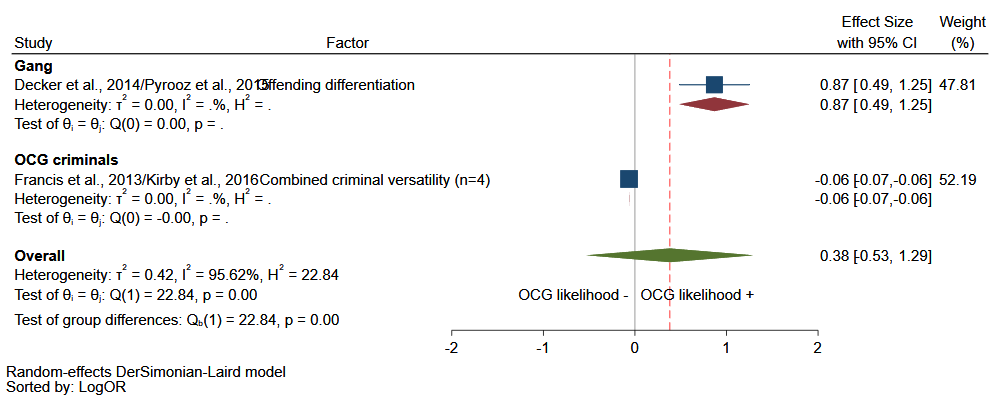


Figure 62. Moderator – Economic condition, risk factors


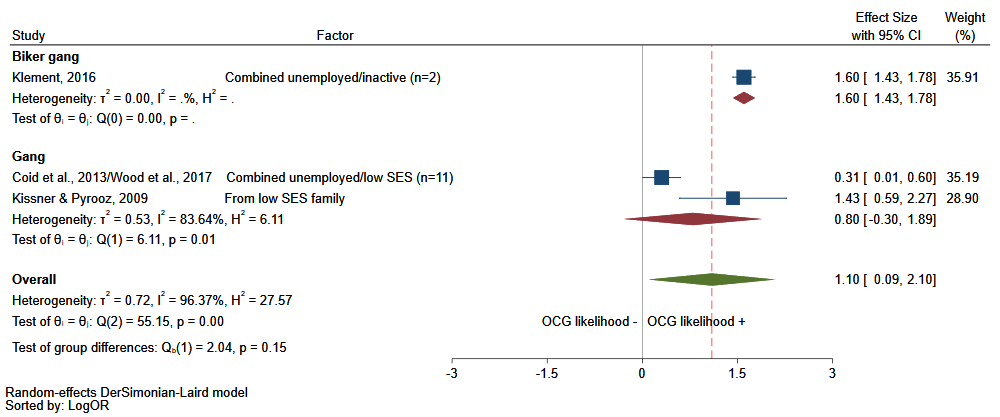


Figure 63. Moderator – Economic condition, protective factors


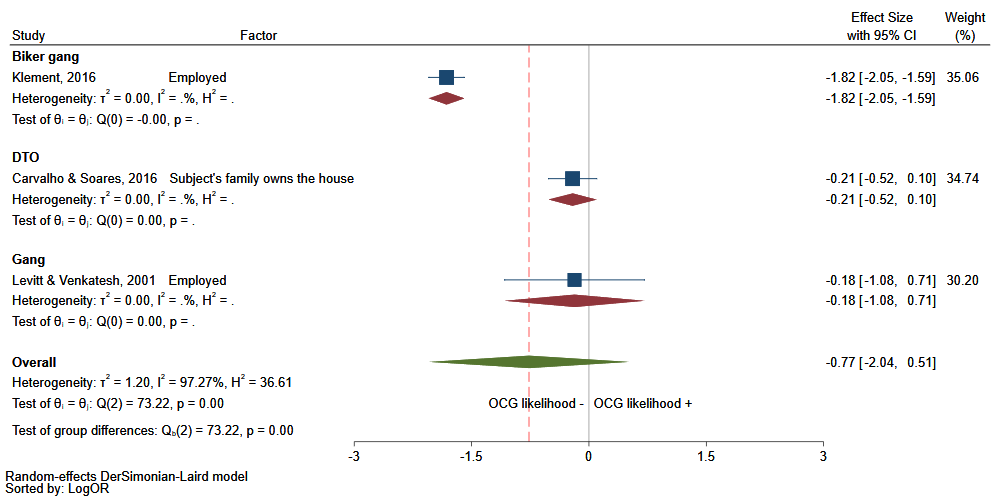


Figure 64. Moderator – Education


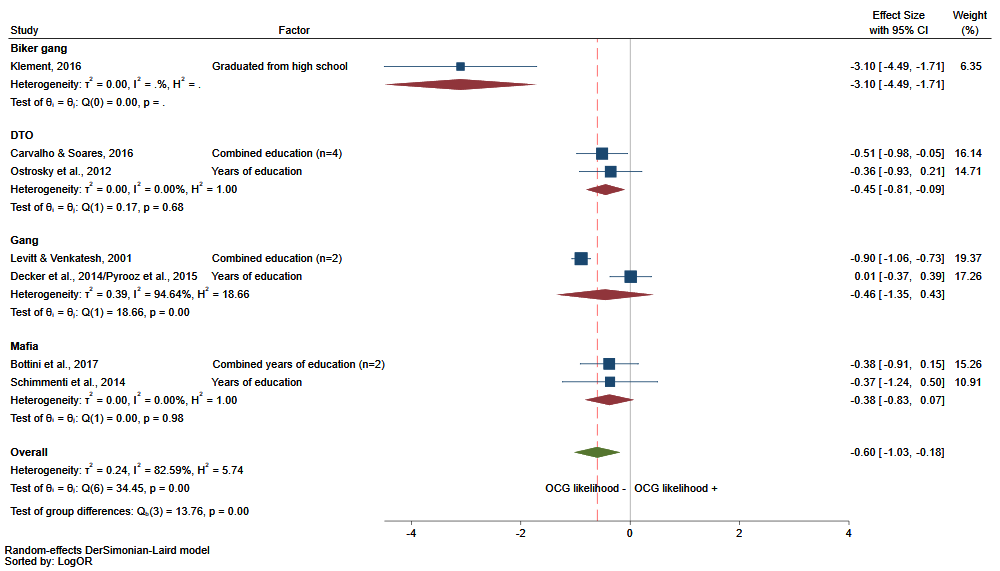


Figure 65. Moderator – High School


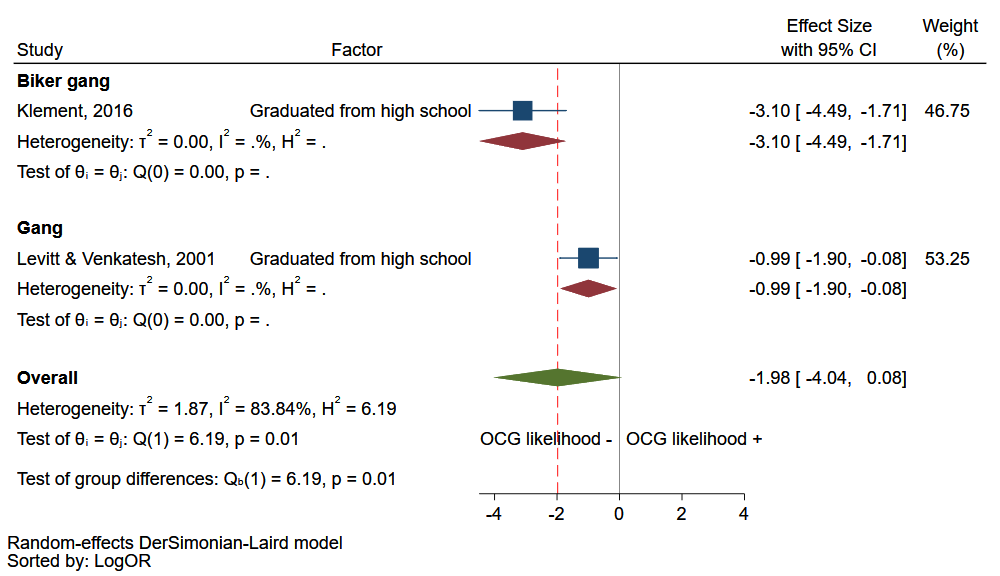


Figure 66. Moderator – Ethnicity, black


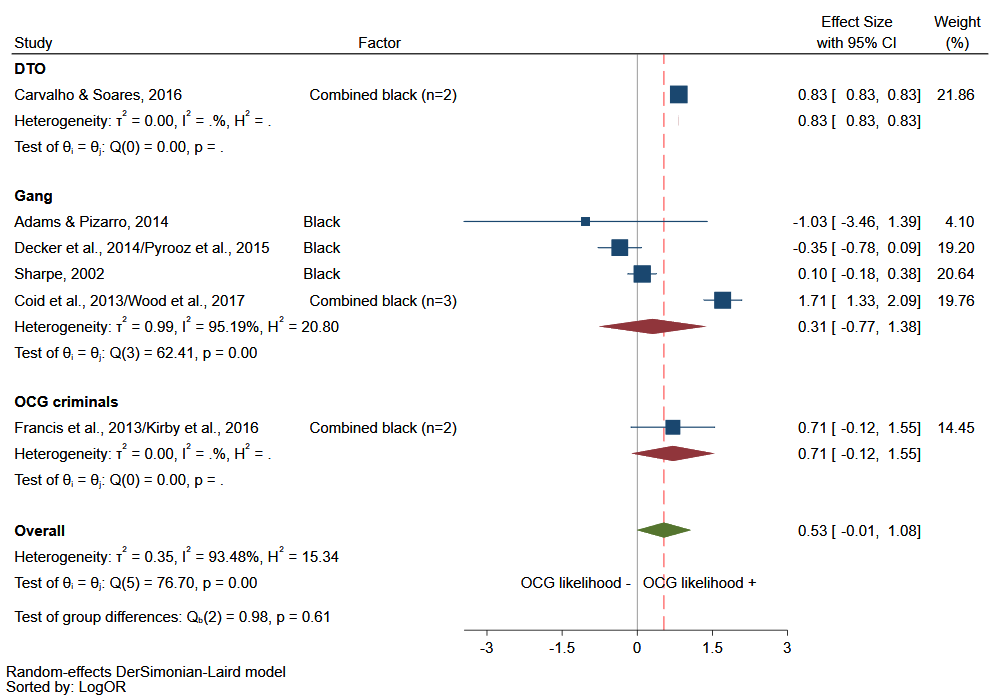


Figure 67. Moderator – Ethnicity, white


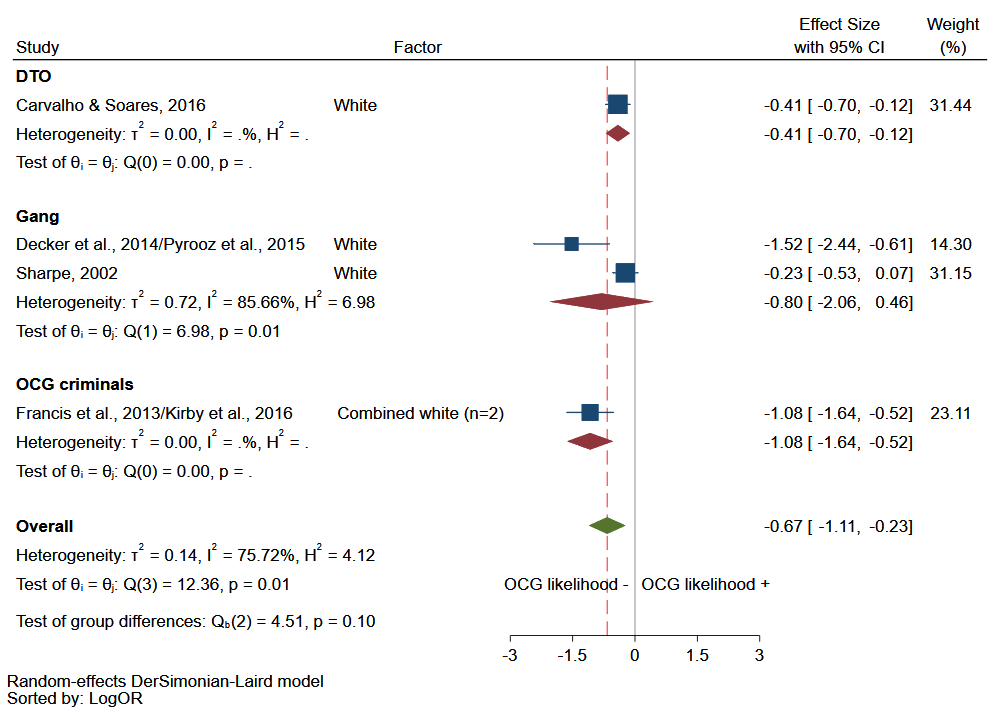


Figure 68. Moderator – Ethnicity, any (non-white)


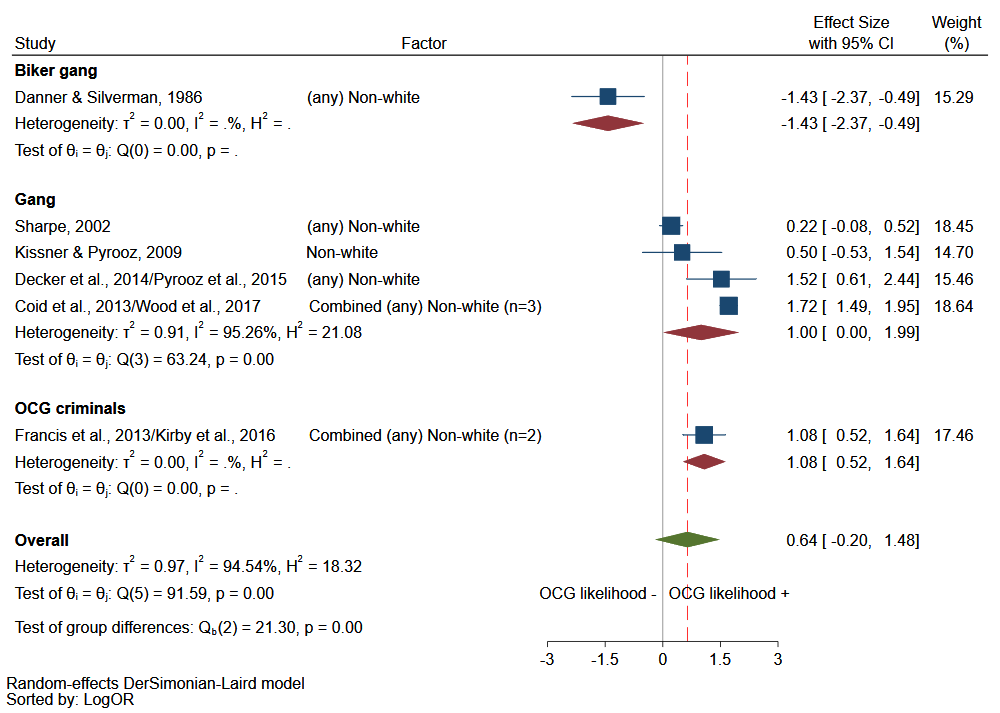


Figure 69. Moderator – Foreign born


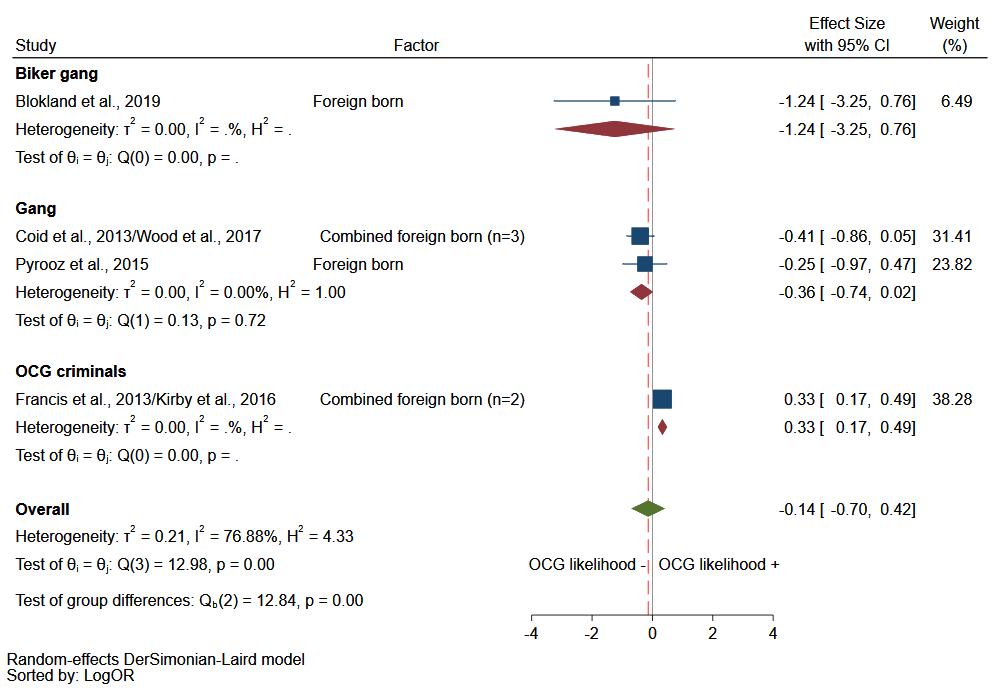


Figure 70. Moderator – Low self-control


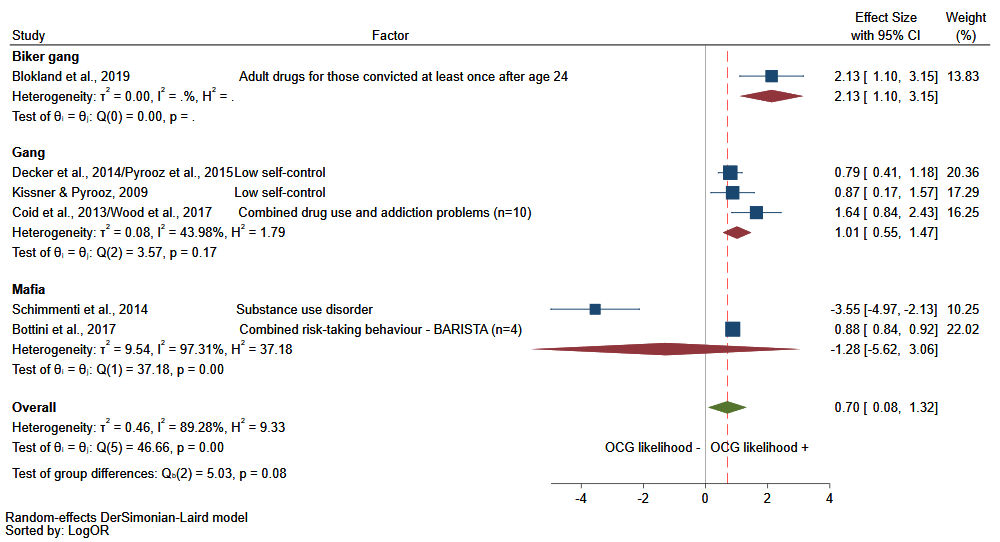


Figure 71. Moderator – Offence/contact with the criminal justice system – Predictors


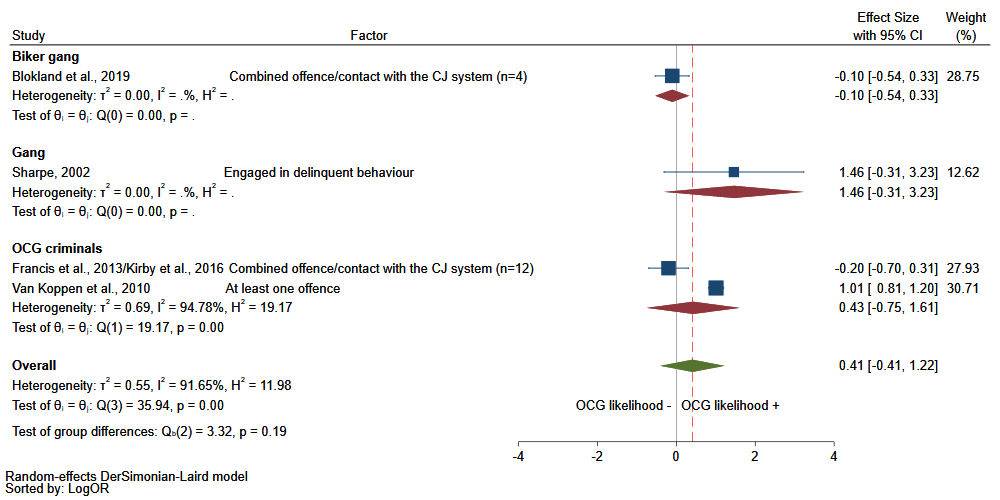


Figure 72. Moderator – N. of convictions – Predictors


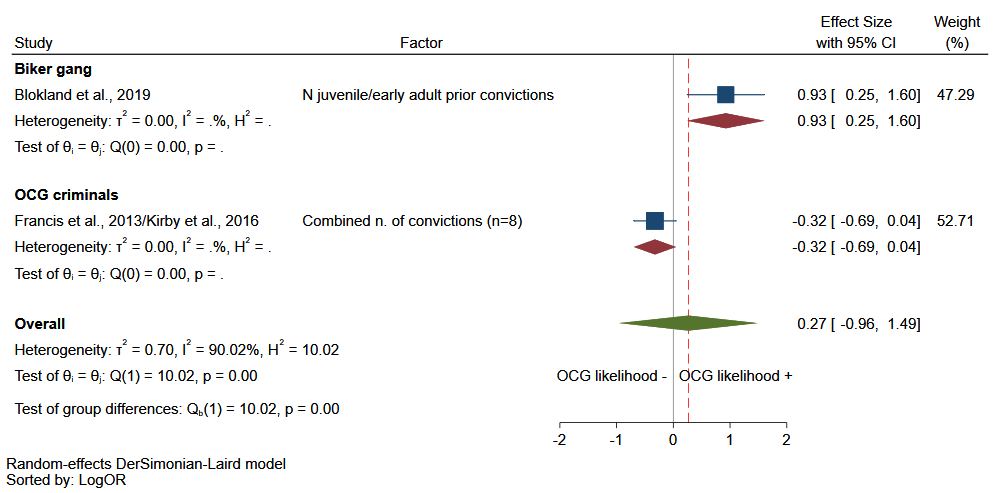


Figure 73. Moderator – Offence/contact with the criminal justice system – Correlates


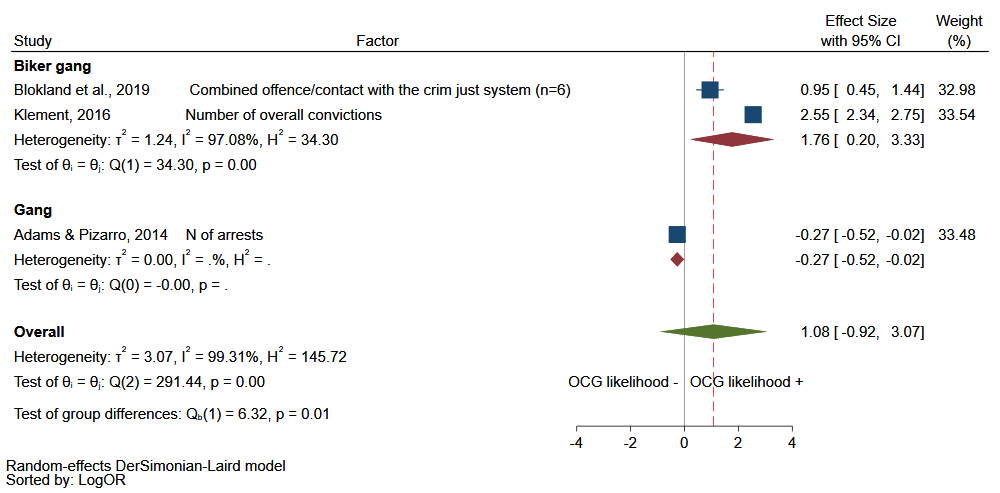


Figure 74. Moderator – N. of convictions – Correlates


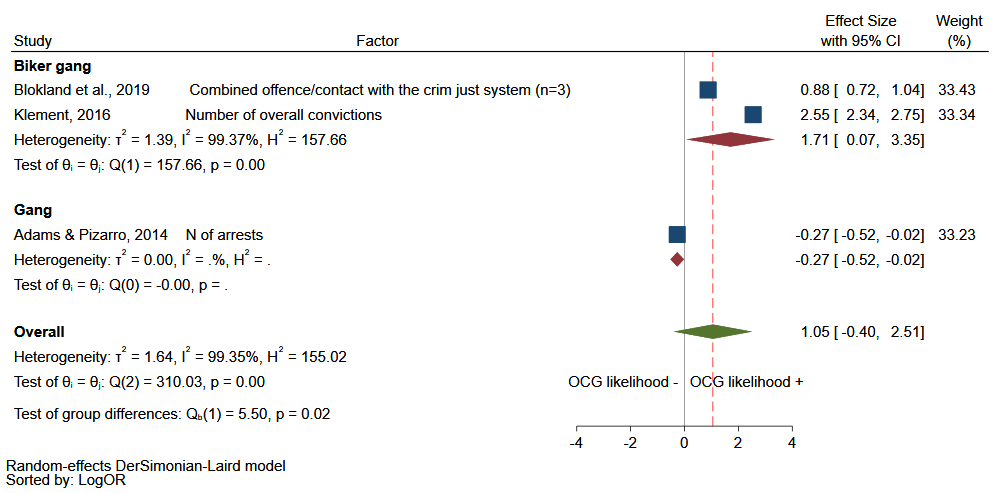


Figure 75. Moderator – Drug use and addiction problems


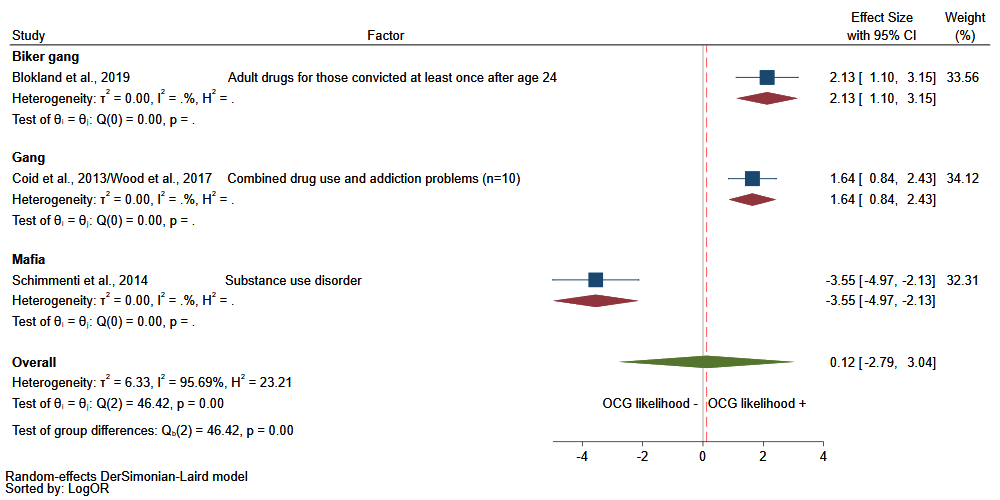


Figure 76. Moderator – First offence: other


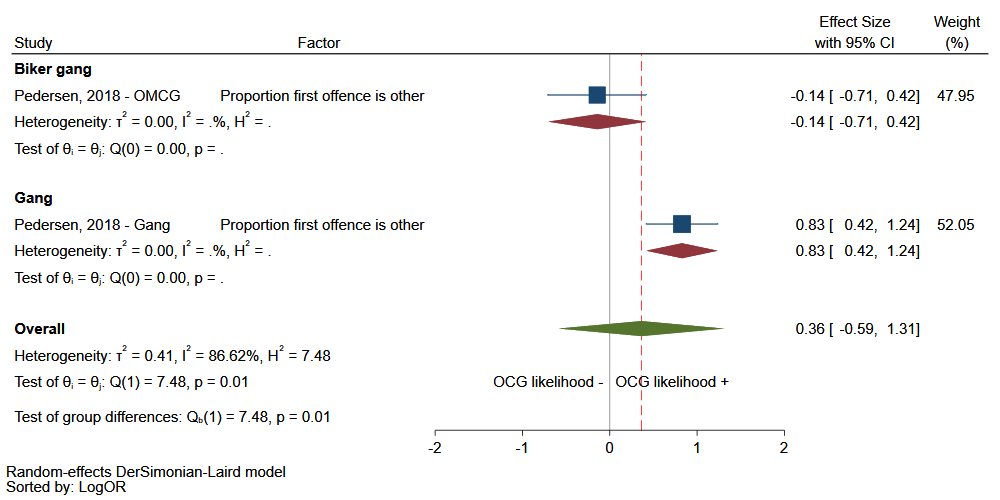


Figure 77. Moderator – First offence: sexual


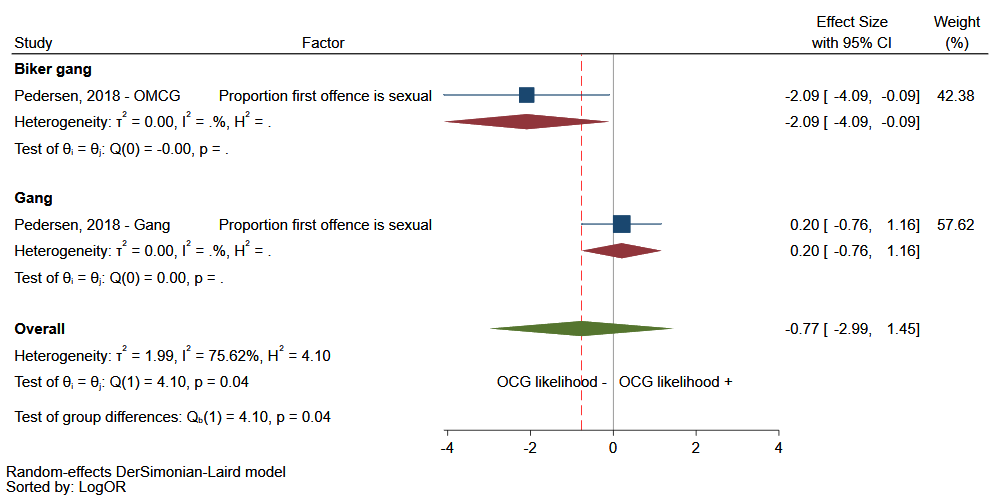


Figure 78. Moderator – Weapon offences – Predictors


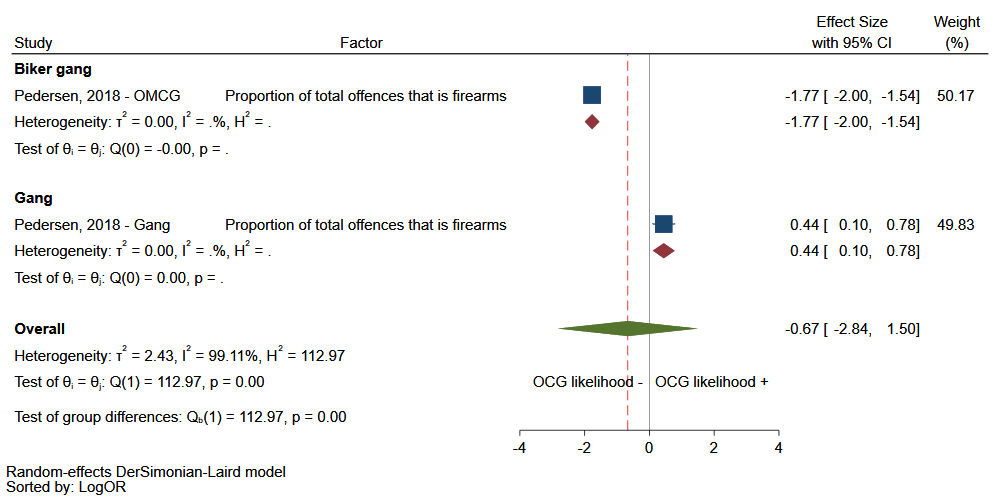


Figure 79. Moderator – Drug offences – Correlates


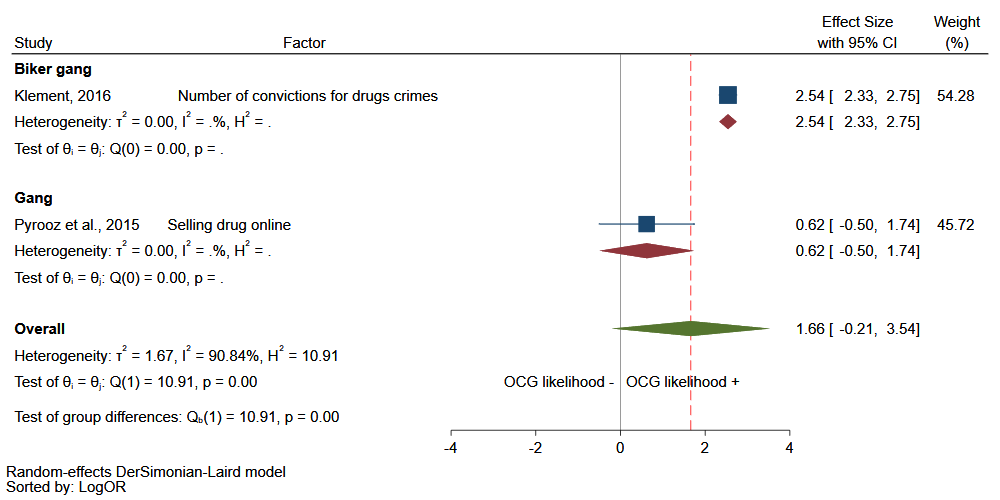


Figure 80. Moderator – Property offences – Correlates


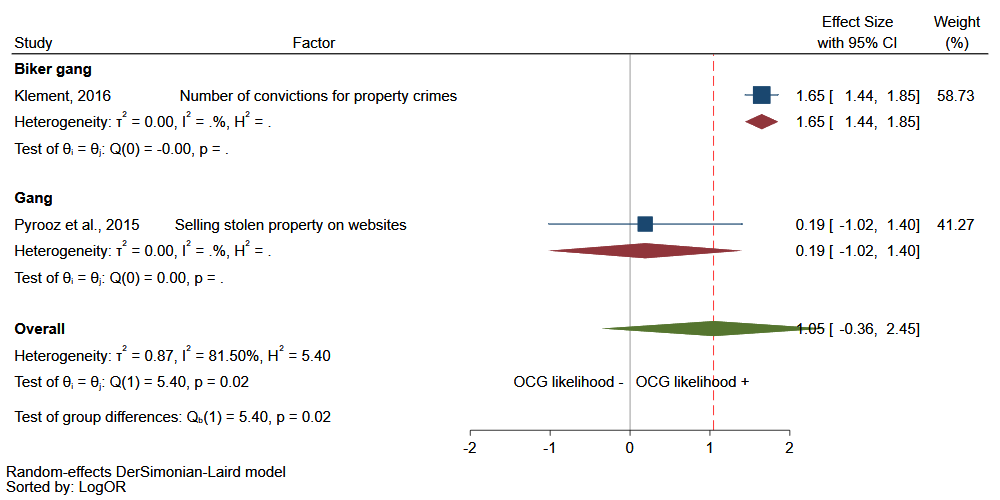


Figure 81. Moderator – Psychopathy and antisocial personality disorder


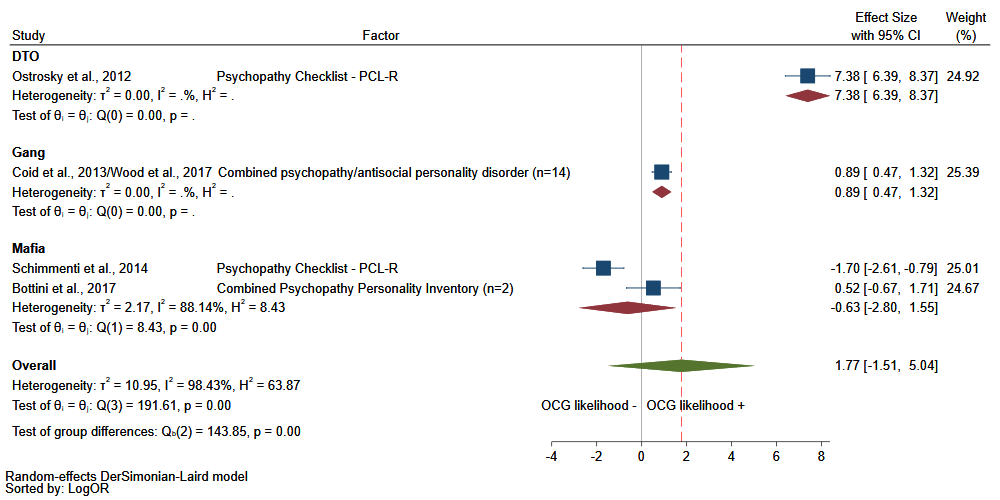


Figure 82. Moderator – Psychopathy


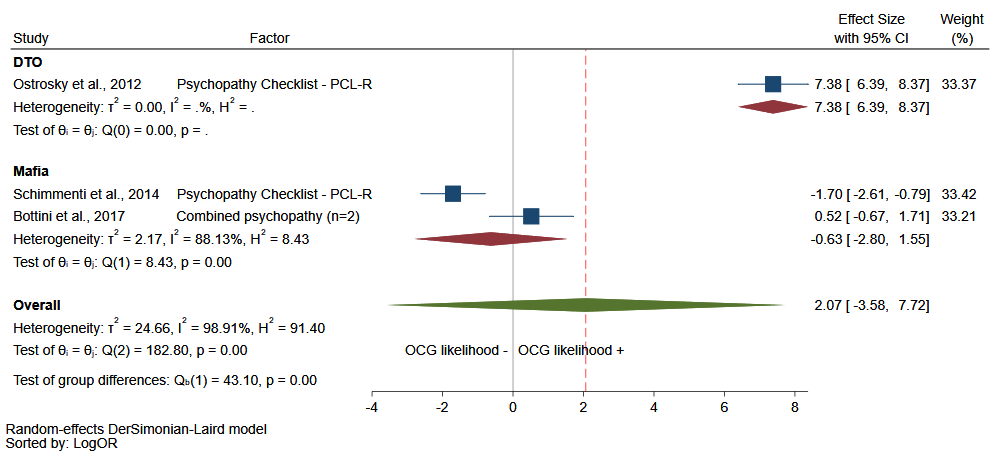


Figure 83. Moderator – Sanction seriousness


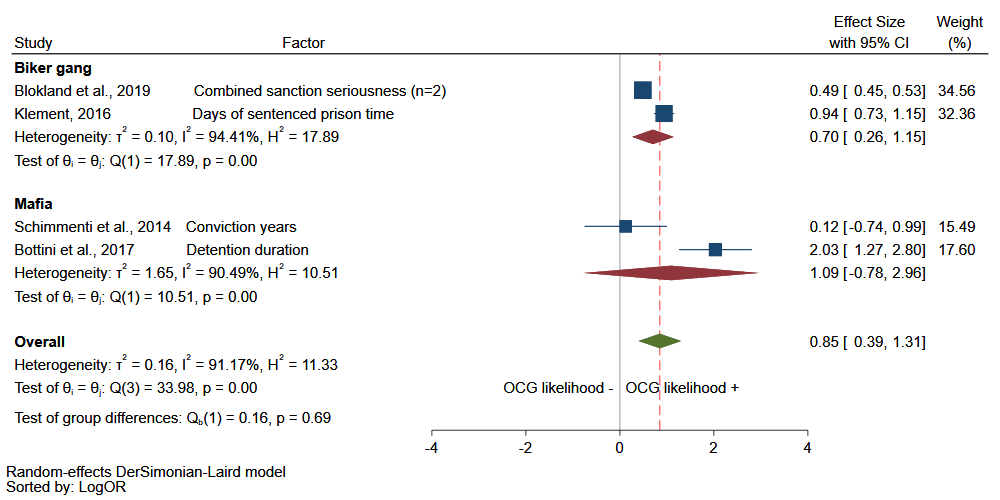


Figure 84. Moderator – Violence – Predictors


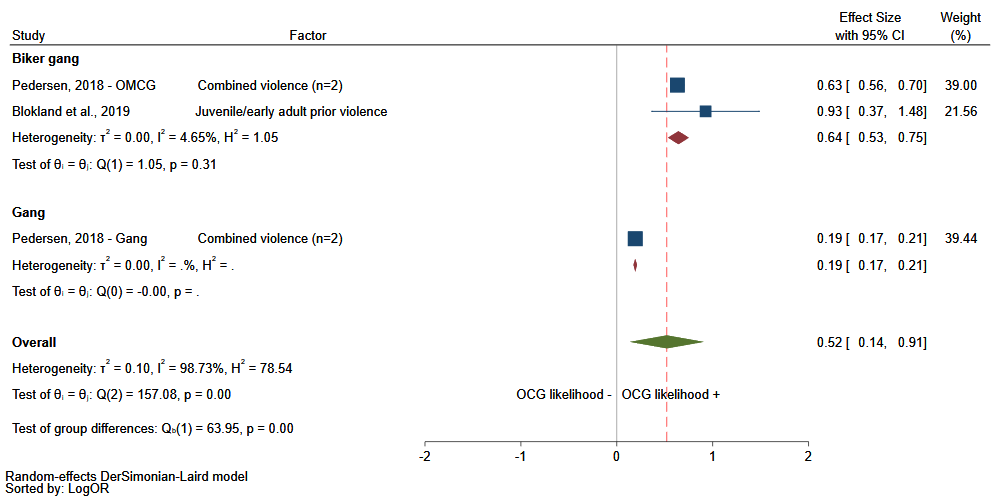


Figure 85. Moderator – Violent first offence


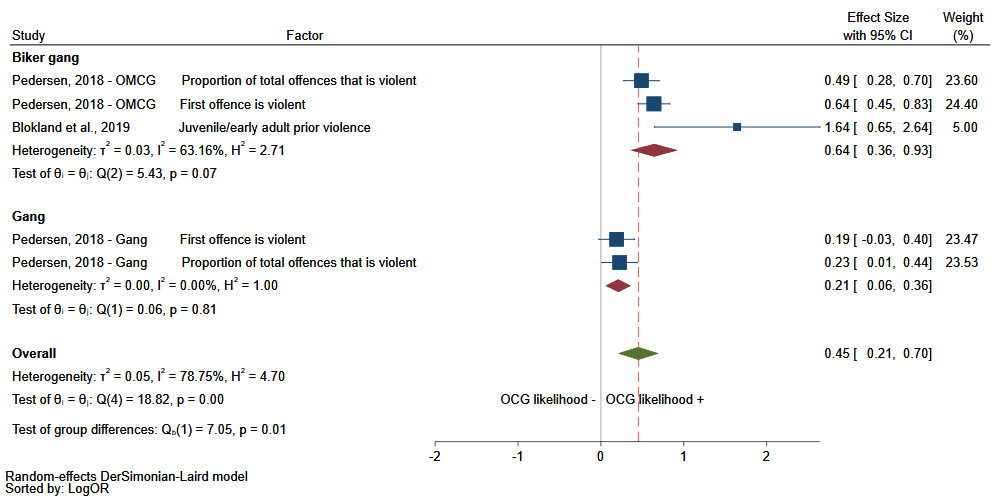


Figure 86. Moderator – Violent offences – Predictors


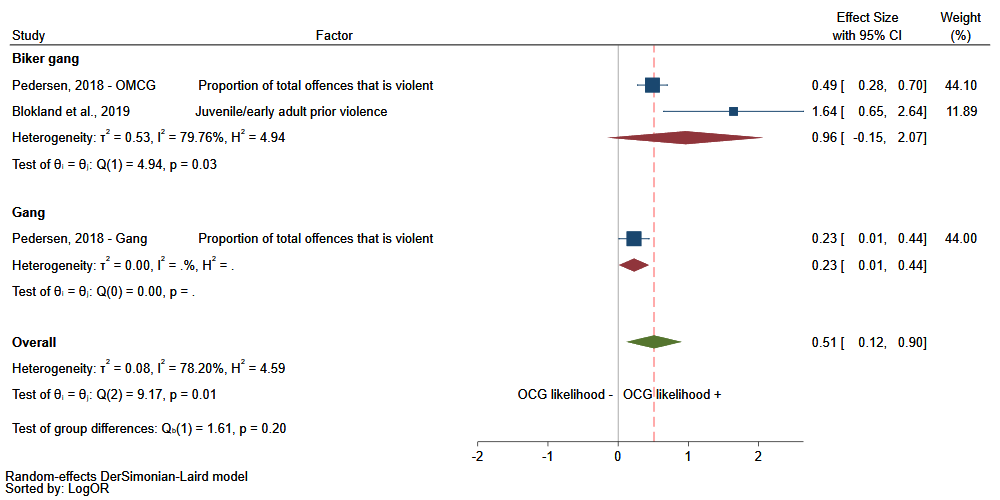


Figure 87. Moderator – Violence – Correlates


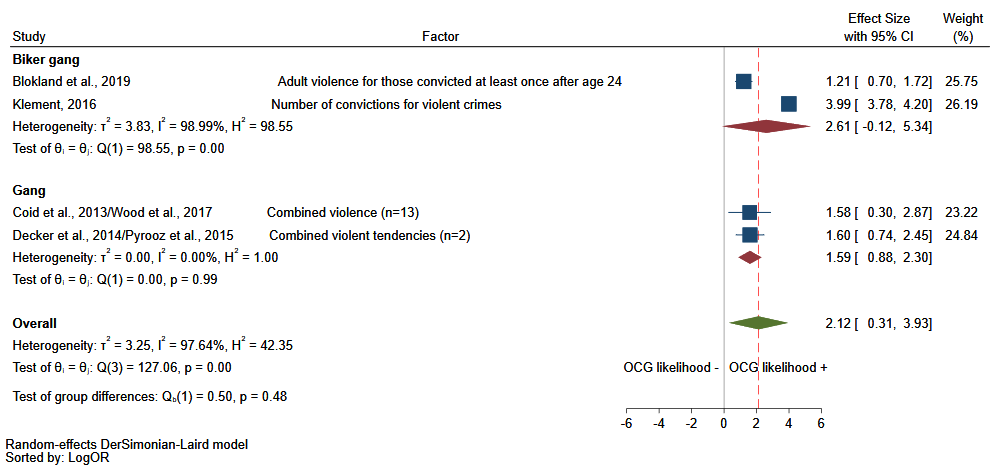


Figure 88.Moderator – Violent offences – Correlates


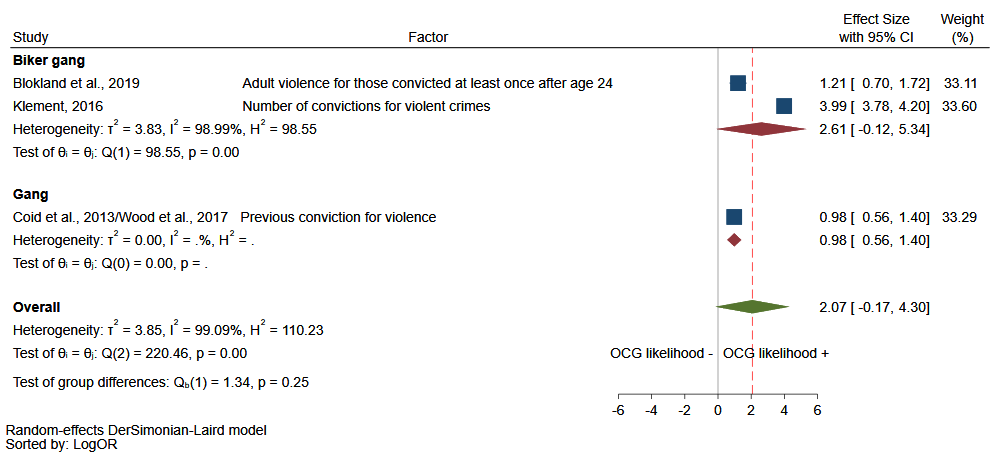

Supplement: Supplementary file 1 — Supporting information. [file CL2-18-e1218-s001.docx]
